# Cabotegravir and rilpivirine for treatment of HIV infection in Africa: week 96 results from the phase 3b randomized, open-label, noninferiority CARES trial

**DOI:** 10.1038/s41591-025-04041-7

**Published:** 2025-11-04

**Authors:** Cissy Kityo, Ivan K. Mambule, Joseph Musaazi, Simiso Sokhela, Henry Mugerwa, Ibrahim Yawe, Fiona Cresswell, Abraham Siika, Josphat Kosgei, Reena Shah, Logashvari Naidoo, Kimton Opiyo, Caroline Otike, Karlien Möller, Max Okwero, Charity Wambui, Veerle Van Eygen, Perry Mohammed, Fafa Addo Boateng, Nicholas I. Paton

**Affiliations:** 1 Joint Clinical Research Centre, Kampala, Uganda; 2 Infectious Diseases Institute, Kampala, Uganda; 3 Ezintsha, Faculty of Health Sciences, University of the Witwatersrand, Johannesburg, South Africa; 4 Joint Clinical Research Centre, Fort Portal, Uganda; 5 London School of Hygiene and Tropical Medicine, London, UK; 6 Centre for Global Health and Infection, Brighton and Sussex Medical School, Brighton, UK; 7 Department of Medicine, Moi University School of Medicine, Eldoret, Kenya; 8 Kenya Medical Research Institute/US Army Medical Research Directorate, Kericho, Kenya; 9 Aga Khan University Hospital, Nairobi, Kenya; 10 Chatsworth Clinical Research Site, South African Medical Research Council, Durban, South Africa; 11 Janssen Research And Development, Beerse, Belgium; 12 Johnson and Johnson, High Wycombe, UK; 13 Johnson and Johnson Middle East FZ LLC, Accra, Ghana; 14 Infectious Diseases Translational Research Programme and Department of Medicine, Yong Loo Lin School of Medicine, National University of Singapore, Singapore, Singapore; 15 Present Address: ViiV Healthcare, Ltd., London, UK

**Keywords:** Translational research, Randomized controlled trials

## Abstract

Evaluation of the durable efficacy and safety of long-acting injectable therapy for HIV is needed in African populations. In a multicenter, open-label phase 3b trial, 512 African adults with HIV-1, stable on first-line oral therapy, with screening plasma viral load (VL) <50 copies ml^−1^ and without past virologic failure were randomized (1:1) to continue oral therapy or switch to cabotegravir (600 mg) and rilpivirine (900 mg) intramuscular injections every 8 weeks (optional 4-week oral lead-in). VL was monitored every 24 weeks. Here the primary outcome for our analysis up to 96 weeks was VL <50 copies ml^−1^, using the Food and Drug Administration snapshot algorithm (noninferiority margin 10%) in the intention-to-treat exposed population. At 96 weeks, 247/255 (97%) in the long-acting group and 250/257 (97%) in the oral therapy group had VL <50 copies ml^−1^ (difference −0.4%; 95% confidence interval −3.1% to 2.0%), demonstrating noninferiority. Adverse events of severity grade ≥3 occurred in 41/255 (16%) in the long-acting group and in 22/257 (9%) in the oral therapy group, mostly considered unrelated to the study drug; only one treatment-related adverse event in the long-acting group led to a decision to discontinue treatment (injection-site abscess). Cabotegravir and rilpivirine long-acting therapy produced durable virologic suppression, met the prespecified noninferiority endpoint compared with oral therapy and demonstrated an acceptable safety and tolerability profile. Long-acting therapy may be considered for use in African treatment programs. PACTR registration: 202104874490818.

## Abstract (web summary)

In this follow-up study presenting results at 96 weeks, cabotegravir and rilpivirine long-acting therapy remained noninferior to oral therapy, had acceptable safety and tolerability profiles, and resulted in durable virologic suppression, further supporting its consideration for African treatment programs.

## Main

The World Health Organization (WHO) recommends a first-line regimen comprising a combination of three drugs taken as daily oral therapy; tenofovir disoproxil fumarate and lamivudine (nucleoside reverse-transcriptase inhibitors, NRTIs) and dolutegravir (an integrase strand-transfer inhibitor, INSTI)^1^. This regimen demonstrated good viral suppression in African trials^2^ and forms the anchor of the WHO public health approach, in which a small number of standardized regimens are administered with simplified monitoring and clinical management^3^.

A two-drug regimen of cabotegravir (an integrase inhibitor) and rilpivirine (a non-NRTI, NNRTI) given by intramuscular injection once every 4 or 8 weeks, has been shown to maintain viral suppression and to increase treatment satisfaction in participants switching from standard therapy in registrational trials done mainly in Europe and North America^4–7^. Such long-acting therapy may also be a valuable alternative to standard oral therapy in treatment programs in Africa, but evidence is currently limited to one trial (this trial), which reported successful outcomes at 48 weeks^8^. Longer-term follow-up is needed to establish the durability of this regimen in a setting typical of the public health approach and in a population with a high proportion of women and Black participants, extensive prior exposure to NNRTI-containing regimens, and viral subtypes common in Africa.

Here, we report the comprehensive 96-week efficacy and safety findings of long-acting cabotegravir and rilpivirine within a public health approach.

## Results

### Participant disposition

**Fig. 1:**
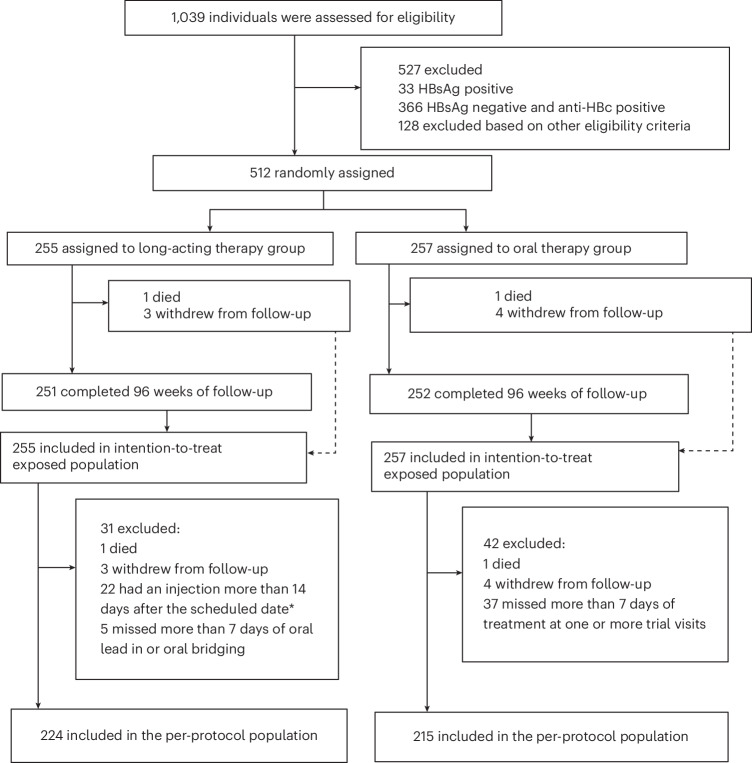
CONSORT diagram. HBsAg, hepatitis B surface antigen; anti-HBc, antibody against hepatitis B virus core antigen. *For assessing eligibility for per-protocol population, the duration of the injection delay excludes days when oral bridging was used, if any. Figure adapted with permission from ref. ^8^, Elsevier.

A total of 1,039 participants were screened for eligibility at 8 sites in Uganda, Kenya and South Africa commencing 1 September 2021, and 512 were enrolled into the trial and randomized between 15 September 2021 and 31 August 2022. Eligible participants were required to have been taking a regimen of tenofovir, lamivudine (or emtricitabine) and either dolutegravir, efavirenz or nevirapine, without past history of virological failure and to have a screening viral load below 50 copies ml^−1^ (Fig. 1). All participants took at least one dose of their randomly assigned study medication and were included in the intention-to-treat exposed population. Seven (1%) of 512 withdrew from follow-up, and 2 died before week 96 (Fig. 1). One protocol deviation was classified as critical (delayed review of laboratory report in a participant with reduced hemoglobin of grade 4 severity), and 43 were classified as major (the majority due to use of an outdated version of the consent form); none was judged to have affected participant outcomes in the trial.

Baseline characteristics were broadly similar between randomized groups, with 380 (74%) having prior NNRTI exposure (Table 1). Proviral DNA testing from peripheral blood mononuclear cells yielded a sequence in reverse transcriptase, integrase or both in 433 participants. Of these, 236 (55%) of 433 had subtype A1 virus (none had subtype A6 virus); 30 (7%) of 401 with a reverse transcriptase sequence had archived rilpivirine resistance mutations; 20 (10%) of 202 with an integrase sequence had archived cabotegravir resistance mutations (Table 1 and Extended Data Tables 1–3); and 32 of 401 (8%) had archived NRTI resistance (Supplementary Table 1). Overall, 108 (21%) had obesity at trial entry (Table 1). Of the 401 with complete data available on the presence or absence of the three putative baseline risk factors for subsequent virological failure that have been identified in previous studies (that is, subtype A1/A6 virus, rilpivirine resistance mutations and obesity), 221 (55%) had one factor and a further 52 (13%) had more than one of these factors present (Extended Data Table 4).

**Table 1:** Baseline characteristics of participants

Characteristic	Long-acting therapy (*n* = 255)	Oral therapy (*n* = 257)	Overall (*N* = 512)
Sex, no. (%)			
Female	146 (57)	149 (58)	295 (58)
Male†	109 (43)	108 (42)	217 (42)
Median age (IQR), years	43 (36–51)	42 (35–49)	42 (35–51)
Age group, no. (%)			
18–34 years	52 (20)	63 (25)	115 (22)
35–49 years	127 (50)	130 (51)	257 (50)
≥50 years	76 (30)	64 (25)	140 (27)
Country of residence, no. (%)			
Uganda	121 (47)	123 (48)	244 (48)
Kenya	78 (31)	84 (33)	162 (32)
South Africa	56 (22)	50 (19)	106 (21)
Black race, no. (%)^‡^	254 (100)	256 (100)	510 (100)
Median BMI (IQR), kg m^−^^2^	25.4 (21.5–29.5)	25.8 (22.3–29.0)	25.5 (21.9–29.2)
Obesity, no. (%)^§^	57 (22)	51 (20)	108 (21)
Median CD4^+^ cell count (IQR), mm^−^^3^	702 (513–882)	725 (561–898)	707 (536–888)
HIV-1 viral load ≥50 copies ml^−1^, no. (%)^¶^	5 (2)	10 (4)	15 (3)
Viral subtype A1, no. (%)^ǁ^	116/218 (53)	120/215 (56)	236/433 (55)
Rilpivirine resistance mutations, no. (%)**	14/208 (7)	16/193 (8)	30/401 (7)
Rilpivirine intermediate/high-level resistance, no. (%)^††^	4/208 (2)	8/193 (4)	12/401 (3)
Cabotegravir resistance mutations, no. (%)**	8/99 (8)	12/103 (12)	20/202 (10)
Cabotegravir intermediate/high-level resistance, no. (%)^††^	3/99 (3)	2/103 (2)	5/202 (2)
Median time on first-line ART (IQR), years	8 (4-13)	7 (4-13)	8 (4-13)
Previous exposure to NNRTI, no. (%)	189 (74)	191 (74)	380 (74)
Regimen at trial entry (screening), no. (%)			
Integrase inhibitor-containing regimen	231 (91)	240 (93)	471 (92)
NNRTI-containing regimen	24 (9)	17 (7)	41 (8)

Date are *n* (%), *n*/*N* (%) or median (IQR). The table presents characteristics for the intention-to-treat exposed population. IQR denotes interquartile range. Data previously published in ref. ^8^; updated with additional viral subtype and resistance results, and with exclusion of APOBEC context drug resistance mutations occurring on sequences affected by APOBEC editing.

^†^Sex was as assigned at birth. Information on gender identity was not collected.

^‡^One participant in the long-acting therapy group is white, and one in the oral therapy group is Asian.

^§^Obesity is defined as BMI ≥30 kg m^−^^2^.

^¶^Viral load range 52–2,855 copies ml^−1^ at baseline (all had viral load <50 copies ml^−1^ at screening).

^ǁ^Sequencing performed on archived viral DNA extracted from peripheral blood mononuclear cells stored at baseline. Subtype determined by comparison of sequence to the Los Alamos National Laboratory HIV sequence database; primary determination made from reverse transcriptase sequence, and integrase sequence where reverse transcriptase not available. No subtype A6 was identified.

**Resistance mutations listed in the 2022 edition of the International Antiviral Society-USA drug resistance mutations list. Drug resistance mutations listed as APOBEC-context drug resistance mutations in the Stanford HIV drug resistance database were disregarded if they occurred on a sequence with at least one signature APOBEC mutation. Rilpivirine resistance mutations disregarded were E138K and M230I. Cabotegravir resistance mutations discarded were G118R, E138K, G140R and R263K.

^††^Susceptibility determined using Stanford risk algorithm.

Trial outcomes reported below are viral load <50 copies ml^−1^ at week 96 (the primary outcome for this analysis); confirmed virological failure (two consecutive values ≥200 copies ml^−1^) by week 96; key secondary outcome); viral load ≥50 copies ml^−1^, <200 copies ml^−1^ and confirmed virological failure with new genotypic drug resistance mutation by week 96 (secondary outcomes); grade 3 or higher incident adverse events, serious adverse events, human immunodeficiency virus (HIV) disease progression events, events leading to discontinuation of treatment and injection-site reactions (safety outcomes); change from baseline to week 96 in CD4^+^ cell count, weight, body mass index (BMI), body composition by dual-energy X-ray absorptiometry (DEXA), blood pressure, laboratory metabolic and safety parameters, quality of life, treatment satisfaction; incident obesity, hypertension, diabetes or hyperlipidemia to week 96; and treatment preference at week 96 (exploratory outcomes). All of these outcomes are presented in this Article.

### Treatment selection and adherence

**Extended Data Fig. 1:**
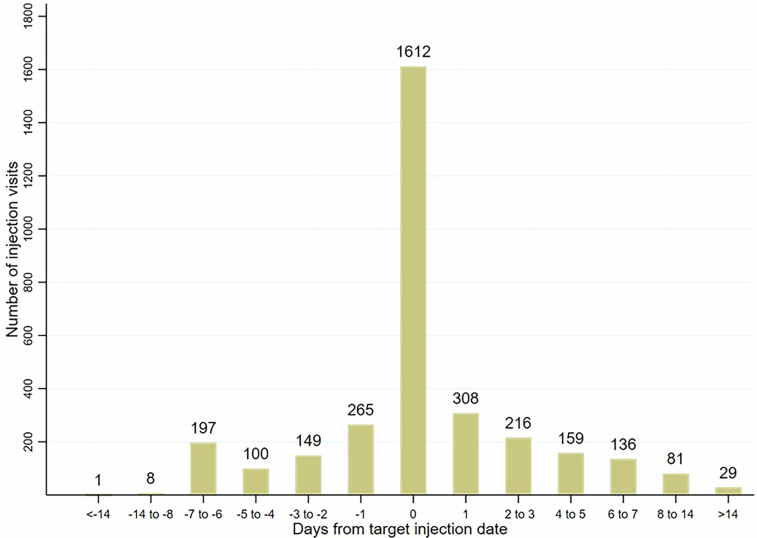
Timing of injections in the long-acting therapy group. Timing of injections in 255 participants in the long-acting group in relation to the target date, until the time of withdrawal from the trial (3 participants), death (1 participant) or switch to standard treatment (for virological failure in 3 participants and adverse events in 2 participants). The target date for all injections was set at the time of randomisation (4, 8, 16, 24, 32, 40, 48, 56, 64, 72, 80, 88, and 96 weeks from randomisation). For one participant who interrupted all ART at day 7 (due to incarceration) and had the first injection day at day 125 (delay of 97 days), the target date was determined from the time of the first injection day. For 10 additional participants at one site who had injection delays of more than 14 days, from the time of each injection delay the target date for each subsequent injection was determined from the date of last injection plus 56 days (plus 28 days if the initial injection was delayed); with the target reset using the same principle at the time of each subsequent injection. There were 29 injections that were delayed > 14 days from the target date (occurring in 25 participants; 4 participants had two injections delayed > 14 days, none of which were consecutive injections). The interval from the target date to each delayed injection was 15 to 21 days for 20 injections; 22 to 28 days for 7 injections and > 28 days for 2 injections. Oral lead in with cabotegravir and rilpivirine was extended by 16 days in 1 participant to cover a period of travel before the initial injection; oral bridging was used to cover periods of travel in 2 participants when return to the site was not possible (one with cabotegravir and rilpivirine for 28 days and one with dolutegravir and rilpivirine for 19 days); and oral bridging was used to replace one injection to allow resolution of an injection site abscess in one participant (cabotegravir and rilpivirine for 56 days). Of the 25 participants with one or more injections delayed > 14 days from the target date, 22 had viral loads < 50 copies/ml at all measurement time points; three had a single measured viral load ≥ 50 copies/ml at one timepoint (66, 108 and 381 copies/ml) and all had VL < 50 copies/ml at week 96.

In those assigned to long-acting therapy, 214 (84%) of 255 participants chose to switch to oral rilpivirine and cabotegravir for 4 weeks before first injection; 25 (10%) had an injection administered more than 14 days after the scheduled date on one or two (at maximum) occasions (Extended Data Fig. 1), all of whom had viral load <50 copies ml^−1^ at week 96; 2 (1%) took oral bridging therapy for 8 weeks when planned overseas travel prevented return to the site; and 1 (<1%) changed from long-acting to standard oral treatment for an adverse event (reported below). In the oral therapy group, 239 (93%) of 257 participants took tenofovir disoproxil fumarate, lamivudine and dolutegravir (Supplementary Table 2); 37 (14%) of 257 missed more than 7 days of treatment at one or more visits (there were no missed doses at 83% of participant visits); and 7 (3%) changed oral drugs in the regimen (Table 4).

### Primary and secondary efficacy outcomes

**Fig. 2:**
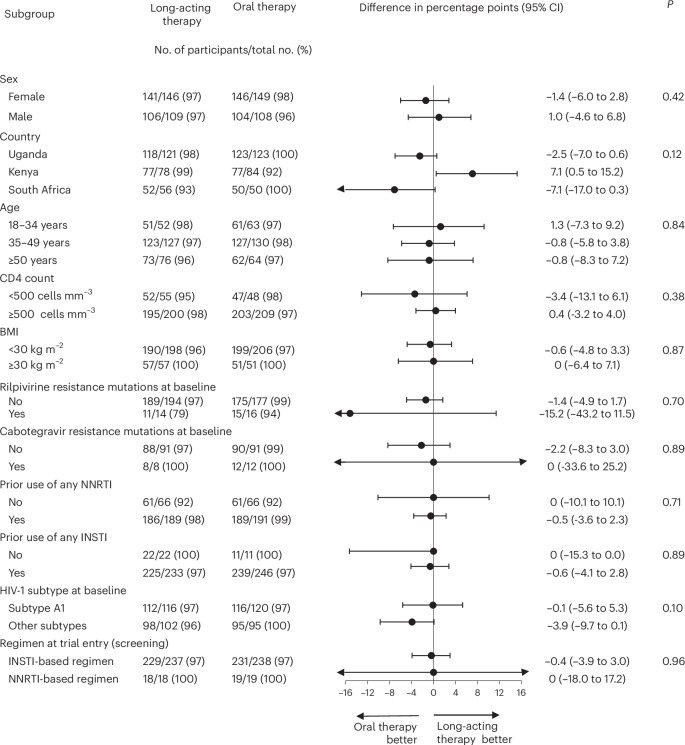
Subgroup analysis of viral suppression in the long-acting and oral therapy groups. Proportion of participants with viral suppression below 50 copies per milliliter at week 96, as classified by the FDA snapshot algorithm, shown by randomly assigned treatment group and prespecified subgroups. The point estimate for each subgroup represents the weighted risk difference between the proportion with viral suppression in each treatment group, and the error bars represent the 95% CI for those risk differences. These estimates are obtained using Cochran–Mantel–Haenszel-weighted Miettinen and Nurminen method, without adjustment for third-drug class, including all participants with data available for that subgroup classification. The widths of the CIs have not been adjusted for multiple comparisons and cannot be used to infer treatment effects. The *P* value is for a two-sided test of homogeneity using the Cochran–Mantel–Haenszel method; a *P* value of less than 0.05 may indicate evidence of heterogeneity across levels of a stratum, although the threshold has not been adjusted for multiple testing and cannot be used to infer differences in treatment effect between subgroups.

In the intention-to-treat exposed population, viral load <50 copies ml^−1^ was found in 247 (97%) of 255 participants in the long-acting therapy group and 250 (97%) of 257 in the oral therapy group (difference −0.4 percentage points; 95% confidence interval (CI) −3.1 to 2.0), meeting the prespecified noninferiority criterion (Table 2). Findings were generally similar across subgroups. In the small subgroup with baseline rilpivirine resistance mutations, the proportion with viral suppression <50 copies ml^−1^ appeared to be lower in the long-acting therapy (11 (79%) of 14) versus oral therapy group (15 (94%) of 16; difference 15.2 (95% CI −43.2 to 11.5) percentage points; *P* = 0.71 for heterogeneity of treatment response by baseline resistance mutations; Fig. 2). However, one of the three participants in the long-acting therapy group and the sole participant in the oral therapy group who were classified as not having viral suppression were so classified because they had no viral load measurement at week 96. Both participants had withdrawn from the trial before week 96 at a time when they both had viral suppression.

**Table 2:** Main efficacy outcomes at week 96

Outcome	Long-acting therapy (*n* = 255)	Oral therapy (*n* = 257)	Difference (95% CI)*, percentage points	Noninferiority margin (%)
**Primary outcome**				
HIV-1 viral load level, no. (%)				
<50 copies ml^−1^^†^	247 (97%)	250 (97%)	−0.4 (**−3.1** to 2.0)	**–**10%
≥50 copies ml^−1^^‡^	4 (2%)	2 (1%)	0.8 (−0.7 to 3.2)	–
No virological data^§^	4 (2%)	5 (2%)	–	–
**Primary outcome, sensitivity analyses**				
HIV-1 viral load level, no. (%)				
<50 copies ml^−1^ (additional adjustment)^¶^	97%	97%	−0.4 (−3.4 to 2.5)	–
<50 copies ml^−1^ (per protocol)^‖^	220/224 (98%)	214/215 (>99%)	−1.3 (−4.2 to -0.1)	–
<50 copies ml^−1^ (complete case)	247/251 (98%)	250/252 (99%)	−0.8 (−3.3 to 0.7)	–
**Secondary and other efficacy outcomes**				
HIV-1 viral load <200 copies ml^−1^, no. (%)	249 (98%)	251 (98%)	−0.01 (−2.4 to 2.4)	–
Confirmed virological failure, no. (%)**	4 (2%)	0	1.6 (0.4 to **4.2**)	4%
Confirmed virological failure (per protocol), no. (%)^‖^	4 (2%)	0	1.6 (0.4 to 4.2)	–
Confirmed virological failure with ≥1 major acquired resistance mutation, no. (%)	3 (1%)	0	–	–
CD4^+^ cell count change from baseline, cells mm^−^^3^^††^	−7 ± 213	18 ± 228	−25 (−64 to 13)	–

Data are *n* (%) or *n*/*N* (%) unless otherwise specified. All analyses of viral load suppression above or below threshold use the FDA snapshot algorithm and are done in the intention-to-treat exposed population, except where stated. Analyses of confirmed virological failure are done in the intention-to-treat exposed population unless otherwise stated. Analysis of change in CD4^+^ cell count uses complete case analysis. The widths of CIs have not been adjusted for multiplicity and cannot be used to infer treatment effects on secondary and other efficacy outcomes.

*Differences are expressed in percentage points (except for change from baseline in CD4 count, where differences are expressed in cells per mm³). Estimates of difference and 95% CI for primary and secondary virological outcomes used the Cochran–Mantel–Haenszel-weighted Miettinen and Nurminen method, adjusting for third-drug class (INSTI or NNRTI) at screening; except for sensitivity analysis (additional adjustment) that used modified Poisson regression with unbiased sandwich estimate of variance; and except for analysis of confirmed virological failure for which no adjustment for third-drug class was performed. Estimate of difference and 95% CI for CD4 cell count change used Student’s -test. Estimated differences are for the long-acting therapy group minus the oral therapy group. For the primary outcome (viral suppression <50 copies ml^−1^), noninferiority is met if the lower limit of the 95%CI for the difference (shown in bold) lies above the prespecified noninferiority margin (criterion met). For the key secondary outcome (confirmed virological failure), noninferiority is met if the upper limit of the 95% CI for the difference (shown in bold) lies below the prespecified noninferiority margin (criterion not met).

^†^In four participants from one site, the week 96 viral load test was delayed deliberately to coincide with a rescheduled injection visit (test done between weeks 103 and 109); to avoid a selective bias in one arm, these four viral load results were considered to be within the week 96 window period (prespecified as weeks 84 to 102).

^‡^Viral load ≥50 copies ml^−1^ is specified as a secondary outcome. Three of the viral load values ≥50 copies ml^−1^ at week 96 in the long-acting therapy group were values assigned due to earlier treatment switch for virological failure; all others were observed values from a test done during the week 96 window period.

^§^Of the nine participants with no virological data within the window period, seven withdrew before week 96 for reasons other than adverse events and two died.

^¶^Adjusted for third-drug class (INSTI or NNRTI) at screening, site and sex.

^‖^The per-protocol population excludes four participants in the long-acting therapy group and five in the oral therapy group who withdrew from the trial or died before week 96; 22 in the long-acting group with injection more than 14 days after the target date without oral bridging and a further five who missed more than 7 days of oral treatment; and 37 in the oral treatment group who missed more than 7 days of treatment at one or more visits.

**Confirmed virological failure is defined as two consecutive viral load values ≥200 copies ml^−1^. One participant in the long-acting group had a viral load of 44,984 copies ml^−1^ at week 48 that could not be confirmed (participant died 9 days after the visit, before viral load test could be repeated, following an operation for strangulated umbilical hernia). The level of viremia and accompanying resistance profile were considered to have precluded resuppression on long-acting therapy, if a confirmatory viral load had been performed.

^††^CD4^+^ cell count data were available for 501 participants; missing values were due to withdrawal or death before week 96 (four in the long-acting group, five in the oral therapy group) and missed sample collection (one in each group).

Noninferiority was also demonstrated in sensitivity analyses using the per-protocol population, as well as in secondary analyses examining outcomes of viral nonsuppression (≥50 copies ml^−1^) and suppression (<200 copies ml^−1^) (Table 2).

### Virological failure and resistance

Confirmed virological failure (one without a confirmatory viral load test, but with high-level resistance that would have precluded resuppression had a confirmatory test been performed) occurred in 4/255 (2%) participants in the long-acting therapy group and in no participants in the oral therapy group (difference 1.6 percentage points; 95% CI 0.4 to 4.2). This outcome did not meet the prespecified noninferiority criterion (Table 2). There was no difference in the CD4^+^ cell count change to week 96 between the long-acting and oral therapy groups.

Of the four participants with confirmed virological failure in the long-acting therapy group, three (participants 1, 2 and 4) had viral sequences available at time of failure (Table 3). All three had new resistance mutations to both rilpivirine (predicted to confer low-, intermediate- and high-level resistance in one participant each) and cabotegravir (predicted to confer intermediate-level resistance in one and high-level resistance in two participants). Participant 1 had potential low-level, participant 2 high-level and participant 4 low-level predicted dolutegravir resistance. Following a switch to standard-of-care treatment (dolutegravir with tenofovir disoproxil fumarate and lamivudine), participants 1, 3 and 4 achieved viral resuppression to <50 copies ml^−1^ by week 96; participant 2 died (of an unrelated cause) before therapy could be switched.

**Table 3:** Characteristics of the four participants with virological failure

	Participant 1	Participant 2	Participant 3	Participant 4
**Injection adherence and virological failure**				
Time in trial at virological failure, weeks	48	48	72	72
Delayed injections (>7 days from target), *n* (%)	0	0	0	0
Viral load, copies ml^−1^	8608; 1612	44984; no repeat	798; 563	259; 16161
Rilpivirine resistance mutations*	V108I, E138K	K103N/S, V106V/A, E138A, M230M/L	Test failed	E138A
Rilpivirine resistance level^†^	Intermediate	High	Test failed	Low
Cabotegravir resistance mutations*	E92E/V, N155H, L74M	G118R	Test failed	Q148R (M50I)
Cabotegravir resistance level^†^	Intermediate	High	Test failed	High
Dolutegravir resistance level^†^	Potential low	High	Test failed	Low
NRTI mutations*	Nil	Nil	Nil	Nil
Outcome after failure	Viral load resuppressed on TLD	Died (unrelated cause)	Viral load resuppressed on TLD	Viral load resuppressed on TLD
**Baseline characteristics and treatment course**				
Sex	Female	Male	Male	Male
BMI, kg m^−^^2^	25.9	22.0	22.2	19.9
Duration of prior antiretroviral therapy, months	24	28	33	12
Prior exposure to NNRTI	No	No	Yes	No
Antiretroviral therapy regimen at baseline	TLD	TLD	TLD	TLD
Viral subtype^‡^	A1	D	A1	C
Rilpivirine resistance mutations^§^	Nil	K103N/S, E138A	E138A	Nil
Rilpivirine resistance level^†^	Nil	Low	Low	Nil
Cabotegravir resistance mutations^§^	L74M	Nil	Test failed	Nil
Cabotegravir resistance level^†^	Low	Nil	Test failed	Nil

TLD, tenofovir, lamivudine and dolutegravir.

*Resistance mutations listed in the 2022 edition of the International Antiviral Society-USA drug resistance mutations list.

^†^Susceptibility determined using Stanford risk algorithm

^‡^Baseline subtype was determined from sequencing of DNA extracted from peripheral blood mononuclear cells. Subtype was identified from reverse transcriptase sequence and determined by reference to the Los Alamos National Laboratory HIV Sequence Database.

^§^Baseline resistance mutations were determined from sequencing of DNA extracted from peripheral blood mononuclear cells. Resistance mutations listed in the 2022 edition of the International Antiviral Society-USA drug resistance mutations list. None of the baseline sequences had APOBEC mutations.

Two participants (2%) of 116 with subtype A1 versus two (2%) of 102 with subtype other than A1 (subtypes C and D in one participant each) had virological failure in the long-acting therapy group (1% of 136 and 2% of 119, respectively, estimated in the full population switching to long-acting therapy). Two participants (14%) of 14 with rilpivirine resistance mutations on retrospective sequencing of DNA stored at baseline (predicted to confer low level resistance in both cases) versus two (1%) of 194 without rilpivirine resistance mutations at baseline had virological failure in the long-acting therapy group (12% of estimated 17 with and 1% of estimated 238 without rilpivirine mutations in the full population switching to long-acting therapy). None of those with obesity at baseline had virological failure in the long-acting therapy group.

### Safety

Adverse events of grade 3 or greater severity occurred in 41/255 (16%) participants in the long-acting therapy group and 22/257 (9%) in the oral therapy group (difference 7.5%, 95% CI 1.9% to 13.2%); 5 and 4 events, respectively, were considered related to a study drug (Table 4 and Supplementary Table 3). Serious adverse events occurred in 12 participants in the long-acting therapy and nine in the oral therapy group; none was considered related to the study drug (Table 4 and Supplementary Tables 4 and 5). HIV disease progression events occurred in two participants in each group (Table 4). Injection-site reactions were reported by 197 (77%) participants on long-acting therapy; mostly pain and of grade 1–2 severity (Extended Data Table 5) with only one (injection-site nodule) that was grade 3; and one (injection-site sterile abscess) that led to a decision to discontinue treatment (Table 4).

**Table 4:** Adverse events occurring between baseline and week 96

	Long-acting therapy (*n* = 255)	Oral therapy (*n* = 257)	Difference (95% CI)*
**Participants with at least one adverse event grade** ≥**3, no. (%)**			
Any	41 (16)	22 (9)	7.5 (1.9 to 13.2)
Related to study drug^†^	5 (2)	4 (2)	0.4 (−1.9 to 2.7)
**Participants with at least one serious adverse event, no. (%)**			
Any	12 (5)	9 (4)	1.2 (−2.2 to 4.6)
Related to study drug	0		–
**Participants with at least one HIV disease progression event, no. (%)**			
Any	2	2	–
Death^‡^	1	1	–
AIDS-defining event	0	0	–
Serious non-AIDS event^‡^	1	2	–
**Participants with at least one adverse event of any grade, no. (%)**			
Any	230 (90)	185 (72)	18.2 (11.6 to 24.8)
Leading to study drug discontinuation^¶^	2 (1)	5 (2)	−0.8 (−2.6 to 1.1)
Excluding injection-site reactions	202 (79)	185 (72)	7.2 (−0.2 to 14.6)
Injection-site reactions	197 (77)	0	–

Data are *n* (%). The table presents the number of participants with at least one adverse event in each event category.

*Differences are expressed in percentage points. Estimates of difference and 95% CI used the normal approximation to the binomial method.

^†^Grade ≥3 adverse events considered at least possibly related to study drug were injection-site nodule in one participant, increased low-density lipoprotein cholesterol in two participants, proteinuria in one participant and increased blood triglycerides in one participant in the long-acting group; and decreased estimated glomerular filtration rate (eGFR) in two participants, increased aspartate aminotransferase in one participant and increased blood glucose in one participant in the standard oral therapy group.

^‡^Death was due to postoperative complications of surgery for strangulated umbilical hernia for the participant in the long-acting group; and due to encephalopathy associated with parapharyngeal squamous cell carcinoma for the participant in the oral therapy group. Serious non-AIDS events were lung adenocarcinoma in one participant in the long-acting group; and parapharyngeal squamous cell carcinoma (fatal) and prostate adenocarcinoma in one participant each in the oral therapy group.

^¶^Adverse events leading to study drug discontinuation were injection-site sterile abscess and lung adenocarcinoma each in one participant in the long-acting treatment group (switched to standard oral treatment); osteoporosis in two participants (one switched tenofovir to abacavir and the other switched tenofovir to zidovudine), decreased eGFR in one participant (switched tenofovir to abacavir), increased blood glucose in one participant (switched dolutegravir to efavirenz) and increased aspartate aminotransferase in one participant (switched dolutegravir to efavirenz) in the standard oral therapy group. In addition, two participants in the standard oral therapy group with low eGFR at baseline switched tenofovir to abacavir during follow-up to prevent further decline in eGFR.

### Exploratory outcomes

CD4 count was stable, with no difference between treatment groups (Table 2).

**Extended Data Fig. 2:**
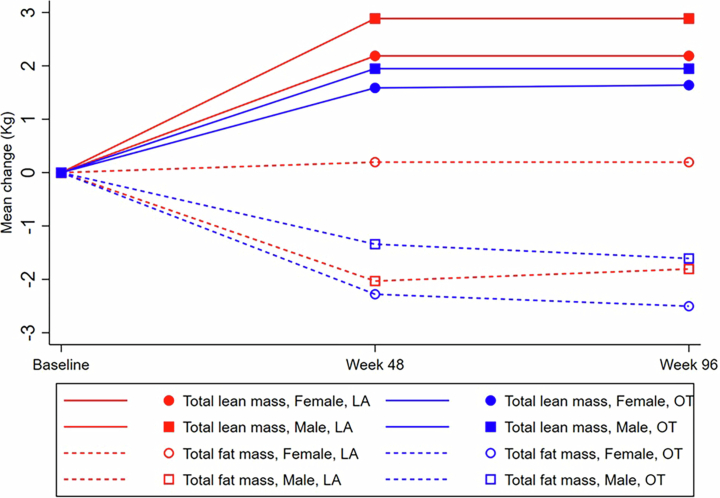
Changes in total fat mass and total lean mass measured by DEXA. Change in fat and lean mass from baseline to week 48 and week 96 in complete cases. Values shown are mean change within each treatment group, by sex.

Women in the long-acting therapy group had greater increase in body weight, BMI and higher rate of incident obesity compared with those in the oral therapy group (Extended Data Tables 6 and 7 and Supplementary Fig. 1); there were no differences in these outcomes between treatment groups in men. In the DEXA substudy, women in the long-acting therapy group had a relative increase in trunk fat mass (adjusted difference 1.01 (0.18 to 1.84) kg, *P* = 0.018); as well as a relative increase in limb fat mass and overall fat mass, all in comparison with the oral therapy group (Extended Data Table 8). However, this relative increase in fat was driven by the combination of stable fat mass in women in the long-acting group in contrast to fat loss in the oral therapy group (Extended Data Fig. 2). There were no differences in fat mass between treatment groups seen in men, or differences in lean mass between treatment groups (in either sex). Women in the long-acting therapy group were more likely to start new antihypertensive medication (12% versus 3%), despite no observed difference between treatment groups in measured blood pressure or incident hypertension (Supplementary Table 6). They also experienced a greater rise in total cholesterol and triglycerides, with more frequent cases of incident increased cholesterol (15% versus 4%) and hyperlipidaemia (18% versus 3%) compared with women in the oral therapy group (Supplementary Table 7). In men, the only difference between treatment groups observed in these hypertension and lipid outcomes was a greater rise in cholesterol in those in the long-acting therapy group (Supplementary Table 7). Estimated glomerular filtration rate increased and alkaline phosphatase decreased in the long-acting therapy group compared with the oral therapy group (in both sexes; Supplementary Table 8).

A total of 13 pregnancies occurred during the 96-week study period: three pregnancies among two women in the long-acting therapy group (resulting in one healthy live birth, one miscarriage and one anembryonic pregnancy), and ten pregnancies among nine women in the oral therapy group (resulting in seven healthy live births, two miscarriages and one elective abortion).

### Person-centered outcome measures

Overall quality of life was high and similar between groups (Supplementary Tables 9 and 10). Treatment satisfaction score increased more from baseline to week 96 in the long-acting therapy group compared with the oral therapy group (adjusted mean difference 9.6 percentage points; 95% CI 7.5 to 11.8; *P* < 0.0001; Supplementary Table 11). Of the 244 participants in the long-acting therapy group who provided an indication of their treatment preference at week 96, 243 (>99%) responded that they preferred long-acting therapy over oral therapy. The sole participant who responded that they preferred oral therapy indicated that this was driven by a desire to access oral treatment from a local clinic to avoid lengthy travel distance.

## Discussion

We found that switching from standard oral therapy to 8-weekly injections of cabotegravir and rilpivirine maintained viral suppression in 97% of participants at 96 weeks, demonstrating noninferiority to continued standard oral therapy. This extends our earlier finding of noninferiority at 48 weeks, providing the necessary evidence of durability required for this intervention to be considered for use in HIV program settings. This trial represents the definitive evaluation of long-acting therapy in a population representative of those receiving treatment in the public health approach in sub-Saharan Africa, with a majority of participants being women and with past exposure to NNRTIs; with sparse viral load monitoring; and with the comparison group taking tenofovir disoproxil fumarate, lamivudine and dolutegravir, the established standard of care in this setting (taken by few participants in the earlier registrational trials)^4–7^. This demonstration of noninferior virological suppression in comparison with standard of care is an essential first step towards establishing suitability of long-acting therapy in the public health approach. Although the trial did not meet the noninferiority criterion for confirmed virological failure, the proportion of participants with failure at 96 weeks (2%) was low and comparable to the 1% reported in previous trials of 4- to 8-weekly long-acting therapy with these agents^4–7^.

In addition to assessing performance on these standard virological outcomes, there are two additional attributes of any regimen that are especially important to consider in evaluating suitability for use in the public health approach. First, the regimen should have sufficient forgiveness to be able to maintain efficacy despite periods of nonadherence, in this case injection delays, typical of those that might occur in program settings. Regimen forgiveness is difficult to evaluate in a clinical trial that provides additional resources to implement visit reminders and to reimburse participants for expenses associated with attending visits. However, although the large majority of injections were given on schedule, 10% of participants had one to two injections delayed by more than 14 days from the target date. All these participants had viral suppression at week 96, providing some confidence that regimen efficacy may be maintained despite occasional injection delays lasting a few weeks. Forgiveness was not demonstrably worse than that of the standard treatment regimen when exposed to episodes of missed doses of longer than 7 days, which occurred in a similar proportion of participants. Ongoing trials in people with adherence challenges in the public health approach, such as IMPALA (NCT05546242), may provide further insight on this issue; and the incidence and impact of longer delays will need to be monitored in large-scale implementation studies.

Second, the regimen should have a sufficiently high genetic barrier to resistance to minimize the risk of jeopardizing available salvage regimens during virological failure. Compared with individualized care in resource-rich settings, this risk may be greater in the public health approach due to later detection of virological failure with accompanying prolonged resistance selection pressure. The therapeutic consequences of this risk of resistance selection are amplified in the public health approach by limited access to resistance testing and by a restricted range of drugs available to construct salvage regimens. The risk of transmission of drug resistance during failure is also an important consideration, although the impact of mutations on the fitness and transmissibility of viral strains is a complex consideration and may vary with viral subtype. Our trial design, with viral load monitoring only every 24 weeks, was designed to test the regimen under relevant programmatic conditions to better quantify the risk accompanying failure in a program setting. Our finding that all three participants in the long-acting therapy group who had a sequence available at virological failure had mutations predicted to confer intermediate-to-high-level resistance to cabotegravir (with accompanying predicted resistance to rilpivirine) is consistent with the high rates of cabotegravir resistance (44–75%) in participants with virological failure observed in previous switch trials, together suggesting that this combination regimen has a relatively low genetic barrier to resistance^9,10^. We did not observe any episodes of confirmed virological failure (or dolutegravir resistance) on the standard oral treatment regimen (tenofovir disoproxil fumarate, lamivudine and dolutegravir) during the trial, but numerous trials and extensive programmatic experience in first-line therapy have found that dolutegravir resistance is rare at the time of virological rebound, indicating that this regimen has a high genetic barrier to resistance^11^. Even when the standard regimen is used in the more challenging situation of second-line therapy, with resistance mutations present to both tenofovir and lamivudine, acquired mutations predicted to confer intermediate to high-level dolutegravir resistance are seen in only a minority (around 20%) of those who develop virological failure^11,12^, still below the level seen with long-acting cabotegravir and rilpivirine. However, what really matters is the loss of future treatment options—which, in the public health approach, can be defined as development of intermediate to high-level resistance to either dolutegravir or darunavir or both (given that development of tenofovir and lamivudine resistance does not prevent successful treatment with these two drugs in combination regimens that include dolutegravir or darunavir)^12^. A similar endpoint has been used previously for evaluating the clinically relevant risk of resistance associated with confirmed virological rebound in a long-term trial of a switch strategy^13^. Framed this way, the finding that, among the three participants with confirmed virological failure and predicted intermediate to high-level cabotegravir resistance, one had potential low-level and one had low-level predicted resistance to dolutegravir—consistent with preserved dolutegravir susceptibility seen in previous trials^5,7^—and that they were able to achieve re-suppression after switching to a standard oral regimen, suggests that the overall impact on future treatment options (for example, tenofovir, lamivudine, dolutegravir or darunavir-based regimens) following failure on cabotegravir and rilpivirine may be relatively limited. However, given the relatively short follow-up time in the trial following switch back to standard treatment, we were not able to assess the longer-term outcomes in these two participants (and in one other participant with failure without a sequence performed) who resuppressed on reintroduction of the standard oral regimen. This will also be an important aspect to monitor in any future implementation studies.

A screening algorithm is used in resource-rich settings to identify people with predicted higher risk of virological failure on long-acting therapy, in whom switch may be avoided. This algorithm identifies three factors associated with a higher risk of failure of long-acting therapy: viral subtype A6/A1 (later disaggregated, as explained below), baseline rilpivirine mutations and baseline obesity, with risks multiplied when more than one factor is present^14^. We did not perform real-time sequencing on archived DNA before switching to long-acting therapy, but the retrospective testing allows us to evaluate the potential value that such a selection step might add in a program setting. Our finding of absence of association between subtype A1 and virological failure provides the critical evidence to support a contemporary reinterpretation of the published prediction model that suggests risk is specifically associated with subtype A6 (rather than subtype A1 virus); and subtype A6 was not present in our population (consistent with its known very low prevalence in Africa). Thus, there appears to be minimal value in baseline sequencing to establish subtype before switch in this population. Our observation of a higher rate of virological failure in participants with baseline rilpivirine resistance mutations (estimated risk of 12%, consistent with the published prediction model) may at first appear to represent a compelling case for sequencing archived DNA in the public health approach before switch. However, based on our study population, testing would need to be performed in approximately 127 people to avoid one person developing virological failure on long-acting therapy; and for each person with baseline mutations at genuine risk of virological failure who did not switch to long-acting therapy, approximately 7 others would be denied, needlessly, the opportunity to switch to long-acting therapy. Furthermore, there are additional costs and technical challenges associated with sequencing of archived DNA that have received little attention in studies so far but may have important consequences for feasibility and interpretation, including the requirement for rigorous quality control;^15^ and the exclusion of APOBEC-associated mutations that are unlikely to be predictive of treatment failure (because the virus with which they are associated is rendered nonfunctional)^15,16^. In this analysis, in which we excluded APOBEC mutations, the prevalence of baseline rilpivirine resistance in the long-acting group was estimated as 7%, compared with the earlier estimate of 12% when these APOBEC mutations were retained^8^. Thus, if the prediction algorithm were applied in a public health setting without excluding APOBEC mutations, the number of individuals unnecessarily denied long-acting therapy would approximately double. Obesity, the third factor in the published model, was not associated with an increased risk of virological failure in our participants. This is despite the fact that longer needles, considered to increase the likelihood of successful intramuscular injection of long-acting therapy in those with obesity, were used inconsistently and in a minority of such participants, due to challenges with access in the participating countries. Overlap between baseline obesity and rilpivirine resistance at baseline is uncommon, indicating that the potential for synergistic interaction to increase risk of failure is small. Taken together, the low yield, low positive predictive value, high additional cost, challenges of performing and interpreting sequencing on proviral DNA and infrequent overlap with obesity suggests that routine testing of proviral DNA for resistance mutations would be unfeasible and unnecessary as a routine selection step to screen people before starting cabotegravir and rilpivirine long-acting therapy in the public health approach in this region. Furthermore, the high efficacy of long-acting therapy in this trial, in which baseline sequencing was not performed in real time, provides strong pragmatic evidence that it is not needed. However, our findings should not be taken to imply lack of utility of the prediction model in specific cases, in other regions or outside HIV program settings. The trial enrolled a high proportion of people with past exposure to NNRTI-containing regimens. Although those with known history of virological failure were excluded, some (as in program settings) may have had occult failure before programmatic switch to the standard tenofovir, lamivudine, dolutegravir regimen done without viral load testing, or may have chosen not to disclose their full treatment history. If there were compelling reasons to consider someone with definite past virological failure on an NNRTI-containing regimen for long-acting therapy, then a resistance test before switch might have more value. Similarly, testing of viral resistance and subtype before switch in regions where subtype A6 virus is common would also seem appropriate.

Long-acting therapy was well tolerated, as in previous trials, with only one grade 3 injection-site reaction and one adverse event leading to treatment discontinuation. Our finding that women in the long-acting therapy group experienced increases in weight and BMI, along with a higher incidence of new-onset obesity compared with women in the oral therapy group, is notable. Although substantial weight gain has been described in trials of dolutegravir given with nucleoside reverse transcriptase inhibitors in first-line therapy^2^, weight gain has been relatively modest in previous trials switching oral to long-acting therapy, with changes broadly comparable between the treatment groups^4,5,7,17^. This may be because female participants, in particular those from sub-Saharan Africa, who seem to be particularly prone to treatment-related weight gain, were not well represented in these earlier trials. DEXA measurement of body composition revealed that the weight gain in women in the long-acting therapy group largely comprised lean tissue. Indeed, lean tissue increased in all trial participants, in both sexes, which may reflect natural weight gain promoted by continued use of integrase inhibitors (with little difference between dolutegravir and its analog, cabotegravir). The minimal increase in fat (in trunk and limbs) in women on long-acting therapy together with the relative fat gain compared with those in the oral therapy group is explained by a decrease in fat mass in participants on oral therapy. This decrease in fat mass may be attributable to continued use of tenofovir, which is known to have weight-suppressive effects that may extend to selective effects on body fat. This is also consistent with the known lipid-lowering effects of tenofovir (which would also account for the marked increase in blood lipid measurements in those on long-acting therapy stopping tenofovir)^18^ and with activation of thermogenesis pathways in adipose tissue by tenofovir, recently demonstrated in a humanized mouse model of HIV, which may prevent lipogenesis^19^. It is possible that weight changes seen in women on long-acting therapy may have other adverse health consequences, and we noted an increased rate of starting antihypertensive drugs in this group^20^. An important safety concern with long-acting therapy is the risk of HBV reactivation in those with this co-infection. We excluded participants with hepatitis B surface antigen or antibody against hepatitis B virus core antigen (anti-HBc). The latter indicates past exposure but not necessarily active infection, and exclusion is a relatively conservative approach. Testing for hepatitis B surface antigen with exclusion only of those who are positive, and vaccination of those who are negative and not previously vaccinated, may exclude fewer people and enhance HBV protection.

The main limitation of the trial was the lack of blinding, which was not feasible due to the different routes of administration between the experimental and standard treatments. Open-label administration was necessary to assess participant acceptability of the intervention. This is unlikely to have biased the laboratory-based main outcome. Although participants were approached consecutively for participation in the DEXA substudy, more participants in the long-acting therapy than the oral therapy group had baseline scans, possibly because the more intensive initial visit schedule and contact in the long-acting therapy group increased opportunities to schedule scans within the requisite month from randomization. The potential for selection bias means that the DEXA comparison should be interpreted as observational data rather than as a strict randomized comparison. These trial findings from sub-Saharan Africa are not necessarily generalizable outside of this region, just as earlier studies from resource-rich settings could not be assumed to be generalizable to sub-Saharan Africa. However, the similarity of main findings across this and trials in other settings provide a high level of confidence that this regimen has global applicability.

This trial provides the essential scientific evidence to support discussions on the use of long-acting therapy in sub-Saharan Africa. This intervention has high treatment satisfaction, observed in this and all previous trials. Ultimately, uptake will depend on availability of sufficient and sustained funding, on the availability of drugs and on difficult decisions of what interventions to prioritize for the public health approach in resource-constrained settings. Following the initial results of this trial, the WHO has recommended long-acting therapy with cabotegravir and rilpivirine as an alternative switching option for adults and adolescents with undetectable HIV viral load on oral antiretroviral therapy and without active hepatitis B infection, thereby heralding a new era of long-acting HIV treatment in the public health approach^21^

## Methods

### Trial design and oversight

This was a prospective, multicenter, randomized, open-label, noninferiority, 96-week trial comparing switch to long-acting therapy versus maintaining daily oral therapy in adult participants with established virological suppression on standard oral therapy managed in sub-Saharan Africa. The trial was anticipated to enroll a high proportion of female participants reflecting the distribution of HIV infection in sub-Saharan Africa, and an analysis of the primary outcome by sex (as assigned at birth) was prespecified as a subgroup analysis. The trial was registered on the Pan African Clinical Trials Registry (PACTR, registration number 202104874490818, date of registration 16 April 2021). Trial protocol version 1 (22 November 2020) was amended on 11 July 2022, 16 June 2023 and 13 December 2023 to add a hepatitis B baseline serology substudy, a social science substudy and information on post-trial drug access, respectively, with otherwise minor changes to the protocol text for clarification purposes.

The study design and results of the primary outcome (week 48 viral load below 50 copies ml^−1^) have been previously reported^8^. The trial methods described in this Article are, by necessity, identical to those reported in the earlier report for the first 48 weeks of the trial^8^. They are repeated in summary here to enable comprehension of the trial and to comply with standard clinical trial reporting requirements. Additional detail of the methods can be found in the report of the primary outcome of the trial^8^ and in the trial protocol (Supplementary Information). The information on the follow-up assessments performed between week 48 and week 96, the description of the approach to identification and analysis of APOBEC-context drug resistance mutations in baseline proviral DNA (not performed in the earlier analysis), and the description of the conduct and analysis of DEXA scans were not presented in the earlier paper.

### Ethical approval

This trial complies with all relevant ethical regulations and was conducted in accordance with the trial protocol and the ethical principles originating in the Declaration of Helsinki. The trial protocol was approved by the ethics body responsible for each clinical research site (Joint Clinical Research Centre Research Ethics Committee and the Uganda National Council for Science and Technology, all in Uganda; Moi Teaching and Referral Hospital Institutional Scientific and Ethics Review Committee, Aga Khan University Institutional Scientific and Ethics Review Committee, and Kenya Medical Research Institute Scientific and Ethics Review Unit, all in Kenya; University of The Witwatersrand Johannesburg Human Research Ethics Committee, and South African Medical Research Council Human Research Ethics Committee, all in South Africa) and by national regulatory agencies responsible for reviewing and approving trials in the participating countries. All trial participants provided written informed consent.

### Ethics and inclusion statement

Researchers in participating countries were involved in the study design, study implementation, data ownership and authorship of publications. The study was designed to be relevant to programs following the WHO public health approach to antiretroviral therapy in sub-Saharan Africa, including locally relevant sparse viral load and safety monitoring. Roles and responsibilities were agreed among collaborators before starting the trial. The trial was managed by an institution in Uganda for the purposes of capacity-building, with external support from an experienced trialist as part of the trial management team. This research was conducted entirely in sub-Saharan Africa, in the setting of the researchers. The research did not result in stigmatization, incrimination, discrimination, or personal risk to participants, aside from the safety risks associated with antiretroviral therapy, which were mitigated through clinical monitoring and care provided during the trial. No biological materials have been transferred out of the participating countries, other than for the purpose of protocol-mandated resistance testing that was centralized in Uganda. Discussion of research findings includes a number of relevant citations of previous antiretroviral therapy trials conducted by sub-Saharan investigators and institutions participating in this trial.

### Trial population

Participants were enrolled from those attending for their routine clinical care at the trial site, or by referral from clinics within the surrounding areas. Screening and subsequent trial procedures were done only at the trial site. Eligible participants were at least 18 years of age; had taken a regimen of tenofovir, lamivudine (or emtricitabine) and either dolutegravir, efavirenz or nevirapine for at least 6 months continuously before screening; and had a viral load below 50 copies ml^−1^ at screening and at 4–6 months before screening. Women were eligible if they were not pregnant (confirmed by negative urine pregnancy tests at screening and baseline) or lactating; were not intending to become pregnant during the next year; and were either not of reproductive potential, or were postmenopausal, or were willing to take effective contraception before and for 52 weeks following discontinuation of long-acting therapy. Male participants were eligible if they were willing to wear a condom during sexual activity and if they agreed not to donate sperm for the purpose of reproduction during the study and for a minimum of 90 days following discontinuation of long-acting therapy. Participants with asymptomatic chronic hepatitis C virus infection were eligible if their liver enzyme levels met the entry criteria, they had undergone appropriate evaluation and they did not have advanced or unstable liver disease.

The exclusion criteria were having two consecutive viral load tests of ≥50 copies ml^−1^ in the 12 months before screening; history of virological failure (two consecutive viral load tests ≥200 copies ml^−1^) at any time; current participation in another intervention trial; active Centers for Disease Control and Prevention stage 3 disease (except cutaneous Kaposi’s sarcoma); active tuberculosis co-infection requiring antituberculous therapy; severe hepatic impairment or history of liver cirrhosis (with or without hepatitis viral co-infection); preexisting physical or mental condition (including substance abuse disorder and suicide risk) which the investigator considered may interfere with compliance with the dosing schedule or protocol evaluations or may compromise the safety of the participant; one or more seizures in the year before study entry, or unstable or poorly controlled seizure disorder, or assessed by the investigator to be at high risk of seizures; tattoo or other dermatological condition overlying the gluteus region; positive test for hepatitis B surface antigen or anti-HBc at screening; ongoing or clinically important medical conditions that, in the opinion of the investigator, may interfere with the absorption, distribution, metabolism or excretion of the study interventions or could affect participant safety; history of coagulopathies, or current or anticipated need for chronic anticoagulation (apart from low-dose aspirin); known major integrase inhibitor or NNRTI resistance-associated mutation (except for K103N) based on any historical resistance test result; any grade 4 laboratory abnormality; estimated creatinine clearance <50 ml min^−1^ per 1.73m^2^ via the CKD-EPI method; alanine aminotransferase no greater than three times the upper limit of normal; exposure to experimental drug or experimental vaccine within either 30 days, 5 half-lives of the test agent or twice the duration of the biological effect of the test agent, whichever is longer, before baseline; received treatment with radiation therapy, cytotoxic chemotherapeutic agents, tuberculosis therapy (with the exception of isoniazid), anticoagulation agents, immunomodulators (such as chronic systemic corticosteroids, interleukins or interferons) within 28 days of screening; or treatment with an HIV-1 immunotherapeutic vaccine within 90 days of screening; or treatment with any agent with documented activity against HIV-1 within 28 days of randomization (with the exception of antiretroviral therapy drugs used for standard treatment); treatment with any medication prohibited by the protocol and unwilling or unable to switch to an alternate medication; confirmed or suspected coronavirus disease 2019 infection or close contact with a person with known or suspected coronavirus disease 2019 infection. Additional definitions related to these criteria are listed in the protocol.

### Randomization and masking

Participants were randomly assigned (1:1 ratio) to switch to long-acting therapy or to stay on oral therapy. Randomization was conducted using a web-based system preprogrammed with a computer-generated list, using random permuted blocks and stratified by the third-drug class (integrase inhibitor or NNRTI) at screening. Randomization was performed by the study coordinator at each site, who could access the next number on the system but not the whole list.

The trial management team did not have access to aggregate unmasked data except for serious adverse events and pregnancy reports. The trial statistician had access to unmasked data through formal requests to the data management group when required for study analyses. Treatment allocation was not masked to site staff and participants.

### Treatment

Participants in the long-acting therapy group had the option to switch to 30 mg of cabotegravir and 25 mg of rilpivirine as daily oral therapy (to allow an assessment of tolerability) or continue their current regimen in the first 4 weeks after randomization. At weeks 4 and 8 and then every 8 weeks until week 96 (window of 7 days before or after the scheduled date), participants were scheduled to receive 600 mg of cabotegravir and 900 mg of rilpivirine by injection into the gluteus muscle. Use of longer needles was recommended but not mandated for participants with obesity. For preplanned occasions where the participant could not attend for scheduled injection within the window, a period of bridging with oral cabotegravir and rilpivirine was permitted. Participants in the oral therapy group took the WHO-recommended first-line regimen of 300 mg of tenofovir, 300 mg of lamivudine and 50 mg of dolutegravir as a fixed-dose combination once daily. Other regimens with dolutegravir or efavirenz in combination with NRTIs at the standard doses recommended in treatment guidelines were also permitted.

### Assessments and outcomes

Participants in the long-acting therapy group had scheduled trial visits at weeks 4 and 8, then every 8 weeks to week 96, coinciding with scheduled injections; and those in the oral therapy group at week 12, then every 12 weeks to week 96. Assessments at each visit included adherence (oral therapy) assessed by pill count and participant interview in the event of discrepancies; injection-site reactions (long-acting therapy); and adverse events. Body weight, measured on standard weighing scales, and blood pressure were recorded every 24 weeks. Standard safety blood tests (full blood count, sodium, potassium, creatinine, glucose, alanine aminotransferase, bilirubin and alkaline phosphatase) were done at weeks 4 (long-acting therapy), 12 (oral therapy), 16 (long-acting therapy) and 24, 48, 72 and 96 (both groups). Plasma lipid profile was done at weeks 48 and 96. Urine pregnancy tests were done at all visits in women of child-bearing potential. Clinical and laboratory adverse events were graded using standard criteria^22^

Viral load was monitored every 24 weeks up to week 96. For the long-acting therapy group, a viral load result ≥200 copies ml^−1^ prompted a repeat test after 4–6 weeks. For the oral treatment group, a higher threshold of ≥1,000 copies ml^−1^ was used, with a repeat test after 10–16 weeks. The higher threshold and longer retest interval allowed for adherence counseling, in line with WHO guidelines^1^. CD4 count was measured at weeks 24, 48 and 96.

Genotypic resistance testing was performed in real time by standard RNA testing if the retest viral load remained at or above the treatment-group-specific threshold (≥200 or ≥1,000 copies ml^−1^); and also at the end of the trial in all with viral load ≥200 copies ml^−1^. Genotypic resistance testing and determination of viral subtype was also done on archived proviral DNA extracted from peripheral blood mononuclear cells stored at baseline in all participants. DNA extraction and sequencing were done retrospectively in batches when participants had completed at least 48 weeks of trial follow-up; results were returned to clinicians upon request at the end of trial follow-up. Viral subtype was determined by reference to the Los Alamos National Laboratory Panel. The presence of drug resistance mutations in proviral DNA was determined by reference to the IAS-USA 2022 list^23^. These drug resistance mutations were then compared against the list of 14 APOBEC-context drug resistance mutations in reverse transcriptase or integrase listed in the Stanford HIV drug resistance database (RT:67N, RT:138K, RT:184I, RT:190E, RT:190S, RT:230I, IN:118R, IN:138K, IN:140R, IN:140S, IN:163K, IN:163R, IN:232N, IN:263K) to identify those that had the potential to have arisen from APOBEC activity^24^. Where an APOBEC-context drug resistance mutation was identified, the corresponding viral sequence was examined for evidence of APOBEC activity, indicated by the presence of at least one of the 154 reverse transcriptase or 95 integrase signature APOBEC mutations listed in the Stanford database^24^; or by the presence of at least one stop codon. APOBEC context drug resistance mutations were disregarded in the analysis if they occurred on a sequence with evidence of APOBEC activity; otherwise they were retained in the analysis. Other IAS-USA-listed mutations that are not APOBEC-context drug resistance mutations were included in the analysis, irrespective of whether there was evidence of APOBEC activity in the corresponding viral sequence. Drug resistance mutations were used to predict phenotypic susceptibility using the Stanford algorithm, version 9.8^24^

Changes in trunk fat mass and other body composition parameters were assessed by DEXA scans, performed in a subgroup of participants, enrolled consecutively at four of the eight sites participating in the main trial (two in Uganda, one each in Kenya and South Africa). There were no eligibility criteria for this substudy in addition to those required for the main trial. Participation was optional and participants signed a specific consent form, separate to that of the main trial. The baseline DEXA scan was required to be performed within 30 days of the baseline trial visit, with follow-up scans at weeks 48 and 96. In women who became pregnant during the trial, scans were deferred until the end of pregnancy plus 180 days or omitted if that date fell after week 96. Delays in follow-up DEXA scans were also permitted for logistical or technical reasons. Follow-up scans were performed on the same DEXA machine in each country, and all three machines were from the same manufacturer (Hologic). Scans were analyzed at each scan facility using standard manufacturer’s software.

Quality of life was assessed with the MOS-HIV questionnaire and treatment satisfaction with HIV Treatment Satisfaction Questionnaire (HIVTSQ), change version; both evaluated at weeks 48 and 96 (refs. ^25,26^). At the week 96 visit, participants in the long-acting therapy group were asked whether they preferred daily oral treatment or long-acting therapy.

### Outcomes

Baseline characteristics, including viral subtypes and resistance are reported in Table 1, Extended Data Tables 1–4 and Supplementary Table 1. Treatment regimens and adherence are reported in the Results and in Extended Data Fig. 2 and Supplementary Table 2.

The primary outcome for the trial was a viral load <50 copies ml^−1^ at week 48, determined using a modified Food and Drug Administration (FDA) snapshot algorithm. The modification specified that temporary regimen changes lasting no more than 31 days, use of oral bridging cabotegravir and rilpivirine with a subsequent return to injectable therapy, and within-class changes in the oral therapy group should not be considered treatment switches^27^. The primary outcome at week 48 has been previously reported^8^. The main outcome for the present analysis was viral load <50 copies ml^−1^ at week 96, analyzed in the same way (reported in Table 2). This outcome of viral suppression was chosen as the primary outcome, in preference to the outcome of viral nonsuppression (viral load ≥50 copies ml^−1^) recommended by the FDA for switch studies, because it aligns more closely with programmatic goals for HIV treatment and with the trial objective to provide relevant data on effectiveness of long-acting therapy under conditions of the public health approach in Africa.

The prespecified key secondary outcome was confirmed virological failure (two consecutive values ≥200 copies ml^−1^) by week 96 (reported in Table 2). Additional prespecified secondary outcomes were viral nonsuppression (≥50 copies ml^−1^), viral suppression <200 copies ml^−1^ and confirmed virological failure with new genotypic drug resistance mutation by week 96 (reported in Tables 2 and 3).

Safety outcomes comprise incident (from baseline to week 96) grade 3 or higher adverse events (reported in Table 4 and Supplementary Table 3), serious adverse events (reported in Table 4 and Supplementary Tables 4 and 5); HIV disease progression events (defined as death from any cause, serious acquired immunodeficiency syndrome (AIDS)-defining events based on Centers for Disease Control and Prevention criteria for AIDS^28^, and serious non-AIDS events based on criteria from the INSIGHT network^29^, reported in Table 4); events leading to discontinuation of treatment between baseline and week 96 (reported in Table 4); and injection-site reactions (reported in Table 4 and Extended Data Table 5).

Other prespecified (exploratory) outcomes are change from baseline to week 96 in CD4^+^ cell count (reported in Table 2); change in weight (post-hoc) and BMI (reported in Extended Data Tables 6 and 7 and Supplementary Fig. 1); change in body composition by DEXA (reported in Extended Data Table 8 and Extended Data Fig. 2); change in blood pressure and incident hypertension (both post-hoc; reported in Supplementary Table 6); change in selected laboratory metabolic and safety parameters (reported in Supplementary Tables 7 and 8); change in quality of life (reported in Supplementary Tables 9 and 10); treatment satisfaction at baseline and week 96 (reported in Supplementary Table 11); and participant treatment preference (reported in the text of the results). Outcomes of the hepatitis B baseline serology and social science substudies will be reported separately.

Further details regarding the conduct of the trial are provided in the protocol (Supplementary Information).

### Statistical analysis

The statistical analysis and sample size estimation described in this Article are, by necessity, identical to those reported in the earlier report for the first 48 weeks of the trial^8^. They are repeated in summary here to enable comprehension of the trial and to comply with standard clinical trial reporting requirements. The sample size justification for the analysis of DEXA scans was not presented in the earlier paper.

The main outcome was analyzed in the intention-to-treat exposed population (all those who received a dose of assigned intervention). The between-group difference was estimated using the Cochran–Mantel–Haenszel-weighted Miettinen and Nurminen method, adjusting for third-drug class (INSTI or NNRTI) at screening. Noninferiority of long-acting therapy could be concluded if the lower limit of the two-sided 95% CI for the difference between groups in the proportion of participants with viral load <50 copies ml^−1^ at week 96 was above −10 percentage points. This margin is consistent with the range (10–12%) used for judging noninferiority for the outcome of virological suppression (whether as primary or secondary outcome) in treatment trials done in the African program setting, and in previous long-acting trials^4,6,7,30–32^

Sensitivity analyses were performed using a model adjusting for site, sex and third-drug class; using a per-protocol population (excluding those who withdrew; had an injection more than 14 days from the scheduled date; or missed oral treatment for more than 7 days at one or more visits); and with the use of complete cases. The key secondary outcome of confirmed virological failure was also analyzed for noninferiority in the intention-to-treat exposed population, following a hierarchical approach conditional on demonstration of noninferiority on the primary outcome. For the analysis of this secondary outcome, a margin of 4% was prespecified for determining noninferiority.

Other outcomes were analyzed following the intention-to-treat approach but using complete case analysis. Analyses were exploratory, with descriptive presentation of data; groups were compared using *t*-tests or *χ*^2^ on only a limited number of prespecified parameters.

We estimated the sample size based on consideration of both the primary and the key secondary outcome at week 48. For the primary outcome, we assumed that 94% of participants in each group would have viral load <50 copies ml^−1^ at week 48 (based on previous 8-weekly long-acting trials);^5^ with 10% noninferiority margin, 5% two-sided significance level and 90% power we calculated that 238 participants (119 per group) were required to show noninferiority. For the key secondary outcome, we assumed that 1.7% would have confirmed virological failure in both arms (based on previous 8-weekly long-acting trials)^33^; with a hierarchical testing procedure, 4% noninferiority margin, 5% two-sided significance level and 85% power, we calculated that 512 participants (256 per group) were required to show noninferiority. We selected the larger of these two sample sizes to give adequate power for both analyses and to provide a substantive body of safety data that might give more confidence for adoption of long-acting therapy in the public health approach. The power to show noninferiority for the primary outcome with the stated parameters was close to 100%.

For the DEXA substudy, the target sample size was set at a minimum of 120 total participants, enrolled across the four participating sites. This was estimated to give at least 90% power to be able to detect a difference of 1.2 kg between treatment arms in the change in trunk fat mass from baseline (standard deviation of 2 kg; alpha 5%); based on body composition data in treatment naïve participants starting HIV treatment in Africa^2^. Given the limited data on which to base the sample size estimation, the desire to explore other body composition outcomes and the uncertain rate of attrition at later follow-up, sites were allowed to enroll beyond minimum sample size.

Secondary and other analyses are described in the statistical analysis plan (Supplementary Information). All analyses were conducted using Stata software version 16.1 (StataCorp), except for the estimation of differences in proportions for the efficacy analysis, which was performed using R version 4.3.3.

### Reporting summary

Further information on research design is available in the Nature Portfolio Reporting Summary linked to this article.

## Online content

Any methods, additional references, Nature Portfolio reporting summaries, source data, extended data, supplementary information, acknowledgements, peer review information; details of author contributions and competing interests; and statements of data and code availability are available at 10.1038/s41591-025-04041-7.

## Supplementary information

Supplementary Information Supplementary Tables 1–11, Fig. 1, Protocol and Statistical analysis plan.

Reporting Summary

## Figures and Tables

**Extended Data Table 1:**
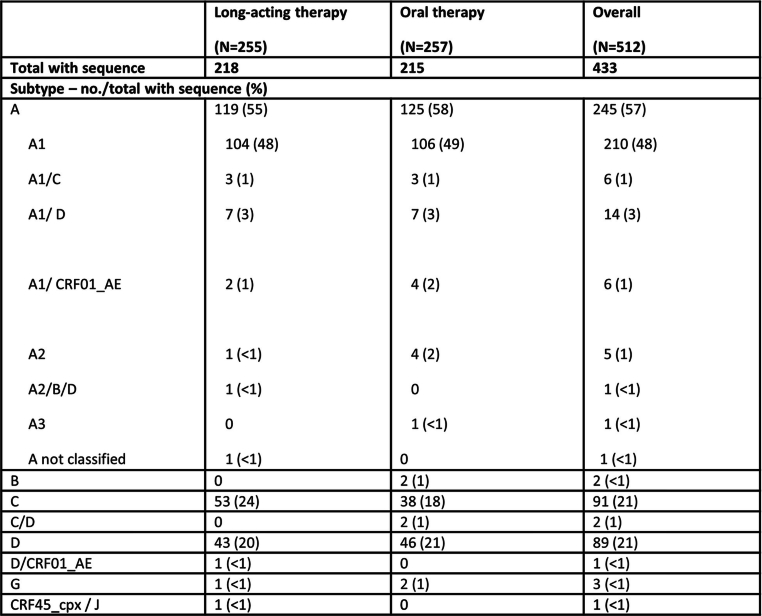
Subtype profile at baseline

Baseline subtype was determined from sequencing of DNA extracted from peripheral blood mononuclear cells. Reverse transcriptase and integrase sequences were obtained from 401 and 202 participants respectively. Subtype was identified from reverse transcriptase sequence in 401; and from integrase sequence in 32 participants where a reverse transcriptase sequence was not available.

Subtype was determined by reference to the Los Alamos National Laboratory HIV Sequence Database. Subtype based only on available integrase sequencing found an overall proportion of subtype A of 57%; and of subtype A1 of 55%. No subtype A6 virus was identified by reverse transcriptase or integrase sequencing.

Data previously published in ref. ^8^; updated with additional viral subtype from 19 participants and reclassification of subtype in 6 participants.

**Extended Data Table 2:**
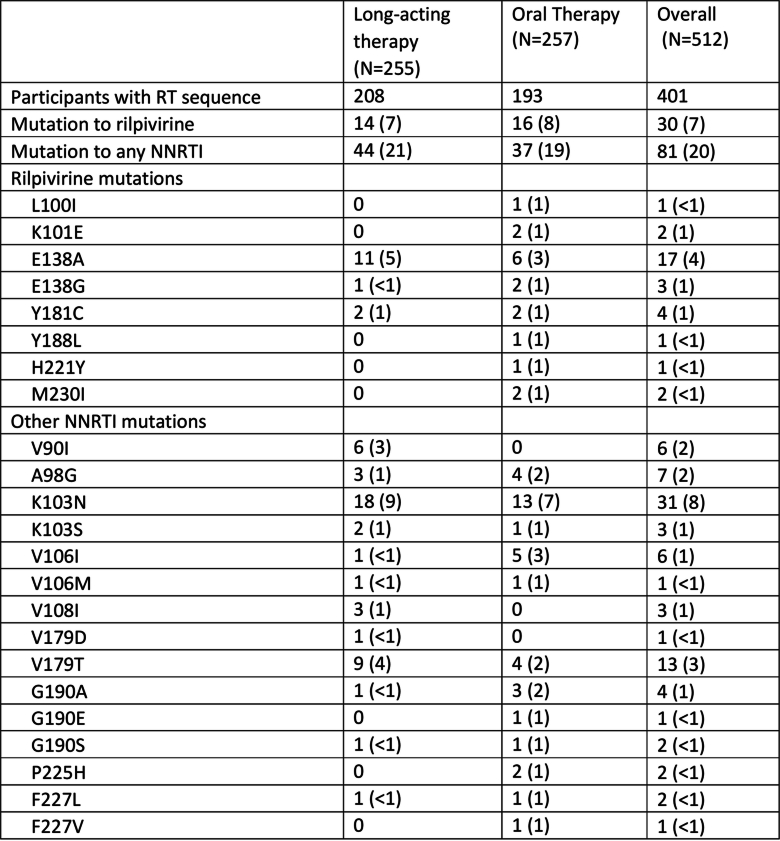
Resistance profile at baseline: non- nucleoside reverse transcriptase inhibitors

Data are n (%). Percentage is the proportion of sequences that have the specified mutation. NNRTI resistance mutations are those listed in the 2022 edition of the IAS–USA drug resistance mutations list (L100I, K101EP, E138AGKQR, V179L, Y181CIV,

Y188L, H221Y, F227C, M230IL for rilpivirine). NNRTI drug- resistance mutations were disregarded if they were APOBEC- context drug resistance mutations occurring on a sequence considered to be affected by APOBEC editing, indicated by the presence of at least one signature APOBEC mutation (as listed in the Stanford database). There were 33 sequences in the long-acting therapy group and 30 sequences in the oral therapy group that were considered to be affected by APOBEC editing. The rilpivirine resistance mutations disregarded for this reason are M230I in 12 participants and E138K in 2 participants (one with M230I) in the long-acting therapy group; and M230I in 12 participants in the oral therapy group. Additional NNRTI resistance mutations disregarded for this reason are G190E in 2 participants in the long-acting therapy group. Table adapted with permission from ref. ^8^, Elsevier.

**Extended Data Table 3:**
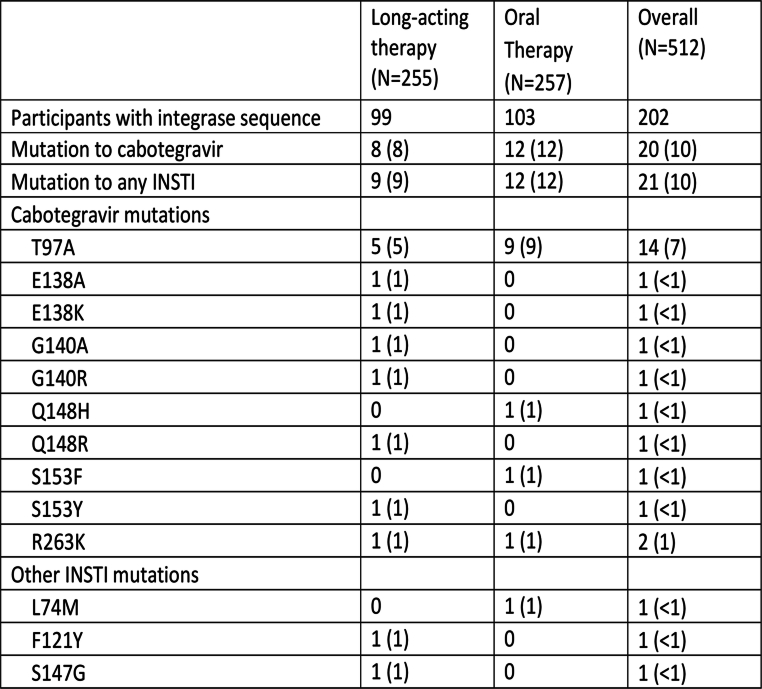
Resistance profile at baseline: integrase strand transfer inhibitors

Data are n (%). Percentage is the proportion of sequences that have the specified mutation. INSTI resistance mutations are those listed in the 2022 edition of the IAS–USA drug resistance mutations list. INSTI drug-resistance mutations were disregarded if they were APOBEC-context drug resistance mutations occurring on a sequence considered to be affected by APOBEC editing, indicated by the presence of at least one signature APOBEC mutation (as listed in the Stanford

database). There were 24 sequences in the long-acting therapy group and 32 sequences in the oral therapy group

that were considered to be affected by APOBEC editing. The INSTI resistance mutations disregarded for this reason are E138K in one participant, G140R in 5 participants and R263K in one participant in the long-acting therapy group; and G118R in one participant, E138K in one participant, G140R in three

participants in the oral therapy group. Table adapted with permission from ref. ^8^, Elsevier.

**Extended Data Table 4:**
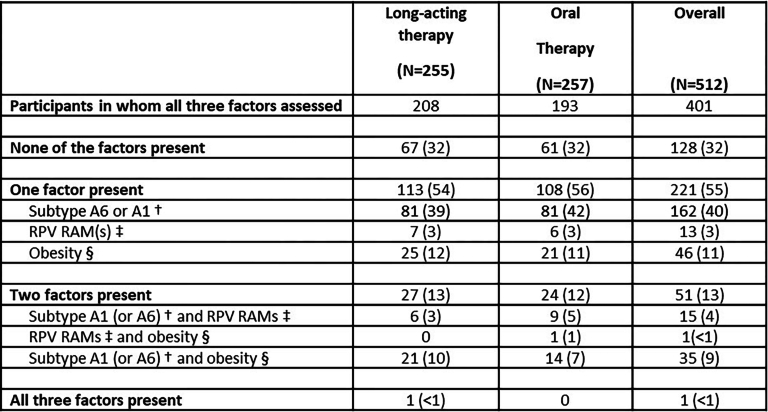
Profile of potential risk factors (from other studies) for virological failure at baseline

* Table shows number of participants (and proportion of those assessed) with each potential risk factor or combination of risk factors for virological failure within the 401 participants in whom all 3 risk factors were assessed. The three risk factors are those identified in multivariable analyses of pooled data from previous trials of long-acting therapy (Orkin et al, Clin Infect Dis 2023; 77 (10) 1423-1431).

RPV RAM(s) denotes rilpivirine resistance associated mutation(s)

† In the previously-published model, 90% of the participants categorised as having the higher risk A6 or A1 subtype had subtype A6 virus, and 10% had subtype A1 virus. In the population of the current trial, all participants under the category of subtype A6 or A1 virus had subtype A1; there was no subtype A6 virus identified.

‡ Rilpivirine resistance mutations are those listed in the 2022 edition of the IAS–USA drug resistance mutations list (L100I, K101EP, E138AGKQR, V179L, Y181CIV, Y188L, H221Y, F227C,

M230IL). RPV drug-resistance mutations listed as APOBEC context DRMs in the Stanford database and that occurred on a sequence considered to be affected by APOBEC editing were disregarded (not counted as a risk factor; E138K in 2 participants, M230I in 24 participants overall).

§ Obesity is defined as body mass index (BMI) ≥ 30 kg/m^2^. BMI is the weight in kilograms divided by the square of the height in meters. Table adapted with permission from ref. ^8^, Elsevier.

**Extended Data Table 5:**
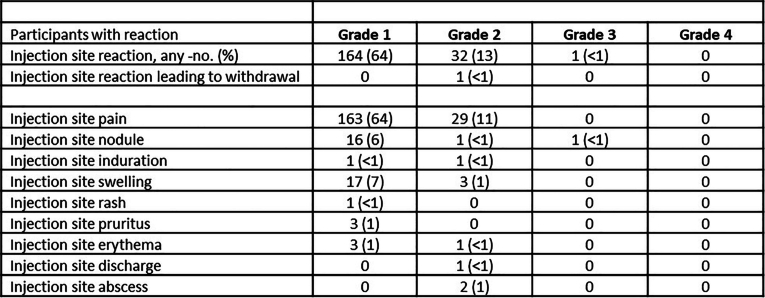
Injection site reactions in the long-acting therapy group from baseline to week 96

*Number of participants with injection site reactions, by maximum grade. Shown is the proportion of participants with reaction of that grade out of the total 255 participants in the long-acting group.

**Extended Data Table 6:**
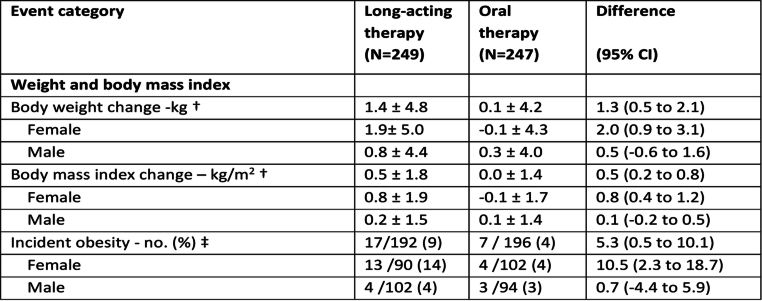
Weight and body mass index change from baseline to week 96

* Analysis performed on complete cases; plus-minus values are means ± SD. Analysis of body weight change is post-hoc

† Body weight change and body mass index (BMI) change from baseline to week 96 are estimated in the assessable population that excludes 7 female participants (2 long-acting therapy group, 5 oral therapy group) who were pregnant at any time during the 180 days prior to the week 96 visit; and 9 participants (4 long-acting therapy group, 5 oral therapy group) who withdrew from the trial or died before week 96.

P values for body mass index change are: women 0.0003, men 0.50, overall P = 0.0007.

‡ Incident obesity is defined as BMI ≥ 30 kg/m^2^ at week 96 in the assessable population that excludes 108 participants (57 long acting-therapy group, 51 oral therapy group) who had BMI ≥ 30 kgs/m^2^ at baseline; 7 female participants who were

pregnant at any time during the 180 days prior to the week 96

visit and 9 participants who withdrew from the trial or died before week 96 (assignment to treatment groups as above).

**Extended Data Table 7:**
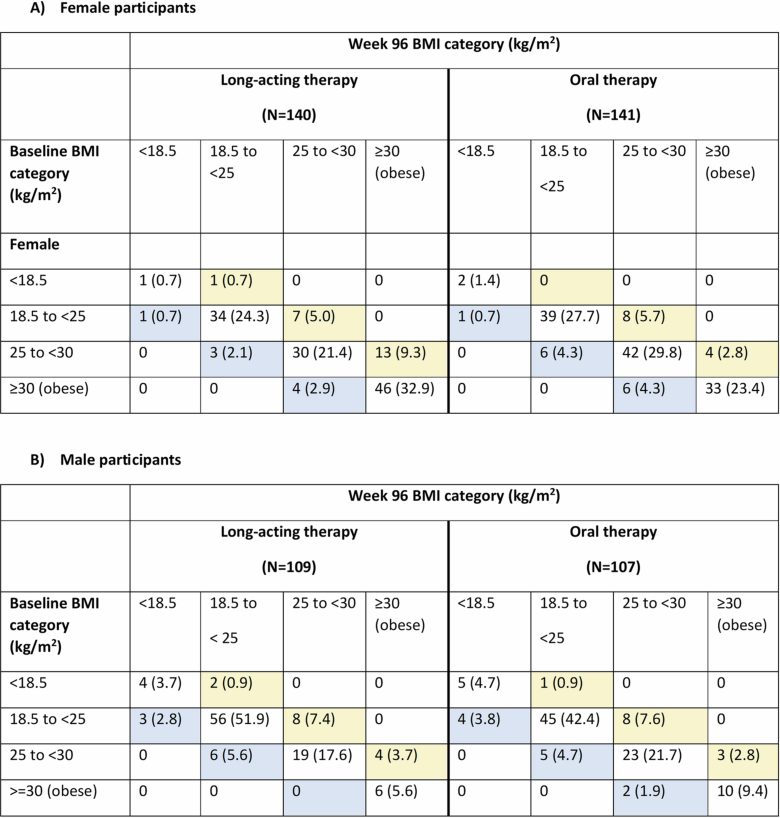
Shift change in body mass index from baseline to week 96

* Tables show the number of participants by BMI category at week 96, by treatment group and by sex; and the proportion of total participants within that treatment group and sex. The assessable population excludes 7 female participants (2 in

long-acting therapy, 5 in oral therapy arm) who were pregnant at any time during the 180 days prior to the week 96 visit; and

9 participants (4 long-acting therapy group, 5 oral therapy group) who withdrew from the trial or died before week 96. Cells highlighted in yellow indicate shift to higher BMI category; cells highlighted in blue indicate shift to lower BMI category.

**Extended Data Table 8:**
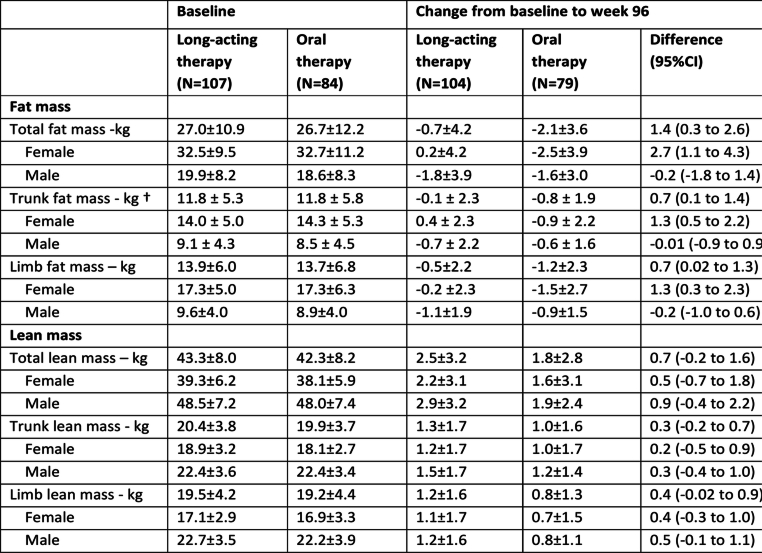
Body composition by DEXA at baseline and change to week 96

* Analysis of change was done in complete cases. Plus-minus values are means ± SD. DEXA scans were performed on the same machine at baseline and week 96. One DEXA machine was used in each country, all manufactured by Hologic Inc.

Scans were analysed at each site using standard

manufacturer’s software.

DEXA scans were not repeated at week 96 in 3 participants in the long-acting group (1 had died and 2 withdrew from follow- up before week 96) and 5 participants in the oral therapy group (2 withdrew from follow-up before week 96; 1 declined to have the scan repeated; 2 were unable to schedule repeat scans).

† For trunk fat mass change to week 96, pre-specified as the main DEXA outcome, we used an analysis of covariance (ANCOVA) model including baseline trunk fat mass, treatment group, sex, age ( < 50 vs ≥50 years), baseline ART third-agent class (NNRTI vs INI), and study site. In this model, the adjusted difference in trunk fat mass change (change in long-acting minus change in oral therapy group) was 1.01 (0.18 to 1.84) kg in women (P = 0.018), 0.21 (-0.72 to 1.14) kg in men (P = 0.657)

and 0.654 (0.03 to 1.27) kg overall (P = 0.038).

## Data Availability

Anonymized individual participant data that underlie the results reported in this Article (including data dictionaries) and study documents can be requested for a period of up to 24 months after publication of this Article. An independent review process of data access requests is required under the trial governance structure which formed the basis for local ethics and regulatory approvals. Requests should be sent to C.K. (ckityo@jcrc.org.ug) accompanied by an outline proposal for the intended analysis and a list of variables required. Requests will be reviewed by and subject to approval of the independent Trial Steering Committee. A response will be provided to the requesting party within a maximum of 2 months of receipt of the request. DNA and RNA sequences will be made available in GenBank upon publication of the Article, under accession numbers [X -Y] and are available at www.ncbi.nlm.nih.gov.
