# Supplementary material for: Cabotegravir and rilpivirine for treatment of HIV infection in Africa: week 96 results from the phase 3b randomized, open-label, noninferiority CARES trial
Source: Nat Med. 2025 Nov 4;32(1):168–77. doi: 10.1038/s41591-025-04041-7 (PMC12823427; doi:10.1038/s41591-025-04041-7)
Supplement: Supplementary file 1 — Supplementary Tables 1–11, Fig. 1, Protocol and Statistical analysis plan. [file 41591_2025_4041_MOESM1_ESM.pdf]

nature medicine

Supplementary information https://doi.org/10.1038/s41591-025-04041-7

Cabotegravir and rilpivirine for treatment of HIV infection in Africa: week 96 results from the phase 3b randomized, open-label, noninferiority CARES trial

In the format provided by the authors and unedited

TABLE OF CONTENTS

Members of the CARES trial team ……………………………………………………………………………....................3-4

Supplementary tables

Table S1 Resistance profile at baseline: nucleoside reverse transcriptase inhibitors ……………….….......5

Table S2 Treatment regimens at screening and following randomisation ……………………………….…......6

Table S3 Adverse events of at least grade 3 severity between baseline and week 96…..………………..7-8

Table S4 Serious adverse events between baseline and week 96 ………………………………………………….9

Table S5 Serious adverse events between baseline and week 96, by seriousness category……………..10

Table S6 Blood pressure changes from baseline to week 96 and incident hypertension…………………11

Table S7 Glucose and lipid metabolism changes from baseline to week 96 ……………………………… 12-13

Table S8 Changes in routine safety parameters from baseline to week 96 ……………………………….…..14

Table S9 MOS-HIV quality of life subscales at baseline and week 96 ………………………………………………15

Table S10 Change in MOS-HIV summary scores from baseline to week 96 …………………………………....16

Table S11 Treatment satisfaction score at baseline and change at week 96……………………………………17

Supplementary figure

Figure S1 Transition of participants between BMI categories from baseline to week 96 ………………18

MEMBERS OF THE CARES TRIAL TEAM

Participating sites and laboratories

Uganda

Joint Clinical Research Centre (JCRC), Lubowa (108): Henry Mugerwa, William Tamale, Joshua Yiga, Susan Esther Asaasira, Nigel Kinyera, Christine Nambi, Dridah L. Nakiboneka, Rose Kabatana, Winfred Kiyimba

JCRC Fort Portal, Fort Portal (69): Gilbert Ategeka, Ibrahim Yawe, Adolf Alinaitwe, Aidah Zawedde, George Wasswa, Allan Arinda, Angela Rweyora, Mary Goretti Kangah, Oscar Ruhweza, Felly Kaguma, Beatrice Venny Kabaswahil.

Infectious Diseases Institute, Kampala (67): Fiona Cresswell, Barbara Castelnuovo, Arvind Kaimal, Patience Ogwal, Neville Muhumuza, Max Okwero, Peruth Ayebare, Vivian Nakate, Jesca Asienzo, Hamza Mayanja, Eva Laker.

Kenya

Aga Khan University Hospital, Nairobi (40): Reena Shah, Felix Riunga, , Peter Odhiambo Onyango, Josephine Wanja, Shaheen Sayed, Jaimini Gohil, Isaiah Mungathia, Alfred Mburu Githuka, Haron Kibwage.

Moi University Clinical Research Center, Eldoret (62): Abraham Mosigisi Siika, Charity Wambui Kanyoro, Viola Cherotich Kirui, Jairus Kipyego, Natalie Sang, Martha Mokeira Bisieri Mokaya, Consolata Chepkorir, Joan Kotut, Millicent Orido, Florence Njulu, Hilda Kaziga

KEMRI-MRDA, Kericho (60): Josphat Kosgei, Fredrick Sawe, Magdaline Adhiambo Omol, Faith Riziki, Daud Ibrahim, Leelgo Kimetto, Billy Omalla Okumu, Francis Lipuku

South Africa

Ezintsha, Johannesburg (97): Simiso Sokhela, Francois Venter, Karlien Moller, Nompumelelo Nzuza, Gontse Ramela, Noxolo Tom, Tsitsi Nyamuzihwa, Philadelphia Macholo, Hlamulani Macebele, Godspower Akpomiemie.

Chatsworth Clinical Research Site, South African Medical Research Council (SAMRC), Durban (9): Logashvari Naidoo, Nitesha Jeenarain, Nivriti Hurbans, Mayuri Reddy, Gerald Thsepo Mphisa

Trial coordination

Joint Clinical Research Centre (JCRC), Lubowa, Uganda
Cissy Kityo, Nicholas Paton, Ivan Kiggundu, Mambule, Kimton Opiyo, Joseph Musaazi, Caroline Otike, Ritah Kabanyoro, Francis Sekajja, Sandra Nantubwe, Harriet Sekabira, Paul Ocitti, Benson Ouma, Immaculate Nankya, Pamela Ainembabazi

IQVIA, South Africa
Melissa Lötter, Saeeda Mohamed, Madel Herbst, Tarryn Peters, Heena Mehta, Shaguftha Khan, Marlien Kruger

Janssen Pharmaceutica, Belgium
Willemijn van Rein-van der Horst, Fafa Addo Boateng, Rodica Van Solingen, Kati Vandermeulen, Veerle Van Eygen, Herta Crauwels, Donghan Luo, Donna Votto, Idahosa Awhonukeh, Fridah Mwendia, Lynne Klasko-Foster, Malavika Bondai, Ingrid Eshun-Wilsonova, Perry Mohammed

ViiV Heathcare Ltd., UK
William Spreen, Ronald D'Amico

Trial Governance

Trial Steering Committee (TSC): Independent members: Pontiano Kaleebu (Chair), Sylvia Ojoo, Milly Katana, Yunus Moosa; Trial-related members: Cissy Kityo, Nicholas Paton, Henry Mugerwa, Fredrick Sawe, Simiso Sokhela. Non-voting members: Fafa Addo Boateng

Independent Data Monitoring Committee (IDMC)
Sam Phiri (chair), Paula Munderi, Andrew Hill

Table S1 Resistance profile at baseline: nucleoside reverse transcriptase inhibitors

	Long-acting therapy (N=255)	Oral Therapy (N=257)	Overall (N=512)
Participants with RT sequence	208	193	401
Mutation to any NRTI	16 (8)	16 (8)	32(8)
NRTI mutations			
M41L	3 (1)	3 (2)	6 (1)
A62V	2 (1)	1 (1)	3 (1)
K65R	0	1 (1)	1 (<1)
D67N	1 (<1)	1 (1)	2 (<1)
K70E	2 (1)	1 (1)	3 (1)
K70R	1 (<1)	1 (1)	2 (<1)
L74V	1 (<1)	0	1 (<1)
V75I	1 (<1)	2 (1)	3 (1)
M184I	0	0	0
M184V	11 (5)	10 (5)	21 (5)
L210W	1 (<1)	3 (2)	4 (1)
T215F	1 (<1)	1 (1)	2 (<1)
T215Y	0	1 (1)	1 (<1)
K219Q	0	1 (1)	1 (<1)

Data are n (%). Percentage is the proportion of sequences that have the specified mutation. NRTI resistance mutations are those listed in the 2022 edition of the IAS–USA drug resistance mutations list. NRTI drug-resistance mutations were disregarded if they were APOBEC-context drug resistance mutations occurring on a sequence considered to be affected by APOBEC editing, indicated by the presence of at least one signature APOBEC mutation (as listed in the Stanford database). There were 33 sequences in the long-acting therapy group and 30 sequences in the oral therapy group that were considered to be affected by APOBEC editing. The NRTI resistance mutations disregarded for this reason are M184I in 10 participants in the long-acting therapy group; and D67N in 1 participant and M184I in 13 participants (combined with the D67N mutation in one participant) in the oral therapy group.

Data previously published in Kityo, Mambule, Musaazi et al; Lancet Infect Dis 2024; 24: 1083-1092; updated with additional sequences from 19 participants and removal of APOBEC-context drug resistance mutations.

Table S2 Treatment regimens at screening and following randomisation

	Long-acting therapy (N=255)	Oral therapy (N=257)	Overall (N=512)
**Treatment regimen at screening no. (%)**			
**DTG with NRTIs**			
TDF and 3TC	230 (90)	240 (93)	470 (92)
TDF and FTC	1 (<1)	0	1 (<1)
**EFV with NRTIs**			
TDF and 3TC	14 (5)	10 (4)	24 (5)
TDF and FTC	10 (4)	7 (3)	17 (3)
**Initial regimen following randomization no. (%)**			
**CAB + RPV**			
4 weeks CAB+RPV oral, then injection	214 (84)	N/A	N/A
4 weeks standard oral, then injection	41 (16)	N/A	N/A
**DTG with NRTIs**			
TDF and 3TC	N/A	239 (93)	N/A
TDF and FTC	N/A	0	N/A
**EFV with NRTIs**			
TDF and 3TC	N/A	10 (4)	N/A
TDF and FTC	N/A	8 (3)	N/A

Data previously published in Kityo, Mambule, Musaazi et al; Lancet Infect Dis 2024; 24: 1083-1092.

Table S3: Adverse events of at least grade 3 severity between baseline and week 96, by system organ class and preferred term*

	**Long-acting therapy** (N=255)	**Oral therapy** (N=257)	**Overall** (N=512)
**Participants with adverse event grade ≥ 3 - no (%)**	41 (16)	22 (9)	63 (12)
**Infections and infestations**	**2 (1)**	**0**	**2 (<1)**
Malaria	1 (<1)	0	1 (<1)
Meningitis viral	1 (<1)	0	1 (<1)
**Blood and lymphatic system disorders**	**4 (2)**	**4 (2)**	**8 (2)**
Anaemia	4 (2)	1 (<1)	5 (1)
Immune thrombocytopenia	0	1 (<1)	1 (<1)
Thrombocytopenia	0	2 (1)	2 (<1)
**Vascular disorders**	**1** (<1)	**2 (1)**	**3 (1)**
Hypertension	1 (<1)	2 (1)	3 (1)
**General disorders and administration site conditions**	1 (<1)	0	1 (<1)
Injection site nodule	1 (<1)	0	1 (<1)
**Injury, poisoning and procedural complications**	**5 (2)**	**1** (<1)	**6 (1)**
Ankle fracture	2 (1)	0	2 (<1)
Human bite	1 (<1)	0	1 (<1)
Lower limb fracture	1 (<1)	0	1 (<1)
Radius fracture	0	1 (<1)	1 (<1)
Road traffic accident	1 (<1)	0	1 (<1)
**Psychiatric disorders**	**1 (<1)**	**1 (<1)**	**2 (<1)**
Major depression	0	1 (<1)	1 (<1)
Substance-induced psychotic disorder	1 (<1)	0	1 (<1)
**Investigations**	**16 (6)**	**9 (4)**	**25 (5)**
Aspartate aminotransferase increased	0	1 (<1)	1 (<1)
Blood bilirubin increased	1 (<1)	0	1 (<1)
Blood cholesterol increased	4 (2)	0	4 (1)
Blood glucose increased	0	1 (<1)	1 (<1)
Blood sodium increased	0	2 (1)	2 (<1)
Blood sodium decreased	3 (1)	1 (<1)	4 (1)
Blood triglycerides increased	3 (1)	0	3 (1)
Estimated glomerular filtration rate decreased	0	3 (1)	2 (<1)
Haemoglobin decreased	1 (<1)	0	1 (<1)
Low density lipoprotein increased	8 (3)	2 (1)	10 (2)
Neutrophil count decreased	0	1 (<1)	1 (<1)
**Renal and urinary disorders**	**3 (1)**	**1 (<1)**	**4 (1)**
Glycosuria	2 (1)	1 (<1)	3 (1)
Proteinuria	1 (<1)	0	1 (<1)

**Metabolism and nutrition disorders**	**9 (4)**	**5 (2)**	**14 (3)**
Abnormal loss of weight	2 (1)	1 (<1)	3 (1)
Diabetes mellitus	3 (1)	1 (<1)	4 (1)
**Pregnancy, puerperium and perinatal conditions**	**1 (<1)**	**1 (<1)**	**2 (<1)**
Abortion spontaneous	1 (<1)	0	1 (<1)
Oligohydramnios	0	1 (<1)	1 (<1)
**Gastrointestinal disorders**	**3 (1)**	**0**	**3 (1)**
Abdominal incarcerated hernia	1 (<1)	0	1 (<1)
Strangulated umbilical hernia	1 (<1)	0	1 (<1)
Vomiting	1 (<1)	0	1 (<1)
**Neoplasms benign, malignant and unspecified (incl cysts and polyps)**	**1 (<1)**	**1 (<1)**	**2 (<1)**
Lung adenocarcinoma	1 (<1)	0	1 (<1)
Prostate adenocarcinoma	0	1 (<1)	1 (<1)
**Nervous system disorders**	**0**	**2 (1)**	**2 (<1)**
Encephalopathy	0	1 (<1)	1 (<1)
Generalised tonic-clonic seizure	0	1 (<1)	1 (<1)

*Adverse event grading based on Division of AIDS Toxicity Criteria. QTc prolongation required QTcF ≥ 500ms. Coded according to Medical Dictionary for Regulatory Activities (MedDRA). Numbers shown are number of participants with at least one adverse event in each category.

Table S4: Serious adverse events between baseline and week 96, by system organ class and preferred term

System Organ Class<br>Preferred Term	Long-acting therapy (n=255)	Oral therapy (n=257)	Overall (n=512)
**Participants with at least one SAE – no (%)**	**12 (5)**	**9 (4)**	**21 (4)**
**Infections and infestations**	**2 (1)**	**0**	**2 (<1)**
Malaria	1	0	1
Meningitis viral	1	0	1
**Injury, poisoning and procedural complications**	**5 (2)**	**1 (<1)**	**6 (1)**
Ankle fracture	2	0	2
Human bite	1	0	1
Lower limb fracture	1	0	1
Road traffic accident	1	0	1
Soft tissue injury	0	1	1
**Neoplasms benign, malignant and unspecified (incl cysts and polyps)**	**1 (<1)**	**2 (1)**	**3 (1)**
Lung adenocarcinoma	1	0	1
Parapharyngeal squamous cell carcinoma	0	1	1
Prostate adenocarcinoma	0	1	1
**Pregnancy, puerperium and perinatal conditions**	**1 (<1)**	**3 (1)**	**4 (1)**
Abortion spontaneous	1	2	3
Oligohydramnios	0	1	1
**Psychiatric disorders**	**1 (<1)**	**1 (<1)**	**2 (<1)**
Major depression	0	1	1
Substance-induced psychotic disorder	1	0	1
**Gastrointestinal disorders**	**3 (1)**	**1 (<1)**	**4 (1)**
Abdominal incarcerated hernia	1	1	2
Strangulated umbilical hernia	1	0	1
Vomiting	1	0	1
**Nervous system disorders**	**0**	**2 (1)**	**2 (<1)**
Encephalopathy	0	1	1
Generalised tonic-clonic seizure	0	1	1
**Reproductive system and breast disorders**	0	1	1
Benign prostatic hyperplasia	0	1	1

Numbers shown are number of participants with at least one adverse event in each category.

Table S5: Serious adverse events between baseline and week 96, by seriousness category

	Long-acting therapy (n=255)	Oral therapy (n=257)	Total (n=512)
Participants with at-least one SAE, n (%)	**12 (5)**	**9 (4)**	**21 (4)**
Total SAEs, N	***14***	***11***	***25***
Fatal *	1	1	2
Life-threatening	0	0	0
Hospitalisation	11	7	18
Persistent or major disability or incapacity	0	0	0
Congenital anomaly or birth defect	0	0	0
Other important medical condition	2	4	6

Numbers for total SAEs are number of events within each category of classification. One participant in the oral therapy group had an event that was initially classified as hospitalization that later progressed to death.

***** Death was due to post-operative complications of surgery for strangulated umbilical hernia for the participant in the long-acting group; and due to encephalopathy associated with parapharyngeal squamous cell carcinoma for the participant in the oral therapy group.

Table S6 Blood pressure changes from baseline to week 96 and incident hypertension *

Event category	Long-acting therapy (N=255)	Oral therapy (N=257)	Difference (95% CI)
Systolic bp change – mmHg †	-3.0 ±13.2	-3.2 ±12.6	0.3 (-2.0 to 2.5)
Female	-2.6±14.0	-4.6±11.8	2.0 (-1.1 to 5.0)
Male	-3.4 ±12.0	-1.5 ±13.5	-2.0 (-5.4 to 1.5)
Diastolic bp change – mmHg †	-2.1±9.9	-1.5±8.8	-0.7 (-2.3 to 1.0)
Female	-2.5±10.9	-1.3±8.7	-1.2 (-3.6 to 1.1)
Male	-1.6±8.5	-1.7±9.0	0.1 (-2.3 to 2.4)
Incident systolic bp > 140 mmHg – no. (%) ‡	22/207 (11)	21/208 (10)	0.5 (-5.3 to 6.4)
Female	12 /111 (11)	15 /118 (13)	-1.9 (-10.2 to 6.4)
Male	10 /96 (10)	6 /90 (7)	3.8 (-4.2 to 11.7)
Incident diastolic bp > 90 mmHg – no. (%) §	24/220 (11)	21/215 (10)	1.1 (-4.6 to 6.9)
Female	11 /117 (9)	14 /125 (11)	-1.8 (-9.4 to 5.9)
Male	13 /103 (13)	7 /90 (8)	4.8 (-3.6 to 13.3)
Started antihypertensive drug – no. (%) ¶	20 / 213 (9)	9 /214 (4)	5.2 (0.4 to 9.9)
Female	14 /119 (12)	4 /123 (3)	8.5 (1.9 to 15.1)
Male	6 /94 (6)	5 /91 (5)	0.9 (-5.9 to 7.7)
Incident hypertension – no (%) ‖	29/183 (16)	22 /182 (12)	3.8 (-3.3 to 10.9)
Female	17 /97 (18)	16 /104 (15)	2.1 (-8.1 to 12.4)
Male	12 /86 (14)	6 /78 (8)	6.3 (-3.2 to 15.7)

* Analysis performed on complete cases; plus-minus values are means ± SD. Analyses of blood pressure changes and incident hypertension are post-hoc.

† Systolic bp change and diastolic bp change from baseline to week 96 are estimated in the assessable population that excludes 11 female participants (2 long-acting therapy group, 9 oral therapy group) who became pregnant at any time during the trial and 9 participants (4 long-acting therapy group, 5 oral therapy group) who withdrew from the trial or died before week 96. Baseline bp is the average of measured screening and baseline bp values.

‡ Incident systolic bp > 140 mmHg is defined as at least one bp reading at any time following the baseline study day until week 96 in the assessable population that excludes 86 participants (46 long-acting therapy group, 40 oral therapy group) who had a systolic bp > 140mmHg at baseline, and 11 female participants (2 long-acting therapy group, 9 oral therapy group) who became pregnant at any time during the trial.

§ Incident diastolic blood pressure >90mmHg is defined as at least one bp measurement at any time following the baseline study day until week 96 in the assessable population that excludes 66 participants (33 long-acting therapy group, 33 oral therapy group) who had a diastolic bp > 90mmHg at baseline, and 11 female participants (2 long-acting therapy group, 9 oral therapy group) who became pregnant at any time during the trial.

¶ The number of participants that started a new antihypertensive drug between baseline and week 96 is estimated in the assessable population that excludes 74 participants (40 long-acting therapy group, 34 oral therapy group) who were on any antihypertensive drug at baseline, and 11 female participants (2 long-acting therapy group, 9 oral therapy group) who became pregnant at any time during the trial.

‖ Incident hypertension is defined as one or more of incident systolic bp > 140mmHg, incident diastolic bp > 90mmHg or started a new antihypertensive drug in the assessable population that excludes 147 participants (72 long-acting therapy group, 75 oral therapy group) who had elevated bp at baseline, or who were on an antihypertensive drug at baseline or who became pregnant at any time during the trial.

Table S7: Glucose and lipid metabolism changes from baseline to week 96 *

	Long-acting therapy (n=255)	Standard oral therapy (n=257)	Difference (95% CI)
**Diabetes**			
Change in glucose (mmol/L) fasted †	-0.04 ± 0.71	0.01 ±1.27	-0.06 (-0.24, 0.13)
Female	-0.05 ± 0.72	-0.09 ± 1.10	0.04 (-0.18, 0.26)
Male	-0.03 ± 0.70	0.17 ± 1.48	-0.20 (-0.52, 0.12)
Incident glucose > 7mmol/L, fasted– no. (%) ‡	6 /248 (2)	18/248 (7)	-4.8 (-8.6, -1.1)
Female	2/140 (1)	7/143 (5)	-3.5 (-7.5, 0.6)
Male	4/108 (4)	11/105 (10)	-6.8 (-13.6, 0.1)
Started anti-diabetes medication– no. (%) §	4/251 (2)	3/251 (1)	0.4 (-1.7, 2.4)
Female	2/143 (1)	2/145 (1)	0.02 (-2.7, 2.7)
Male	2/108 (2)	1/106 (1)	0.9 (-2.2, 4.0)
Incident diabetes – no (%) ¶	7/247 (3)	17/246 (7)	-4.1 (-7.9, -0.3)
Female	3/140 (2)	6/142 (4)	-2.0 (-6.1, 2.0)
Male	4/107 (4)	11/104 (11)	-6.8 (-13.8, 0.1)
**Hyperlipidaemia**			
Change in total cholesterol (mg/dL) ‖	17.15± 32.86	2.67 ± 28.67	14.48 (9.05, 19.90)
Female	17.49 ± 34.48	1.15± 27.53	16.34 (9.09, 23.59)
Male	16.69 ± 30.72	4.76 ± 30.18	11.93 (3.69, 20.18)
Change in triglycerides (mg/dL) ‖	16.78± 57.58	4.17± 54.53	12.61 (2.74, 22.47)
Female	15.02± 45.89	1.06± 47.95	13.97 (3.08, 24.85)
Male	19.13± 70.41	8.52± 62.55	10.61 (-7.49, 28.70)
Change in HDL (mg/dL) **	5.92± 19.76	2.37± 10.45	3.55 (-1.13, 8.24)
Female	5.79 ± 20.55	2.52± 11.15	3.27 (-3.22, 9.75)
Male	6.18± 18.50	2.18± 9.65	4.00 (-2.82, 10.81)
Change in LDL **	14.31± 54.11	1.90± 34.90	12.42 (-1.04, 25.88)
Female	13.00± 62.64	-2.06± 36.51	15.05 (-5.01, 35.11)
Male	16.79± 33.44	6.86± 32.55	9.92 (-5.76, 25.60)
Incident total cholesterol ≥240 mg/d – *no.(%)*††	26/242 (11)	9/236 (4)	6.9 (2.3, 11.5)
Female – no. (%)	20/135 (15)	6/136 (4)	10.4 (3.5, 17.3)
Male – no. (%)	6 /107 (6)	3/100 (3)	2.6 (-2.9, 8.1)
Incident LDL ≥ 160mg/dL – no. (%) ‡‡	18/53 (34)	2/50 (4)	30.0 (16.1, 43.8)
Female– no. (%)	13/30 (43)	2/29 (7)	36.4 (16.4, 56.4)
Male – no. (%)	5/23 (22)	0/21 (0)	21.7 (4.9, 38.6)
Started cholesterol-reducing drug – no. (%)§§	15/251 (6)	7/255 (3)	3.2 (-0.3, 6.8)
Female – no. (%)	11/145 (8)	2/148 (1)	6.2 (1.5, 10.9)
Male – no. (%)	4/106 (4)	5/107 (5)	-0.9 (-6.3, 4.5)
Incident hyperlipidaemia – no. (%) ¶¶	30/209 (14)	8/211 (4)	10.6 (5.2, 16.0)
Female – no. (%)	20/112 (18)	4/124 (3)	14.6 (6.9, 22.4)
Male – no. (%)	10/97 (10)	4/87 (5)	5.7 (-1.8, 13.2)

* Analysis performed on complete cases; plus-minus values are mean ± SD.

† Paired values for measuring change from baseline to week 96 for fasting glucose were available in 482/512 (94%; 243 long acting-therapy group, 239 oral therapy group) .

‡ Incident fasted glucose > 7 mmol/L is defined as at least one glucose measurement at any time following the baseline study day until week 96, in the assessable population that excludes 16 participants (7 long-acting therapy group, 9 oral therapy group) who had fasted glucose > 7 mmol/L at baseline.

§ The number of participants starting a new antidiabetic drug between baseline and week 96 is estimated in the assessable population that excludes 10 participants (4 long-acting therapy group, 6 oral therapy group) who were on any anti-diabetes drug at baseline.

¶ Incident diabetes is defined as one or both of incident fasted glucose > 7mmol/L or starting a new anti-diabetic drug in the assessable population that excludes 19 participants (8 long-acting therapy group, 11 oral therapy group) who had either fasting glucose > 7 mmol/L or were on any anti-diabetes drugs at baseline.

|| Paired values for measuring change from baseline to week 96 for total cholesterol and triglycerides were available in 499/512 (97%; 250 long acting-therapy group, 249 oral therapy group).

** Paired values for measuring change from baseline to week 96 for HDL and LDL were available in 180/512 (35%; 92 long-acting therapy group, 88 oral therapy group).

†† Incident total cholesterol ≥240 mg/dL (6.19mmol/L) is defined as at least one total cholesterol measurement at week 48 and/or week 96 in the assessable population that excludes 13 participants (5 long-acting, 8 oral-therapy who did not have total cholesterol measurement at baseline paired with a measurement at week 48 or 96; and 21 participants (8 long-acting therapy group, 13 oral therapy group) who had total cholesterol ≥240 mg/dL at baseline.

‡‡ Incident LDL ≥160mg/dL (4.12mmol/L) is defined as at least one LDL measurement at week 48 and/or week 96 in the assessable population that excludes 332 participants (163 long-acting, 169 oral-therapy who did not have an LDL measurement at baseline paired with a measurement at week 48 or 96; and 77 participants (39 long-acting therapy group, 38 oral therapy group) who had LDL ≥160mg/dL at baseline.

§§ The number of participants starting a new cholesterol-reducing drug between baseline and week 96 is estimated in the assessable population that excludes 6 participants (4 long-acting therapy group, 2 oral therapy group) who were taking any cholesterol-lowering drug at baseline.

¶¶ Incident hyperlipidaemia is defined as having one or more of incident total cholesterol ≥240 mg/dL, LDL ≥160 mg/dL, and/or starting a new cholesterol-reducing drug (meeting definitions above) in the assessable population that excludes 92 participants (46 long-acting therapy group, 46 oral therapy group) who did not have either a cholesterol measurement at baseline paired with a measurement at week 48 or 96, or an LDL measurement at baseline paired with a measurement at week 48 or 96; and/or who had total cholesterol ≥240 mg/dL or LDL ≥160 mg/dL at baseline; and/ or who were taking any cholesterol-lowering drug at baseline.

Table S8: Changes in routine safety parameters from baseline to week 96*

	Long-acting therapy (n=255)	Standard oral therapy (n=257)	Difference (95% CI)
Change in Haemoglobin (g/dL) †	0.05 ± 1.39	-0.21 ± 1.31	0.27 (0.03, 0.50)
Female	0.29 ± 1.43	-0.24 ± 1.41	0.54 (0.21, 0.87)
Male	-0.26 ± 1.27	-0.17 ±1.17	-0.09 (-0.42, 0.24)
Change in eGFR (ml/min/1.73 m^2) † ‡	7.57 ± 11.85	-2.48 ± 11.81	10.05 (7.98, 12.12)
Female	6.87 ± 12.33	-2.68 ± 13.38	9.55 (6.57, 12.53)
Male	8.49 ± 11.18	-2.21 ± 9.28	10.70 (7.93, 13.47)
Change in ALT (IU/L) †	-2.98 ± 15.18	-1.02 ± 11.32	-1.96 (-4.30, 0.38)
Female	-1.97 ± 18.11	-1.02 ± 10.85	-0.95 (-4.40, 2.49)
Male	-4.32 ± 9.99	-1.02 ± 11.98	-3.29 (-6.26, -0.32)
Change in total bilirubin (mg/dL) †	0.02 ± 0.27	-0.005 ± 0.27	0.03 (-0.018, 0.078)
Female	0.04 ± 0.21	-0.01 ± 0.20	0.06 (0.01, 0.10)
Male	-0.001 ± 0.33	0.003 ± 0.36	-0.0048 (-0.10, 0.09)
Change in alkaline phosphatase (IU/L) †	-10.63± 21.01	2.56± 28.25	-13.19 (-17.55, -8.83)
Female	-11.84 ± 21.45	2.38 ± 30.23	-14.22 (-20.30, -8.14)
Male	-9.02 ± 20.40	2.81 ± 25.41	-11.83 (-18.04, -5.63)

* Plus-minus values are mean ± SD.

† Paired values for measuring change from baseline to week 96 were available for eGFR, ALT, alkaline phosphatase and bilirubin in 503/512 (98%; 251 long acting-therapy group, 252 oral therapy group); and for haemoglobin in 502/512 (98%; 250 long-acting, 252 oral-therapy)

‡ Estimated Glomerular Filtration Rate (eGFR) obtained by 2021 CKD-EPI equation.

Table S9 MOS-HIV quality of life subscales at baseline and week 96*

Sub-scales	Long-acting therapy (N= 251)		Oral therapy (N= 252)	
	**Baseline**	**Week 96**	**Baseline**	**Week 96**
General health perception	89.4 ± 14.0	87.7 ± 18.1	88.8 ± 14.8	88.6 ± 17.0
Physical functioning	95.6 ± 16.0	97.5 ± 11.6	97.4 ± 9.9	98.9 ± 5.0
Social functioning	97.1 ± 12.4	98.1 ± 11.2	99.1 ± 4.1	99.2 ± 8.9
Role functioning	98.8 ± 9.9	98.8 ± 9.9	98.0 ± 11.6	99.4 ± 7.0
Cognitive functioning	95.0 ± 13.9	97.4 ± 9.4	96.3 ± 11.8	98.5 ± 5.3
Pain	95.3 ± 12.1	95.5 ± 14.9	95.6 ± 11.4	97.3 ± 8.1
Vitality/Energy/Fatigue	88.5 ± 15.9	88.6 ± 15.8	88.7 ± 17.2	89.3 ± 13.4
Mental health/Emotional well-being	90.2 ± 13.3	89.1 ± 17.2	89.9 ± 14.8	89.9 ± 14.3
Health distress	94.7 ± 11.7	96.9 ± 12.2	95.6 ± 11.7	97.2 ± 8.9
Overall quality of life†	83.6 ± 15.0	81.9 ± 18.8	84.4 ± 16.3	81.3 ± 18.7
Health transition†	74.6 ± 24.2	78.2 ± 25.2	76.9 ± 23.3	79.8 ± 24.1
**Summary scores**				
Physical summary score (PHS)†	60.5 ± 4.2	60.8 ± 5.0	60.8 ± 3.7	61.4 ± 2.5
Mental summary score (MHS)†	61.4 ± 5.7	61.4 ± 6.5	61.6 ± 5.4	61.6 ± 5.0

* MOS-HIV denotes the Medical Outcomes Study-HIV questionnaire. Responses to individual questions were converted to scores on 11 subscales, ranging from 0 to 100, with higher scores indicating better health.

Plus-minus values are mean ± SD. Analysis is performed on complete cases. The assessable population excluded 9 participants who withdrew or died before week 96 (4 in long-acting group, 5 in oral-therapy group). Scores for overall quality of life and health transition at week 96 were missing for one additional participant in the oral-therapy group, so the week 96 values for these items and the summary score was based on 251 participants in this group.

Table S10 Change in MOS-HIV physical and mental health summary scores from baseline to week 96*

	**Long-acting therapy (N=251)**	**Oral therapy (N=251)**	**Difference (95% CI)**	**P**
Physical summary score (PHS) change to week 96	0.3 ± 6.2	0.5 ± 4.2	-0.2 (-1.2 to 0.7)	0.64
Mental summary score (MHS) change to week 96	0.002 ± 8.2	0.02 ± 6.3	-0.02 (-1.3 to 1.3)	0.98

* MOS-HIV denotes the Medical Outcomes Study-HIV questionnaire.

Plus-minus values are mean ± SD. Analysis was performed on complete cases. The assessable population excluded 9 participants who withdrew or died before week 96 (4 in long-acting group, 5 in oral-therapy group) and on additional participant in the oral therapy group with missing data on some items at week 96.

Changes from baseline to week 96 in the PHS and MHS scores were compared between groups using student T-test.

Table S11: Treatment satisfaction score at baseline and change at week 96 *

	**Long-acting therapy (N= 251)**	**Oral therapy (N= 252)**	**Adjusted mean difference (95% CI)**	**P value**
Baseline (HIVTSQs) †	53.7 ± 10.5	52.1 ± 12.6	-	-
Change at week 96 (HIVTSQc) ‡	27.1 ± 11.2	17.9 ± 18.6	9.6 (7.5 to 11.8)	<0.0001

* HIVTSQs denotes HIV Treatment Satisfaction Questionnaire status version. HIVTSQc denotes HIV Treatment Satisfaction Questionnaire change version. Plus-minus values are mean ± SD

† Baseline treatment satisfaction score was obtained using the HIVTSQs administered at baseline. Total score was derived from the first 11 questions, each scored 0 to 6, giving a total score out of 66.

‡ Treatment satisfaction change at week 96 was obtained using the HIVTSQc administered at week 96. Total score was derived from the first 11 questions, giving a permitted range of total scores from -33 to 33. Analysis of HIVTSQc was performed on complete cases, excluding 9 participants who withdrew from the trial or died (4 in long-acting and 5 in oral therapy group). Adjusted HIVTSQc was obtained using linear regression adjusting sex at birth (male, female), baseline CD4 count (<200, ≥ 200 cells/mL), age at baseline (<50, ≥ 50 years), baseline ART third agent class (NNRTI, INI), and site. Treatment groups point estimates were obtained using margins after fitting the adjusted linear regression model.

Figure S1 Transition of participants between BMI categories from baseline to week 96

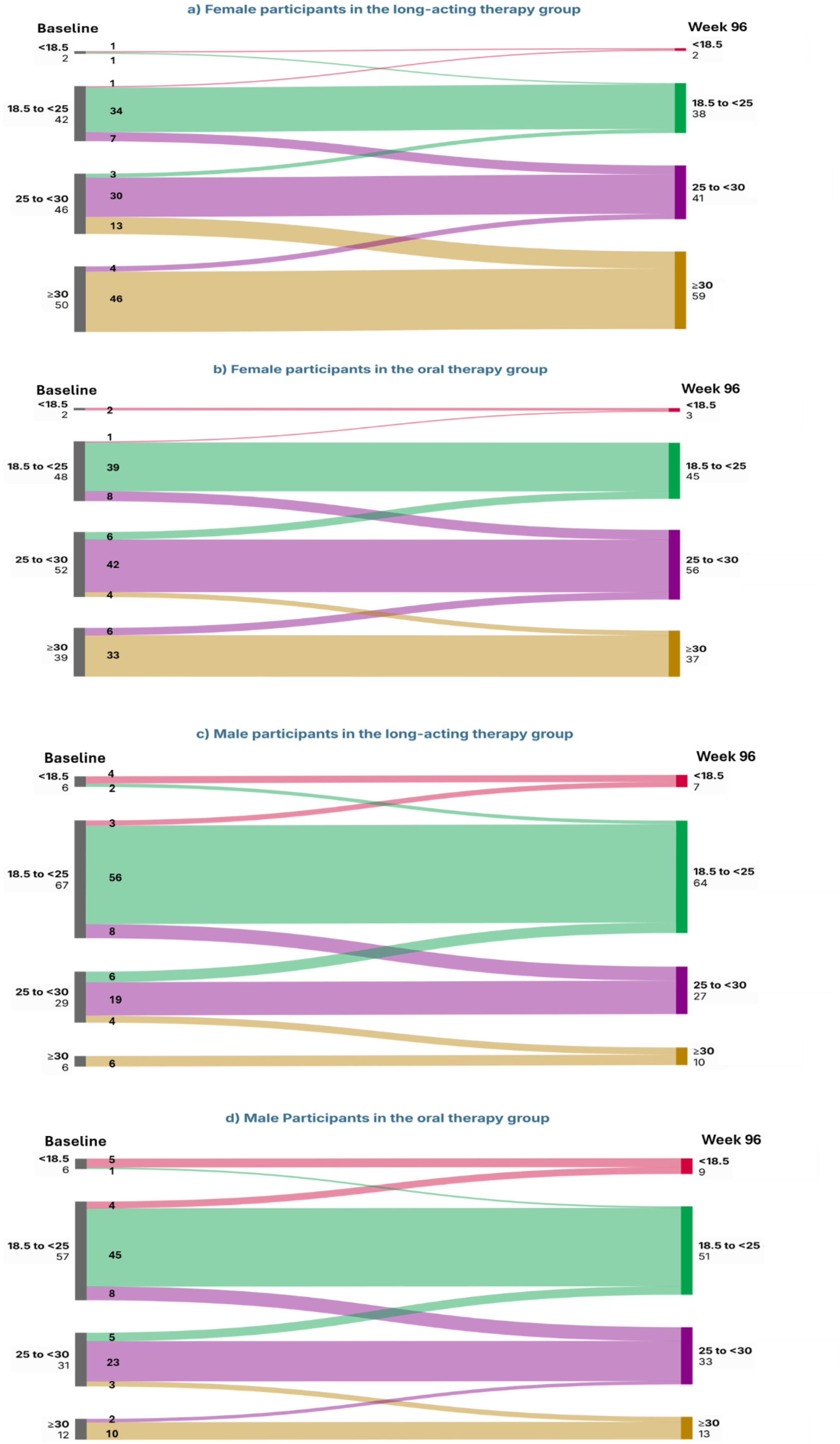

Figure shows the number of participants in the intention to treat exposed population classified in each of the 4 BMI categories (<18.5, 18.5 to < 25, 25 to <30 and ≥30 kg/m^2) at baseline (number to the left of the bars) and week 96 (number to the right of the bars); and the number of participants that transition between each of the BMI categories from baseline to week 96 (number overlying the bars).

The colour of the bars represents the final week 96 BMI category (red, <18.5; green, 18.5 to < 25; purple, 25 to <30; brown, ≥30 kg/m^2), and the width of the bars is proportional to the number of participants in each transition group (i.e. the baseline and week 96 category combination). Comparison of the width of the bars in brown (representing BMI ≥30 kg/m^2 at week 96) between sex and treatment groups illustrates that more women than men are in the category of obese (BMI ≥30 kg/m^2) at baseline and week 96; and more women in the long-acting therapy group transition from the overweight (BMI 25 kg/m^2) to the obese category between baseline and week 96 than do women in the oral therapy group, or men in either treatment group.

Joint Clinical Research Centre (JCRC) & Janssen EMEA *

Clinical Protocol

Protocol Title
A Phase 3b, Randomized, Multicenter, Open-Label Study Evaluating the Efficacy, Safety, and Tolerability of Switching to Long-Acting Cabotegravir Plus Long-Acting Rilpivirine From Current Antiretroviral Regimen in HIV-1 Infected, Virologically Suppressed Adults in Sub-Saharan Africa

---

Short Title
Cabotegravir And Rilpivirine: Efficacy and Safety Study (CARES)

Protocol TMC278LAHTX3002 Amendment 3; Phase 3b
Version: Amendment 3

TMC278 (rilpivirine)

*This is a collaborative study between JCRC, Uganda, acting as Sponsor, and Janssen EMEA as a collaborative partner and funder. Janssen EMEA is a regional organization that operates through different legal entities in various countries.

Status: Approved
Date: 13 December 2023
Prepared by: Janssen-Cilag Limited
EDMS number: EDMS-RIM-230200, 5.0

GCP Compliance: This study will be conducted in compliance with Good Clinical Practice, and applicable regulatory requirements.

Confidentiality Statement
The information provided herein contains Company trade secrets, commercial or financial information that the Company customarily holds close and treats as confidential. The information is being provided under the assurance that the recipient will maintain the confidentiality of the information under applicable statutes, regulations, rules, protective orders or otherwise.

PROTOCOL AMENDMENT SUMMARY OF CHANGES TABLE

DOCUMENT HISTORY	
**Document**	**Date**
Amendment 3	13 December 2023
Amendment 2	16 June 2023
Amendment 1	11 July 2022
Original Protocol	22 November 2020

Amendment 3 (13 December 2023)

Overall Rationale for the Amendment: The protocol was amended to include the social science sub-study participants.

The changes made to the clinical protocol TMC278LAHTX3002 as part of Protocol Amendment 3 are listed below, including the rationale of each change and a list of all applicable sections. Changes made in previous protocol amendments are listed in Section 10.14 Appendix 14: Protocol Amendment History.

Section number and Name	Description of Change	Brief Rationale
Section 6.6. Continued Access to Study Intervention After the End of the Study	Duplicated text regarding continued access to CAB LA+RPV LA was removed from the section.<br><br>The following text was moved to the end of the section:<br>'*Participants in the cART group will continue to receive cART medications as part of the national program.*'	To remove duplicate information.
Section 8.1.4.3. HIV Treatment Satisfaction Questionnaire: HIVTSQ	The section was modified from *'To understand the choice and lived experiences of participants randomized to the CAB LA+RPV LA arm receiving OLI versus those directly transitioning to injectable CAB LA+RPV LA…'*<br><br>to<br><br>*'To understand the choice and lived experiences of participants randomized to the CAB LA+RPV LA arm (including evaluation of differences between those choosing OLI versus those directly transitioning to injectable CAB LA+RPV LA) and in those randomized to the standard of care arm…'*<br><br>The following text was added to the section:<br>*The perceptions and experiences of health care workers managing participants in the trial will also be sought using similar methodology. Approaches to these interviews in all groups are described in detailed interview guides, separately from this protocol.*	To include the social science sub-study participants.
Throughout the protocol	Minor grammatical, formatting, or spelling changes were made.	Minor errors were noted

TABLE OF CONTENTS

PROTOCOL AMENDMENT SUMMARY OF CHANGES TABLE 2

TABLE OF CONTENTS 3

LIST OF IN-TEXT TABLES AND FIGURES 6

1. PROTOCOL SUMMARY 7
1.1. Synopsis 7
1.2. Schema 12
1.3. Schedule of Activities (SoA) 13

2. INTRODUCTION 16
2.1. Study Rationale 17
2.2. Background 17
2.2.1. Comparator Therapy 19
2.3. Benefit-Risk Assessment 19
2.3.1. Oral RPV and RPV LA 20
2.3.2. Oral CAB and CAB LA 22
2.3.3. Other Risks of the CAB LA+RPV LA Regimen 25
2.3.3.1. Other Clinically Relevant Information 26
2.3.4. Significance of Anti-HBc Positivity Among Screen Failures 26
2.3.5. Benefits for Study Participation 26
2.3.6. Benefit-Risk Assessment for Study Participation 26

3. OBJECTIVES AND ENDPOINTS 27

4. STUDY DESIGN 29
4.1. Overall Design 29
4.2. Scientific Rationale for Study Design 31
4.2.1. Study-Specific Ethical Design Considerations 31
4.3. Justification for Dose 32
4.4. End of Study Definition 32

5. STUDY POPULATION 32
5.1. Inclusion Criteria 33
5.2. Exclusion Criteria 34
5.3. Lifestyle Considerations 37
5.4. Screen Failures 37

6. STUDY INTERVENTION AND CONCOMITANT THERAPY 38
6.1. Study Interventions Administered 38
6.2. Preparation/Handling/Storage/Accountability 39
6.3. Measures to Minimize Bias: Randomization and Blinding 40
6.4. Study Intervention Compliance 41
6.5. Dose Modification 41
6.6. Continued Access to Study Intervention After the End of the Study 41
6.7. Treatment of Overdose 42
6.8. Concomitant Therapy 43
6.8.1. Permitted Medications and Non-drug Therapies 43
6.8.2. Prohibited Medications and Non-drug Therapies 44
6.8.3. Prohibited Medications With CAB and/or RPV 44

7. DISCONTINUATION OF STUDY INTERVENTION AND PARTICIPANT DISCONTINUATION/WITHDRAWAL 45
7.1. Discontinuation of Study Intervention 45
7.1.1. Liver Chemistry Stopping Criteria 47
7.1.2. Temporary Discontinuation 48

7.1.3. Rechallenge ... 48
7.2. Participant Discontinuation/Withdrawal From the Study ... 48
7.3. Lost to Follow-up ... 48

8. STUDY ASSESSMENTS AND PROCEDURES ... 50
8.1. Efficacy Assessments ... 51
8.1.1. Viral Load by FDA Snapshot algorithm ... 51
8.1.1.1. Repeat HIV-1 RNA Testing ... 52
8.1.1.2. Alternative Threshold for Testing and Definition of Virologic Failure in the cART Group ... 53
8.1.1.3. Additional samples ... 54
8.1.1.4. Management of Confirmed Virological Failure ... 54
8.1.2. Genotyping ... 54
8.1.3. Immunology ... 54
8.1.4. Patient-Reported Outcomes ... 55
8.1.4.1. EuroQoL-5D-5L Health Questionnaire ... 55
8.1.4.2. Medical Outcomes Study HIV Health Survey: MOS-HIV ... 55
8.1.4.3. HIV Treatment Satisfaction Questionnaire: HIVTSQ ... 55
8.2. Safety Assessments ... 56
8.2.1. Physical Examinations ... 56
8.2.2. Vital Signs ... 57
8.2.3. Clinical Safety Laboratory Assessments ... 57
8.2.4. Pregnancy Testing ... 57
8.2.5. Dual Energy X-ray Absorptiometry Scan ... 57
8.3. Adverse Events, Serious Adverse Events, and Other Safety Reporting ... 58
8.3.1. Time Period and Frequency for Collecting Adverse Event and Serious Adverse Event Information ... 59
8.3.2. Method of Detecting Adverse Events and Serious Adverse Events ... 59
8.3.3. Follow-up of Adverse Events and Serious Adverse Events ... 59
8.3.4. Regulatory Reporting Requirements for Serious Adverse Events ... 60
8.3.5. Pregnancy ... 60
8.3.6. Disease-Related Events and Disease-Related Outcomes Not Qualifying as Adverse Events or Serious Adverse Events ... 60
8.4. Medical Resource Utilization and Health Economics ... 60
8.5. Additional Stored Blood Samples for Characterization of HBV Profile in Screen Failures Based on HBV Serology ... 61

9. STATISTICAL CONSIDERATIONS ... 61
9.1. Statistical Hypotheses ... 62
9.2. Sample Size Determination ... 62
9.3. Populations for Analysis Sets ... 62
9.4. Statistical Analyses ... 62
9.4.1. General Considerations ... 62
9.4.2. Primary Endpoint ... 62
9.4.3. Secondary Endpoints ... 63
9.4.4. Exploratory Endpoints ... 63
9.4.5. Safety Analyses ... 63
9.5. Interim Analysis ... 64

10. SUPPORTING DOCUMENTATION AND OPERATIONAL CONSIDERATIONS ... 66
10.1. Appendix 1: Abbreviations ... 66
10.2. Appendix 2: Clinical Laboratory Tests ... 68
10.3. Appendix 3: Liver Safety: Suggested Actions and Follow-up Assessments ... 69
10.4. Appendix 4: Specific Toxicities ... 74
10.4.1. General Guidance ... 74
10.4.2. Neuropsychological Symptoms ... 75
10.4.3. Cutaneous Events/Rash ... 75
10.4.4. Acute Systemic Allergic Reaction ... 76

10.4.5. Aspartate Aminotransferase and Alanine Aminotransferase Elevation 77
10.4.6. Hepatitis B or C 78
10.4.7. Clinical Hepatitis 79
10.4.8. Renal Complications 79
10.4.9. Nausea (With or Without Vomiting) 79
10.4.10. Diarrhea 80
10.4.11. Hyperglycemia 80
10.4.12. Hypertriglyceridemia and Hypercholesterolemia 81
10.4.13. Lactic Acidosis 81
10.4.14. Lipodystrophy/Fat Redistribution/Body Changes 82
10.4.15. Lipase Elevations 83
10.4.16. Peripheral Neuropathy 83
10.4.17. Injection Site Reactions 83
10.5. Appendix 5: Division of AIDS Table for Grading the Severity of Adult and Pediatric Adverse Events 84
10.6. Appendix 6: CDC Classification for HIV-1 Infection (2014) 107
10.7. Appendix 7: Regulatory, Ethical, and Study Oversight Considerations 109
10.7.1. Regulatory and Ethical Considerations 109
10.7.2. Financial Disclosure 112
10.7.3. Informed Consent Process 112
10.7.4. Data Protection 113
10.7.5. Long-Term Retention of Samples for Additional Future Research 113
10.7.6. Committees Structure 114
10.7.7. Data Quality Assurance 115
10.7.8. Case Report Form Completion 116
10.7.9. Source Documents 116
10.7.10. Monitoring 117
10.7.11. On-Site Audits 118
10.7.12. Record Retention 118
10.7.13. Study and Site Start and Closure 119
10.8. Appendix 8: Adverse Events, Serious Adverse Events, Product Quality Complaints, and Other Safety Reporting: Definitions and Procedures for Recording, Evaluating, Follow-up, and Reporting 120
10.8.1. Adverse Event Definitions and Classifications 120
10.8.2. Attribution Definitions 121
10.8.3. Severity Criteria 121
10.8.4. Special Reporting Situations 121
10.8.5. Procedures 122
10.8.6. Product Quality Complaint Handling 123
10.8.7. Contacting Sponsor Regarding Safety, Including Product Quality 124
10.9. Appendix 9: Contraceptive and Barrier Guidance 125
10.10. Appendix 10: EuroQoL-5D-5L Health Questionnaire (EQ-5D-5L) – Sample UK English Version 128
10.11. Appendix 11: MOS-HIV – 35 Item Instrument 131
10.12. Appendix 12: HIV Treatment Satisfaction Questionnaire 137
10.13. Appendix 13: Guidance on Study Conduct During a Pandemic 138
10.14. Appendix 14: Protocol Amendment History 143

11. REFERENCES 148

INVESTIGATOR AGREEMENT 150

LIST OF IN-TEXT TABLES AND FIGURES

TABLES

Table 1: Summary of Cutaneous Events/Rash 76
Table 2: Summary of Acute Systemic Allergic Reaction 77
Table 3: Summary of AST and ALT Elevations 78
Table 4: Summary of Nausea (With or Without Vomiting) 80
Table 5: Summary of Diarrhea 80

FIGURES

Figure 1: Schematic Overview of the Study 12

1. PROTOCOL SUMMARY

1.1. Synopsis

A Phase 3b, Randomized, Multicenter, Open-Label Study Evaluating the Efficacy, Safety, and Tolerability of Switching to Long-Acting Cabotegravir Plus Long-Acting Rilpivirine From Current Antiretroviral Regimen in HIV-1 Infected, Virologically Suppressed Adults in Sub-Saharan Africa

Cabotegravir And Rilpivirine: Efficacy and Safety (CARES) Study

Long-acting (LA) injectable therapy for the treatment of Human Immunodeficiency Virus (HIV)-1 infection offers a reduced dosing frequency and an additional option to the currently available triple-drug oral combinations. A parenteral LA formulation of rilpivirine (RPV) for intramuscular (IM) injection in combination with ViiV Healthcare's parenteral LA formulation of the integrase inhibitor cabotegravir (CAB) may offer a better tolerability and resistance profile, as well as improved adherence and treatment satisfaction in virologically suppressed patients. The combination regimen has been developed for maintenance of viral suppression (HIV-1 RNA <50 copies/mL) in HIV-1 infected individuals previously treated with standard-of-care antiretroviral therapy.

OBJECTIVES AND ENDPOINTS

Objectives	Endpoints
**Primary**	
• To demonstrate the non-inferior antiviral activity of switching to IM CAB LA+RPV LA administered every 2 months compared with continuation of cART administered daily over 12 months in HIV-1 infected participants in a resource limited setting.	• Proportion of participants with a virologic response (plasma HIV-1 RNA <50 c/mL)* at Month 12
**Secondary**	
• To demonstrate the antiviral and immunologic activity of switching to IM CAB LA+RPV LA every 2 months compared to continuation of cART over 12 and 24 months of follow-up	• Proportion of participants with confirmed virologic failure (2 consecutive plasma HIV-1 RNA levels ≥200 c/mL)** at Month 12 and Month 24.<br>• Proportion of participants with non-response (plasma HIV-1 RNA ≥50 c/mL) at Month 12 and Month 24.<br>• Proportion of participants with virologic response (plasma HIV-1 RNA <50 c/mL) at Month 24.<br>• Change from baseline in CD4+ lymphocyte count at Month 12 and Month 24.<br>• Incidence of disease progression (HIV-associated conditions, AIDS and death) through 12 and 24 months.
• To evaluate the safety and tolerability of switching to CAB LA+RPV LA every 2 months compared to continuation of cART.	• Incidence of Grade 3 and Grade 4 AEs and laboratory abnormalities over time through Month 12 and Month 24.<br>• Proportion of participants who discontinue treatment due to Aes through Month 12 and Month 24.<br>• Absolute values and changes in laboratory parameters through Month 12 and Month 24.
• To assess viral resistance in participants experiencing protocol-defined confirmed virologic failure (plasma HIV-1 RNA ≥200 c/mL).<br>• To assess the incidence of on-treatment genotypic resistance to CAB, RPV and other on-study cART up to Month 12 and 24.	• To assess and compare genotypic drug resistance in individuals with confirmed virologic failure between the CAB LA+RPV LA versus cART.

Objectives	Endpoints
• To evaluate adherence to treatment.	• Adherence, pill count for cART and dosing visits for CAB LA+RPV LA.
**Exploratory**	
• To evaluate the effects of CAB LA+RPV LA every 2 months on BMI over time compared to continuation of cART over time.	• Change from Baseline in BMI over time including Month 12 and Month 24.
• To evaluate the effect of CAB LA+RPV LA on trunk fat compared with cART	• Change from baseline in trunk fat measured by DEXA scan at Month 12 and Month 24
• To explore the effect of patient characteristics (eg, demographic factors, baseline disease characteristics) on the virologic and immunologic responses to CAB LA+RPV LA compared to continuation of cART.	• Proportion of participants by patient subgroup(s) (eg, by age, gender, BMI, HIV-1 subtype) with virologic response (<50 c/mL) over time including Month 12, and 24.<br>• Proportion of participants by patient subgroup(s) (eg, by age, gender, BMI, race, HIV-1 subtype, baseline CD4+ cell count) with plasma HIV-1 RNA >50 c/mL over time including Month 12, and 24.
• Retrospective analysis of archived resistance and virological outcomes using PBMCs at baseline.	• Investigate correlation of archived resistance and HIV-1 subtype determined in baseline PBMCs and virologic outcomes at Month 12 and 24.
• To evaluate the effect of CAB LA+RPV LA on well-being and health status.	• Change from baseline in EQ-5D-5L scores at Month 12 and Month 24.
• To evaluate the effect of CAB LA+RPV LA on quality of life.	• Change from baseline in MOS-HIV at Month 12 and Month 24.
• To assess participant satisfaction with the injectable intervention.	• For participants in the CAB LA+RPV LA group, perception of injection and change from baseline in HIVTSQ scores at Month 12 and Month 24.
• To assess preference for CAB LA+RPV LA compared to oral cART.	• For participants randomized to the CAB LA+RPV LA group, preference for CAB LA+RPV LA compared to oral cART regimen at Month 12 and Month 24 using a single dichotomous preference question.
• To evaluate pharmacokinetics in women who become pregnant on CAB LA+RPV LA.	• Measure antiretroviral plasma concentrations in patients on long-acting antiretrovirals who become pregnant during 1st, 2nd, 3rd trimester and postpartum.
• To evaluate MRU among participants in the study.	• In both study groups, MRU over 24 months:<br>− Rates of opportunistic infections<br>− Rates of hospitalizations.
• To determine the prevalence of HBV DNA among participants on stable cART containing NRTI who test negative for HbsAg and positive for anti-HBc at screening.	• Proportion of participants who test negative for HbsAg and positive for anti-HBc with a detectable HBV viral load at screening.
• To determine the prevalence of anti-HBs positivity among participants on stable cART containing NRTI who test negative for HbsAg and positive for anti-HBc at screening.	• Proportion of participants at screening who test positive for anti-HBs among those who test negative for HbsAg and positive for anti-HBc at screening.

Abbreviations: AE: adverse event; BMI: body mass index; AIDS: Acquired immunodeficiency syndrome; CAB: cabotegravir; cART: combination retroviral therapy; c/mL: copies/milliliter; DEXA: dual energy X-ray absorptiometry; EQ-5D-5L: EuroQol 5 dimension 5 level; HIV: Human Immunodeficiency Virus; HIVTSQs: HIV Treatment Satisfaction Questionnaire; IM: intramuscular; LA: long-acting; MOS-HIV: Medical Outcomes Study HIV Health Survey; MRU: medical resource utilization; OLI: Oral lead-in; PBMC: Peripheral blood mononuclear cells; RPV: rilpivirine; RNA: ribonucleic acid

*HIV-1 RNA levels in the study will be analyzed using the Food and Drug Administration (FDA) Snapshot Algorithm.

**The designated threshold for testing of virologic failure may change during the course of the study in response to changes in national or international treatment guidelines. The new threshold will be declared and adopted consistently for management of all subsequent participants at that site.

Hypothesis

The antiviral effect of switching to CAB LA+RPV LA is non-inferior (margin -10%) to continuation of first-line treatment with daily triple drug oral cART at Month 12 in HIV-1 infected, virologically suppressed participants.

OVERALL DESIGN

CARES is a randomized, open-label, active-controlled, multicenter, interventional study in virologically suppressed (<50 c/mL) HIV-1 infected adult participants to demonstrate that the antiviral effect of switching to IM CAB LA+RPV LA is non-inferior to continuation of first-line cART containing 2 nucleoside reverse transcriptase inhibitor (NRTIs; tenofovir [TDF] plus either lamivudine [3TC] or emtricitabine [FTC]) plus an INI (dolutegravir [DTG]) or a non-nucleoside reverse transcriptase inhibitors (NNRTI) (efavirenz [EFV] or nevirapine [NVP]) at Month 12. After providing written informed consent, participants will be evaluated for eligibility during the 28-day screening period. On Day 1, participants will be randomized to either continue cART or to discontinue cART and begin therapy with CAB LA+RPV LA administered every 2 months. Baseline assessments will be performed on Day 1. Participants randomized to the CAB LA+RPV LA group will be given the option of a 4-week Oral Lead-in (OLI) Phase with oral CAB and oral RPV, or to directly receive the injectable CAB LA+RPV LA. This decision to dose with or without an OLI Phase will be determined by the study participant following informed consent discussions with the investigator.

Starting Day 1, the total duration of the study will be 24 months. Any participant who has received at least a single dose of CAB LA+RPV LA and discontinues the regimen for any reason before Month 24 must start suppressive cART within 2 months of the last LA injection. Investigators must discuss the choice of the follow-up cART regimen with the lead scientist/chief investigator prior to initiating the new regimen with the participant. The participants must remain in the study unless consent is withdrawn, and complete their scheduled assessments up to the Month 24 visit.

Note: Throughout the protocol, visits and analysis timepoints expressed in months from baseline are interpreted as multiples of 4-weeks from baseline ie, Month 12 is interpreted as Week 48 and Month 24 is interpreted as Week 96 from baseline.

NUMBER OF PARTICIPANTS

A target of 512 participants will be enrolled in this study and randomized 1:1 to continue cART or switch to the CAB LA+RPV LA for a treatment period of 24 months. Participants will be recruited across 8 sites from 3 countries (3 sites in Uganda, 3 sites in Kenya and 2 sites in South Africa).

INTERVENTION GROUPS AND DURATION

The interventions received are as follows:

cART Group: Participants will take a regimen of 2 NRTIs (TDF 300 mg + [3TC 300 mg/FTC 200 mg]) + DTG (50 mg)/EFV (600 mg)/NVP (200 mg), as a single tablet or Fixed-Dose Combination regimen as per local country guidelines up to Month 24. Participants will be permitted to switch cART drugs in case of toxicity or for treatment optimization and convenience after viral load testing.

CAB LA+RPV LA Group: The participants who opt for the OLI Phase will receive the study intervention in 2 phases:

Oral Lead-in Phase: Starting on Day 1, participants will receive CAB 30 mg + RPV 25 mg once daily for 4 weeks to be taken at approximately the same time each day with a meal. The purpose of the optional OLI Phase is to allow an opportunity, when desired, for participants to assess tolerability of the combination prior to administration of CAB LA+RPV LA.

Maintenance Phase: After the 4-week OLI Phase, participants will return for the Month 1 visit to take the last dose of oral CAB+RPV at the study-site, and to receive the first IM CAB LA 600 mg + RPV LA 900 mg initiations injections. The second initiation injections with CAB LA 600 mg + RPV LA 900 mg will be administered at Month 2, and then continuation injections will be administered every 2 months thereafter.

Participants who opt for direct CAB LA+RPV LA (ie, without the OLI Phase) injections will remain on cART for 4 weeks after randomization on Day 1, and will receive the first initiation injection of CAB LA+RPV LA at the Month 1 visit. The second initiation injections with CAB LA+RPV LA will be administered at Month 2, followed by continuation injections of CAB LA+RPV LA every 2 months thereafter.

The total duration of the study will be 24 months. At the end of their participation in the study, participants from the CAB LA+RPV LA group who have completed the study and are benefiting from the study intervention, as determined by their investigator, will be able to receive continued access to both CAB LA and RPV LA up to 2 years after study completion or until the participant no longer derives clinical benefit, the participant meets a protocol-defined reason for discontinuation, until CAB LA and RPV LA are registered and reimbursed in the country, or until either CAB LA or RPV LA development program is terminated, whichever occurs earlier. The participant will then be transitioned to a SOC regimen based on the investigator’s clinical judgment and local country guidelines. Participants in the cART group will continue to receive cART medications as part of the national program.

At the end of their participation in the study, participants from the CAB LA + RPV LA group who have completed the study and continue to benefit clinically from the study intervention, as determined by their investigator and are willing to continue treatment, will receive continued access to CAB LA and RPV LA for up to 4 years after study completion. Participants will continue to receive study intervention until:-

- the participant no longer derives clinical benefit;
- the participant meets a protocol-defined reason for discontinuation;
- CAB LA and RPV LA are registered and reimbursed in the country; or
- either CAB LA or RPV LA development program is terminated; whichever occurs earlier.

This continued access will be provided through a post-trial access (PTA) program. If this program is not operational at the end of the study, participants may continue to receive CAB LA and RPV LA via the study in order to avoid treatment interruption. The continued access to CAB LA and RPV LA within the study will serve as a bridge to the operationalization of the post-trial access program. During this bridging, participants will continue to receive care at their respective study sites.

Under the PTA program, Janssen will continue to provide access to CAB LA and RPV LA until patients can obtain the study intervention outside the post-trial access program or through commercial access within local healthcare systems per local/national regulations. Post-trial access will continue for up to four years, after which an evaluation of available local access options will be performed and access determined on a case-by-case basis, based on patient needs and available access options. For the purpose of this study, 'commercial access' means after approval by the competent national authorities for reimbursement under the national health insurance or comparable third-party payment programs.

EFFICACY EVALUATIONS

Key efficacy assessments for the primary and secondary endpoints at Month 12 include virologic response (plasma HIV-1 RNA <50 c/mL), virologic non-response (plasma HIV- RNA ≥50 c/mL) per the FDA snapshot algorithm, and virologic failure (confirmed plasma HIV-1 RNA ≥200 c/mL on 2 consecutive tests 4-6 weeks apart) per the FDA Snapshot Algorithm. In addition, virologic response, non-response and confirmed virologic failure will be documented at Month 24. CD4+ lymphocyte count, and disease progression will also be documented at Month 12 and Month 24.

Disease-related quality-of-life will also be measured using the Medical Outcomes Study HIV Health Survey (MOS-HIV) and EuroQol 5-dimension 5-level (EQ-5D-5L) questionnaire. Participant satisfaction with the injectable intervention will be assessed using the HIV Treatment Satisfaction Questionnaire (HIVTSQ). Treatment preference will be assessed using the treatment preference question among participants in the CAB LA+RPV LA arm. Medical resource utilization will also be evaluated during the study.

SAFETY EVALUATIONS

Key safety assessments will include monitoring of AEs, physical examinations, measurement of body weight, vital sign measurements, clinical laboratory tests and pregnancy testing. Change from baseline in BMI, absolute weight change and weight gain >5% and >10% from baseline will also be assessed.

STATISTICAL METHODS

Assuming a true 94% virologic response rate in each group, a non-inferiority margin of -10%, and a 2-sided 5% significance level, a sample size of 256 participants per treatment group will provide at least 90% power to test the hypothesis of non-inferiority of CAB LA+RPV LA administered every 2 months vs. cART. In addition, to account for a testing of key secondary hypothesis of non-inferiority in virologic failure, this sample size will provide 85% power at a 2-sided 5% significance level with a non-inferiority margin of 4% and assumption of 1.7% virologic failure in a hierarchical testing procedure. At Week 48, the virologic response rate 94% and virologic failure rate 1.7% were observed on Q8W group from the ATLAS-2M study. Therefore, 256 participants per group with total 512 participants are considered adequate for testing the primary and key secondary hypotheses.

For the primary analysis of efficacy, the adjusted estimate of the difference in the rate of responders between the 2 study groups will be presented based on a stratified analysis using Cochran Mantel-Haenszel Weighted Miettinen & Nurminen Method. The analysis will be stratified according to the baseline third agent class (NNRTI or INI). Treatment with CAB LA+RPV LA will be considered non-inferior to cART if the lower limit of the CMH-based 95% confidence interval of the difference in efficacy between the study groups is >-10%.

The key secondary endpoint of virologic failure at Month 12 will be analyzed to test the non-inferiority hypothesis. A hierarchical testing procedure will be used for the secondary endpoint of virologic failure. The secondary hypothesis will be conducted only when the primary hypothesis is significant at 2-sided 5% level of significance to demonstrate non-inferiority. The secondary hypothesis will be then conducted at two-sided 5% level of significance. The non-inferiority will be concluded if the upper bound of the 2-sided 95% confidence interval (CI) of the difference between treatment groups: (CAB LA+RPV LA group – cART group) in virologic failure rate is <4%.

1.2. Schema

Figure 1: Schematic Overview of the Study

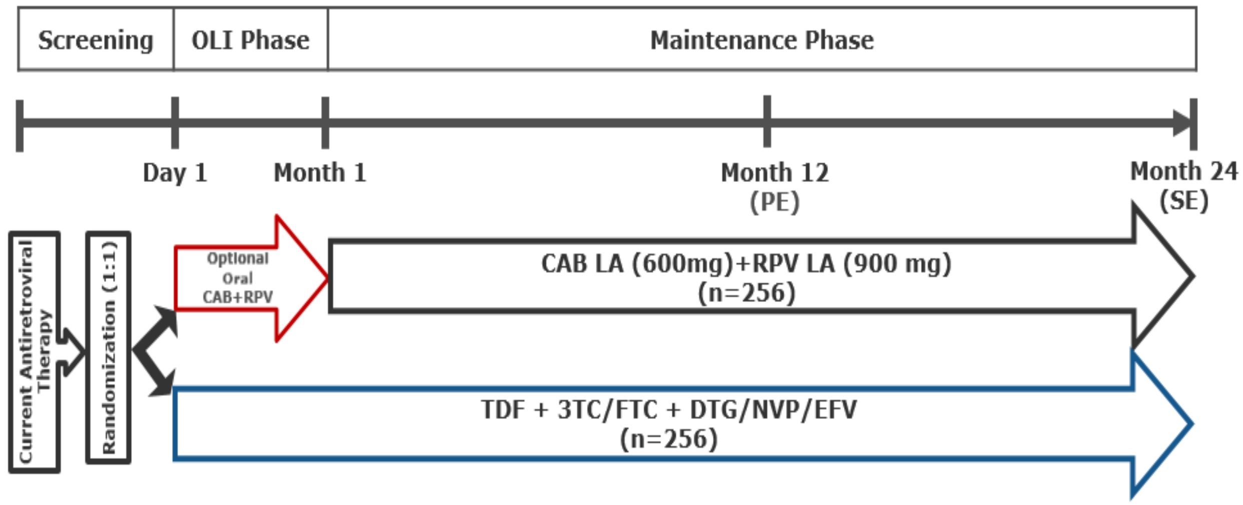

3TC=lamivudine; CAB=Cabotegravir; DTG=dolutegravir; EFV=efavirenz; FTC=emtricitabine; LA=Long Acting; NVP=nevirapine; OLI=Oral Lead-in; PE=Primary Endpoint; RPV=Rilpivirine; SE=Secondary endpoint; SFU=safety follow-up; TDF=tenofovir

1.3. Schedule of Activities (SoA)

Phase		OLI[a]		Maintenance Phase (Months)[b]																						
**Period**	**Scr.**	**Bas.**	**1**	**2**	**3**	**4**	**5**	**6**	**7**	**8**	**9**	**10**	**11**	**12**	**13**	**14**	**15**	**16**	**17**	**18**	**19**	**20**	**21**	**22**	**23**	**24[c]**
**Visit Window (days)**			±8	±7 days																						
**Study visits**																										
CAB LA+RPV LA	X	X	X	X		X		X		X		X		X		X		X		X		X		X		X
cART	X	X			X			X			X			X			X			X			X			X
**Clinical assessments**																										
Informed consent	X																									
Inclusion/exclusion criteria	X	X																								
Medical history and demographics	X	X																								
Adherence/tablet count[d]	X	X	X		X			X			X			X			X			X			X			X
Concomitant medication	X	X	X	X	X	X		X		X	X	X		X		X	X	X		X		X	X	X		X
Vital signs, physical examination	X	X	X		X	X		X						X						X						X
ISR			X	X		X		X		X		X		X		X		X		X		X		X		X
Adverse events		X	X	X	X	X		X		X	X	X		X		X	X	X		X		X	X	X		X
Weight	X	X						X						X						X						X
Height		X																								
**Quality of Life and Healthcare Utilization**																										
MRU								X						X						X						X
MRU survey[e]														X												
EQ-5D		X						X						X						X						X
MOS-HIV		X												X												X
HIVTSQ$_S$		X			X	X																				
HIVTSQ$_C$								X						X						X						X
Treatment preference question														X												X
**Imaging and ECG**																										
Chest X-ray	X																									
ECG	X																									
DEXA[f]		X												X												X
**Urine**																										
Urine pregnancy test	X	X	X	X	X	X		X		X	X	X		X		X	X	X		X		X	X	X		X
Urinalysis[g]								X						X												X
**Blood**																										

**Phase**		**OLI[a]**		**Maintenance Phase (Months)[b]**																						
**Period**	**Scr.**	**Bas.**	**1**	**2**	**3**	**4**	**5**	**6**	**7**	**8**	**9**	**10**	**11**	**12**	**13**	**14**	**15**	**16**	**17**	**18**	**19**	**20**	**21**	**22**	**23**	**24[c]**
**Visit Window (days)**			**±8**	**±7 days**																						
HIV-1 RNA [h]	X	X						X						X						X						X
CD4	X	X						X						X												X
Full blood count, biochemistry [i]	X	X	X		X	X		X						X						X						X
Fasting cholesterol, triglycerides		X												X												X
HBVsAg, anti-HBc, HCV	X																									
Store PBMC [j]		X																								
Store plasma [k]	X [l]	X																								
Store serum [m]	X																									

Abbreviations: DEXA**:** dual energy X-ray absorptiometry; ECG: electrocardiogram; HBc: Hepatitis B core antibody; HBsAg: hepatitis B surface antigen; HCV: Hepatitis C virus; HIV: Human Immunodeficiency Virus; HIVTSQs: HIV Treatment Satisfaction Questionnaire; ISR: Injection site reaction; MOS-HIV: Medical Outcomes Study HIV Health Survey; MRU: Medical resource utilization; OLI: Oral lead-in; PBMC: Peripheral blood mononuclear cells; RNA: ribonucleic acid; TB: tuberculosis

Footnotes:

a. Optional for the CAB LA+RPV LA group only after participant discussion with the investigator at the time of informed consent.

b. Visits and analysis timepoints expressed in months from baseline are interpreted as multiples of 4-weeks from baseline throughout the protocol ie., Month 12 is interpreted as Week 48 and Month 24 is interpreted as Week 96 from baseline.

c. Perform Month 24 assessments in all patients who withdraw early from the study, within 72 hours of withdrawal; and to the extent that participants are willing.

d. Adherence assessment and tablet count only in participants receiving cART, or oral lead-in/bridging treatment in CAB LA+RPV LA group.

e. Detailed healthcare resource data survey to be performed at selected sites only.

f. DEXA measurement of body fat performed at selected sites only and in subset of patients; requires separate written informed consent; first scan must be performed within 1 month of baseline visit in the main trial.

g. Urinalysis for protein and glucose.

h. Additional viral load testing after 4-6 weeks in participants with viral load ≥200 c/mL in CAB LA+RPV LA group; additional plasma sample stored (for batched, blinded viral load testing) after 4-6 weeks in participants with viral load ≥200 c/ml in cART arm; and additional viral load after 12 (10-16) weeks in participants with viral load ≥1000 c/mL in cART arm; resistance testing performed in participants with ≥200 c/mL in 2 consecutive tests 4-6 weeks apart in the CAB LA+RPV LA group or ≥1000 c/mL in 2 consecutive tests 10-16 weeks apart in the cART group.

i. Full blood count: hemoglobin, total white cell count, neutrophil count, platelets. Biochemistry: sodium, potassium, creatinine, glucose, ALT, bilirubin (total, direct and indirect), alkaline phosphatase; additional full blood count and biochemistry performed 4 weeks after discontinuation of CAB+RPV

j. PBMC stored for retrospective testing of archived genotypic drug resistance.

k. Plasma (one 5ml EDTA sample) stored for future research at the following time-points (requires additional consent at study entry): at baseline visit and at each visit where blood is taken for confirmation of viral load rebound (for future additional resistance testing or biomarker tests; both arms); at 1, 3, 6 and 12 months following cessation of CAB LA+RPV LA in patients who develop TB (for drug levels; CAB LA+RPV LA group only); at 1st, 2nd and 3rd trimester and postpartum in pregnant women (for drug levels; LA arm only).

l. Plasma (4ml EDTA) stored at screening for batched testing for HBV DNA.

m. Serum (4ml redtop) stored at screening for batched testing for anti-HBs.

2. INTRODUCTION

The development of innovative antiretroviral (ARV) compounds for the treatment of human immunodeficiency virus type 1 (HIV-1) should target drugs with high barrier to resistance, improved tolerability, safety and simplified fixed-dose combination (FDC) regimens. Currently, most patients infected with HIV-1 are treated with a combination of 3 or 4 drugs, including nucleo(t)side reverse transcriptase inhibitors (N[t]RTIs), non-nucleoside reverse transcriptase inhibitors (NNRTIs), protease inhibitors (PIs) and/or integrase inhibitors (INIs) (CROI, 2020; DHHS. Panel on Antiretroviral Guidelines for Adults and Adolescents, 2016).

Virologic suppression and treatment response in HIV-1 infected patients are primarily dependent on adherence to antiretroviral therapy (ART). The pill burden of currently available treatments, dosing frequency, and safety concerns frequently contribute to non-compliance and the emergence of drug-resistant virus in resource limited settings (Nachega, 2012). Further simplifying chronic treatment is important for increasing treatment compliance and improving the quality of life for patients living with HIV. The need to develop NRTI-sparing regimens for long-term treatment remains, to avoid known NRTI-associated adverse drug reactions and long-term toxicities (Pau, 2014).

In 2016, the WHO included an INI (dolutegravir [DTG])-based regimen as an alternative first-line treatment for HIV. Dolutegravir is associated with fewer drug interactions with a higher genetic barrier to resistance, and the single tablet combination of tenofovir/lamivudine/DTG is now included as the preferred first-line treatment option in more than 50 low- and middle-income countries (WHO Briefing Note, 2018).

Single tablet, fixed-dose two-drug regimens, JULUCA® (rilpivirine [RPV] plus DTG), DOVATO® (DTG plus lamivudine), have been approved for the treatment of HIV-1 infection in patients switching from stable ART and treatment-naïve patients, respectively (FDA Homepage, Accessed August 24, 2020). These two-drug oral regimens eliminate additional toxicity, potential drug interactions, and the third drug/class is spared in case of future drug resistance.

Long-acting (LA) injectable therapy offers a greatly reduced dosing frequency and an additional option among two-drug oral regimens now available (Fernandez, 2019). A parenteral LA formulation of RPV for intramuscular (IM) injection in combination with ViiV Healthcare's parenteral LA formulation of the INI cabotegravir (CAB) is in Phase 3b of development (Orkin, 2019; Swindells, 2020). This two-drug combination CAB LA+RPV LA may offer a better treatment option for virologically suppressed patients due to less frequent dosing and therefore reduced stigma, improved adherence, treatment satisfaction leading to longer retention in care (Dolgin, 2014). There are also no food requirements associated with LA injectables, and NRTI-related adverse events, gastrointestinal (GI) adverse events and drug-drug interactions in the GI tract can be avoided. The combination regimen has been developed for maintenance of viral suppression in HIV-1 infected individuals and has received marketing approval as Cabenuva® (co-packaged product, marketed by ViiV) in Canada on 18 March 2020.

For the most comprehensive nonclinical and clinical information regarding CAB LA+RPV LA, refer to the latest version of the Investigator's Brochure (IB) for RPV and CAB (EDURANT, Investigator Brochure, 2020; TIVICAY, Investigator Brochure, 2015).

2.1. Study Rationale

The CAB LA+RPV LA regimen offers the benefit of reduced dosing from daily pills to IM injections every 2 months. This could have a positive impact on the patient's quality-of-life and has the potential to improve adherence to treatment and engagement in care and reduce the stigma associated with the daily use of oral pills.

Cabotegravir LA+RPV LA administered on a monthly schedule (Q4W) has demonstrated non-inferiority to oral triple therapy standard-of-care (SOC) in Phase 3 studies (ATLAS [201585], FLAIR [201584]) in virologically suppressed patients (HIV-1 RNA <50 copies/mL). The eligible participants had to be on a stable cART regimen for at least 6 months and had no history of virologic failure and no history of resistance to any NNRTI or INI. The Phase 3b study ATLAS-2M demonstrated the non-inferiority of the every 2 months schedule compared to the monthly administration.

The current study aims to investigate whether CAB LA+RPV LA administered every 2 months is non-inferior to SOC in resource limited settings, where the majority of the population affected by HIV-1 consists of black African women, and where access to regular clinical and laboratory monitoring is more challenging. Furthermore, in most Sub-Saharan Africa (SSA) countries, resistance to NNRTIs has reached levels greater than 10% (WHO HIV Drug Resistance Report, 2017), which has necessitated a switch to INI-based regimens for durable virologic control.

This study also aims to bridge other data gaps specific to SSA including women of childbearing potential and pregnant women and HIV-1 subtypes in different regions across SSA.

The term "study intervention" throughout the protocol, refers to the study intervention as defined in Section 6.1, Study Interventions Administered. The term "sponsor" used throughout this document refers to the entities listed in the Contact Information page(s), which will be provided as a separate document. The term "participant" throughout the protocol refers to the common term "subject".

2.2. Background

The details on the nonclinical and clinical studies with RPV oral and parenteral formulations are presented in the sections below. Please refer to the IB for CAB (TIVICAY, Investigator Brochure, 2015), for details on the nonclinical studies with CAB oral and parenteral formulation.

Clinical Studies With Rilpivirine

Human Pharmacokinetics

Multiple dose administration of RPV LA (3 monthly doses of 1200, 600 and 600 mg) was evaluated in a panel of healthy participants in study C158. After each IM injection of RPV LA,

RPV plasma concentrations rapidly increased over the first days, with a median t_{max} 2-3 days after injection. Thereafter, there was a more gradual release of RPV from the injection site, resulting in a stable plasma concentration-time profile.

Following administration of multiple IM injections of RPV LA in study TMC278-MWRI-01, maximum plasma concentrations of RPV were achieved by 7 days after dosing. Drug accumulation was evident in all anatomical compartments after repeat injections of the RPV LA formulation. There was an apparent prolongation of the t_{max} in the female genital tract with repeat dosing. Results from study LAI11542834 in healthy adult participants showed that successive monthly IM injections of RPV LA (1200/900 mg or 1200/600 mg) in combination with CAB LA give plasma concentrations comparable to those of oral RPV 25mg taken twice daily.

The 200056 (LATTE-2; ViiV study) study analyzed the mean (SD) RPV plasma concentrations over time after monthly (Q4W) or every 2 months (Q8W) IM injections of RPV LA 600 mg or 900 mg, respectively, in HIV-1 infected adult participants. Both RPV LA dosing regimens result in a prolonged plasma release of RPV with gradual increase to a mean RPV pre-dose plasma concentration at Week 48 of 95 ng/mL for the Q4W regimen and 64 ng/mL for the Q8W regimen.

The 201585 (ATLAS; ViiV study) and 201584 (FLAIR; ViiV study) trials analyzed monthly IM injections of RPV LA 600 mg (with first IM dose RPV LA 900 mg at Week 4 after oral RPV 25 mg q.d.) in HIV-1 infected adults. Geometric mean RPV plasma concentrations at Week 48 were 86 ng/mL, similar to pharmacokinetics after monthly dosing in Phase 2 study LATTE-2.

Efficacy/Safety Studies

The LATTE-2 study was a Phase 2b study to demonstrate non-inferiority of CAB LA+RPV LA every 8 weeks (Q8W) compared with CAB LA+RPV LA every 4 weeks (Q4W) in treatment-naïve participants. After 48 weeks, Q4W dosing resulted in modestly lower rates of virologic non-response to Q8W (1 participant with Q4W vs 8 participants with Q8W) and was at that time the selected dose for pivotal Phase 3 studies. At Week 96, viral suppression was maintained at 87% with Q4W dosing and 94% with Q8W. The Week 160 results showed that the combination of RPV LA and CAB LA effectively maintained viral load <50 c/mL for 160 weeks (ie, 160 weeks of maintenance therapy) at rates of 83% with Q4W dosing and 90% with Q8W. These long-term results supported the further evaluation of Q8W dosing in a Phase 3b study.

The FLAIR study was a Phase 3 study to demonstrate non-inferior antiviral activity of switching to RPV LA + CAB LA compared with remaining on combination antiretroviral therapy (cART) in HIV-1 infected ARV treatment-naïve adult participants. The primary efficacy analysis demonstrated that Q4W CAB+RPV was non-inferior to CAR in maintaining virologic suppression in HIV-1 infected subjects at Week 48, with the proportion of subjects having plasma HIV-1 RNA ≥50 c/mL at Week 48 per the Snapshot Algorithm. Overall, these results support the therapeutic potential of Q4W CAB LA+RPV LA. The Week 96 data further demonstrated durability of HIV-1 RNA suppression (<50 c/mL) with CAB LA+RPV LA and non-inferiority to cART (86.6% on CAB LA+RPV LA and 89.4% on cART). Patients reported a significantly greater increase in treatment satisfaction with LA regimen over time vs cART.

The ATLAS study is an ongoing Phase 3 study to demonstrate non-inferior antiviral activity of switching to CAB LA+RPV LA compared with remaining on cART in HIV-1 infected ART-experienced adult participants. The Week 48 data showed that the regimen of Q4W injections of CAB LA+RPV LA was non-inferior to continued 3-drug oral ART at Week 48 for maintaining suppression of HIV-1 (93% on CAB LA+RPV LA vs 95% on cART). Participants continuing in the study at Week 96 had sustained viral load suppression <50 c/mL (100% in CAB LA+RPV LA group; 97% in the Extension Switch to CAB LA+RPV LA group). Patients reported a significantly greater increase in treatment satisfaction with LA regimen over time versus cART. These data continue to support the investigation of the CAB LA+RPV LA regimen for the management of HIV-1 infection.

The ATLAS-2M study is an ongoing Phase 3b study in HIV-1-infected adults switching from the SOC to the CAB LA or RPV LA Q4W or Q8W regimen, or from CAB LA+RPV LA Q4W to the Q8W regimen, or remain on the CAB LA + RPV LA Q4W regimen, to demonstrate non-inferiority of the CAB LA+RPV LA Q8W regimen compared with the Q4W regimen. The study showed that Q8W dosing was non-inferior to Q4W dosing at the 48-week mark for both the key virologic outcomes-viral suppression (94.3% vs 93.5%) and viral load ≥50 c/mL (1.7% and 1.0%). The safety profiles of both dosing regimens were similar, with only mild or moderate injection site reactions (ISRs) reported. Of the ATLAS-2M participants who were treated with the Q8W regimen after having received >48 weeks of Q4W treatment in ATLAS, 93% (115/124) reported a preference for Q8W dosing. These results support the Q8W CAB LA+RPV LA as a treatment option for HIV-1 infected participants who are virologically suppressed for continued maintenance therapy.

Safety data from Phase 2b and 3/3b clinical studies conducted by ViiV Healthcare (200056, 201584, 201585, and 207966) support a favorable safety profile of CAB LA+RPV LA, both for monthly and for every 2 months dosing.

2.2.1. Comparator Therapy

The World Health Organization (WHO) has published the consolidated guidelines on the use of ARV drugs for treating HIV infection and recommended tenofovir disoproxil fumarate (TDF)+lamivudine (3TC) (or emtricitabine, FTC)+efavirenz (EFV) 600 mg as the preferred first-line ART regimen in adults in a resource limited setting. Since 2018, these guidelines have been updated to include DTG. Participants in the control group will continue their current ARV regimen of 2 NRTIs (tenofovir [TDF] plus either 3TC or FTC) plus an INI (DTG) or a NNRTI (EFV or NVP) per local country guidelines. Participants will be permitted to switch cART drugs in case of toxicity, or for treatment optimization and convenience; viral load must be tested and shown to be <1000 c/mL prior to elective treatment switch; there must be confirmed virologic rebound to ≥1000 c/mL prior to switch for treatment failure.

2.3. Benefit-Risk Assessment

More detailed information about the known and expected benefits and risks of oral and LA CAB or oral and LA RPV can be found in the Investigator's Brochures for rilpivirine and cabotegravir, respectively (EDURANT, Investigator Brochure, 2020; TIVICAY, Investigator Brochure, 2015).

Oral RPV is an approved medicinal product and detailed information on its benefit/risk profile together with any risk mitigation measures are described in local product labeling (Edurant®).

Current ART administered in this protocol has 2 NRTIs plus an NNRTI or INI as the third agent. These regimens are established treatments that have been in clinical use for several years and detailed information on the well-established benefit/risk profiles together with risk mitigation measures are described in their respective local product labels.

The following section outlines the risk assessment and mitigation strategy for this protocol:

2.3.1. Oral RPV and RPV LA

The following risks have primarily been identified for oral and LA RPV during routine preclinical testing and/or in the clinical trial experience to date and are considered of potential relevance to clinical usage in the context of this protocol. Additional information about the clinical experience to date and possible risks associated with treatment using RPV can be found in the Summary of Data and Guidance for the Investigator section of the IB.

**ORAL RPV (TMC278)**<br>For safety and risk mitigation for oral RPV refer to the RPV local prescribing information [Edurant® Product Information].<br>**RPV LA (TMC278 LA)**<br>Information about the clinical experience to date and possible risks associated with treatment using RPV LA can be found in the Summary of Data and Guidance for the Investigator section of the IB. Beyond what has already been identified with oral RPV, no new systemic adverse reactions to RPV LA (same active moiety) have been observed. The following risks are considered to be of specific clinical relevance in the context of IM use.		
Injection Site Reactions	Clinical experience to date has demonstrated ISRs occur in the majority of exposed participants treated with RPV LA but are generally mild (Grade 1) or moderate (Grade 2) and include events of pain, tenderness, erythema, or nodule formation of several days duration (median duration for individual events <1 week). ISRs may occur more than once in an individual participant receiving multiple injections. Although some Grade 3 ISRs were reported, overall ISRs have been well tolerated and have not to date been associated with an excess of participants' withdrawal due to ISRs.<br>None of the ISRs was serious and no clinical significant complications were reported.	• Administration advice to minimize risk of poor administration technique giving rise to injection site reactions. Advice on care, monitoring, natural course, and treatment of ISRs given in study documentation.<br>• Advice to participants on care of injection site on day/days immediately post administration, use of analgesia, compresses where appropriate.<br>• Participants will be closely monitored for ISRs particularly for signs of pain, tenderness, infections, erythema, swelling, induration, or nodules (granulomas or cysts) throughout the study.<br>• Complications of ISRs such as infections (abscess, cellulitis) and collections of fluid requiring drainage will be monitored.<br>• Significant ISRs may be photographed and referred to a dermatologist for specialist advice.
Rash	Some observations of rash with oral RPV have been reported in clinical studies executed to date (the majority are Grade 1 or 2).	• In this study, RPV LA administration will be preceded by an oral RPV lead-in to evaluate tolerability in individual participants.

		• Participants with a Grade 1 or 2 rash will be allowed to continue treatment or to be rechallenged, depending on the clinical judgment of the investigator.<br>• All participants experiencing a Grade 3 or 4 rash should discontinue their ARV medication (study medication and background regimen) and be withdrawn from the study but will be followed-up to assess resolution of the event.<br>• All rash events should be assessed with special attention to systemic symptoms, laboratory abnormalities, or mucosal involvement. Close clinical follow-up, including follow-up of laboratory abnormalities, and appropriate medical intervention, including referral to dermatologist as appropriate, should be instituted for these events; daily follow-up is recommended for 5 days from the onset of the event to monitor for progression of the event. See Section 10.4.3 for additional guidance on management of rash events.
Development of Resistance	Residual concentrations of RPV LA would remain in the systemic circulation of participants who stopped treatment (eg, for tolerability issues or treatment failure) for prolonged periods (up to 12 months or longer).<br>Participants discontinuing a LA regimen may be at risk for developing resistance to RPV many weeks after discontinuing injectable therapy.	• Alternative oral cART regimens will be prescribed within 2 months after the last dose of LA therapy. This would be anticipated to result in rapid resuppression of HIV-1 RNA thus minimizing the risk of emergent resistance.
Drug-Drug Interactions	For a detailed listing of permitted and prohibited concurrent medications for RPV and RPV LA, refer to Section 6.8. RPV LA should not be co-administered with the following medicinal products, as significant decreases in RPV plasma concentrations may occur (due to CYP3A enzyme induction), which may result in loss of therapeutic effect of RPV LA.<br>– the anticonvulsants carbamazepine, oxcarbazepine, phenobarbital, phenytoin<br>– the antimycobacterials rifampicin, rifapentine, rifabutin<br>– the glucocorticoid systemic dexamethasone, except as a single dose treatment<br>– St John's wort (Hypericum perforatum).<br>Of note, evidence to date indicates that clinically relevant DDIs with RPV LA and other antiretrovirals are unlikely to occur, therefore there are no limitations to the	All participants will be informed of prohibited medications throughout the study and updates provided as needed via informed consent.

	alternative ARV regimen after stopping RPV LA, from a DDI perspective.<br>Oral RPV administration only:<br>– Antacid products containing divalent cations (eg, aluminum, calcium and magnesium) must be taken at least 2 hours before or at least 4 hours after RPV.<br>– H_2-antagonists must be taken at least 12 hours before or at least 4 hours after taking RPV.<br>– RPV should not be co-administered with proton pump inhibitors, such as esomeprazole, lansoprazole, omeprazole, pantoprazole, rabeprazole;	
Inadvertent Intravenous Injection (Accidental Maladministration)	As with any intramuscular injection, it is possible that RPV LA can be inadvertently administered intravenously instead of intramuscularly resulting in higher than expected concentrations of RPV. This could be due to administrator error, improper injection technique and / or improper needle length used based on body type.<br>In clinical trials, serious post-injection reactions were reported within minutes after the injection of RPV, including dyspnea, agitation, abdominal cramping, flushing, sweating, oral numbness, and changes in blood pressure. These events occurred in 0.01% of injections (4/39626) and in less than 0.5% (4/1667) of patients who received injections and began to resolve within a few minutes after the injection. These events may have been associated with inadvertent (partial) intravenous administration.	• Training will be provided to all sites on proper injection technique.<br>• Should IM maladministration be suspected at any time (eg, suspected under or overdose or inadvertent IV dosing), post dose ECG monitoring and vital signs or any other supportive testing may be obtained at the discretion of the investigator, and the lead scientist/chief investigator notified.<br>• Additionally, an unscheduled PK sample may be drawn for future evaluation of RPV concentrations.<br>• Laboratory samples for safety parameters and HIV-1 RNA will be closely monitored in all participants.
Post-injection Reactions	In clinical trials, serious post-injection reactions have been reported within minutes after the injection of rilpivirine, including dyspnea, agitation, abdominal cramping, flushing, sweating, oral numbness, and changes in blood pressure. These events occurred in approximately 0.01% of injections (4/39626) and in less than 0.5% (4/1667) of subjects who received injections. These events began to resolve within a few minutes after the injection and may have been associated with inadvertent (partial) intravenous administration.	• Training will be provided to all sites on proper injection technique.<br>• Site-staff will be instructed to follow the Instructions for Use when preparing and administering RPV LA to avoid accidental intravenous administration. If a patient experiences a post-injection reaction, they will be monitored and treated as clinically indicated.

2.3.2. Oral CAB and CAB LA

The following risks have primarily been identified for oral and LA CAB during routine preclinical testing and/or in the clinical trial experience to date and are considered of potential relevance to clinical usage in the context of this protocol. Additional information about the clinical experience

to date and possible risks associated with treatment using CAB can be found in the Summary of Data and Guidance for the Investigator section of the IB.

Potential Risks of Clinical Significance	Summary of Data/ Rationale for Risk	Mitigation Strategy
Drug Induced Liver Injury (DILIs)	A small proportion of participants in the CAB program to date (total exposure approximately 1198 to 01 April 2016) have developed transaminitis (elevated liver transaminases characterized by predominant Alanine aminotransferase (ALT) elevation). In some of these participants' transient transaminitis were explained by acute hepatitis C infection while a small number of others did not have alternative explanations, suggesting a mild form of DILI without hepatic dysfunction which resolved upon withdrawal of treatment with CAB.<br><br>Of the 5 participants with possible or probable cases of DILI identified in Phase 2 studies, 4 participants were receiving oral CAB and one participant developed probable DILI following CAB IM or Placebo IM administration.	• Exclusion criteria as described in Section 5.2 will prohibit participants with significant liver impairment based on screening liver chemistry including transaminases (ALT and Aspartate aminotransferase [aspartate transaminase (AST)]) as well on prior medical history. Participants with a history of chronic liver disease with ongoing inflammation and/or fibrosis will have additional confirmatory assessments to confirm suitability for entry into the study.<br>• A 4-week Oral Lead- in Phase is being implemented in this study, where all participants will receive oral CAB prior to the administration of IM CAB to determine individual safety and tolerability.<br>• Liver transaminases (ALT and AST) will be monitored in this study (refer to Time & Events Table) and the liver chemistry stopping criteria will be adopted as described in Section 7.1.1 of this protocol.<br>• Participants will be withdrawn from CAB treatment where no compelling alternative cause is identified, and DILI is suspected. Please refer to Appendix 3 for suggested actions and follow-up in case of liver abnormalities.
Injection Site Reactions (ISRs)	Clinical, experience to date has demonstrated ISRs occur in the majority of exposed participants treated with CAB LA but are generally mild (Grade 1) or moderate (Grade 2) and include events of pain, tenderness, erythema, or nodule formation of several days duration (median duration for individual events <1 week). ISRs may occur more than once in an individual participant receiving multiple injections. Although some Grade 3 ISRs were reported, overall ISRs have been well tolerated and have not to date been associated with an excess of participants' withdrawal.<br>None of the ISRs reported to date was serious and no clinically significant complications were reported.	• Administration advice will be given to minimize risk of poor administration technique giving rise to ISR. Advice on care, monitoring, natural course, and treatment of ISRs is given in study documentation.<br>• Advice will be given to participants on care of injection site on day/days immediately post administration, use of analgesia, compresses where appropriate.<br>• Participants will be closely monitored for ISRs particularly for signs of pain, tenderness, infections, erythema, swelling, induration, or nodules (granulomas or cysts) throughout the study.

Potential Risks of Clinical Significance	Summary of Data/ Rationale for Risk	Mitigation Strategy
		• Complications of ISRs such as infections (abscess, cellulitis) and collections of fluid requiring drainage will be monitored.<br>• Significant ISRs may be photographed and referred to a dermatologist for specialist advice.
Hypersensitivity Reactions (HSR)	Hypersensitivity reactions have been reported as uncommon occurrences with integrase inhibitors, including the closely related compound DTG, and were characterized by rash, constitutional findings, and sometimes, organ dysfunction, including liver injury.<br><br>While there have been no clinical cases of hypersensitivity to CAB, there is a theoretical risk of systemic or severe hypersensitivity reactions with or without hepatic symptoms associated with use of IM CAB. The long exposures anticipated after IM CAB injection may complicate the management of a drug hypersensitivity reaction, were it to occur.	• The risk of developing a hypersensitivity reaction post administration of IM CAB will be minimized by the use of a 4-week oral lead-in of oral CAB to determine individual safety and tolerability prior to the introduction of IM CAB.<br>• Clinical assessments, laboratory tests (including liver transaminases) and vital signs will be performed throughout this study (refer to Schedule of Activities, Section 1.3). Results from these assessments may aid early detection of HSR.<br>• Oral CAB will be withdrawn immediately for cases with suspected HSR during the oral CAB Lead-in Phase and would not proceed to the injection phase. Participants in the injection phase would not receive further injections. During oral and IM CAB treatment, any HSR reactions that occur would be managed supportively.
Development of Resistance following discontinuation of CAB LA	Residual concentrations of CAB would remain in the systemic circulation of participants who stopped CAB LA treatment for prolonged periods (up to 1 year) despite stopping treatment (eg, for tolerability issues or treatment failure). Participants discontinuing CAB LA regimen may be at risk for developing HIV-1 resistance to CAB many weeks after discontinuing injectable therapy.	• Alternative oral cART regimens will be prescribed within a maximum of 2 months after the last dose of CAB LA. This would be anticipated to result in rapid resuppression of HIV-1 RNA thus minimizing the risk of emergent resistance.
Drug-Drug Interactions (DDIs)	For a detailed listing of permitted and prohibited concurrent medications for CAB and CAB LA, refer to Section 6.13<br>CAB and CAB LA should not be co-administered with the following medicinal products, as significant decreases in CAB plasma concentrations may occur (due to UGT enzyme induction), which may result in loss of therapeutic effect of CAB.<br>- the anticonvulsants carbamazepine, oxcarbazepine, phenobarbital, phenytoin<br>- the antimycobacterials rifampicin, rifapentine, rifabutin	• All participants will be informed of prohibited medications throughout the study and updates provided as needed via the informed consent.<br>• Participants in the CAB LA+RPV LA group who are diagnosed with tuberculosis will stop the LA treatment and immediately start suppressive cART and TB treatment. They will be followed-up for at least 12 months after the last dose of CAB LA+RPV LA. Plasma samples will be collected from these participants 1, 3, 6 and 12 months

Potential Risks of Clinical Significance	Summary of Data/ Rationale for Risk	Mitigation Strategy
	- St John's wort (Hypericum perforatum).<br>Chronic use of oral glucocorticoids must be avoided; however, short treatment courses (for example, 14 days or less) and topical, inhaled or intranasal use of glucocorticoids will be allowed.<br>Oral CAB administration only: Antacid products containing divalent cations (eg, aluminum, calcium and magnesium) must be taken at least 2 hours before or at least 4 hours after CAB.<br>Participants in the cART group who are diagnosed with tuberculosis will be treated as clinically indicated by their ART regimens. Participants in the CAB LA+RPV LA group who are diagnosed with tuberculosis will have the treatment stopped.	after the last dose of CAB LA+RPV LA for measurement of levels of ART drugs.
Inadvertent Intravenous Injection (Accidental Maladministration)	As with any intramuscular injection, it is possible that CAB LA can be inadvertently administered intravenously instead of intramuscularly resulting in higher than expected concentrations of CAB. This could be due to administrator error, improper injection technique and / or improper needle length used based on body type. The clinical consequences of overdose with CAB LA are currently unknown.<br>HIV-1 viral suppression may not be effective following accidental maladministration.	• Training will be provided to all sites on proper injection technique.<br>• Should IM maladministration be suspected at any time (eg, suspected under or overdose or inadvertent IV dosing), a post dose electrocardiogram (ECG), vital signs, or any other supportive testing may be obtained at the discretion of the investigator, and the lead scientist/chief investigator will be notified.<br>• Laboratory samples for safety parameters and HIV-1 RNA will be closely monitored in all participants.<br>• Additionally, an unscheduled PK sample may be drawn for future evaluation of CAB concentrations.

2.3.3. Other Risks of the CAB LA+RPV LA Regimen

Other Risks		
Risk of Treatment Failure	This study employs a novel maintenance approach to the treatment of HIV-1 infection. Following viral suppression, participants will be transitioned off a 3 drug ART regimen to a 2 drug LA ART regimen that remains experimental. Although both IM CAB and RPV have demonstrated antiviral activity in large clinical studies (LATTE-2, ATLAS, FLAIR, ATLAS-2M), the risk of virologic failure is unknown. Doses of the CAB LA and RPV LA have been selected to achieve exposures that are expected to maintain virologic efficacy on	• HIV-1 RNA viral loads will be closely monitored throughout the injection period of the study.

	the basis of available data with the oral formulations.<br>Due to administration error, it is possible that a participant could receive an inadequate dose of CAB LA or RPV LA. Sub-therapeutic concentrations of either CAB LA or RPV LA could lead to virologic failure and possibly the development of resistance.	

2.3.3.1. Other Clinically Relevant Information

Additional details concerning safety observations from clinical studies and for which a causal association has not been established or which are of minimal clinical significance may be found in the RPV and CAB IBs.

2.3.4. Significance of Anti-HBc Positivity Among Screen Failures

The WHO recommends testing for HBsAg for all newly diagnosed HIV cases, and immediate initiation of treatment with TDF, 3TC or FTC, regardless of stage of disease or HBV DNA level (WHO 2021). Discontinuation of TDF and/or 3TC may be associated with HBV reactivation, hepatic flares and in rare cases hepatic decompensation (WHO 2021). Patients with HBV co-infection have been excluded from previous trials of CAB LA+RPV LA. In the CARES trial patients are screened for HBsAg and anti-HBc and are not enrolled if positive on either of these tests. For further details on the consequences of this exclusion, please refer Section 8.5.

2.3.5. Benefits for Study Participation

Oral RPV is an established antiviral agent against HIV-1 in treatment naive patients, with long-term durability (>96 weeks in Phase 3 and >240 weeks in Phase 2b). The antiviral activity against HIV-1 of CAB has been well-established through Phase 2a and Phase 2b studies. The Phase 3 program confirmed the safety outcomes of the Phase 2 studies; over 96 weeks, the safety data from participants switching to CAB + RPV was favorable, and no new AEs of clinical concern were observed.

Participants receiving CAB LA+RPV LA are anticipated to benefit from maintenance of virological suppression using LA agents. Participants randomized to the CAB LA+RPV LA group will receive Q8W dosing without the need to take concomitant daily oral therapy, thereby benefiting from potentially lower stigma associated with this treatment. Participants can also avoid the GI-related Aes associated with oral ARV therapy from the drug-drug interactions in the GI tract. Adherence in these participants is expected to be improved and will be directly recorded during IM injections. The reduction of drugs from the cART and the discontinuation of NRTIs, may both offer long-term safety and tolerability benefits in these participants.

2.3.6. Benefit-Risk Assessment for Study Participation

Considering the measures taken to minimize risk to participants of this study, the potential risks identified in association with CAB LA+RPV LA are justified by the anticipated benefits that may be afforded to virologically suppressed participants with HIV-1 infection.

3. OBJECTIVES AND ENDPOINTS

Objectives	Endpoints
**Primary**	
• To demonstrate the non-inferior antiviral activity of switching to IM CAB LA+RPV LA administered every 2 months compared with continuation of cART administered daily over 12 months in HIV-1 infected participants in a resource limited setting.	• Proportion of participants with a virologic response (plasma HIV-1 RNA <50 c/mL)* at Month 12
**Secondary**	
• To demonstrate the antiviral and immunologic activity of switching to IM CAB LA+RPV LA every 2 months compared to continuation of cART over 12 and 24 months of follow-up	• Proportion of participants with confirmed virologic failure (2 consecutive plasma HIV-1 RNA levels ≥200 c/mL)** at Month 12 and Month 24.<br>• Proportion of participants with non-response (plasma HIV-1 RNA ≥50 c/mL) at Month 12 and Month 24.<br>• Proportion of participants with virologic response (plasma HIV-1 RNA <50 c/mL) at Month 24.<br>• Change from Baseline in CD4+ lymphocyte count at Month 12 and Month 24.<br>• Incidence of disease progression (HIV-associated conditions, AIDS and death) through 12 and 24 months.
• To evaluate the safety and tolerability of switching to CAB LA+RPV LA every 2 months compared to continuation of cART.	• Incidence of Grade 3 and Grade 4 Aes and laboratory abnormalities over time through Month 12 and Month 24.<br>• Proportion of participants who discontinue treatment due to Aes through Month 12 and Month 24.<br>• Absolute values and changes in laboratory parameters through Month 12 and Month 24.
• To assess viral resistance in participants experiencing protocol-defined confirmed virologic failure (plasma HIV-1 RNA ≥200 c/mL).<br>• To assess the incidence of on-treatment genotypic resistance to CAB, RPV and other on-study cART up to Month 12 and 24.	• To assess and compare genotypic drug resistance in individuals with confirmed virologic failure between the CAB LA+RPV LA versus cART.
• To evaluate adherence to treatment.	• Adherence, pill count for cART and dosing visits for CAB LA+RPV LA.
**Exploratory**	
• To evaluate the effects of CAB LA+RPV LA every 2 months on BMI over time compared to continuation of cART over time.	• Change from Baseline in BMI over time including Month 12 and Month 24.
• To evaluate the effect of RPV LA + CAB LA on trunk fat compared with cART	• Change from baseline in trunk fat measured by DEXA scan at Month 12 and Month 24
• To explore the effect of patient characteristics (eg, demographic factors, baseline disease characteristics) on the virologic response/non-response and immunologic response to CAB LA+RPV LA compared to continuation of cART.	• Proportion of participants by patient subgroup(s) (eg, by age, gender, BMI, HIV-1 subtype) with virologic response (<50 c/mL) over time including Month 12, and 24.<br>• Proportion of participants by patient subgroup(s) (eg, by age, gender, BMI, race, HIV-1 subtype, baseline CD4+ cell count) with plasma HIV-1 RNA ≥50 c/mL over time including Month 12, and 24.
• Retrospective analysis of archived resistance and virological outcomes using PBMCs at baseline.	• Investigate correlation of archived resistance and HIV-1 subtype determined in baseline PBMCs and virologic outcomes at Month 12 and 24.

Objectives	Endpoints
• To evaluate the effect of CAB LA+RPV LA on well-being and health status.	• Change from baseline in EQ-5D-5L scores at Month 12 and Month 24.
• To evaluate the effect of CAB LA+RPV LA on quality of life.	• Change from baseline in MOS-HIV at Month 12 and Month 24.
• To assess participant satisfaction with the injectable intervention.	• For participants in the CAB LA+RPV LA group, perception of injection and change from baseline in HIVTSQ scores at Month 12 and Month 24.
• To assess preference for CAB LA+RPV LA compared to oral cART.	• For participants randomized to the CAB LA+RPV LA group, preference for CAB LA+RPV LA compared to oral cART regimen at Month 12 and Month 24 using a single dichotomous preference question.
• To evaluate pharmacokinetics in women who become pregnant on CAB LA+RPV LA.	• Measure antiretroviral plasma concentrations in patients on long-acting antiretrovirals who become pregnant during 1st, 2nd, 3rd trimester and postpartum.
• To evaluate MRU among participants in the study.	• In both study groups, MRU over 24 months:<br>− Rates of opportunistic infections<br>− Rates of hospitalizations.
• To determine the prevalence of HBV DNA among participants on stable cART containing NRTI who test negative for HbsAg and positive for anti-HBc at screening.	• Proportion of participants who test negative for HbsAg and positive for anti-HBc with a detectable HBV viral load at screening.
• To determine the prevalence of anti-HBs positivity among participants on stable cART containing NRTI who test negative for HbsAg and positive for anti-HBc at screening.	• Proportion of participants at screening who test positive for anti-HBs among those who test negative for HbsAg and positive for anti-HBc at screening.

Abbreviations: AE: adverse event; BMI: body mass index; AIDS: Acquired immunodeficiency syndrome; CAB: cabotegravir; cART: combination retroviral therapy; c/mL: copies/milliliter; DEXA: dual energy X-ray absorptiometry; EQ-5D-5L: EuroQol 5 dimension 5 level; HIV: Human Immunodeficiency Virus; HIVTSQ: HIV Treatment Satisfaction Questionnaire; IM: intramuscular; LA: long-acting; MOS-HIV: Medical Outcomes Study HIV Health Survey; MRU: medical resource utilization; OLI: Oral lead-in; PBMC: Peripheral blood mononuclear cells; RPV: rilpivirine; RNA: ribonucleic acid

*HIV-1 RNA levels in the study will be analyzed using the Food and Drug Administration (FDA) Snapshot Algorithm.

**The designated threshold for testing of virologic failure may change during the course of the study in response to changes in national or international treatment guidelines. The new threshold will be declared and adopted consistently for management of all subsequent participants at that site.

Analysis of primary and secondary endpoints will be carried out on the intent-to-treat (ITT) population, which includes participants who have received at least one dose of study intervention (in the OLI or Maintenance Phase) within the study. Analysis of safety will be performed on the Safety population. Refer to Section 8, Study Assessments and Procedures for evaluations related to endpoints.

HYPOTHESIS

The antiviral effect of switching to CAB LA+RPV LA is non-inferior (margin -10%) to continuation of first-line treatment with daily triple drug oral cART at Month 12 in HIV-1 infected, virologically suppressed participants.

4. STUDY DESIGN

4.1. Overall Design

CARES is a randomized, open-label, active-controlled, multicenter, interventional study in virologically suppressed (<50 c/mL) HIV-1infected adult participants to demonstrate that the antiviral effect of switching to IM CAB LA+RPV LA is non-inferior to continuation of first-line cART containing 2 NRTIs (tenofovir [TDF] plus either 3TC or FTC) plus an INI (DTG) or a NNRTI (EFV or NVP) at Month 12. This is a non-inferiority study designed to assess the antiviral activity and safety of a two-drug regimen of CAB LA+RPV LA compared with maintenance of cART.

A target of 512 participants will be enrolled in this study and randomized 1:1 to continue cART or switch to the CAB LA+RPV LA for a treatment period of 24 months.

After providing written informed consent, participants will be evaluated for eligibility during the 28-day screening period. On Day 1, participants will be randomized to either continue cART or to discontinue cART and begin therapy with CAB LA+RPV LA administered every 2 months. The randomization will be stratified according to the baseline third agent class (NNRTI or INI) received by the participants as part of their cART at screening. Baseline assessments will be performed on Day 1. Participants randomized to the CAB LA+RPV LA group will be given the option of a 4-week Oral Lead-in (OLI) Phase with oral CAB and oral RPV, or to directly receive injectable CAB LA+RPV LA. This decision to dose with or without an OLI Phase will be determined by the study participant following informed consent discussions with the investigator.

The interventions received are as follows:

cART Group: Participants will take a regimen of 2 NRTIs (tenofovir [TDF] plus either 3TC or FTC) plus an INI (DTG) or a NNRTI (EFV or NVP) single tablet or FDC regimen as per local country guidelines up to Month 24. Participants will be permitted to switch cART drugs in case of toxicity, or for treatment optimization and convenience; viral load must be tested and shown to be <1000 c/mL prior to elective treatment switch; there must be confirmed virologic rebound to ≥1000 c/mL prior to switch for treatment failure.

CAB LA+RPV LA Group: The participants who opt for the OLI Phase will receive the study intervention in 2 phases:

Oral Lead-in Phase: Starting on Day 1, participants will receive CAB 30 mg + RPV 25 mg once daily for 4 weeks to be taken at approximately the same time each day with a meal. The purpose of the OLI Phase is to determine individual safety and tolerability of the combination prior to administration of CAB LA+RPV LA.

Maintenance Phase: After the 4-week OLI Phase, participants will return for the Month 1 visit to take the last dose of oral CAB+RPV at the study-site and receive the first IM CAB LA 600 mg +RPV LA 900 mg initiation injections. The second initiation injections with CAB LA 600 mg +

RPV LA 900 mg will be administered at Month 2, and then continuation injections will be administered every 2 months thereafter.

Participants who opt for direct CAB LA+RPV LA injections (ie, without the OLI Phase) will remain on cART for 4 weeks after randomization and will receive the first initiation injections of CAB LA+RPV LA at the Month 1 visit. The second initiation injections of CAB LA+RPV LA will be administered at Month 2, followed by continuation injections of CAB LA+RPV LA every 2 months thereafter.

Starting Day 1, the total duration of the study will be 24 months. Key efficacy assessments for the primary and secondary endpoints at Month 12 and Month 24 include virologic response (plasma HIV-1 RNA <50 c/mL), virologic non-response (plasma HIV- RNA ≥50 c/mL) per the FDA snapshot algorithm, and virologic failure (confirmed plasma HIV-1 RNA ≥200 c/mL on consecutive tests) per the FDA Snapshot Algorithm. CD4+ lymphocyte count, and disease progression will also be documented at Month 12 and Month 24. Safety assessments include change from baseline in body mass index (BMI), absolute weight change, weight gain >5% and >10% from baseline, new incidence of obesity (defined as BMI >30 kg/m^2) and change from baseline in body weight (absolute and percentage change). Other safety assessments will include monitoring of AEs, physical examinations, vital sign measurements, clinical laboratory tests and pregnancy testing. Disease-related quality-of-life will be measured using the Medical Outcomes Study HIV Health Survey (MOS-HIV) and EuroQol 5-dimension 5-level (EQ-5D-5L) questionnaires. Participant acceptability to the injectable intervention and feasibility will be assessed, and medical resource utilization will also be evaluated during the study.

Any participant who receives at least a single dose of CAB LA and/or RPV LA and discontinues the CAB LA+RPV LA regimen before Month 24 for any reason must start suppressive cART within 2 months of the last CAB LA+RPV LA dose. The participants must remain in the study unless consent is withdrawn, and complete their scheduled assessments up to the Month 24 visit. Investigators must discuss the choice of the follow-up cART regimen with the lead scientist/chief investigator prior to initiating the new regimen with the participant.

An Independent Data Monitoring Committee (IDMC) will evaluate interim efficacy, tolerability and safety of CAB LA+RPV LA during the study. The committee will first meet when approximately 50% of the participants have completed the Month 4 visit, and then every 6 months till the end of the study, to perform a blinded review of the available data at that time-point. An interim analysis will also be performed once approximately 60% of participants have completed the Month 6 visit. Refer to Committees Structure in Appendix 7, Regulatory, Ethical, and Study Oversight Considerations for details.

Note: Throughout the protocol, visits and analysis timepoints expressed in months from baseline are interpreted as multiples of 4-weeks from baseline ie., Month 12 is interpreted as Week 48 and Month 24 is interpreted as Week 96 from baseline. A diagram of the study design is provided in Section 1.2, Schema.

4.2. Scientific Rationale for Study Design

Blinding, Control, Study Phase/Periods, Intervention Groups

An active control will be used as a benchmark to judge non-inferiority of CAB LA+RPV LA administered every 2 months versus cART. Randomization will be used to minimize bias in the assignment of participants to intervention groups, to increase the likelihood that known and unknown participant attributes (eg, demographic and baseline characteristics) are evenly balanced across intervention groups, and to enhance the validity of statistical comparisons across intervention groups. Given that the routes of administration of the cART group (oral) and intervention group (intramuscular) are different, it is not feasible to blind the study. Furthermore, the requirement to take daily placebo pills would negate the benefits of the infrequent treatment administration in the intervention group and invalidate important patient-centred comparisons of acceptability between the 2 groups. The primary outcome will be measured in the laboratory, blinded to treatment group.

Rationale for Optional Oral Lead-in Phase

Through the course of the clinical development program for CAB LA and RPV LA including the large Phase 3 trials of FLAIR, ATLAS and ATLAS-2M, an OLI Phase of cabotegravir (30mg) along with rilpivirine (25mg), administered daily for 4 weeks, was included in all trials to allow for an initial safety assessment before study participants were allowed to advance to the LA phase of these studies. An assessment of safety data during the OLI Phase was undertaken of the FLAIR and ATLAS studies (extension phase up to Week 124) found no major safety signals during this period. Therefore, in the extension phase of the FLAIR trial, participants on standard treatment who chose to switch to CAB LA and RPV LA were given the option to switch directly to injections, ie, without and OLI Phase. Switching directly to CAB LA+RPV LA IM injections had comparable safety and tolerability to switching with an OLI Phase. Therefore, the CARES study will allow for the OLI Phase to be optional for participants randomized to the CAB LA+RPV LA group. This decision to dose with or without an OLI Phase will be determined by the study participant following informed consent discussions with the investigator.

Medical Resource Utilization Data Collection

Treatment of HIV-1 infected virologically suppressed participants with CAB LA+RPV LA versus cART may result in lower utilization of hospital resources and health services; therefore, comparison will be done across intervention groups.

4.2.1. Study-Specific Ethical Design Considerations

Potential participants will be fully informed of the risks and requirements of the study and, during the study, participants will be given any new information that may affect their decision to continue participation. They will be told that their consent to participate in the study is voluntary and may be withdrawn at any time with no reason given and without penalty or loss of benefits to which they would otherwise be entitled. Only participants who are fully able to understand the risks, benefits, and potential AEs of the study, and provide their consent voluntarily will be enrolled.

The total blood volume to be collected is considered to be an acceptable amount of blood to be collected over this time period from the population in this study.

4.3. Justification for Dose

During the Maintenance Phase of this study, CAB LA and RPV LA will be co-administered as 2 separate IM injections at each dosing visit, every 2 months (with the 2 first LA doses separated by 1 month). In the ATLAS-2M study (CROI, 2020), Q8W (3 ml) dosing was found to be non-inferior to Q4W (2 ml) dosing at the 12-month mark for both of the key virologic outcomes: viral suppression (94.3% vs 93.5%) and viral load ≥50 c/mL (1.7% and 1.0%). The safety profiles of both dosing regimens were similar, with only mild or moderate ISR reported. Of the ATLAS-2M participants who were treated with the Q8W regimen and had previously received >12 months of Q4W treatment in ATLAS, 93% (115/124) reported a preference for Q8W dosing (CROI, 2020). The ATLAS-2M results support the therapeutic potential of every 2 months CAB LA+RPV LA.

Participants in the cART group will follow the WHO recommended regimen of 2 NRTIs (TDF 300 mg/[3TC 300 mg/FTC 200 mg]) + DTG (50 mg)/EFV (600 mg)/NVP (200 mg) single tablet or FDC regimen as per local country guidelines, with appropriate treatment switches allowed to optimize tolerability, minimize toxicity or optimize viral load suppression in the event of treatment failure.

4.4. End of Study Definition

End of Study Definition

The end of study is considered as the last visit for the last participant in the study. The final data from the study-site will be sent to the sponsor (or designee) after completion of the final participant visit at that study-site, in the time frame specified in the Clinical Trial Agreement.

Participants who prematurely discontinue study intervention for any reason before study completion can be considered to have completed the study if they have completed all the scheduled assessments.

5. STUDY POPULATION

Screening for eligible participants will be performed, and the baseline visit (Day 1) will occur within 28 days of the screening visit. Refer to Section 5.4, Screen Failures for conditions under which the repeat of any screening procedures are allowed.

The inclusion and exclusion criteria for enrolling participants in this study are described below. If there is a question about these criteria, the investigator must consult with the appropriate sponsor representative and resolve any issues before enrolling a participant in the study. Waivers are not allowed.

For a discussion of the statistical considerations of participant selection, refer to Section 9.2, Sample Size Determination.

5.1. Inclusion Criteria

Each potential participant must satisfy all of the following criteria to be enrolled in the study:

1. Criterion modified per Amendment 1

1.1. Male or female as determined at birth.

2. Aged ≥18 years.

3. On a stable ARV regimen (TDF plus either 3TC or FTC, plus an INI [DTG] or a NNRTI [EFV or NVP] per local country guidelines) for at least 6 months prior to screening.

4. Virologically suppressed with documented evidence of:

   – Viral load <50 c/mL at screening AND
   – Viral load <50 c/mL on a test preceding screening (within 4-12 months prior to screening) AND
   – No two consecutive viral load tests ≥50 c/mL in the 12 months prior to screening

5. Criterion modified per Amendment 1.

5.1. No prior history of virologic failure (defined as two consecutive plasma HIV-1 RNA measurements >200 c/mL at any time).

6. Must sign an ICF indicating that he or she understands the purpose of, and procedures required for, the study and is willing to participate in the study.

7. Criterion modified per Amendment 1.

7.1. A female participant is eligible to participate if she is not pregnant (as confirmed by a negative human chorionic gonadotrophin [hCG] urine test at screening and a negative urine hCG test at randomization), not lactating, and at least one of the following conditions applies:

   a. ***Non-reproductive potential*** defined as:

   – Pre-menopausal females with one of the following:
   – History of tubal ligation
   – History of hysteroscopic tubal occlusion procedure with follow-up confirmation of bilateral tubal occlusion
   – History of hysterectomy
   – History of Bilateral Oophorectomy

- Postmenopausal defined as 12 months of spontaneous amenorrhea [in questionable cases a blood sample with simultaneous follicle stimulating hormone (FSH) and estradiol levels consistent with menopause (refer to laboratory reference ranges for confirmatory levels)]. Females on hormone replacement therapy and whose menopausal status is in doubt will be required to use one of the highly effective contraception methods if they wish to continue their hormone replacement therapy (HRT) during the study. Otherwise, they must discontinue HRT to allow confirmation of postmenopausal status prior to study enrollment.

b. ***Reproductive potential*** and agrees to follow one of the options listed in the Modified List of Highly Effective Methods for Avoiding Pregnancy in Females of Reproductive Potential from 30 days prior to the first dose of study medication, and for at least 30 days after discontinuation of all oral study medications and for at least 52 weeks after discontinuation of CAB LA and RPV LA.

The investigator is responsible for ensuring that participants understand how to properly use these methods of contraception.

8. A male participant must wear a condom when engaging in any activity that allows for passage of ejaculate to another. Male participants should also be advised of the benefit for a female partner to use a highly effective method of contraception as condom may break or leak.

9. Male participant must agree not to donate sperm for the purpose of reproduction during the study and for a minimum of 90 days after receiving the last dose of study intervention.

10. Participants with asymptomatic chronic hepatitis C virus (HCV) infection will be allowed entry if liver enzymes meet entry criteria, have undergone appropriate work-up, and are not in an advanced, unstable liver disease stage (as defined by any of the following: presence of ascites, encephalopathy, coagulopathy, hypoalbuminemia, esophageal or gastric varices, or persistent jaundice or cirrhosis, known biliary abnormalities [with the exception of Gilbert's syndrome or asymptomatic gallstones or otherwise stable chronic liver disease per investigator assessment]).

11. Is willing and able to adhere to the lifestyle restrictions specified in this protocol.

5.2. Exclusion Criteria

Any potential participant who meets any of the following criteria will be excluded from participating in the study:

1. Is currently participating in any other interventional study.

2. Is pregnant, breastfeeding or intends to become pregnant or breastfeed during the study, within the next 1 year.

3. Has evidence of an active Center for Disease Control and Prevention (CDC) Stage 3 disease, except cutaneous Kaposi's sarcoma not requiring systemic therapy and historical or current CD4 cell counts less than 200 cells/mm^3

4. Has active TB co-infection and requires anti-TB treatment.

5. Has severe hepatic impairment, history of liver cirrhosis with or without hepatitis viral co-infection.

6. Has preexisting physical or mental condition (including substance abuse disorder and suicide risk) which, in the opinion of the Investigator, may interfere with the participant's ability to comply with the dosing schedule and/or protocol evaluations or which may compromise the safety of the participant.

7. Criterion changed per amendment 1.

7.1. Either:

   – Has had one or more seizures within one year (defined as within 365 days) prior to entry, or unstable or poorly controlled seizure disorder, as determined by the investigator or designee, based on available medical records.

   Or

   – Participants determined by the Investigator to have a high risk of seizures, including participants with an unstable or poorly controlled seizure disorder. A participant with a prior history of seizure may be considered for enrolment if the Investigator believes the risk of seizure recurrence is low.

8. Has a tattoo or other dermatological condition overlying the gluteus region.

9. Is positive for HBsAg or anti-HBc at screening.

10. Has ongoing or clinically significant medical conditions that in the opinion of the investigator may interfere with the absorption, distribution, metabolism or excretion of the study interventions or could affect participant safety.

11. Has a history of coagulopathies, or current or anticipated need for chronic anticoagulation except for low dose acetylsalicylic acid (daily dose ≤325 mg).

12. Has known major INI or NNRTI resistance-associated mutation as defined in the International Antiviral Society-USA 2019 list, except for K103N, based on any historical resistance test result.

13. Has any verified Grade 4 laboratory abnormality. A single repeat test is allowed during the Screening Phase to verify a result.

14. Has estimated creatinine clearance <50mL/min per 1.73m^2 via CKD-EPI Method.

15. Has Alanine aminotransferase (ALT) ≥3 × ULN.

16. Has been exposed to any experimental drug or experimental vaccine within either 30 days, 5 half-lives of the test agent, or twice the duration of the biological effect of the test agent, whichever is longer, prior to Day 1 of this study.

17. Has been treated with any of the following agents within 28 days of Screening:

    - radiation therapy;
    - cytotoxic chemotherapeutic agents;
    - tuberculosis therapy with the exception of isoniazid (isonicotinylhydrazid, INH);
    - anticoagulation agents;
    - immunomodulators that alter immune responses (such as chronic systemic corticosteroids, interleukins, or interferons). Note: Participants using short-term steroid tapers, topical, inhaled and intranasal corticosteroids, topical imiquimod are eligible for enrollment.

18. Treatment with an HIV-1 immunotherapeutic vaccine within 90 days of Screening.

19. With the exception of the drugs allowed for the cART standard regimens, has been treated with any agent with documented activity against HIV-1 within 28 days of randomization, including but not limited to:

    - Current or prior history of etravirine (ETR) use
    - Current use of tipranavir/ritonavir or fosamprenavir/ritonavir

20. Has received any prohibited medication listed in Section 6.8.2 and who are unwilling or unable to switch to an alternate medication. Note: Any prohibited medications listed in Section 6.8.2 that decrease CAB or RPV concentrations should be discontinued for a minimum of 4 weeks or a minimum of three half-lives (whichever is longer) prior to the first dose and any other prohibited medications should be discontinued for a minimum of 2 weeks or a minimum of three half-lives (whichever is longer) prior to the first dose (see Section 6.8.2 for details of prohibited medications).

21. During the 6 weeks prior to baseline, have had ANY of (a) confirmed SARS-CoV-2 (COVID-19) infection (test positive), OR (b) suspected SARS-CoV-2 infection (clinical features without documented test results), OR (c) close contact with a person with known or suspected SARS-CoV-2 infection

    – Exception: if recommended by local guidelines and as part of SOC in the respective country, patients may be included with a documented negative result for a validated SARS-CoV-2 test (direct detection methods)

      (i) obtained at least 2 weeks after conditions (a), (b), (c) above (timed from resolution of key clinical features if present, eg, fever, cough, dyspnea)

AND

(ii) with absence of ALL conditions (a), (b), (c) above during the period between the negative test result and the baseline study visit

Precaution: for those who may carry a higher risk for severe COVID-19 illness (eg, those aged over 65 years), follow guidance from local health authorities when weighing the potential benefits and risks of enrolling in the study, and during participation in the study.

NOTE: Investigators should ensure that all study enrollment criteria have been met at screening. If a participant's clinical status changes (including any available laboratory results or receipt of additional medical records) after screening but before the first dose of study intervention is given such that he or she no longer meets all eligibility criteria, then the participant should be excluded from participation in the study. Section 5.4, Screen Failures, describes options for re-testing. The required source documentation to support meeting the enrollment criteria are noted in Appendix 7, Regulatory, Ethical, and Study Oversight Considerations.

5.3. Lifestyle Considerations

Potential participants must be willing and able to adhere to the following lifestyle restrictions during the course of the study to be eligible for participation:

1. Refer to Section 6.8, Concomitant Therapy for details regarding prohibited and restricted therapy during the study.

2. Agree to follow all requirements that must be met during the study as noted in the Inclusion and Exclusion Criteria (eg, contraceptive requirements).

5.4. Screen Failures

Screen failures are defined as participants who provide written informed consent to participate in the clinical trial, but are never subsequently enrolled or randomized. Participants will complete a screening period of up to 35 days. A single repeat of a procedure/lab parameter is allowed to determine eligibility (unless otherwise specified). Participants may be re-screened once which requires a new participant number. Participants who are randomized into the trial and subsequently withdrawn from the study, for any reason, may not be re-screened. Participants may be randomized as soon as all eligibility requirements have been confirmed at the site.

Screen failures who tested negative for HBsAg but positive for anti-HBc, and did not have a sample stored at screening, will be invited for a repeat blood sampling for the testing of HBV DNA and anti-HBs. Informed consent will be obtained from these participants for the repeat blood sampling.

Participant Identification, Enrollment, and Screening Logs

The investigator agrees to complete a participant identification and enrollment log to permit easy identification of each participant during and after the study. This document will be reviewed by the sponsor study-site contact for completeness.

The participant identification and enrollment log will be treated as confidential and will be filed by the investigator in the study file. To ensure participant confidentiality, no copy will be made. All reports and communications relating to the study will identify participants by participant identification and age at initial informed consent. In cases where the participant is not randomized into the study the date seen and age at initial informed consent will be used.

6. STUDY INTERVENTION AND CONCOMITANT THERAPY

6.1. Study Interventions Administered

Study intervention administration must be captured in the source documents and the case report form (CRF). Study-site personnel will instruct participants on how to store study intervention for at-home use as indicated for this protocol.

Oral rilpivirine and rilpivirine LA, and oral cabotegravir and cabotegravir LA, will be supplied by Janssen Pharmaceuticals and GlaxoSmithKline/ViiV Healthcare, respectively. Refer to the IBs of the respective interventions for a list of excipients.

Patients will be randomized to receive study intervention as detailed below. Treatment will be open-label, and drugs will be dispensed at intervals specified in the Schedule of Activities from a dedicated supply of study interventions that will be stored separately from routine clinic drug supplies in a designated section of the pharmacy at the study sites.

For a definition of study intervention overdose, refer to Section 6.7, Treatment of Overdose.

Description of Interventions

Oral Combined Antiretroviral Therapy

Participants randomized to the cART group will continue the standard-of-care therapy received prior to screening, with appropriate treatment switches allowed to optimize tolerability, minimize toxicity or optimize viral load suppression in the event of treatment failure.

Participants randomized to the CAB LA+RPV LA group will receive the following interventions:

Oral Lead-in Phase: CAB + RPV Tablets

CAB is manufactured by GlaxoSmithKline and is formulated as white to almost white oval shaped film-coated 30 mg tablets for oral administration, packaged in high density polyethylene bottles with desiccant and child-resistant closure that include an induction seal. CAB tablets will be packaged in bottles of 30 tablets.

RPV [Edurant®] is provided by Janssen Research & Development, LLC, a division of Janssen Pharmaceuticals as off-white, round, biconvex, film-coated 25 mg tablets. RPV will be labeled and packaged in bottles of 30 tablets.

Maintenance Phase: CAB LA+RPV LA IM Injections

Cabotegravir LA is manufactured by GlaxoSmithKline and is a sterile white to slightly pink suspension containing 200 mg/mL of CAB as free acid for administration by IM injection. The product is packaged in a 3 mL USP Type I glass vial with a 13 mm stopper and aluminum seal. Each vial is for single dose use containing a withdrawable volume of 3.0 mL, and does not require dilution prior to administration.

RPV LA 300 mg/mL Extended Release Suspension for Injection, is provided by Janssen Research & Development, LLC, a division of Janssen Pharmaceuticals, as a sterile white suspension containing 300 mg/mL of RPV as the free base. The route of administration is by IM injection. RPV LA is packaged in a single use 4 mL USP Type I glass vial with a 13 mm stopper and aluminum seal. Each vial contains a nominal fill of 3.0 mL, and does not require dilution prior to administration.

All injections must be given intramuscularly in the gluteus muscle. If possible, injections should be spaced approximately 2 cm from one another, from the site of any previous injection or any injection site reaction. IM injections should be administered at a 90-degree angle into the gluteus muscle using a needle of appropriate gauge and length (In most participants, a 1.5” 23-gauge needle for CAB LA and a 1.5” 23-gauge needle for RPV LA is recommended). The needle should be long enough to reach the muscle mass and prevent study drug from seeping into subcutaneous tissue, but not so long as to involve underlying nerves, blood vessels, or bone. Variable needle lengths and/or needles with different gauge (CAB LA: 21 to 25 gauge; RPV LA: 21 to 23 gauge) are permitted if needed to accommodate individual body types. Longer needle lengths may be required for participants with higher BMIs, example >30), to ensure that injections are administered intramuscularly as opposed to subcutaneously. The time and location of injection will be captured in the eCRF. BMI, needle gauge and length used will also be collected in the eCRF.

6.2. Preparation/Handling/Storage/Accountability

Preparation/Handling/Storage

CAB tablets are to be stored up to 30°C [86°F] and protected from moisture. RPV tablets should be stored at 25°C (excursions permitted to 15°-30°C [59°-86°F]).

CAB LA injectable suspension is to be stored at up to 30°C, do not freeze. RPV LA injectable suspension should be kept in the outer package and stored at 2-8°C (do not freeze). RPV should also be protected from light.

Refer to the CAB and RPV IBs for additional guidance on study intervention preparation, handling, and storage.

Accountability

The investigator is responsible for ensuring that all study intervention received at the site is inventoried and accounted for throughout the study. The dispensing of study intervention during the OLI Phase to the participant, and the return of study intervention from the participant (if applicable), must be documented on the intervention accountability form. Participants must be instructed to return all original containers, whether empty or containing study intervention. The study intervention administered to the participant during the Maintenance Phase must be documented on the intervention accountability form. All study intervention will be stored and disposed of according to the sponsor's instructions. Study-site personnel must not combine contents of the study intervention containers.

Study intervention must be handled in strict accordance with the protocol and the container label, and must be stored at the study-site in a limited-access area or in a locked cabinet under appropriate environmental conditions. Unused study intervention, and study intervention returned by the participant, must be available for verification by the sponsor's study-site monitor during on-site monitoring visits. The return to the sponsor of unused study intervention, or used returned study intervention for destruction, will be documented on the intervention return form. When the study-site is an authorized destruction unit and study intervention supplies are destroyed on-site, this must also be documented on the intervention return form.

Potentially hazardous materials containing hazardous liquids, such as used ampules, needles, syringes and vials, should be disposed of immediately in a safe manner and therefore will not be retained for intervention accountability purposes.

Study intervention should be dispensed under the supervision of the investigator or a qualified member of the study-site personnel, or by a hospital/clinic pharmacist. Study intervention will be supplied only to participants participating in the study. Returned study intervention must not be dispensed again, even to the same participant. Study intervention may not be relabeled or reassigned for use by other participants. The investigator agrees neither to dispense the study intervention from, nor store it at, any site other than the study sites agreed upon with the sponsor.

6.3. Measures to Minimize Bias: Randomization and Blinding

Intervention Allocation

Procedures for Randomization and Stratification

Participants will be randomly assigned to 1 of 2 intervention groups based on a computer-generated randomization schedule prepared before the study by or under the supervision of the sponsor. The randomization will be balanced by using randomly permuted blocks and will be stratified according to the third agent class (NNRTI or INI) at screening. Based on this randomization code, the study intervention will be packaged and labeled for each participant. Participant numbers will be preprinted on the study intervention labels and assigned as participants qualify for the study and are assigned to intervention.

Blinding

As this is an open study, blinding procedures are not applicable.

6.4. Study Intervention Compliance

When participants self-administer oral study treatments at home, compliance will be assessed through querying the participant during the site visits and documented in the source documents and CRF. Study intervention accountability will be evaluated using pill counts of unused CAB and RPV tablets. This assessment will be conducted each time the participant receives a new (refill) supply of oral study medication or completes the OLI Phase. A record of the number of CAB and RPV tablets dispensed to and taken by each participant must be maintained and reconciled with study treatment and compliance records. Treatment start and stop dates, including dates for treatment delays and/or dose reductions will also be recorded in the CRF.

When participants are dosed at the site, they will receive study treatment directly from the investigator or designee, under medical supervision. The date and time of each dose administered in the clinic will be recorded in the participant's eCRF. The dose of study treatment and study participant identification will be confirmed at the time of dosing by a member of the study-site staff other than the person administering the study treatment.

Due to the long-acting nature of the CAB LA and RPV LA it will be imperative that the participant is compliant with the injection visits. Investigators must have plans in place for adherence counseling for both treatment groups of the study. In addition, Investigators must have plans in place to perform visit reminders and to verify the participant's contact information at each visit.

6.5. Dose Modification

Participants will be permitted to switch cART drugs in case of toxicity, or for treatment optimization and convenience; viral load must be tested and shown to be <1000 c/mL prior to elective treatment switch; there must be confirmed virologic rebound to ≥1000 c/mL prior to switch for treatment failure.

Under exceptional circumstances, to address pre-planned missed CAB LA+RPV LA dosing visits, the investigator after authorization by the lead scientist/chief investigator may permit the use of daily oral CAB 30 mg and RPV 25mg as a short-term "bridging" strategy (≤4 weeks duration) for participants who have begun CAB LA+RPV LA. Should a participant need "oral bridging", sites must contact the lead scientist/chief investigator for authorization and guidance for treatment strategies prior to a missed CAB LA+RPV LA dose. Should a participant not notify the site in advance, the lead scientist/chief investigator must be contacted for further treatment guidance.

6.6. Continued Access to Study Intervention After the End of the Study

At the end of their participation in the study, participants from the CAB LA + RPV LA group who have completed the study and continue to benefit clinically from the study intervention, as determined by their investigator and are willing to continue treatment, will receive continued access to CAB LA and RPV LA for up to 4 years after study completion. Participants will continue to receive study intervention until:

- the participant no longer derives clinical benefit;
- the participant meets a protocol-defined reason for discontinuation;
- CAB LA and RPV LA are registered and reimbursed in the country; or
- either CAB LA or RPV LA development program is terminated; whichever occurs earlier.

This continued access will be provided through a post-trial access (PTA) program. If this program is not operational at the end of the study, participants may continue to receive CAB LA and RPV LA via the study in order to avoid treatment interruption. The continued access to CAB LA and RPV LA within the study will serve as a bridge to the operationalization of the post-trial access program. During this bridging, participants will continue to receive care at respective study sites.

Under the PTA program, Janssen will continue to provide access to CAB LA and RPV LA until patients can obtain the study intervention outside the post-trial access program or through commercial access within local healthcare systems per local/national regulations. Post-trial access will continue for up to 4 years, after which an evaluation of available local access options will be performed and access determined on a case-by-case basis, based on patient needs and available access options. For the purpose of this study, 'commercial access' means after approval by the competent national authorities for reimbursement under the national health insurance or comparable third-party payment programs.

Participants in the cART group will continue to receive cART medications as part of the national program.

6.7. Treatment of Overdose

For participants receiving oral CAB, any tablet intake exceeding a total daily dose of 30 mg will be considered an overdose. For participants receiving oral RPV, any dose exceeding a total daily dose of 25 mg will be considered an overdose.

For CAB LA and RPV LA, any single dose in excess of the studied doses will be considered an overdose.

Should IM maladministration, specifically overdose or inadvertent IV dosing, be suspected at any time, the participant will stay on-site for approximately 2-3 hours post dose for safety monitoring and an ECG will be performed at 2 hours post dose. The coordinating center will be notified in the event of a suspected maladministration. In the event of suspected maladministration, additional PK samples may be drawn as soon as possible at the time of the event for evaluation of CAB and RPV concentrations.

For the purposes of this study, an overdose is not an AE (refer to Appendix 8) unless it is accompanied by a clinical manifestation associated with the overdose. If the clinical manifestation presents with serious criteria, the event is a serious adverse event (see Appendix 8). If an overdose occurs and is associated with an adverse event requiring action, all study medications must be temporarily discontinued until the adverse event resolves.

In the event of an overdose, the investigator or treating physician should:

- Contact the lead scientist/chief investigator.
- Evaluate the participant to determine, in consultation with the lead scientist/chief investigator, whether study intervention should be interrupted.
- Closely monitor the participant for AE/SAE and laboratory abnormalities CAB LA+RPV LA.
- Document the quantity of the excess dose as well as the duration of the overdosing in the CRF.

6.8. Concomitant Therapy

All prior antiretroviral therapies received must be recorded at screening.

Non-antiretroviral concomitant therapies must be recorded throughout the study beginning with start of the first dose of study intervention to 30 days after the last dose of study intervention. Concomitant therapies should also be recorded beyond 30 days only in conjunction with new or worsening AEs or serious AEs that meet the criteria outlined in Serious Adverse Events in Section 8.3.1, Time Period and Frequency for Collecting Adverse Event and Serious Adverse Event Information.

All therapies (prescription or over-the-counter medications, including vaccines, vitamins, herbal supplements) different from the study intervention must be recorded in the CRF. Recorded information will include a description of the type of therapy, duration of use, dosing regimen, route of administration, and indication. Modification of an effective preexisting therapy should not be made for the explicit purpose of entering a participant into the study.

6.8.1. Permitted Medications and Non-drug Therapies

Chemoprophylaxis for HIV-associated conditions is encouraged, if appropriate, at the discretion of the participant and their physician. All concomitant medications, blood products, and vaccines taken during the study will be recorded in the eCRF with dates of administration.

Because non-HIV vaccines may cause a temporary increase in the level of plasma HIV-1 RNA, it is recommended that a vaccine, if necessary, be given during or immediately after a scheduled visit after all laboratory tests have been drawn. This approach will minimize the risk of non-specific increases in the level of plasma HIV-1 RNA at the next scheduled assessment.

Other IM injectables (with exceptions below) are permitted but must be administered away from the site of IM administration (should be spaced 2 cm or more away from site of IM injection).

CAB oral administration: Products containing divalent cations (eg, aluminum, calcium and magnesium) must be taken at least 2 hours before or at least 4 hours after CAB.

Concurrent administration of multivitamins is acceptable.

RPV oral administration: Antacid products must be taken at least 2 hours before or at least 4 hours after RPV. H2-Receptor antagonists (eg, cimetidine, famotidine, nizatidine, ranitidine) may

cause significant decreases in RPV plasma concentrations. H2-receptor antagonists should only be administered at least 12 hours before or at least 4 hours after RPV. RPV should not be co-administered with proton pump inhibitors such as esomeprazole, lansoprazole, omeprazole, pantoprazole, rabeprazole.

Administration of clarithromycin, erythromycin and telithromycin is not recommended with RPV due to possible increase in plasma concentration of RPV due to CYP3A enzyme inhibition. Where possible, alternatives such as azithromycin should be considered.

6.8.2. Prohibited Medications and Non-drug Therapies

The following concomitant medications or therapies are not permitted at any time during the study:

- HIV immunotherapeutic vaccines are not permitted at any time during the study.
- Other experimental agents, antiretroviral drugs not otherwise specified in the protocol, cytotoxic chemotherapy, or radiation therapy may not be administered.
- Systemically administered immunomodulators (such as interleukin and interferon agents) are prohibited. This includes topical agents with substantial systemic exposure and systemic effects. Short-term use (30 days or less) of topical imiquimod is permitted.
- Acetaminophen (paracetamol) cannot be used in patients with acute viral hepatitis.
- Chronic use of systemic (oral or parenteral) glucocorticoids must be avoided due to immunosuppressive effect and potential decreases in RPV plasma concentrations; however, short treatment courses (eg, ≤14 days) of oral prednisone/ prednisolone/methylprednisolone are allowed. A single dose of systemic dexamethasone is permitted (more than a single dose may cause significant decrease in RPV plasma concentration and is prohibited), and topical, inhaled or intranasal use of glucocorticoids will be allowed.
- Hepatitis C infection therapy is prohibited during the Maintenance Phase before the Month 12 primary endpoint, and interferon-based HCV therapy or use of any drugs that have a potential for adverse drug-drug interactions with study treatment is prohibited throughout the entire study.

For information on concurrent therapies and interactions suspected to be relevant to cART, please consult the SmPC for the specific drugs.

6.8.3. Prohibited Medications With CAB and/or RPV

For participants receiving either formulation of CAB and/or RPV, the following medications could significantly decrease the levels of CAB and/or RPV due to enzyme induction and therefore must not be administered concurrently:

- Carbamazepine
- Oxcarbazepine
- Phenobarbital
- Phenytoin

- Rifabutin
- Rifampicin / Rifampin
- Rifapentine
- St. John's wort (*Hypericum perforatum*)
- Systemic dexamethasone (more than a single dose)
- Proton pump inhibitors, such as esomeprazole, lansoprazole, omeprazole, pantoprazole, rabeprazole (must not be administered during oral intake of CAB or RPV; no contraindication for injectable formulation).

If the participant cannot discontinue use or change to an allowable alternative while receiving treatment with RPV, the participant should not be randomized into the study.

In addition, for participants receiving CAB LA and RPV LA, use of anticoagulation agents for greater than 14 days is prohibited, with the exception of the use of anticoagulation for deep vein thrombosis prophylaxis (eg, postoperative DVT prophylaxis) or the use of low dose acetylsalicylic acid (≤325mg). Systemic anticoagulation (including prophylaxis doses) on the day of an IM injection should be avoided.

Note: Any prohibited medications that decrease cabotegravir or rilpivirine concentrations should be discontinued for a minimum of 4 weeks or a minimum of three half-lives (whichever is longer) prior to the first dose and any other prohibited medications should be discontinued for a minimum of 2 weeks or a minimum of three half-lives (whichever is longer) prior to the first dose.

7. DISCONTINUATION OF STUDY INTERVENTION AND PARTICIPANT DISCONTINUATION/WITHDRAWAL

7.1. Discontinuation of Study Intervention

If a participant discontinues study intervention for any reason before the end of the Maintenance Phase, then scheduled assessments off the study intervention should be continued up to the Month 24 visit, unless consent is withdrawn. Study intervention assigned to the participant who discontinued study intervention may not be assigned to another participant. Additional participants may be entered to ensure the protocol-specified number of participants complete the study.

A participant's study intervention <u>may</u> be discontinued if:

- The participant has a serious adverse event.
- The participant fails to comply with the protocol or study staff requirements.
- The participant starts treatment with 1 of the medications reported on the list of disallowed medications (see Section 6.8.2). The final decision to withdraw a participant who starts treatment with a disallowed medication will be made by the sponsor following discussions with the investigator.
- The participant develops a Grade 3 adverse event (with the exception of cutaneous events/rash or acute systemic allergic reaction which require discontinuation of study intervention) or

confirmed Grade 3 laboratory abnormality according to the DAIDS adverse event grading scale (Appendix 5).

- For the conditions listed below, study intervention should only be discontinued if the clinical assessment foresees an immediate health risk to the participant (see Section 10.4.1 for additional details).
  - The participant develops 1 of the following Grade 3 AEs or Grade 3 laboratory abnormalities according to the DAIDS adverse event grading scale (Appendix 5):
    - has a cholesterol elevation of Grade 3 under nonfasting conditions
    - has asymptomatic cholesterol elevations of Grade 3
    - has asymptomatic pancreatic amylase elevations of Grade 3 with no past or active history of pancreatitis
    - had preexisting diabetes and experiences a glucose elevation of Grade 3
    - has a glucose or triglyceride elevation of Grade 3 under non-fasted conditions
    - has asymptomatic glucose or triglyceride elevations of Grade 3
    - experiences a Grade 3 adverse event or Grade 3 laboratory abnormality that is considered not related or doubtfully related to the study intervention.
  - The participant develops 1 of the following Grade 4 AEs or Grade 4 laboratory abnormalities according to the DAIDS adverse event grading scale (Appendix 5):
    - had preexisting diabetes and experiences a glucose elevation of Grade 4
    - has a glucose or triglyceride elevation of Grade 4 under non-fasted conditions
    - has asymptomatic glucose or triglyceride elevations of Grade 4
    - experiences a Grade 4 adverse event or Grade 4 laboratory abnormality that is considered not related or doubtfully related to the study intervention.

  Note: If clinical assessment foresees an immediate health risk to the participant, the study intervention **must** be discontinued.

A participant's study treatment **must** be discontinued if:

- The investigator believes that for safety reasons or tolerability reasons (eg, adverse event) it is in the best interest of the participant to discontinue study treatment.
- Treatment failure indicated by the participant having virologic failure confirmed by 2 consecutive HIV-1 RNA levels ≥200 c/mL obtained 6 weeks (window of 4-6 weeks) apart. The participant should be discontinued from the study treatment and should then be treated as per the local treatment guideline recommendations, as for any first-line treatment failure.
- The participant develops a Grade 4 adverse event or Grade 4 confirmed laboratory abnormality according to the DAIDS adverse event grading scale (see Appendix 5).

  Note: See bullet under "a participant's study treatment may be discontinued" above and Section 10.4.1, General Guidance.
- The participant develops 1 of the following Grade 3 or 4 AEs according to the DAIDS adverse event grading scale (see Appendix 5):

  - alterations in personality behavior or in mood that are considered at least possibly related to the study intervention (see Section 10.4.2, Neuropsychological Symptoms)
  - cutaneous reaction/rash or allergic reaction (see Section 10.4.3, Cutaneous Events/Rash, and Section 10.4.4, Acute Systemic Allergic Reaction).

- The participant develops renal complications that are considered at least possibly related to the study intervention (see Section 10.4.8, Renal Complications).
- The participant develops Grade 4 study intervention-related nausea or diarrhea according to the DAIDS adverse event grading scale (see Section 10.4.9, Nausea (With or Without Vomiting), and Section 10.4.10, Diarrhea; Appendix 5)
- The participant develops clinical hepatitis after baseline.
- The participant has confirmed hyperlactatemia (>2 x ULN) with symptoms as described in Section 10.4.13, Lactic Acidosis.
- The participant develops Grade 4 elevations of pancreatic amylase according to the DAIDS adverse event grading scale (see Appendix 5) and/or the participant's Grade 3 or 4 lipase levels persist ≥14 days following interruption of all study interventions, or if the toxicity recurs more than twice (see Section 10.4.15, Lipase Elevations).
- The participant is taking CAB LA+RPV LA and requires treatment with rifampicin.
- The participant experiences a confirmed QT interval corrected for heart rate according to Fridericia (QTcF) >500 msec (a confirmatory test should be performed, preferably within 48 hours [if practically feasible] after the results have become available in case a QTcF >500 msec occurs).
- The participant 's total cumulative duration of treatment interruptions for suspected toxicities is more than 2 months or the duration of a single treatment interruption for a suspected toxicity is more than 1 month.
- A participant randomized to the CAB LA+RPV LA group is diagnosed with tuberculosis after baseline. They will be followed-up for at least 12 months after the last dose of CAB LA+RPV LA. Plasma samples will be collected from these participants 1, 3, 6 and 12 months after the last dose of CAB LA+RPV LA for measurement of levels of ART drugs. Participants in the cART group diagnosed with TB will be allowed to continue in the study and will be treated per local guidelines.

If a participant discontinues study treatment for any reason before the end of the Maintenance Phase, this will not result in automatic withdrawal of the participant from the study. The participants must remain in the study unless consent in withdrawn, and complete their scheduled assessments up to the Month 24 visit. Any participant who has received at least a single dose of CAB LA+RPV LA and discontinues the regimen before Month 24 for any reason must start suppressive cART within 2 months of the last LA dose.

7.1.1. Liver Chemistry Stopping Criteria

Discontinuation of study intervention for abnormal liver tests is required by the investigator when a participant meets one of the conditions outlined in Appendix 3 Liver Safety: Suggested Actions and Follow-Up Assessments and Study Intervention Rechallenge Guidelines, or in the presence of

abnormal liver chemistries not meeting protocol-specified stopping rules if the investigator believes that it is in best interest of the participant.

7.1.2. Temporary Discontinuation

CAB LA+RPV LA or cART may be interrupted at the discretion of the investigator in the event of an AE, according to the severity of the AE.

If one or more antiretroviral medications is held due to toxicity or AEs, all antiretroviral medications must be held to reduce the risk of development of resistance taking into account both the length of the planned interruption and the pharmacokinetic half-life of each antiretroviral of the regimen, in a way to minimize the risk of development of resistance.

Keeping to the Schedule of Activities is an important component to the study. All decisions regarding dose interruption / resumption must be discussed with the scientific lead and chief investigator in advance.

Please refer to Section 6.5 and Appendix 4 for guidance on interruption of study interventions due to AEs and toxicities.

7.1.3. Rechallenge

Please refer to Appendix 3 for guidance on the criteria for rechallenge/restart following toxicity after a liver event.

7.2. Participant Discontinuation/Withdrawal From the Study

A participant will be withdrawn from the study for any of the following reasons:

- Lost to follow-up
- Withdrawal of consent
- Death

When a participant withdraws before study completion, the reason for withdrawal is to be documented in the CRF and in the source document. If the reason for withdrawal from the study is withdrawal of consent, then no additional assessments are allowed.

Withdrawal of Consent

A participant declining to return for scheduled visits does not necessarily constitute withdrawal of consent. Alternate follow-up mechanisms that the participant agreed to when signing the consent form apply (eg, consult with family members, contacting the participant's other physicians, medical records, database searches, use of locator agencies at study completion) as local regulations permit.

7.3. Lost to Follow-up

To reduce the chances of a participant being deemed lost to follow-up, prior to randomization attempts should be made to obtain contact information from each participant, eg, home, work, and

mobile telephone numbers and email addresses for both the participant as well as appropriate family members.

A participant will be considered lost to follow-up if he or she repeatedly fails to return for scheduled visits and is unable to be contacted by the study-site. A participant cannot be deemed lost to follow-up until all reasonable efforts made by the study-site personnel to contact the participant are deemed futile. The following actions must be taken if a participant fails to return to the study-site for a required study visit:

- The study-site personnel must attempt to contact the participant to reschedule the missed visit as soon as possible, to counsel the participant on the importance of maintaining the assigned visit schedule, to ascertain whether the participant wishes to or should continue in the study.
- Before a participant is deemed lost to follow-up, the investigator or designee must make every reasonable effort to regain contact with the participant (where possible, 3 telephone calls, e-mails, fax, and, if necessary, a certified letter to the participant’s last known mailing address, or local equivalent methods). These contact attempts should be documented in the participant’s medical records.
- Should the participant continue to be unreachable, they will be considered to have withdrawn from the study.
- Site personnel, or an independent third party, will attempt to collect the vital status of the participant within legal and ethical boundaries for all participants randomized, including those who did not get study intervention. Public sources may be searched for vital status information. If vital status is determined as deceased, this will be documented and the participant will not be considered lost to follow-up. Sponsor personnel will not be involved in any attempts to collect vital status information.

Should a study-site close, eg, for operational, financial, or other reasons, and the investigator cannot reach the participant to inform them, their contact information will be transferred to another study-site.

8. STUDY ASSESSMENTS AND PROCEDURES

Overview

Participants will be randomized on Day 1 to either receive CAB LA+RPV LA or remain on cART.

Participants in the CAB LA+RPV LA group who opt for the CAB+RPV OLI regimen will start oral CAB+RPV on Day 1. At the Month 1 visit (4 weeks later), participants will take the last dose of the OLI regimen at the study-site and receive the first IM CAB LA+RPV LA initiation injections. The second IM initiation injections will be administered at Month 2, and then continuation injections will be administered every 2 months up to Month 24.

Participants who opt for direct CAB LA+RPV LA injections will remain on cART for 4 weeks after randomization, and will receive the first initiation injections of CAB LA+RPV LA at the Month 1 visit. The second initiation injections of CAB LA+RPV LA will be administered at Month 2, followed by continuation injections of CAB LA+RPV LA every 2 months thereafter.

Participants randomized to the cART group will continue the cART regimen they were taking at screening, or may switch at baseline or after to an alternative INI or NNRTI-based cART regimen, if preferred for reasons of convenience or tolerability.

Please refer to the Schedule of Activities for details on the scheduled study visits for the CAB LA+RPV LA and the cART group. Additional unscheduled visits for collection of blood for confirming viral load rebound or for evaluation of adverse events may be required in both arms. For participants in both groups, the final study visit will occur at Month 24, or at the time when the participants withdraw completely from the study, whichever occurs first.

Some of the study visits may be nurse-led visits to check on adherence, physical well-being and to identify any clinical events that might need further evaluation by a clinician. Laboratory blood tests will be performed, and plasma samples stored at selected clinician visits. Repeat or unscheduled samples may be taken for safety reasons or for technical issues with the samples. Additional nurse-led or clinician visits will be arranged as clinically indicated to follow the development and resolution of any adverse events.

Participants will be expected to attend on the scheduled day unless agreed in advance with the study-site. If they are unable to attend on the day, every effort will be made to complete the visit within the designated visit window. If a scheduled visit is missed without notice, then the study-site should endeavor to contact the patient by phone or by home visit. The site may choose to reschedule visits to allow for public holidays or other unavoidable circumstances that affect the scheduled visit date, but the rescheduled visit should be within the scheduled visit window.

All patient-reported outcomes (PRO) assessments should be conducted/completed before any tests, or other consultations to prevent influencing participant responses. Refer to the PRO completion guidelines for instructions on the administration of PROs. Actual dates and times of assessments will be recorded in the source documentation and CRF.

Please refer to the Schedule of Activities for the frequency and timing of efficacy, safety, medical resource utilization and other measurements applicable to the study. For guidance on study conduct during a pandemic, please refer to Appendix 13.

Sample Collection and Handling

The actual dates and times of sample collection will be recorded in the CRF or laboratory requisition form. Refer to the Schedule of Activities for the timing and frequency of all sample collections.

Instructions for the collection, handling, storage, and shipment of samples are found in the laboratory manual that will be provided. Collection, handling, storage, and shipment of samples must be under the specified, and where applicable, controlled temperature conditions as indicated in the laboratory manual.

8.1. Efficacy Assessments

8.1.1. Viral Load by FDA Snapshot algorithm

Participants in both groups will have routine viral load monitoring by FDA Snapshot algorithm at baseline and Months 6, 12, 18 and 24.

Definition of Virological Response for Statistical Analysis

For the purposes of statistical analysis, a participant will be considered have met the primary endpoint of virological response at Month 12 or 24 if the plasma HIV-1 RNA is <50 c/mL as defined by the FDA's Snapshot algorithm at these time-points.

Definition of Virological Failure for Statistical Analysis

For the purposes of statistical analysis, a participant will be considered have met the secondary endpoint of virological failure if the plasma HIV-1 RNA is ≥ 200 c/mL on two consecutive samples taken 6 weeks apart (window of 4-6 weeks) during the trial follow-up.

Definition of Virological Failure for Clinical Management

For the purposes of clinical management in the CAB LA+RPV LA group, the participant will be considered to have confirmed virological failure if the plasma HIV-1 RNA is ≥200 c/mL on two consecutive samples taken 6 weeks apart (window of 4-6 weeks).

For the purposes of clinical management in the cART arm, the participant will be considered have confirmed virological failure if the plasma HIV-1 RNA is ≥1000 c/mL on 2 consecutive samples (but see section on alternative threshold, below).

8.1.1.1. Repeat HIV-1 RNA Testing

CAB LA+RPV LA Group

Participants with HIV-1 RNA level ≥200 c/mL at any of the scheduled time-points will have a blood sample collected 4-6 weeks after the date of the first sample for confirmation of virologic rebound.

If the participant has recently interrupted treatment with oral CAB+RPV (if on bridging treatment) or if there is an interval of >10 weeks from the time of the last dose of CAB LA+RPV LA, then the repeat test should be performed at least 2 weeks after resuming full dose of oral treatment or at least 2 weeks after the administration of the next dose of CAB LA+RPV LA.

The presence of any other conditions (apart from treatment interruption) that may lead to a transient increase in the HIV-1 RNA level at the time of the repeat (eg, immunization, intercurrent illness, non-adherence) will be recorded on the CRFs but the repeat test will not be delayed.

Interpretation / actions

- *Initial test ≥200 c/mL and repeat test ≥200 c/mL:* This meets the definition of confirmed virological failure for clinical management purposes (see above). Manage as for confirmed virological failure (see below).
- *Initial test ≥ 200 c/mL and repeat test < 200 c/mL:* This does not meet the definition of confirmed virologic failure for clinical management purposes. The participant will be tested at the next study visit where VL testing is scheduled by the protocol (ie, at Month 12, 18 or 24).

cART Group

Participants with HIV-1 RNA level ≥200 c/mL at any of the scheduled viral load testing time-points will have a blood sample collected 4-6 weeks after the date of the first sample for plasma storage (but not for real-time viral load testing; see below). Participants for whom HIV-1 RNA level is ≥1000 c/mL at the scheduled viral load test time-point will, in addition to the plasma storage sample at 4-6 weeks, have an additional sample taken 12 weeks (window of 10-16 weeks) after the date of the first sample for real-time testing to confirm virologic rebound.

If the participant has recently interrupted treatment with oral cART, then the sample at 4-6 weeks and the additional sample at 12 weeks (window of 10-16 weeks) should be collected at least 2 weeks after resuming the full dose of oral treatment.

The presence of any other conditions (apart from treatment interruption) that may lead to a transient increase in the viral load level at the time of the repeat sample(s) (eg, immunization, intercurrent illness, non-adherence) will be recorded on the CRFs but the collection of the repeat test will not be delayed.

The sample collected for plasma storage at 4-6 weeks from the time of the first sample is for research purposes only. This sample will be stored for batched viral load testing that will be performed prior to key study analyses. The results will be blinded and sent to the trial statistician;

they will only be returned to the managing clinician at or after the time when the patient exits the trial. The reason for requiring this early viral load test is to allow a strictly comparable efficacy analysis of the incidence of viral load rebound between the 2 randomized arms (despite the fact that the clinical management strategies differ between the 2 arms). The reason for blinding the viral load test result is that a test performed after 4-6 weeks is not consistent with routine clinical practice in most program settings, which recommend repeating after a longer interval in order to allow for adequate adherence counseling to be performed, and for any resulting change in adherence to have maximum impact on virological suppression prior to re-testing. This deviation could impact clinical management decisions and thereby affect the generalizability of the trial results to program settings. Blinding the test is a more reliable and effective way of preserving (and demonstrating) generalizability than performing the test in real-time and revealing the result to a clinician (and patient) while telling them to ignore the information it provides (which is especially unrealistic during a period of adherence counseling following virological rebound).

The sample collected at 12 weeks (window of 10-16 weeks) from the time of the first sample (only done if the HIV-1 RNA level is ≥1000 c/mL in the first sample), is performed for the definitive confirmation of virological failure for clinical management.

Additional adherence counseling will be given to all participants following any test that shows a viral load result viral load ≥50 c/mL. Intensive adherence counseling will be provided to all participants following any result ≥1000 c/mL following standard practice at the site.

Interpretation /actions

- *Initial test ≥1000 c/mL and repeat test ≥1000 c/mL:* This meets the definition of confirmed virological failure for clinical management purposes (see above), therefore manage as for confirmed virological failure. However, if there are underlying causes for confirmed virological failure that are, in the opinion of the managing clinician, potentially reversible or transient (such as intercurrent illness, recent immunization, ongoing poor adherence), then the clinician may elect to defer changing treatment and perform a further viral load test 12 weeks (window 10-16 weeks) later (following further adherence counseling or other intervention as considered appropriate for the underlying cause). Further testing and clinical management following this repeat viral load test would follow the above approach (if <1000 c/mL then the next test will be done at the time of the next scheduled viral load test; if ≥1000 c/mL then changing cART should be strongly considered).
- *Initial test ≥1000 c/mL and repeat test <1000 c/mL:* This does not meet the definition of confirmed VL failure for clinical management purposes. The participant will be tested at the next study visit where VL testing is scheduled by the protocol (ie, 12, 18 or 24 months).

8.1.1.2. Alternative Threshold for Testing and Definition of Virologic Failure in the cART Group

The threshold for triggering repeat testing and defining virological failure for clinical management purposes in the cART arm is set at ≥1000 c/mL for this protocol, following WHO guidelines and standard practice in program settings at the time this protocol was finalized. However, if the routine practice at the clinical site is that a threshold below ≥1000 c/mL is used as the determinant for repeat viral load testing or treatment switch then the site may opt to use the ≥200 c/mL threshold

for repeat testing and for treatment switch during this trial. The threshold of choice (≥1000 c/mL or ≥200 c/mL) must be formally declared to the sponsor prior to the start of the study and be followed systematically for all patients in the trial at that site. If the threshold starts at ≥1000 c/mL but national or international guidelines change or site practice changes during the trial, the threshold may be reduced to ≥200 c/mL during the course of the trial, provided that this is formally declared to the trial sponsor and the same threshold is prospectively applied to all patients in the cART group at the site from that point onwards.

8.1.1.3. Additional samples

Two additional plasma samples will be drawn at the time of each confirmatory viral load sample in both groups: one saved for real-time clinical genotypic resistance testing to be performed if virological rebound is confirmed; one saved for future research testing (which may include genotypic or phenotypic resistance testing, drug levels, or other tests).

8.1.1.4. Management of Confirmed Virological Failure

Participants in the LA group who have confirmed virological failure must have study intervention discontinued and be switched to oral cART selected by the managing clinician, according to local SOC and considering information from available genotypic resistance testing. Participants will remain in the study until the completion of the Month 24 visit and will then transition to standard program care. Following change of treatment, viral load testing will continue following the standard schedule of viral load tests in the protocol.

Participants in the cART group who have confirmed virological failure may switch to a new cART regimen at clinician discretion and following local treatment guidelines (and considering information from available genotypic resistance testing). They will remain in the study until the completion of the Month 24 visit and will then transition to standard program care. Viral load measurement will continue following the standard schedule of VL tests in the protocol.

8.1.2. Genotyping

At baseline, PBMC samples will be collected to retrospectively assess preexisting resistance and HIV-1 subtype.

At the time of a repeat evaluation for confirmation of virologic failure, 2 additional plasma sample will be collected. If virologic failure is confirmed, the first sample will be tested at a central laboratory for genotypic drug resistance. The second sample will be saved for future resistance testing, genotypic or phenotypic, for research purposes.

8.1.3. Immunology

Blood samples will be collected at the visits indicated in the Schedule of Activities to determine CD4+ T-cell count by flow cytometry (total lymphocyte counts, percentage and absolute CD4+ lymphocyte counts, ratios) according to Schedule of Activities (Section 1.3) and Laboratory Assessments.

8.1.4. Patient-Reported Outcomes

- The PRO instrument will be provided in the local language in accordance with local guidelines.
- The PRO instrument must be available for regulators and for IRB/ERC submissions, therefore the PRO instrument or screen shots need to be attached to the protocol or provided in a companion manual with the instruments that will be submitted with the protocol.
- The PRO and adverse event data will not be reconciled with one another.

8.1.4.1. EuroQoL-5D-5L Health Questionnaire

The EQ-5D-5L is a validated quality-of-life instrument to be completed by the subject (Appendix 10). The EQ-5D-5L provides a visual analogue scale to integrate many aspects of the subject's disease process into a single assessment along with targeted quality-of-life questions. It is a non-disease-specific measure of health status that provides a simple descriptive profile and a single index value that can be used in the clinical and economic evaluation of health care and in population health surveys. The EQ-5D-5L questionnaires will be completed at the times indicated in the Schedule of Activities.

8.1.4.2. Medical Outcomes Study HIV Health Survey: MOS-HIV

The MOS-HIV is a brief, comprehensive measure of health-related quality-of-life (HRQoL) used extensively in HIV/AIDS (Appendix 11) (Wu 1997). The 35-item questionnaire includes 10 dimensions (health perceptions, pain, physical, role, social and cognitive functioning, mental health, energy, health distress and quality of life and takes approximately 5 minutes to complete. Subscales are scored on a 0 100 scale (a higher score indicates better health) and physical and mental health summary scores can be generated. The MOS-HIV has been shown to be internally consistent, correlate with concurrent measures of health, discriminate between distinct groups, predict future outcomes and be responsive to changes over time. The MOS-HIV questionnaires will be completed at the times indicated in the Schedule of Activities.

8.1.4.3. HIV Treatment Satisfaction Questionnaire: HIVTSQ

The HIVTSQ was developed to evaluate treatments for HIV and patient satisfaction (Appendix 12) (Woodcock, 2006). The original HIVTSQ included 10 items and underwent 2 stages of psychometric validation. Recently, the HIVTSQ was adapted to include injectable treatment for HIV. The adaptation of the HIVTSQ included 2 additional items related to the mode of administration (ie, long-acting intramuscular injection). These are:

- Item 11. How easy or difficult have you been finding your treatment to be recently?
- Item 12. How satisfied are you with the amount of discomfort or pain involved with your present form of treatment?

The current study will be using the HIVTSQs (status version) and the revised HIVTSQc (change version) of the HIVTSQ 12-item questionnaire. The HIVTSQ 12-item questionnaire retains the option of calculating the total score as if it only had the original 10 items (as the original 10 items are included in the HIV-TSQ12). In addition, it allows for calculation of an 11-item scale score

including the "easy/difficult" item (item-11). The "pain/discomfort" item (item-12) will be included in the questionnaire as a stand-alone item to evaluate potentially painful injectables. These measures will assess change in treatment satisfaction over time (in the same participants) and compare current satisfaction with previous treatment satisfaction, from an earlier time-point. The HIVTSQ questionnaires will be completed at the times indicated in the Schedule of Activities.

To understand the choice and lived experiences of participants randomized to the CAB LA+RPV LA arm (including evaluation of differences between those choosing OLI versus those directly transitioning to injectable CAB LA+RPV LA) and in those randomized to the standard-of-care arm, in-depth interviews (IDIs) will be conducted to understand individual perspectives of participants and focus group discussions (FGDs) will be used to capture group perspectives. The FGDs may include 6 to 10 participants including both males and females, or segregated gender groups. Informed consent will be obtained from the participants for the collection of this data. The perceptions and experiences of health care workers managing participants in the trial will also be sought using similar methodology. Approaches to these interviews in all groups are described in detailed interview guides, separately from this protocol.

Additionally, treatment preference will be assessed using the treatment preference question among participants in the CAB LA+RPV LA arm at the timepoints detailed in the Schedule of Activities. A detailed survey on medical resource utilization will also be collected at Month 12 at selected sites.

8.2. Safety Assessments

Details regarding the IDMC are provided in Committees Structure in Appendix 7, Regulatory, Ethical, and Study Oversight Considerations.

Adverse events will be reported and followed by the investigator as specified in Section 8.3, Adverse Events, Serious Adverse Events, and Other Safety Reporting, and Appendix 8, Adverse Events, Serious Adverse Events, Product Quality Complaints, and Other Safety Reporting: Definitions and Procedures for Recording, Evaluating, Follow-Up, and Reporting.

Any clinically relevant changes occurring during the study must be recorded on the Adverse Event section of the CRF.

Any clinically significant abnormalities persisting at the end of the study/early withdrawal will be followed by the investigator until resolution or until a clinically stable condition is reached.

The study will include the following evaluations of safety and tolerability according to the time-points provided in the Schedule of Activities.

8.2.1. Physical Examinations

A complete physical examination will include, at a minimum, assessment of the Cardiovascular, Respiratory, Abdominal and Neurological systems. Complete examination will be performed at screening, baseline (Day 1), Month 1 (in the CAB LA+RPV LA group only), Month 12 and Month

24. Targeted physical examination (directed to the evaluation of new symptoms) will be performed at other study visits as required.

Height and weight will also be measured and recorded as per the Schedule of Activities. Participants must be instructed to wear light clothing, empty their pockets, and take off their shoes before weight measurement. Two readings must be taken for consistent readings, using the same calibrated electronic scales at each visit.

The site of IM injection administration should be assessed at every visit for signs of any possible reaction. See Section 10.4.17 for additional information.

8.2.2. Vital Signs

Blood pressure and pulse/heart rate measurements will be assessed in a supine position with a completely automated device. Manual techniques will be used only if an automated device is not available.

Blood pressure and pulse/heart rate measurements should be preceded by at least 5 minutes of rest in a quiet setting without distractions (eg, television, cell phones).

8.2.3. Clinical Safety Laboratory Assessments

Blood samples for hematology (full blood count); serum chemistry (sodium, potassium, creatinine, glucose, ALT, bilirubin (total, direct and indirect), alkaline phosphatase; a fasting metabolic profile (cholesterol, triglycerides); and a random urine sample for urinalysis will be collected as per the Schedule of Activities and as noted in Appendix 2, Clinical Laboratory Tests. The investigator must review the laboratory results, document this review, and record any clinically relevant changes occurring during the study in the adverse event section of the CRF. The laboratory reports must be filed with the source documents.

8.2.4. Pregnancy Testing

Urine pregnancy tests will be performed at all study visits. Additional urine pregnancy tests may be performed, as determined necessary by the investigator or required by local regulation, to establish the absence of pregnancy at any time during the participation in the study or if experiencing a delayed menstrual period (over 1 month between menstrual cycles) or infrequent or irregular menstrual cycles to confirm absence of pregnancy. Female participants who become pregnant during the study will be permitted to continue the study intervention after providing separate informed consent. Plasma samples for pharmacokinetic tests will be collected from the pregnant participants in the 1st, 2nd and 3rd trimester and postpartum, if feasible.

8.2.5. Dual Energy X-ray Absorptiometry Scan

Measurement of trunk fat (and other body composition compartments) by whole-body DEXA scanning will be done in the first 150 patients enrolled in the study who provide separate written informed consent. DEXA scans will be performed at baseline (within 1 month of enrollment), Month 12 and Month 24. Women who have a DEXA scan at baseline but become pregnant later during the study will defer any scheduled follow-up DEXA scan until 6 months after pregnancy

ends; follow-up scans for these female participants will also be canceled if this 6-month deferral period ends after the Month 24 visit window. The same scanner must be used at each visit.

8.3. Adverse Events, Serious Adverse Events, and Other Safety Reporting

Clinical Adverse Events are defined for this protocol as any new, recurrent, or increased severity (on DAIDS grading scale) of:

- A disease
- A symptom
- A physical sign
- A clinically significant laboratory abnormality

The definition of clinically significant laboratory abnormality is one that:

- Suggests underlying disease and/or organ toxicity that is worsening and/or
- Requires additional active management eg, change of dose, discontinuation of drug, close observation, more frequent follow-up assessments or further diagnostic investigation.

All clinical adverse events will be graded by the site team according to the 2004 Division of AIDS toxicity grading scale (Appendix 5) and will also be evaluated to determine whether they meet the criteria for being considered a serious adverse event (see Section 8.3.2).

All clinical adverse events will be reported on the eCRF. See Section 8.3.1 Time Period and Frequency for Collecting Adverse Event and Serious Adverse Event Information.

Timely, accurate, and complete reporting and analysis of safety information, including AEs, serious AEs, and product quality complaint (PQC), from clinical studies are crucial for the protection of participants, investigators, and the sponsor, and are mandated by regulatory agencies worldwide. The sponsor has established Standard Operating Procedures in conformity with regulatory requirements worldwide to ensure appropriate reporting of safety information; all clinical studies conducted by the sponsor or its affiliates will be conducted in accordance with those procedures.

Adverse events will be reported by the participant (or, when appropriate, by a caregiver, surrogate, or the participant's legally acceptable representative) for the duration of the study.

Further details on AEs, serious AEs, and PQC can be found in Appendix 8, Adverse Events, Serious Adverse Events, Product Quality Complaints, and Other Safety Reporting: Definitions and Procedures for Recording, Evaluating, Follow-Up, and Reporting.

8.3.1. Time Period and Frequency for Collecting Adverse Event and Serious Adverse Event Information

All Adverse Events

All AEs and special reporting situations, whether serious or non-serious, will be reported from the time a signed and dated ICF is obtained until completion of the participant's last study-related procedure, which may include contact for follow-up of safety. Adverse events occurring in the interim between screening and randomization will also be recorded.

Serious Adverse Events

All SAEs, as well as PQC, occurring after randomization must be reported to the appropriate sponsor contact person by study-site personnel within 24 hours of their knowledge of the event.

Serious adverse events, including those spontaneously reported to the investigator within 30 days after the last dose of study intervention, must be reported using a Serious Adverse Event Form. The sponsor will evaluate any safety information that is spontaneously reported by an investigator beyond the time frame specified in the protocol.

Information regarding SAEs will be transmitted to the sponsor using the Serious Adverse Event Form, which must be completed and signed by a physician from the study-site, and transmitted to the sponsor within 24 hours. The initial and follow-up reports of a serious adverse event should be made by facsimile (fax). Telephone reporting should be the exception and the reporter should be asked to complete the appropriate form(s) first.

8.3.2. Method of Detecting Adverse Events and Serious Adverse Events

Care will be taken not to introduce bias when detecting AEs or SAEs. Open-ended and nonleading verbal questioning of the participant is the preferred method to inquire about adverse event occurrence.

Solicited Adverse Events

Solicited AEs are predefined local (at the injection site) and systemic events for which the participant is specifically questioned.

Unsolicited Adverse Events

Unsolicited AEs are all AEs for which the participant is not specifically questioned.

8.3.3. Follow-up of Adverse Events and Serious Adverse Events

The investigator is obligated to perform or arrange for the conduct of supplemental measurements and evaluations as medically indicated to elucidate the nature and causality of the adverse event, serious adverse event, or PQC as fully as possible. This may include additional laboratory tests or investigations, histopathological examinations, or consultation with other health care professionals.

Adverse events, including pregnancy, will be followed by the investigator as specified in Appendix 8, Adverse Events, Serious Adverse Events, Product Quality Complaints, and Other Safety Reporting: Definitions and Procedures for Recording, Evaluating, Follow-up, and Reporting.

8.3.4. Regulatory Reporting Requirements for Serious Adverse Events

The sponsor assumes responsibility for appropriate reporting of AEs to the regulatory authorities. The sponsor will also report to the investigator (and the head of the investigational institute where required) all suspected unexpected serious adverse reactions (SUSARs). The investigator (or sponsor where required) must report SUSARs to the appropriate Independent Ethics Committee/Institutional Review Board (IEC/IRB) that approved the protocol unless otherwise required and documented by the IEC/IRB.

8.3.5. Pregnancy

All initial reports of pregnancy in female participants or partners of male participants must be reported to the sponsor by the study-site personnel within 24 hours of their knowledge of the event using the appropriate pregnancy notification form. Abnormal pregnancy outcomes (eg, spontaneous abortion, fetal death, stillbirth, congenital anomalies, ectopic pregnancy) are considered SAEs and must be reported using a serious adverse event reporting form. Any participant from the CAB LA+RPV LA group who becomes pregnant will be permitted to continue the study after providing separate informed consent.

Plasma samples for pharmacokinetic tests will be collected during 1st, 2nd, 3rd trimester and postpartum as needed. Follow-up information regarding the outcome of the pregnancy and any postnatal sequelae in the infant will be required.

8.3.6. Disease-Related Events and Disease-Related Outcomes Not Qualifying as Adverse Events or Serious Adverse Events

The events or outcomes listed in the classification list (see Appendix 6, HIV-Related Events or Outcomes) will be recorded as HIV-related events on the Adverse Event and HIV-Related Events section of the CRF. Class C events will be collected in a separate CRF page. The purpose of this separate CRF page is to collect detailed information of all the diagnostic procedures to make a presumptive or confirmed diagnosis of any Class C AIDS defining illness that occurred during the study.

All events that meet the definition of a serious adverse event will be reported as SAEs, regardless of whether they are protocol-specific assessments.

8.4. Medical Resource Utilization and Health Economics

Medical resource utilization and health economics data, associated with medical encounters, will be collected in the CRF by the investigator and study-site personnel for all participants throughout the study. Protocol-mandated procedures, tests, and encounters are excluded. The data collected may be used to conduct exploratory economic analyses and will include:

- Rates of opportunistic infections
- Duration of hospitalization (total days length of stay, including duration by wards; eg, intensive care unit)
- Rates of non-adherence to medication
- Rates of drop-out and reason for dropouts
- Rates of loss to follow-up

8.5. Additional Stored Blood Samples for Characterization of HBV Profile in Screen Failures Based on HBV Serology

Potential participants who harbor HBV infection are not ideal candidates for switch to CAB LA + RPV LA because of the risk of reactivation of HBV infection following NRTI withdrawal. Such patients are excluded from the trial based on a positive HBsAg test or positive anti-HBc test at screening. Although positive HBsAg indicates active infection that is associated with a true risk or reactivation after stopping standard oral ART, the combination of a negative HBsAg test combined with a positive anti-HBc test is relatively non-specific for identifying patients at risk of reactivation. In most cases this reflects past infection that has cleared, although in a minority of cases it reflects infection with a mutant HBV strain producing HBsAg that may not be detected by HBsAg assays (Raimondo 2008).

Initial experience in the CARES study has shown that >40% of those screened have been excluded from participation due to this profile (HBsAg negative and anti-HBc positive). A number of these patients may have no HBV and have been denied the opportunity to receive CAB LA+RPV LA. This high rate of assumed ineligibility could represent a major barrier to the use of CAB LA+RPV LA in program settings in sub-Saharan Africa. It is therefore essential to explore what proportion of participants with this HBV profile are at real risk of HBV reactivation and hepatic flares.

To further characterize this population, stored plasma and serum samples from participants who test HBsAg negative and anti-HBc positive at screening will be utilized to further explore the HBV profile (HBV DNA and anti-HBs; with additional HBV-related tests as needed to further characterize the profile and reactivation risk). Where residual samples are available in storage from participants screened earlier in the trial these may also be retrieved for further HBV testing.

Where residual/stored samples are not available from the screening visit, for those screen failures who tested negative for HBsAg but positive for anti-HBc, the participants will be invited for a repeat blood sampling for the testing of HBV DNA and anti-HBs. Informed consent will be obtained from these participants for the repeat blood sampling.

9. STATISTICAL CONSIDERATIONS

Statistical analysis will be done by the sponsor. A general description of the statistical methods to be used to analyze the efficacy and safety data is outlined below. Specific details will be provided in the Statistical Analysis Plan.

9.1. Statistical Hypotheses

Dual long-acting injectable ART with CAB and RPV is non-inferior (non-inferiority margin -10%) in the proportion of participants with plasma HIV-1 RNA <50 c/mL as per FDA Snapshot Algorithm at Month 12 compared to daily triple oral current antiretroviral regimen consisting of INI or NNRTI with a backbone of TDF and with l 3TC/FTC regimen.

9.2. Sample Size Determination

Assuming a true 94% virologic response rate in each group, a non-inferiority margin of -10%, and a 2-sided 5% significance level, a sample size of 256 participants per treatment group will provide at least 90% power to test the hypothesis of non-inferiority of CAB LA+RPV LA Q2M vs. cART. In addition, to account for a testing of key secondary hypothesis of non-inferiority in virologic failure, this sample size will provide with an 85% power at a 2 sided 5% significance level with a non-inferiority margin of 4% and assumption of 1.7% virologic failure in a hierarchical testing procedure. The virologic response rate 94% and virologic failure rate 1.7% were observed on Q2M group from the ATLAS-2M study. Therefore, 256 participants per group with total 512 participants are considered adequate for testing the primary and key secondary hypotheses.

9.3. Populations for Analysis Sets

For purposes of analysis, the following populations are defined:

Population	Description
ITT (intent-to-treat)	All randomly assigned participants who receive at least 1 dose of study intervention
PP (per protocol)	All participants in the ITT population who are without any major protocol deviations
Safety	All randomized participants who take at least 1 dose of study intervention. Participants will be assessed according to the actual treatment they received

9.4. Statistical Analyses

The Statistical Analysis Plan will be finalized prior to database lock and it will include a more technical and detailed description of the statistical analyses described in this section. This section is a summary of the planned statistical analyses of the most important endpoints including primary and key secondary endpoints.

9.4.1. General Considerations

At least 2 analyses will be conducted to evaluate primary and secondary objectives of the protocol, one after all participants have completed their visits at Month 12 and one after Month 24. The Month 12 analysis will be primary. No adjustment for multiplicity caused by repeated evaluation of the primary endpoint will be made as the Month 24 analyses will be secondary.

9.4.2. Primary Endpoint

The primary analysis at Month 12 will take place after the last participant has had their Month 12 viral load assessed, including a retest if required. For the primary efficacy analysis, each participant's virologic response (HIV 1 RNA <50 c/mL) will be calculated according to the FDA Snapshot Algorithm.

The primary analysis will be based on the ITT population. The primary comparison will be made at a two-sided 5% level of significance.

For the primary comparison, the adjusted estimate of the difference in the rate of responders between the 2 groups will be presented along with confidence intervals (CIs) based on a stratified analysis using Cochran Mantel-Haenszel Weighted Miettinen & Nurminen Method. All CIs will be two-sided and the analysis will be stratified according to the baseline third agent class (NNRTI or INI).

Treatment with CAB LA+RPV LA will be considered non-inferior to cART if the lower limit of the CMH-based 95% confidence interval of the difference in efficacy between the treatment groups is >-10%.

Since an analysis on the ITT population may not be conservative in a non-inferiority setting, an analysis based on the PP population will also be performed to investigate the impact of excluding participants with major protocol violations and to evaluate the robustness of the primary analysis results.

9.4.3. Secondary Endpoints

The key secondary endpoint of virologic failure at Month 12 will be analyzed to test the non-inferiority hypothesis. The null hypothesis is that the proportion of participants with virologic failure at Month 12 (CAB LA+RPV LA) is more than 4% higher than that in the control treatment group (cART); the alternative hypothesis is that the virologic failure rate in the CAB LA+RPV LA group is at most 4% higher than that in the control group.

A hierarchical testing procedure will be used for the secondary endpoint of virologic failure. The secondary hypothesis will be conducted only when the primary hypothesis is significant at 2-sided 5% level of significance to demonstrate non-inferiority. The secondary hypothesis will be then conducted at two-sided 5% level of significance. The non-inferiority will be concluded if the upper bound of the 2-sided 95% CI of the difference between treatment groups: (CAB LA+RPV LA group cART group) in virologic failure rate is <4%.

For virologic endpoints at Month 24, analyses will be conducted similarly as for Month 12. The proportion of subjects with confirmed virologic failure will be tabulated using descriptive statistics along with 95% CI. The absolute and change from baseline in CD4+ cell counts will be summarized using descriptive statistics.

9.4.4. Exploratory Endpoints

Please refer to the SAP for details on the evaluation and analysis of the exploratory endpoints in the study.

9.4.5. Safety Analyses

All safety analyses will be made on the Safety Population.

Adverse Events

The verbatim terms used in the CRF by investigators to identify AEs will be coded using the Medical Dictionary for Regulatory Activities (MedDRA). Any AE occurring at or after the initial administration of study intervention through the day of last dose plus 30 days is considered to be treatment-emergent. All reported treatment-emergent AEs will be included in the analysis. For each adverse event, the percentage of participants who experience at least 1 occurrence of the given event will be summarized by intervention group.

Summaries, listings, datasets, or participant narratives may be provided, as appropriate, for those participants who die, who discontinue intervention due to an adverse event, or who experience a severe or a serious adverse event.

Clinical Laboratory Tests

Laboratory data will be summarized by type of laboratory test. Reference ranges and markedly abnormal results (specified in the SAP) will be used in the summary of laboratory data. Descriptive statistics will be calculated for each laboratory analyte at baseline and for observed values and changes from baseline at each scheduled time-point. Frequency tabulations of the laboratory abnormalities will be made. A listing of participants with any laboratory results outside the reference ranges will be provided. A listing of participants with any markedly abnormal laboratory results will also be provided.

Vital Signs

Vital signs including pulse/heart rate and blood pressure (systolic and diastolic, supine and standing) will be summarized over time, using descriptive statistics and/or graphically. The percentage of participants with values beyond clinically important limits will be summarized.

Physical Examinations

Descriptive statistics of changes from baseline will be summarized at each scheduled time-point.

Physical examination findings will be summarized at each scheduled time-point. Descriptive statistics will be calculated at baseline and for observed values and changes from baseline at each scheduled time-point. Frequency tabulations of the abnormalities will be made.

Medical Resource Utilization and Health Economics Analyses

Medical resource utilization and health economics will be descriptively summarized by intervention group.

9.5. Interim Analysis

An IDMC will evaluate interim efficacy, tolerability and safety of CAB LA+RPV LA during the study. The committee will first meet when approximately 50% of the participants have completed the Month 4 visit, and then every 6 months till the end of the study, to perform a blinded review of the available data at that time-point. A planned interim analysis will also be conducted when approximately 60% of participants have completed the Month 6 visit, and will include sample size

reassessment or futility analysis. An IDMC will be established to monitor the planned interim analysis as noted in Committees Structure in Appendix 7, Regulatory, Ethical, and Study Oversight Considerations. The SAP will describe the planned interim analyses in greater detail.

10. SUPPORTING DOCUMENTATION AND OPERATIONAL CONSIDERATIONS

10.1. Appendix 1: Abbreviations

3TC	Lamivudine
c/mL	copies/milliliter
AE	Adverse events
AIDS	Acquired immunodeficiency syndrome
ART	Antiretroviral therapy
ALT	alanine aminotransferase
ARV	Antiretroviral
AST	Aspartate transaminase
BMI	Body mass index
CAB	Cabotegravir
CNS	Central nervous system
cART	Combination antiretroviral therapy
CI	Confidence intervals
DAIDS	Division of AIDS
DEXA	Dual energy X-ray absorptiometry
DILI	Drug-induced liver injury
DTG	Dolutegravir
eCRF	Electronic case report form
ECG	Electrocardiogram
eDC	Electronic data capture
EFV	Efavirenz
FDA	Food and Drug Administration
FSH	Follicle stimulating hormone
FTC	Emtricitabine
GCP	Good Clinical Practice
GI	Gastrointestinal
HBc	Hepatitis B core antibody
HbsAg	Hepatitis B surface antigen
HBV	Hepatitis B virus
HCV	Hepatitis C virus
HIV	Human immunodeficiency virus
HSR	Hypersensitivity Reactions
HIVTSQ	HIV Treatment Satisfaction Questionnaire
HRT	Hormone replacement therapy
IEC	Independent Ethics Committee
IM	Intramuscular
INI	Integrase inhibitor
IB	Investigator’s Brochure
ICF	Informed consent form
ICMJE	International Committee of Medical Journal Editors
IDMC	Independent Data Monitoring Committee
INR	International normalized ratio
IRB	Institutional Review Board
ISR	Injection Site Reactions
ITT	Intent-to-treat
IWRS	Interactive web response system
LA	Long-acting
MRU	Medical resource utilization
MOS-HIV	Medical Outcomes Study HIV Health Survey
NNRTI	Non-nucleoside reverse transcriptase inhibitor
NRTI	Nucleoside reverse transcriptase inhibitor
NVP	Nevirapine
OLI	Oral Lead-in
PE	Primary endpoint

PI	Protease inhibitors
PBMC	Peripheral blood mononuclear cells
PP	Per protocol
PQC	Product Quality Complaint
QoL	Quality-of-life
PRO	Patient-reported outcome(s)
RBC	Red blood dell
RNA	Ribonucleic acid
RPV	Rilpivirine
SAE	Serious adverse event
SE	Secondary endpoint
SmPC	Summary of product characteristics
SAP	Statistical analysis plan
SFU	Safety follow-up
SOC	Standard-of-care
SSA	Sub-Saharan Africa
SUSAR	suspected unexpected serious adverse reaction
TB	Tuberculosis
TDF	Tenofovir disoproxil fumarate
ULN	Upper limit of normal
WBC	White blood cell
WHO	World Health Organization

Definitions of Terms

Electronic source system	Contains data traditionally maintained in a hospital or clinic record to document medical care or data recorded in a CRF as determined by the protocol. Data in this system may be considered source documentation.
PRO	Reports directly from the participant without interpretation by clinician or anyone else.

10.2. Appendix 2: Clinical Laboratory Tests

Only baseline resistance from PBMCs will done at central lab. Virologic failures during follow-up will also have resistance testing performed at the central lab. All other tests will be performed at site laboratories.

Protocol-Required Safety Laboratory Assessments

<table>
<tr><th>Laboratory Assessments</th><th colspan="3">Parameters</th></tr>
<tr><td rowspan="2">Hematology</td><td>Platelet count
Red blood cell count
Hemoglobin
Hematocrit</td><td><u>RBC Indices:</u>
MCV
MCH</td><td><u>White Blood Cell (WBC) count with Differential:</u>
Neutrophils
Lymphocytes
Monocytes
Eosinophils
Basophils</td></tr>
<tr><td colspan="3">Note: A WBC evaluation may include any abnormal cells, which will then be reported by the laboratory. A RBC evaluation may include abnormalities in the RBC count, RBC parameters, or RBC morphology, which will then be reported by the laboratory. In addition, any other abnormal cells in a blood smear will also be reported.</td></tr>
<tr><td rowspan="2">Clinical Chemistry</td><td colspan="2">Sodium
Potassium
Creatinine
Glucose [fasting]
Alanine aminotransferase (ALT)/Serum glutamic-oxaloacetic</td><td>Total, Direct, Indirect bilirubin
Alkaline phosphatase
Cholesterol
Triglycerides</td></tr>
<tr><td colspan="3">Note: Details of liver chemistry stopping criteria and required actions and follow-up are given in Appendix 3 Liver Safety. All events of ALT (or AST) ≥3 × upper limit of normal (ULN) and total bilirubin ≥2 × ULN (>35% direct bilirubin) or ALT (or AST) ≥3 × ULN and international normalized ratio (INR) >1.5, if INR measured which may indicate severe liver injury (possible Hy's Law), must be reported as a serious adverse event (excluding studies of hepatic impairment or cirrhosis).</td></tr>
<tr><td>Routine Urinalysis</td><td colspan="3"><u>Dipstick</u>
Glucose
Protein</td></tr>
<tr><td>Other Screening Tests</td><td colspan="3">• Urine Pregnancy Testing for women of childbearing potential only
• Serology (HIV antibody, hepatitis B surface antigen [HBsAg], anti-HBc and HCV antibody)</td></tr>
</table>

10.3. Appendix 3: Liver Safety: Suggested Actions and Follow-up Assessments

A. STOPPING ALGORITHM

ALT ONLY:

Study intervention will be discontinued for a participant if liver chemistry stopping criteria are met.

Phase 3-4 Liver Chemistry Stopping Criteria and Increased Monitoring Algorithm

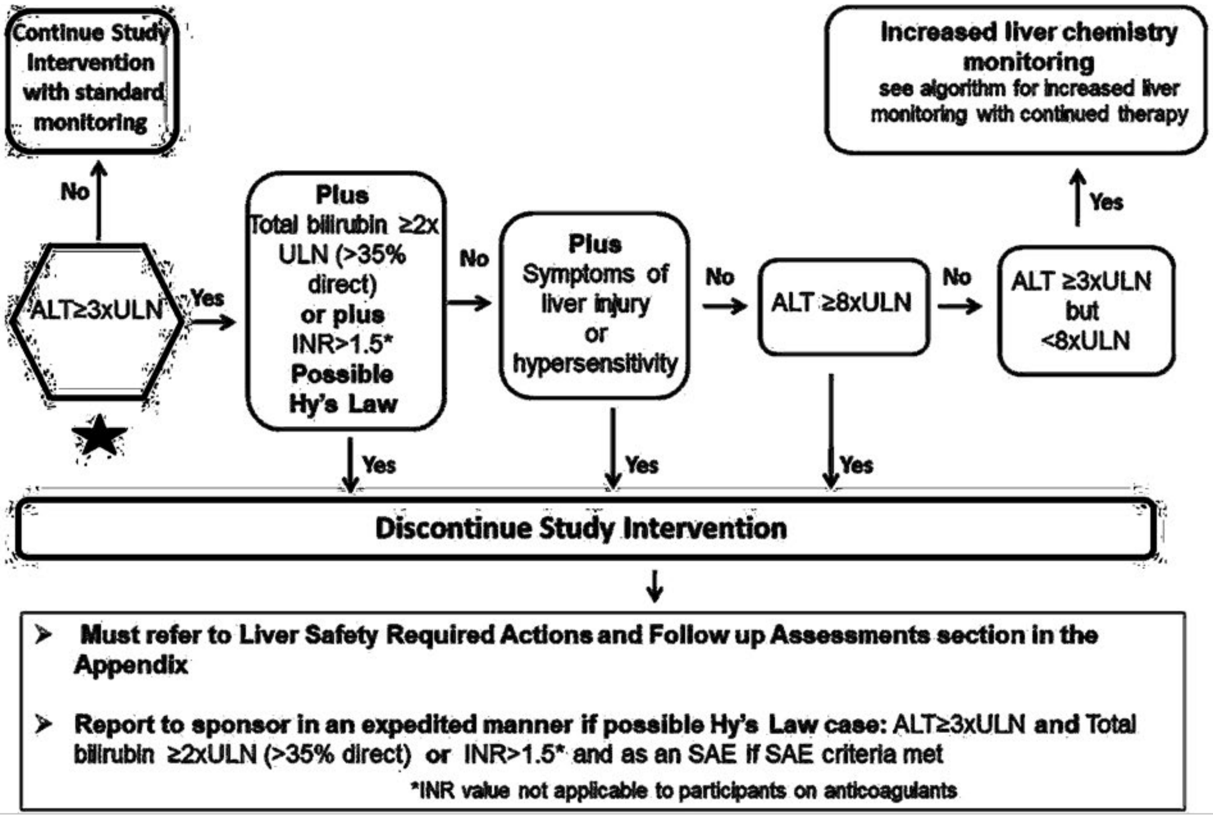

Abbreviations: ALT = alanine transaminase; INR = international normalized ratio; SAE = serious adverse event; ULN = upper limit of normal, Tbili = Total bilirubin

Liver Safety: Suggested Actions and Follow-up Assessments can be found in Appendix 3: Liver Safety: Suggested Actions and Follow-up Assessments

Study intervention will be discontinued for a participant if liver chemistry stopping criteria are met.

Phase 3-4 Liver Chemistry Increased Monitoring Algorithm with Continued Study Intervention for Participants with ALT ≥3xULN but <8xULN

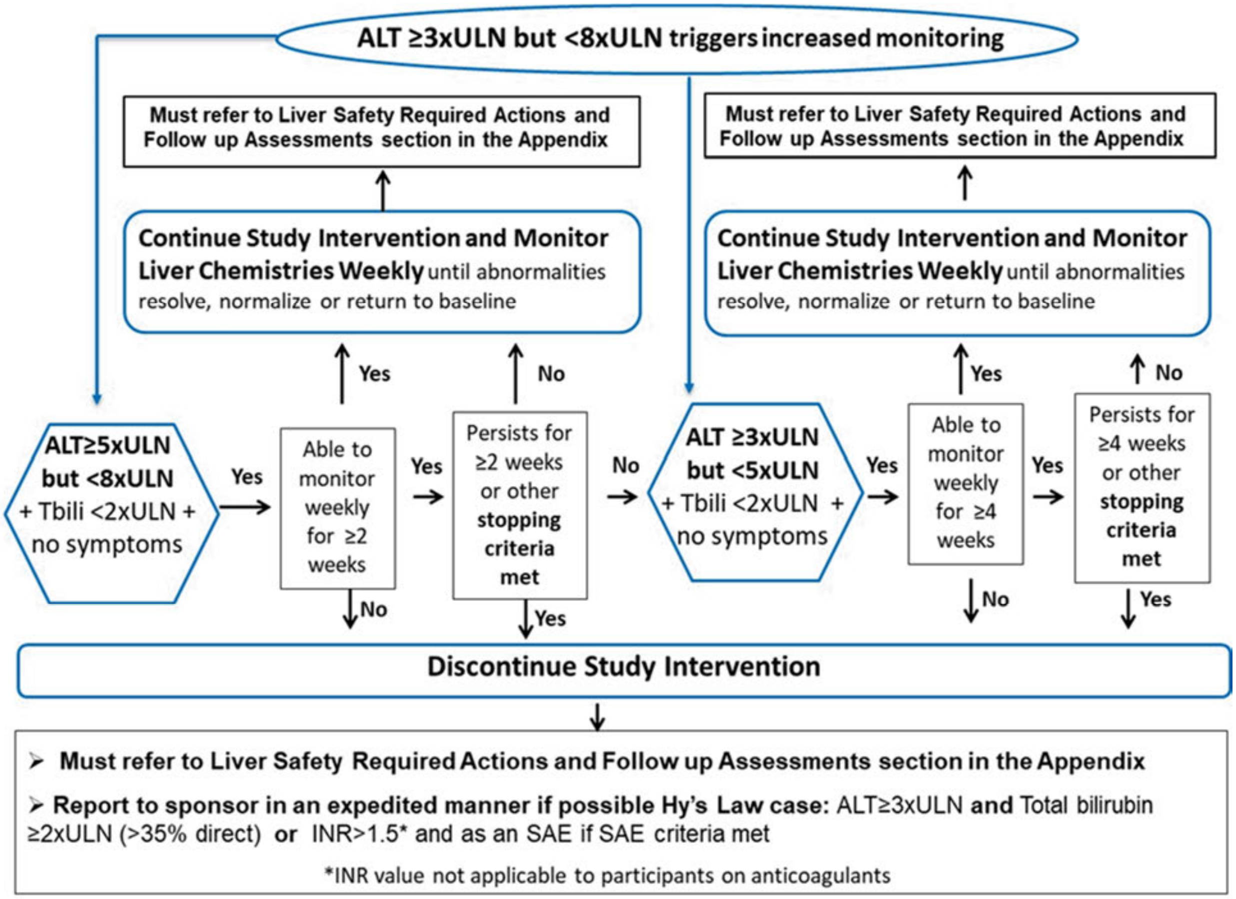

Abbreviations: ALT = alanine transaminase; Tbili = total bilirubin; INR = international normalized ratio; SAE = serious adverse event; ULN = upper limit of normal, Tbili = Total bilirubin.

Refer to Appendix 3: Liver Safety: Suggested Actions and Follow-up Assessments and Study Intervention Rechallenge Guidelines

B. FOLLOW-UP ASSESSMENTS

ALT ONLY:

Phase 3-4 liver chemistry stopping criteria are designed to assure participant safety and to evaluate liver event etiology.

Phase 3-4 Liver Chemistry Stopping Criteria and Follow-Up assessments

**Liver Chemistry Stopping Criteria**	
**ALT-absolute**	ALT ≥8xULN
**ALT Increase**	ALT ≥5xULN but <8xULN persists for ≥2 weeks<br>ALT ≥3xULN but <5xULN persists for ≥4 weeks
**Bilirubin[1, 2]**	ALT ≥3xULN **and** total bilirubin ≥2xULN (>35% direct bilirubin)
**INR[2]**	ALT ≥3xULN **and** international normalized ratio (INR) >1.5, if INR measured
**Cannot Monitor**	ALT ≥5xULN but <8xULN and cannot be monitored weekly for ≥2 weeks<br>ALT ≥3xULN but <5xULN and cannot be monitored weekly for ≥4 weeks
**Symptomatic[3]**	ALT ≥3xULN associated with symptoms (new or worsening) believed to be related to liver injury or hypersensitivity

**Suggested Actions, Monitoring and Follow-up Assessments**	
**Actions**	**Follow-Up Assessments**
• **Immediately** discontinue study intervention<br>• Report the event to the sponsor within **24 hours**<br>• Complete the liver event/expedited reporting form and complete an SAE data collection tool if the event also met the criteria for an SAE[2]<br>• Perform follow-up assessments as described in the Follow-up Assessment column<br>• Monitor the participant until liver chemistry test abnormalities resolve, stabilize, or return to baseline (see **MONITORING**)<br><br>**MONITORING:**<br>**<u>If ALT ≥3xULN AND total bilirubin ≥2xULN or INR >1.5:</u>**<br>• Repeat liver chemistry tests (include ALT, aspartate transaminase [AST], alkaline phosphatase, total bilirubin and INR) and perform liver event follow-up assessments within **24 hours**	• Viral hepatitis serology[4]<br>• Obtain blood sample for pharmacokinetic (PK) analysis after the most recent dose[5]<br>• Obtain a serum creatine phosphokinase (CPK) and lactate dehydrogenase (LDH)<br>• Fractionate bilirubin, if total bilirubin ≥2xULN<br>• Obtain complete blood count with differential to assess eosinophilia<br>• Record the appearance or worsening of clinical symptoms of liver injury, or hypersensitivity liver event/expedited reporting form<br>• Record use of concomitant medications (including acetaminophen, herbal remedies, recreational drugs and other over-the-counter medications)<br>• Record alcohol use on the liver event alcohol intake form<br>**<u>If ALT ≥3xULN AND total bilirubin ≥2xULN or INR >1.5</u>** obtain the following in addition to the assessments listed above**:**

• Monitor participant twice weekly until liver chemistry test abnormalities resolve, stabilize, or return to baseline<br>• A specialist or hepatology consultation is recommended<br>**<u>For all other criteria</u>**<br>• Repeat liver chemistry tests (include ALT, AST, alkaline phosphatase, total bilirubin and INR) and perform liver chemistry follow-up assessments within **24 to 72 hours**<br>• Monitor participants weekly until liver chemistry abnormalities resolve, stabilize, or return to baseline	• Anti-nuclear antibody, anti-smooth muscle antibody, Type 1 anti-liver kidney microsomal antibodies, and quantitative total immunoglobulin G (IgG) or gamma globulins<br>• Serum acetaminophen adduct, when available, assay to assess potential acetaminophen contribution to liver injury in participants with definite or likely acetaminophen use in the preceding week<br>• Liver imaging (ultrasound, magnetic resonance, or computerized tomography) to evaluate liver disease; complete liver Imaging form<br>• Liver biopsy may be discussed with local specialist if available, for instance:<br>– In patients when serology raises the possibility of autoimmune hepatitis (AIH)<br>– In patients when suspected DILI progresses or fails to resolve on withdrawal of study intervention<br>– In patients with acute or chronic atypical presentation: hepatic vascular disorder, chronic hepatitis fibrosis, micro vesicular statosis<br>• If liver biopsy conducted complete liver biopsy form

1. Serum bilirubin fractionation should be performed if testing is available. If serum bilirubin fractionation is not immediately available, discontinue study intervention if ALT ≥3xULN **and** total bilirubin ≥2xULN. Additionally, if serum bilirubin fractionation testing is unavailable, **record the absence/presence of detectable urinary bilirubin on dipstick** which is indicative of direct bilirubin elevations suggesting liver injury.
2. All events of ALT ≥3xULN **and** total bilirubin ≥2xULN (>35% direct bilirubin) or ALT ≥3xULN **and** INR >1.5 may indicate severe liver injury **(possible 'Hy's Law') and must be reported to sponsor in an expedited manner and as an SAE if SAE criteria met (excluding studies of hepatic impairment or cirrhosis)**. The INR stated threshold value will not apply to participants receiving anticoagulants.
3. New or worsening symptoms believed to be related to liver injury (such as fatigue, nausea, vomiting, right upper quadrant pain or tenderness, or jaundice) or hypersensitivity (such as fever, rash or eosinophilia).
4. PK sample may not be required for participants known to be receiving placebo or non-comparator interventions. Record the date/time of the PK blood sample draw and the date/time of the last dose of study intervention prior to the PK blood sample draw on the CRF. If the date or time of the last dose is unclear, provide the participant's best approximation. If the date/time of the last dose cannot be approximated OR a PK sample cannot be collected in the time period indicated above, do not obtain a PK sample.

Phase 3-4 Liver Chemistry Increased Monitoring Criteria with Continued Study Intervention

**Liver Chemistry Increased Monitoring Criteria and Actions with Continued Study Intervention**	
**Criteria**	**Actions**
ALT ≥5xULN and <8xULN **and** total bilirubin <2xULN or INR<1.5 **without** symptoms believed to be related to liver injury or hypersensitivity, **and** who can be monitored weekly for 2 weeks<br>OR<br>ALT ≥3xULN and <5xULN **and** total bilirubin <2xULN **without** symptoms believed to be related to liver injury or hypersensitivity, **and** who can be monitored weekly for 4 weeks	• Notify the sponsor **within 24 hours** of learning of the abnormality to discuss participant safety<br>• Participant must return weekly for repeat liver chemistry tests (ALT, AST, alkaline phosphatase, total bilirubin) until the abnormalities resolve, stabilize, or return to baseline<br>• If at any time, the participant meets liver chemistry stopping criteria, proceed as described above<br>• If ALT decreases from ALT ≥5xULN and <8xULN to ≥3xULN but <5xULN, continue to monitor liver chemistries weekly<br>• If, after 4 weeks of monitoring, ALT <3xULN and total bilirubin <2xULN, monitor participants twice monthly until liver chemistry tests resolve, stabilize, or return to baseline

References

James LP, Letzig L, Simpson PM, et al. Pharmacokinetics of Acetaminophen Adduct in Adults with Acetaminophen Overdose and Acute Liver Failure. Drug Metab Dispos 2009; 37:1779-1784.

Le Gal F, Gordien E, Affolabi D, Hanslik T, Alloui C, Dény P, et al. Quantification of Hepatitis Delta Virus RNA in Serum by Consensus Real-Time PCR Indicates Different Patterns of Virological Response to Interferon Therapy in Chronically Infected Patients. J Clin Microbiol. 2005;43(5):2363 2369.

10.4. Appendix 4: Specific Toxicities

10.4.1. General Guidance

For AEs or toxicities not presented in the subsequent specific toxicity subsections, the guidance in this section should be used for actions related to study intervention.

All AEs will be followed until satisfactory clinical resolution or stabilization (to be agreed upon with the sponsor). All Grade 3 and Grade 4 laboratory abnormalities and laboratory abnormalities resulting in an increase of 2 DAIDS grades or more from baseline will be followed until return to baseline or within 1 grade from baseline.

***Note**: For Grade 3 or 4 laboratory toxicities, participants should have a confirmatory measurement preferably within 48 hours after the laboratory results become available, if possible.*

Grade 1

Participants who develop a Grade 1 adverse event or toxicity may continue intake of study intervention.

Grade 2

Participants who develop a Grade 2 adverse event or toxicity may continue intake of study intervention based on the investigator's clinical judgment.

Grade 3

Participants who develop a Grade 3 adverse event or toxicity should interrupt all study interventions and may resume all medications when the adverse event or laboratory abnormality resolves to within 1 Grade level (≤Grade 2) of the participant's baseline. The following exceptions apply (ie, do not require interruption of study intervention unless clinical assessment foresees an immediate health risk to the participant):

- participants with preexisting diabetes who experience a glucose elevation of Grade 3
- participants who experience a glucose, triglyceride, or cholesterol elevation of Grade 3 under non-fasted conditions
- participants who experience asymptomatic glucose, triglyceride, or cholesterol elevations of Grade 3
- participants who experience a Grade 3 adverse event or Grade 3 laboratory abnormality that is considered not related or doubtfully related to the study intervention.

Grade 4

Participants experiencing a Grade 4 adverse event or toxicity will permanently discontinue the study intervention. The following exceptions apply (ie, do not require permanent discontinuation of study intervention unless clinical assessment foresees an immediate health risk to the participant):

- participants with preexisting diabetes who experience a glucose elevation of Grade 4
- participants who experience a glucose or triglyceride elevation of Grade 4 under non-fasted conditions
- participants who experience asymptomatic glucose or triglyceride elevations of Grade 4
- participants who experience a Grade 4 adverse event or Grade 4 laboratory abnormality that is considered not related or doubtfully related to the study intervention.

10.4.2. Neuropsychological Symptoms

Nervous system-related events, predominantly headache and to a lesser extent dizziness, have been reported with RPV in clinical studies executed to date and will be monitored in all clinical studies with RPV. There have been reports of delusions and inappropriate behavior in participants receiving licensed NNRTIs, especially in participants with a history of mental illness or substance abuse. Severe acute depression (including suicidal ideation/attempts) has also been reported in patients receiving NNRTIs. Participants who experience these symptoms should contact the investigator immediately because medical intervention and discontinuation of the study intervention may be required. Investigators should refer participants reporting such symptoms for immediate psychiatric evaluation/medical intervention.

In case of Grade 3 alterations in personality behavior or in mood (alteration causing inability to perform usual social and functional activities), and Grade 4 alterations in personality behavior or in mood (behavior potentially harmful to self or others [eg, suicidal and homicidal ideation or attempt, acute psychosis] or causing inability to perform basic self-care functions), study intervention must be permanently discontinued if the neuropsychological symptoms are considered at least possibly related to the study intervention in the opinion of the investigator.

10.4.3. Cutaneous Events/Rash

Definitions for each DAIDS toxicity grade for cutaneous events and rashes, along with the required investigator actions related to study intervention are presented in Table 1.

All rashes and cutaneous events must be reported on the adverse event pages of the CRF. All skin events should be evaluated together with other systemic symptoms and laboratory abnormalities. Close clinical follow-up is recommended for participants who have any DAIDS grade cutaneous events or rashes to monitor for any progression of the adverse event. Management will be at the discretion of the investigator, taking into account the actions related to study intervention, and should follow generally accepted medical standards. Cetirizine, levocetirizine, topical corticosteroids, and antipruritic agents will be allowed at the investigator's discretion for all grades of rashes. Systemic corticosteroids will be allowed for Grade 3 and Grade 4 rashes for a limited time period (<4 weeks).

Table 1: Summary of Cutaneous Events/Rash

DAIDS Toxicity Grade	A cutaneous reaction/rash is defined for the indicated grade as follows:	Investigator Action Regarding Study intervention
Grade 1	• Localized macular rash	May continue study intervention or study intervention may be interrupted at the investigator's discretion.
Grade 2	• Diffuse macular, maculo-papular, or morbilliform rash<br>• Target lesions	May continue study intervention or study intervention may be interrupted at the investigator's discretion.
Grade 3	• Diffuse macular, maculo-papular, or morbilliform rash with vesicles or limited number of bullae<br>• Superficial ulcerations of mucous membrane limited to 1 site	Permanently discontinue study intervention.[a]
Grade 4	• Extensive or generalized bullous lesions<br>• Stevens-Johnson syndrome<br>• Ulceration of mucous membrane involving 2 or more distinct mucosal sites<br>• Toxic epidermal necrolysis	Permanently discontinue study intervention.[a]

[a] Participants who have study intervention permanently discontinued must return for a treatment discontinuation visit preferably within 72 hours of the last dose of study intervention and a posttreatment follow-up visit 30 to 35 days after the last dose of study intervention.

10.4.4. Acute Systemic Allergic Reaction

Definitions for each DAIDS toxicity grade for acute systemic allergic reaction, along with the required investigator actions related to study intervention are presented in Table 2.

Management of acute systemic allergic reactions will be at the discretion of the investigator and should follow generally accepted medical standards.

For a Grade 1 or 2 allergic reaction, the participant should be advised to contact the investigator immediately.

For Grade 1 or 2 allergic reactions, antihistamines or topical corticosteroids or antipruritic agents may be prescribed as supportive care.

For a Grade 3 or 4 allergic reaction, participants will be treated as clinically appropriate. Standard management should be undertaken.

Table 2: Summary of Acute Systemic Allergic Reaction

DAIDS Toxicity Grade	An acute systemic allergic reaction is defined for the indicated grade as follows:	Investigator Action Regarding Study intervention
Grade 1	• Localized urticaria (wheals) with no medical intervention indicated	May continue study intervention or study intervention may be interrupted at the investigator's discretion.
Grade 2	• Localized urticaria with medical intervention indicated, or<br>• Mild angioedema with no medical intervention indicated	May continue study intervention or study intervention may be interrupted at the investigator's discretion.
Grade 3	• Generalized urticaria, or<br>• Angioedema with medical intervention indicated, or<br>• Symptomatic mild bronchospasm	Permanently discontinue study intervention.[a]
Grade 4	• Acute anaphylaxis, or<br>• Life-threatening bronchospasm, or<br>• Laryngeal edema	Permanently discontinue study intervention.[a]

a participants who have study intervention permanently discontinued must return for a treatment discontinuation visit preferably within 72 hours of the last dose of study intervention and a posttreatment follow-up visit 30 to 35 days after the last dose of study intervention.

10.4.5. Aspartate Aminotransferase and Alanine Aminotransferase Elevation

Definitions for each DAIDS toxicity grade for AST and ALT elevations, along with the required investigator actions related to study intervention are presented in Table 3.

Management will be at the discretion of the investigator and should follow generally accepted medical standards.

Note*: For Grade 3 or 4 laboratory toxicities, participants should have a confirmatory measurement preferably within 48 hours of receipt of the laboratory alert report.*

Table 3: Summary of AST and ALT Elevations

DAIDS Toxicity Grade	AST and ALT ranges for the indicated grade as follows:	Investigator Action Regarding Study intervention	Rechallenge instructions
Grade 1	≥1.25 to <2.5 x ULN	May continue study intervention	NA
Grade 2	≥2.5 to <5.0 x ULN	May continue study intervention	NA
Grade 3	≥5.0 to <10.0 x ULN	Interrupt study intervention (except if co-infected with hepatitis B or C [see below]).<br>• Upon resolution of abnormality to at a lower Grade level (grade 2 or lower) the participants may resume study intervention under the guidance of the investigator and after the investigator has consulted with a sponsor's physician<br><br>Participants with baseline chronic hepatitis B and/or C:<br>• May continue study intervention if they develop a Grade 3 elevation in AST or ALT providing they:<br>– do not have a Grade 3 or 4 elevation in alkaline phosphatase or total bilirubin or<br>– do not have signs and symptoms of clinically active hepatitis (see Section 10.4.7).<br>• Permanently discontinue study intervention.[a]<br>– If signs or symptoms of clinically active hepatitis occur (see Section 10.4.7), or<br>– If AST or ALT increases to Grade 4	If recurrence of Grade 3 or 4 increase in AST or ALT after restarting study intervention, permanently discontinue the study intervention.[a]
Grade 4	≥10.0 x ULN	Permanently discontinue study intervention.[a]	No

a Participants who have study intervention permanently discontinued must return for a treatment discontinuation visit preferably within 72 hours of the last dose of study intervention and a posttreatment follow-up visit 30 to 35 days after the last dose of study intervention.

ALT= alanine aminotransferase; AST= aspartate aminotransferase; NA=not applicable; ULN=upper limit of laboratory normal range.

10.4.6. Hepatitis B or C

If a participant is diagnosed with acute clinical viral hepatitis during the study, the participant must discontinue study intervention immediately. Participants who have study intervention permanently discontinued must return for a treatment discontinuation visit, preferably within 72 hours of the last dose of study intervention and a posttreatment follow-up visit 30 to 35 days after the last dose of study intervention.

Information on participants with baseline chronic hepatitis B and/or C is presented in Table 3.

Severe acute exacerbations of hepatitis B have been reported in individuals who are co-infected with HBV and HIV-1 and have discontinued TDF or 3TC, which are components of the background regimen. Participants who are co-infected with HIV-1 and HBV should be closely monitored with both clinical and laboratory follow-up for at least several months after stopping treatment with CAB LA+RPV LA. If appropriate, initiation of anti-hepatitis B therapy may be warranted.

10.4.7. Clinical Hepatitis

Participants should be monitored for the development of signs and symptoms of acute hepatitis, which include, but are not limited to, fatigue, malaise, anorexia, nausea, dark urine, and clay-colored stools, bilirubinuria, jaundice, liver tenderness, or hepatomegaly, with or without initially abnormal serum transaminase levels. Participants with severe signs and symptoms suggestive of clinical hepatitis must seek medical attention immediately and have hepatic status assessed. In these cases, the study intervention must be discontinued permanently.

10.4.8. Renal Complications

Renal safety will be monitored at every study visit by evaluating serum creatinine levels, calculate creatinine clearance, and serum chemistry results. Investigator should closely monitor for disturbances in serum creatinine. If renal complications develop, participants must be treated as clinically appropriate. The study intervention may be continued if the renal complication is considered not to be related to the study interventions in the opinion of the investigator.

***Note**: Rare cases of acute renal insufficiency have been reported in participants receiving TDF as part of a combination HIV therapy, more specifically in cases where moderate to severe diarrhea resulted in dehydration. Special attention should be given to the appropriate clinical management of diarrhea, dehydration, and electrolyte losses, if applicable. In certain cases, temporary interruption of HIV therapy may be appropriate until the condition has improved. TDF and 3TC are primarily eliminated by the kidneys. Renal impairment, including cases of acute renal failure and Fanconi syndrome, has been reported in association with the use of TDF. The majority of these cases occurred in participants with underlying systemic or renal disease, or in participants taking nephrotoxic agents; however, some cases occurred in participants without identified risk factors.*

10.4.9. Nausea (With or Without Vomiting)

Although common, nausea following initiation of therapy with ARV medications usually subsides or resolves during the first few weeks of treatment.

Definitions for each DAIDS toxicity grade for nausea, along with the required investigator actions related to study intervention and are presented in Table 4.

Table 4: Summary of Nausea (With or Without Vomiting)

DAIDS Toxicity Grade	Nausea is defined for the indicated grade as follows:	Investigator Action Related to Study intervention
Grade 1	Transient (<24 hours) or intermittent; no or minimal interference with oral intake	May continue study intervention.<br>– Treat as needed with anti-emetics given orally or by suppository.
Grade 2	Persistent nausea resulting in minimal oral intake for 24 to 48 hours	May continue study intervention.<br>– Treat as needed with anti-emetics given orally or by suppository.
Grade 3	Persistent nausea resulting in decreased intake for >48 hours or rehydration indicated (eg, IV fluids)	Interrupt study intervention.<br>– Treat as needed with anti-emetics given orally or by suppository.<br>– Study intervention may be resumed when the nausea resolves to Grade 2 or lower.
Grade 4	Life-threatening consequences (eg, hypotensive shock)	Permanently discontinue study intervention.[a]

a Participants who have study intervention permanently discontinued must return for a treatment discontinuation visit preferably within 72 hours of the last dose of study intervention and a posttreatment follow-up visit 30 to 35 days after the last dose of study intervention.

10.4.10. Diarrhea

Definitions for each DAIDS toxicity grade for diarrhea, along with the required investigator actions related to study intervention are presented in Table 5.

Table 5: Summary of Diarrhea

DAIDS Toxicity Grade	Diarrhea is defined for the indicated grade as follows:	Investigator Action Related to Study intervention
Grade 1	• Transient or intermittent episodes of unformed stools, or<br>• Increase of ≤3 stools over baseline per 24 hours	May continue study intervention.<br>– Loperamide or diphenoxylate can be administered.
Grade 2	• Persistent episodes of unformed to watery stools, or<br>• Increase of ≤4-6 stools over baseline per 24 hours	May continue study intervention.<br>– Loperamide or diphenoxylate can be administered.
Grade 3	• Increase of ≥7 stools per 24 hours, or<br>• Intravenous fluid replacement required	Interrupt study intervention.<br>– Study intervention may be resumed when the diarrhea resolves to Grade 2 or lower.
Grade 4	• Life-threatening consequences (eg, hypotensive shock)	Permanently discontinue study intervention.[a]

a Participants who have study intervention permanently discontinued must return for a treatment discontinuation visit preferably within 72 hours of the last dose of study intervention and a posttreatment follow-up visit 30 to 35 days after the last dose of study intervention.

10.4.11. Hyperglycemia

- Grade 3: 13.89 to <27.75 mmol/L (>250-500 mg/dL)
- Grade 4: ≥27.75 mmol/L (>500 mg/dL)

If elevated glucose levels are from a nonfasting blood draw, a blood draw should be repeated after an 8-hour fast. Management decisions must be based on fasted results. Participants who experience asymptomatic glucose elevations of Grade 3 or 4 and participants with preexisting diabetes may continue study intervention unless clinical assessment foresees an immediate health risk to the participant. Participants with persistent Grade 3 or 4 glucose elevations despite appropriate anti-hyperglycemic treatment should be permanently discontinued with the exception of participants with preexisting diabetes. Appropriate clinical management of hyperglycemia must be started in a timely fashion.

10.4.12. Hypertriglyceridemia and Hypercholesterolemia

Hypertriglyceridemia

- Grade 3: >5.7 to 11.4 mmol/L (>500 to <1000 mg/dL)
- Grade 4: >11.4 mmol/L (>1000 mg/dL)

Hypercholesterolemia

- Grade 3: ≥7.77 mmol/L (≥300 mg/dL)
- Grade 4: not applicable

Management decisions should be based on fasted results. If elevated triglyceride or cholesterol levels are from a nonfasting blood draw, a blood draw should be repeated after an 8-hour fast. Participants who experience asymptomatic triglyceride elevations of Grade 3 or 4, or cholesterol elevations of Grade 3, may continue to receive study intervention.

Hypertriglyceridemia and hypercholesterolemia should be treated according to the specific guidelines for treating HIV-infected participants. Appropriate clinical management of dyslipidemia in the setting of HIV disease should be started in a timely fashion. Investigators may choose to initiate pharmacologic treatment in addition to the usual counseling on diet and exercise. Medications should be introduced with caution. Participants may receive atorvastatin, fluvastatin, pravastatin, or rosuvastatin; however, the lowest possible starting dose of atorvastatin, fluvastatin, or rosuvastatin should be initiated with careful monitoring for signs and symptoms of muscle weakness or myopathy including rhabdomyolysis.

10.4.13. Lactic Acidosis

The relevance of asymptomatic lactic acid elevations is unclear, and lactates are not part of the routine safety evaluations for this study. Routine lactate monitoring is not currently recommended. However, lactate monitoring should be performed if there is a clinical suspicion of lactic acidosis (see description below).

A sometimes-fatal syndrome of lactic acidosis, often associated with evidence of hepatic steatosis, is a recognized but rare complication of N[t]RTI therapy. This syndrome is felt to be secondary to mitochondrial toxicity induced by the inhibitory effect of N[t]RTIs on DNA polymerase gamma, a key enzyme needed for mitochondrial DNA synthesis. Current knowledge regarding this syndrome is incomplete. Obesity and prolonged N[t]RTI exposure may be risk factors. Women

are also at greater risk. Symptoms of lactic acidosis are frequently non-specific such as fatigue, weakness, and fever, but in the majority of cases also include symptoms suggestive of hepatic dysfunction such as nausea, vomiting, abdominal or epigastric discomfort, abdominal distension, hepatomegaly, and new-onset elevated liver enzymes. A high index of suspicion may be required to diagnose this condition. Alternatively, it is possible that unwarranted concern may be raised by over interpretation of lactic acid levels. N[t]RTI toxicity is only one cause of lactic acidosis. Type "B" lactic acid elevations or those without clinically apparent tissue hypoxia are also seen in the context of diabetes mellitus, uremia, liver disease, infections, malignancies, alkaloses, and drug and toxin ingestion of substances such as ethanol, methanol, ethylene glycol, and salicylates.

The following case definition of lactic acidosis, defined differently for symptomatic versus asymptomatic individuals, will be used in this protocol:

Symptomatic Hyperlactatemia

New, otherwise unexplained, persistent (≥2 weeks) occurrence of 1 or more of the following symptoms:

- nausea and vomiting
- abdominal pain or gastric discomfort
- abdominal distension
- increased ALT or AST
- unexplained fatigue
- dyspnea

AND

- lactate level >2 x ULN confirmed by repeat lactate level analysis

***Note**: All lactate levels >2 x ULN should be repeated as soon as possible; generally within 1 week.*

Lactate levels should not be assessed following physical exertion as this causes elevated lactate levels and may confound assessment of the clinical significance of these findings. If the second result confirms hyperlactatemia (>2 x ULN) in participants with symptoms as described above, participants should immediately discontinue their study intervention and return for a treatment discontinuation visit preferably within 72 hours of the last dose of study intervention and a posttreatment follow-up visit 30 to 35 days after the last dose of study intervention. Standard management should be initiated with follow-up to resolution. Processing of the lactate needs to be done according to strict guidelines both in the preparation of the participant (ideally, fasting and with no recent exercise) and in the blood drawing/processing procedure (ideally, the blood is drawn without a tourniquet, the participant does not clench their hand, and the blood is drawn into a chilled tube and processed immediately). Following these guidelines minimizes false elevations of lactates.

10.4.14. Lipodystrophy/Fat Redistribution/Body Changes

Investigators should avoid using the term "lipodystrophy acquired" to describe and report fat redistribution abnormalities associated with ART as this term is not very descriptive. The different

symptoms and gradings are listed in the DAIDS Grading Table under Endocrine/Metabolic (Appendix 5). The relevant terms include: lipohypertrophy, lipoatrophy, and gynecomastia.

Although metabolic abnormalities such as hyperlipidemia or hyperglycemia are often associated with body fat changes, these events should be reported separately for adverse event reporting.

10.4.15. Lipase Elevations

For asymptomatic Grade 1 and 2 lipase elevations participants should be carefully evaluated and followed closely.

For confirmed Grade 3 or 4 elevations of lipase, participants should interrupt all study interventions until lipase returns to Grade ≤2, at which time study intervention could be reintroduced. If Grade 3 or 4 lipase levels persist ≥14 days following interruption of all study interventions, or if the toxicity recurs more than twice, all study interventions should be permanently discontinued and the participants should have a treatment discontinuation visit preferably within 72 hours of the last dose of study intervention and a posttreatment follow-up visit 30 to 35 days after the last dose of study intervention.

10.4.16. Peripheral Neuropathy

Participants should be monitored for the development of peripheral neuropathy, which is usually characterized by numbness, tingling and/or pain in the feet or hands. Peripheral neuropathy has commonly been reported as an adverse event of TDF. Treatment of peripheral neuropathy is according to the physician, but generally begins with non-opioid analgesics, including non-steroidal anti-inflammatory agents and acetaminophen, and the use of tricyclic antidepressants and other agents when more severe symptoms are present.

10.4.17. Injection Site Reactions

Injection site reactions will be managed through investigator assessment throughout the study. All ISRs that are either serious, Grade 3 or higher or persisting beyond 2 weeks must be discussed with the lead scientist/chief investigator to determine etiology and assess appropriate continued study participation. Digital photographs may be documented, where possible, on all participants who have an injection site reaction, with observable findings, that is either serious or Grade 3 or higher, or that persist beyond 2 weeks. Dermatology will be consulted on all participants who have an injection site reaction considered serious, Grade 3 or above, or if clinically significant and persistent beyond 30 days and others if the Investigator or lead scientist/chief investigator feels it is medically necessary.

Details regarding photo collection and any other follow-up will be given by the lead scientist/chief investigator at the time of assessment. ISR discomfort can be managed symptomatically (eg, cold/warm compress, acetaminophen, ibuprofen) if the reaction is interfering with the participant's ability to perform activities of daily living. The required intervention should be documented on the appropriate eCRF page.

10.5. Appendix 5: Division of AIDS Table for Grading the Severity of Adult and Pediatric Adverse Events

The Division of AIDS Table for Grading the Severity of Adult and Pediatric Adverse Events (version 2.1, March 2017), or 'DAIDS Grading Table', is a descriptive terminology to be utilized for adverse event reporting in this study. A grading (severity) scale is provided for each adverse event term.

General Instructions

Estimating Severity Grade

If the need arises to grade a clinical adverse event that is not identified in the DAIDS Grading Table, use the category 'Estimating Severity Grade' located at the top of the table on the following page. In addition, all deaths related to an adverse event are to be classified as Grade 5.

Grading Adult and Pediatric Adverse Events

The DAIDS Grading Table includes parameters for grading both adult and pediatric AEs. When a single set of parameters is not appropriate for grading specific types of AEs for both adult and pediatric populations, separate sets of parameters for adult and/or pediatric populations (with specified respective age ranges) are provided. If there is no distinction in the table between adult and pediatric values for a type of adverse event, then the single set of parameters listed is to be used for grading the severity of both adult and pediatric events of that type.

Determining Severity Grade

If the severity of an adverse event could fall under either 1 of 2 grades (eg, the severity of an adverse event could be either Grade 2 or Grade 3), select the higher of the 2 grades for the adverse event.

Laboratory normal ranges should be taken into consideration to assign gradings to a laboratory value.

Definitions

Basic self-care functions	Adults: activities such as bathing, dressing, toileting, transfer/movement, continence, and feeding
Usual social & functional activities	Activities which adults perform on a routine basis and those which are part of regular activities of daily living, for example:<br><br>Adults: adaptive tasks and desirable activities, such as going to work, shopping, cooking, use of transportation, or pursuing a hobby
Intervention	Medical, surgical, or other procedures recommended or provided by a healthcare professional for the treatment of an adverse event

PARAMETER	GRADE 1<br>MILD	GRADE 2<br>MODERATE	GRADE 3<br>SEVERE	GRADE 4<br>POTENTIALLY LIFE-THREATENING
**Clinical** adverse event **NOT** identified elsewhere in the grading table	Mild symptoms causing no or minimal interference with usual social & functional activities with intervention not indicated	Moderate symptoms causing greater than minimal interference with usual social & functional activities with intervention indicated	Severe symptoms causing inability to perform usual social & functional activities with intervention or hospitalization indicated	Potentially life threatening symptoms causing inability to perform basic self care functions with intervention indicated to prevent permanent impairment, persistent disability, or death

MAJOR CLINICAL CONDITIONS				
CARDIOVASCULAR				
**PARAMETER**	**GRADE 1 MILD**	**GRADE 2 MODERATE**	**GRADE 3 SEVERE**	**GRADE 4 POTENTIALLY LIFE-THREATENING**
**Arrhythmia** (by ECG or physical examination) *Specify type, if applicable*	No symptoms AND No intervention indicated	No symptoms AND Non urgent intervention indicated	Non life threatening symptoms AND Non urgent intervention indicated	Life threatening arrhythmia OR Urgent intervention indicated
**Blood Pressure Abnormalities**[1]				
Hypertension (with the lowest reading taken after repeat testing during a visit) *≥18 years of age*	140 to <160 mmHg systolic OR 90 to <100 mmHg diastolic	≥160 to <180 mmHg systolic OR ≥100 to <110 mmHg diastolic	≥180 mmHg systolic OR ≥110 mmHg diastolic	Life threatening consequences in a participant not previously diagnosed with hypertension (eg, malignant hypertension) OR Hospitalization indicated
<18 years of age	>120/80 mmHg	≥95th to <99th percentile + 5 mmHg adjusted for age, height, and gender (systolic and/or diastolic)	≥99th percentile + 5 mmHg adjusted for age, height, and gender (systolic and/or diastolic)	Life threatening consequences in a participant not previously diagnosed with hypertension (eg, malignant hypertension) OR Hospitalization indicated
Hypotension	No symptoms	Symptoms corrected with oral fluid replacement	Symptoms AND IV fluids indicated	Shock requiring use of vasopressors or mechanical assistance to maintain blood pressure
**Cardiac Ischemia or Infarction** *Report only one*	NA	NA	New symptoms with ischemia (stable angina) OR New testing consistent with ischemia	Unstable angina OR Acute myocardial infarction
**Heart Failure**	No symptoms AND Laboratory or cardiac imaging abnormalities	Symptoms with mild to moderate activity or exertion	Symptoms at rest or with minimal activity or exertion (eg, hypoxemia) OR Intervention indicated (eg, oxygen)	Life threatening consequences OR Urgent intervention indicated (eg, vasoactive medications, ventricular assist device, heart transplant)
**Hemorrhage** (with significant acute blood loss)	NA	Symptoms AND No transfusion indicated	Symptoms AND Transfusion of ≤2 units packed RBCs indicated	Life threatening hypotension OR Transfusion of >2 units packed RBCs (for children, packed RBCs >10 cc/kg) indicated

[1] Blood pressure norms for children <18 years of age can be found in: Expert Panel on Integrated Guidelines for Cardiovascular Health and Risk Reduction in Children and Adolescents. *Pediatrics* 2011;128;S213; originally published online November 14, 2011; DOI: 10.1542/peds.2009-2107C.

MAJOR CLINICAL CONDITIONS				
**CARDIOVASCULAR**				
**PARAMETER**	**GRADE 1 MILD**	**GRADE 2 MODERATE**	**GRADE 3 SEVERE**	**GRADE 4 POTENTIALLY LIFE-THREATENING**
**Prolonged PR Interval or AV Block**<br>*Report only one*<br>*>16 years of age*	PR interval 0.21 to <0.25 seconds	PR interval ≥0.25 seconds OR Type I 2nd degree AV block	Type II 2nd degree AV block OR Ventricular pause ≥3.0 seconds	Complete AV block
≤16 years of age	1st degree AV block (PR interval > normal for age and rate)	Type I 2nd degree AV block	Type II 2nd degree AV block OR Ventricular pause ≥3.0 seconds	Complete AV block
**Prolonged QTc Interval[2]**	0.45 to 0.47 seconds	>0.47 to 0.50 seconds	>0.50 seconds OR ≥0.06 seconds above baseline	Life threatening consequences (eg, Torsade de Pointes, other associated serious ventricular dysrhythmia)
**Thrombosis or Embolism**<br>*Report only one*	NA	Symptoms AND No intervention indicated	Symptoms AND Intervention indicated	Life threatening embolic event (eg, pulmonary embolism, thrombus)

[2] As per Bazett's formula.

DERMATOLOGIC				
**PARAMETER**	**GRADE 1 MILD**	**GRADE 2 MODERATE**	**GRADE 3 SEVERE**	**GRADE 4 POTENTIALLY LIFE-THREATENING**
**Alopecia** (scalp only)	Detectable by study participant, caregiver, or physician AND Causing no or minimal interference with usual social & functional activities	Obvious on visual inspection AND Causing greater than minimal interference with usual social & functional activities	NA	NA
**Bruising**	Localized to one area	Localized to more than one area	Generalized	NA
**Cellulitis**	NA	Non parenteral treatment indicated (eg, oral antibiotics, antifungals, antivirals)	IV treatment indicated (eg, IV antibiotics, antifungals, antivirals)	Life threatening consequences (eg, sepsis, tissue necrosis)
**Hyperpigmentation**	Slight or localized causing no or minimal interference with usual social & functional activities	Marked or generalized causing greater than minimal interference with usual social & functional activities	NA	NA
**Hypopigmentation**	Slight or localized causing no or minimal interference with usual social & functional activities	Marked or generalized causing greater than minimal interference with usual social & functional activities	NA	NA
**Petechiae**	Localized to one area	Localized to more than one area	Generalized	NA
**Pruritus**[3] (without skin lesions)	Itching causing no or minimal interference with usual social & functional activities	Itching causing greater than minimal interference with usual social & functional activities	Itching causing inability to perform usual social & functional activities	NA
**Rash** *Specify type, if applicable*	Localized rash	Diffuse rash OR Target lesions	Diffuse rash AND Vesicles or limited number of bullae OR superficial ulcerations of mucous membrane limited to one site	Extensive or generalized bullous lesions OR Ulceration of mucous membrane involving 2 or more distinct mucosal sites OR Stevens Johnson syndrome OR Toxic epidermal necrolysis

[3] For pruritus associated with injections or infusions, see the *Site Reactions to Injections and Infusions* section.

ENDOCRINE AND METABOLIC				
**PARAMETER**	**GRADE 1 MILD**	**GRADE 2 MODERATE**	**GRADE 3 SEVERE**	**GRADE 4 POTENTIALLY LIFE-THREATENING**
**Diabetes Mellitus**	Controlled without medication	Controlled with medication OR Modification of current medication regimen	Uncontrolled despite treatment modification OR Hospitalization for immediate glucose control indicated	Life threatening consequences (eg, ketoacidosis, hyperosmolar non ketotic coma, end organ failure)
**Gynecomastia**	Detectable by study participant, caregiver, or physician AND Causing no or minimal interference with usual social & functional activities	Obvious on visual inspection AND Causing pain with greater than minimal interference with usual social & functional activities	Disfiguring changes AND Symptoms requiring intervention or causing inability to perform usual social & functional activities	NA
**Hyperthyroidism**	No symptoms AND Abnormal laboratory value	Symptoms causing greater than minimal interference with usual social & functional activities OR Thyroid suppression therapy indicated	Symptoms causing inability to perform usual social & functional activities OR Uncontrolled despite treatment modification	Life threatening consequences (eg, thyroid storm)
**Hypothyroidism**	No symptoms AND Abnormal laboratory value	Symptoms causing greater than minimal interference with usual social & functional activities OR Thyroid replacement therapy indicated	Symptoms causing inability to perform usual social & functional activities OR Uncontrolled despite treatment modification	Life threatening consequences (eg, myxedema coma)
**Lipoatrophy**[4]	Detectable by study participant, caregiver, or physician AND Causing no or minimal interference with usual social & functional activities	Obvious on visual inspection AND Causing greater than minimal interference with usual social & functional activities	Disfiguring changes	NA
**Lipohypertrophy**[5]	Detectable by study participant, caregiver, or physician AND Causing no or minimal interference with usual social & functional activities	Obvious on visual inspection AND Causing greater than minimal interference with usual social & functional activities	Disfiguring changes	NA

[4] Definition: A disorder characterized by fat loss in the face, extremities, and buttocks.
[5] Definition: A disorder characterized by abnormal fat accumulation on the back of the neck, breasts, and abdomen.

GASTROINTESTINAL				
**PARAMETER**	**GRADE 1 MILD**	**GRADE 2 MODERATE**	**GRADE 3 SEVERE**	**GRADE 4 POTENTIALLY LIFE-THREATENING**
**Anorexia**	Loss of appetite without decreased oral intake	Loss of appetite associated with decreased oral intake without significant weight loss	Loss of appetite associated with significant weight loss	Life threatening consequences OR Aggressive intervention indicated (eg, tube feeding, total parenteral nutrition)
**Ascites**	No symptoms	Symptoms AND Intervention indicated (eg, diuretics, therapeutic paracentesis)	Symptoms recur or persist despite intervention	Life threatening consequences
**Bloating or Distension** *Report only one*	Symptoms causing no or minimal interference with usual social & functional activities	Symptoms causing greater than minimal interference with usual social & functional activities	Symptoms causing inability to perform usual social & functional activities	NA
**Cholecystitis**	NA	Symptoms AND Medical intervention indicated	Radiologic, endoscopic, or operative intervention indicated	Life threatening consequences (eg, sepsis, perforation)
**Constipation**	NA	Persistent constipation requiring regular use of dietary modifications, laxatives, or enemas	Obstipation with manual evacuation indicated	Life threatening consequences (eg, obstruction)
**Diarrhea** *≥1 year of age*	Transient or intermittent episodes of unformed stools OR Increase of ≤3 stools over baseline per 24 hour period	Persistent episodes of unformed to watery stools OR Increase of 4 to 6 stools over baseline per 24 hour period	Increase of ≥7 stools per 24 hour period OR IV fluid replacement indicated	Life threatening consequences (eg, hypotensive shock)
<1 year of age	Liquid stools (more unformed than usual) but usual number of stools	Liquid stools with increased number of stools OR Mild dehydration	Liquid stools with moderate dehydration	Life threatening consequences (eg, liquid stools resulting in severe dehydration, hypotensive shock)
**Dysphagia or Odynophagia** *Report only one and specify location*	Symptoms but able to eat usual diet	Symptoms causing altered dietary intake with no intervention indicated	Symptoms causing severely altered dietary intake with intervention indicated	Life threatening reduction in oral intake
**Gastrointestinal Bleeding**	Not requiring intervention other than iron supplement	Endoscopic intervention indicated	Transfusion indicated	Life threatening consequences (eg, hypotensive shock)
**Mucositis or Stomatitis** *Report only one and specify location*	Mucosal erythema	Patchy pseudomembranes or ulcerations	Confluent pseudomembranes or ulcerations OR Mucosal bleeding with minor trauma	Life threatening consequences (eg, aspiration, choking) OR Tissue necrosis OR Diffuse spontaneous mucosal bleeding
**Nausea**	Transient (<24 hours) or intermittent AND No or minimal interference with oral intake	Persistent nausea resulting in decreased oral intake for 24 to 48 hours	Persistent nausea resulting in minimal oral intake for >48 hours OR Rehydration indicated (eg, IV fluids)	Life threatening consequences (eg, hypotensive shock)

GASTROINTESTINAL				
**PARAMETER**	**GRADE 1 MILD**	**GRADE 2 MODERATE**	**GRADE 3 SEVERE**	**GRADE 4 POTENTIALLY LIFE-THREATENING**
**Pancreatitis**	NA	Symptoms with hospitalization not indicated	Symptoms with hospitalization indicated	Life threatening consequences (eg, circulatory failure, hemorrhage, sepsis)
**Perforation** (colon or rectum)	NA	NA	Intervention indicated	Life threatening consequences
**Proctitis**	Rectal discomfort with no intervention indicated	Symptoms causing greater than minimal interference with usual social & functional activities OR Medical intervention indicated	Symptoms causing inability to perform usual social & functional activities OR Operative intervention indicated	Life threatening consequences (eg, perforation)
**Rectal Discharge**	Visible discharge	Discharge requiring the use of pads	NA	NA
**Vomiting**	Transient or intermittent AND No or minimal interference with oral intake	Frequent episodes with no or mild dehydration	Persistent vomiting resulting in orthostatic hypotension OR Aggressive rehydration indicated (eg, IV fluids)	Life threatening consequences (eg, hypotensive shock)

MUSCULOSKELETAL				
**PARAMETER**	**GRADE 1 MILD**	**GRADE 2 MODERATE**	**GRADE 3 SEVERE**	**GRADE 4 POTENTIALLY LIFE-THREATENING**
**Arthralgia**	Joint pain causing no or minimal interference with usual social & functional activities	Joint pain causing greater than minimal interference with usual social & functional activities	Joint pain causing inability to perform usual social & functional activities	Disabling joint pain causing inability to perform basic self care functions
**Arthritis**	Stiffness or joint swelling causing no or minimal interference with usual social & functional activities	Stiffness or joint swelling causing greater than minimal interference with usual social & functional activities	Stiffness or joint swelling causing inability to perform usual social & functional activities	Disabling joint stiffness or swelling causing inability to perform basic self care functions
**Myalgia** (generalized)	Muscle pain causing no or minimal interference with usual social & functional activities	Muscle pain causing greater than minimal interference with usual social & functional activities	Muscle pain causing inability to perform usual social & functional activities	Disabling muscle pain causing inability to perform basic self care functions
**Osteonecrosis**	NA	No symptoms but with radiographic findings AND No operative intervention indicated	Bone pain with radiographic findings OR Operative intervention indicated	Disabling bone pain with radiographic findings causing inability to perform basic self care functions
**Osteopenia**[6]<br>*≥30 years of age*<br>*<30 years of age*	BMD t score 2.5 to 1<br>BMD z score 2 to 1	NA<br>NA	NA<br>NA	NA<br>NA
**Osteoporosis**[6]<br>*≥30 years of age*<br>*<30 years of age*	NA<br>NA	BMD t score < 2.5<br>BMD z score < 2	Pathologic fracture (eg, compression fracture causing loss of vertebral height)<br>Pathologic fracture (eg, compression fracture causing loss of vertebral height)	Pathologic fracture causing life threatening consequences<br>Pathologic fracture causing life threatening consequences

[6] BMD t and z scores can be found in: Kanis JA on behalf of the World Health Organization Scientific Group (2007). Assessment of osteoporosis at the primary health care level. Technical Report. World Health Organization Collaborating Centre for Metabolic Bone Diseases, University of Sheffield, UK. 2007: Printed by the University of Sheffield.

NEUROLOGIC				
**PARAMETER**	**GRADE 1 MILD**	**GRADE 2 MODERATE**	**GRADE 3 SEVERE**	**GRADE 4 POTENTIALLY LIFE-THREATENING**
**Acute CNS Ischemia**	NA	NA	Transient ischemic attack	Cerebral vascular accident (eg, stroke with neurological deficit)
**Altered Mental Status** (for Dementia, see *Cognitive, Behavioral, or Attentional Disturbance* below)	Changes causing no or minimal interference with usual social & functional activities	Mild lethargy or somnolence causing greater than minimal interference with usual social & functional activities	Confusion, memory impairment, lethargy, or somnolence causing inability to perform usual social & functional activities	Delirium OR Obtundation OR Coma
**Ataxia**	Symptoms causing no or minimal interference with usual social & functional activities OR No symptoms with ataxia detected on examination	Symptoms causing greater than minimal interference with usual social & functional activities	Symptoms causing inability to perform usual social & functional activities	Disabling symptoms causing inability to perform basic self care functions
**Cognitive, Behavioral, or Attentional Disturbance** (includes dementia and attention deficit disorder) *Specify type, if applicable*	Disability causing no or minimal interference with usual social & functional activities OR Specialized resources not indicated	Disability causing greater than minimal interference with usual social & functional activities OR Specialized resources on part time basis indicated	Disability causing inability to perform usual social & functional activities OR Specialized resources on a full time basis indicated	Disability causing inability to perform basic self care functions OR Institutionalization indicated
**Developmental Delay** *<18 years of age* *Specify type, if applicable*	Mild developmental delay, either motor or cognitive, as determined by comparison with a developmental screening tool appropriate for the setting	Moderate developmental delay, either motor or cognitive, as determined by comparison with a developmental screening tool appropriate for the setting	Severe developmental delay, either motor or cognitive, as determined by comparison with a developmental screening tool appropriate for the setting	Developmental regression, either motor or cognitive, as determined by comparison with a developmental screening tool appropriate for the setting
**Headache**	Symptoms causing no or minimal interference with usual social & functional activities	Symptoms causing greater than minimal interference with usual social & functional activities	Symptoms causing inability to perform usual social & functional activities	Symptoms causing inability to perform basic self care functions OR Hospitalization indicated OR Headache with significant impairment of alertness or other neurologic function
**Neuromuscular Weakness** (includes myopathy and neuropathy) *Specify type, if applicable*	Minimal muscle weakness causing no or minimal interference with usual social & functional activities OR No symptoms with decreased strength on examination	Muscle weakness causing greater than minimal interference with usual social & functional activities	Muscle weakness causing inability to perform usual social & functional activities	Disabling muscle weakness causing inability to perform basic self care functions OR Respiratory muscle weakness impairing ventilation

NEUROLOGIC				
**PARAMETER**	**GRADE 1 MILD**	**GRADE 2 MODERATE**	**GRADE 3 SEVERE**	**GRADE 4 POTENTIALLY LIFE-THREATENING**
**Neurosensory Alteration** (includes paresthesia and painful neuropathy) *Specify type, if applicable*	Minimal paresthesia causing no or minimal interference with usual social & functional activities OR No symptoms with sensory alteration on examination	Sensory alteration or paresthesia causing greater than minimal interference with usual social & functional activities	Sensory alteration or paresthesia causing inability to perform usual social & functional activities	Disabling sensory alteration or paresthesia causing inability to perform basic self care functions
**Seizures**				
New Onset Seizure *≥18 years of age*	NA	NA	1 to 3 seizures	Prolonged and repetitive seizures (eg, status epilepticus) OR Difficult to control (eg, refractory epilepsy)
<18 years of age (includes new or preexisting febrile seizures)	Seizure lasting <5 minutes with <24 hours postictal state	Seizure lasting 5 to <20 minutes with <24 hours postictal state	Seizure lasting ≥20 minutes OR >24 hours postictal state	Prolonged and repetitive seizures (eg, status epilepticus) OR Difficult to control (eg, refractory epilepsy)
Preexisting Seizure	NA	Increased frequency from previous level of control without change in seizure character	Change in seizure character either in duration or quality (eg, severity or focality)	Prolonged and repetitive seizures (eg, status epilepticus) OR Difficult to control (eg, refractory epilepsy)
**Syncope**	Near syncope without loss of consciousness (eg, pre syncope)	Loss of consciousness with no intervention indicated	Loss of consciousness AND Hospitalization or intervention required	NA

PREGNANCY, PUERPERIUM, AND PERINATAL				
**PARAMETER**	**GRADE 1 MILD**	**GRADE 2 MODERATE**	**GRADE 3 SEVERE**	**GRADE 4 POTENTIALLY LIFE-THREATENING**
**Stillbirth** (report using mother's participant ID) *Report only one*	NA	NA	Fetal death occurring at ≥20 weeks gestation	NA
**Preterm Birth** (report using mother's participant ID)	Live birth at 34 to <37 weeks gestational age	Live birth at 28 to <34 weeks gestational age	Live birth at 24 to <28 weeks gestational age	Live birth at <24 weeks gestational age
**Spontaneous Abortion or Miscarriage**[7] (report using mother's participant ID) *Report only one*	Chemical pregnancy	Uncomplicated spontaneous abortion or miscarriage	Complicated spontaneous abortion or miscarriage	NA

[7] Definition: A pregnancy loss occurring at <20 weeks gestational age.

PSYCHIATRIC				
**PARAMETER**	**GRADE 1 MILD**	**GRADE 2 MODERATE**	**GRADE 3 SEVERE**	**GRADE 4 POTENTIALLY LIFE-THREATENING**
**Insomnia**	Mild difficulty falling asleep, staying asleep, or waking up early causing no or minimal interference with usual social & functional activities	Moderate difficulty falling asleep, staying asleep, or waking up early causing more than minimal interference with usual social & functional activities	Severe difficulty falling asleep, staying asleep, or waking up early causing inability to perform usual social & functional activities requiring intervention or hospitalization	NA
**Psychiatric Disorders** (includes anxiety, depression, mania, and psychosis) *Specify disorder*	Symptoms with intervention not indicated OR Behavior causing no or minimal interference with usual social & functional activities	Symptoms with intervention indicated OR Behavior causing greater than minimal interference with usual social & functional activities	Symptoms with hospitalization indicated OR Behavior causing inability to perform usual social & functional activities	Threatens harm to self or others OR Acute psychosis OR Behavior causing inability to perform basic self care functions
**Suicidal Ideation or Attempt** *Report only one*	Preoccupied with thoughts of death AND No wish to kill oneself	Preoccupied with thoughts of death AND Wish to kill oneself with no specific plan or intent	Thoughts of killing oneself with partial or complete plans but no attempt to do so OR Hospitalization indicated	Suicide attempted

RESPIRATORY				
**PARAMETER**	**GRADE 1 MILD**	**GRADE 2 MODERATE**	**GRADE 3 SEVERE**	**GRADE 4 POTENTIALLY LIFE-THREATENING**
**Acute Bronchospasm**	Forced expiratory volume in 1 second or peak flow reduced to ≥70 to <80% OR Mild symptoms with intervention not indicated	Forced expiratory volume in 1 second or peak flow 50 to <70% OR Symptoms with intervention indicated OR Symptoms causing greater than minimal interference with usual social & functional activities	Forced expiratory volume in 1 second or peak flow 25 to <50% OR Symptoms causing inability to perform usual social & functional activities	Forced expiratory volume in 1 second or peak flow <25% OR Life threatening respiratory or hemodynamic compromise OR Intubation
**Dyspnea or Respiratory Distress** *Report only one*	Dyspnea on exertion with no or minimal interference with usual social & functional activities OR Wheezing OR Minimal increase in respiratory rate for age	Dyspnea on exertion causing greater than minimal interference with usual social & functional activities OR Nasal flaring OR Intercostal retractions OR Pulse oximetry 90 to <95%	Dyspnea at rest causing inability to perform usual social & functional activities OR Pulse oximetry <90%	Respiratory failure with ventilator support indicated (eg, CPAP, BPAP, intubation)

SENSORY				
**PARAMETER**	**GRADE 1 MILD**	**GRADE 2 MODERATE**	**GRADE 3 SEVERE**	**GRADE 4 POTENTIALLY LIFE-THREATENING**
**Hearing Loss** *≥12 years of age*	NA	Hearing aid or intervention not indicated	Hearing aid or intervention indicated	Profound bilateral hearing loss (>80 dB at 2 kHz and above) OR Non serviceable hearing (ie, >50 dB audiogram and <50% speech discrimination)
<12 years of age (based on a 1, 2, 3, 4, 6 and 8 kHz audiogram)	>20 dB hearing loss at ≤4 kHz	>20 dB hearing loss at >4 kHz	>20 dB hearing loss at ≥3 kHz in one ear with additional speech language related services indicated (where available) OR Hearing loss sufficient to indicate therapeutic intervention, including hearing aids	Audiologic indication for cochlear implant and additional speech language related services indicated (where available)
**Tinnitus**	Symptoms causing no or minimal interference with usual social & functional activities with intervention not indicated	Symptoms causing greater than minimal interference with usual social & functional activities with intervention indicated	Symptoms causing inability to perform usual social & functional activities	NA
**Uveitis**	No symptoms AND Detectable on examination	Anterior uveitis with symptoms OR Medical intervention indicated	Posterior or pan uveitis OR Operative intervention indicated	Disabling visual loss in affected eye(s)
**Vertigo**	Vertigo causing no or minimal interference with usual social & functional activities	Vertigo causing greater than minimal interference with usual social & functional activities	Vertigo causing inability to perform usual social & functional activities	Disabling vertigo causing inability to perform basic self care functions
**Visual Changes** (assessed from baseline)	Visual changes causing no or minimal interference with usual social & functional activities	Visual changes causing greater than minimal interference with usual social & functional activities	Visual changes causing inability to perform usual social & functional activities	Disabling visual loss in affected eye(s)

SYSTEMIC				
**PARAMETER**	**GRADE 1 MILD**	**GRADE 2 MODERATE**	**GRADE 3 SEVERE**	**GRADE 4 POTENTIALLY LIFE-THREATENING**
**Acute Allergic Reaction**	Localized urticaria (wheals) with no medical intervention indicated	Localized urticaria with intervention indicated OR Mild angioedema with no intervention indicated	Generalized urticaria OR Angioedema with intervention indicated OR Symptoms of mild bronchospasm	Acute anaphylaxis OR Life threatening bronchospasm OR Laryngeal edema
**Chills**	Symptoms causing no or minimal interference with usual social & functional activities	Symptoms causing greater than minimal interference with usual social & functional activities	Symptoms causing inability to perform usual social & functional activities	NA
**Cytokine Release Syndrome**[8]	Mild signs and symptoms AND Therapy (ie, antibody infusion) interruption not indicated	Therapy (ie, antibody infusion) interruption indicated AND Responds promptly to symptomatic treatment OR Prophylactic medications indicated for ≤24 hours	Prolonged severe signs and symptoms OR Recurrence of symptoms following initial improvement	Life threatening consequences (eg, requiring pressor or ventilator support)
**Fatigue or Malaise**<br>*Report only one*	Symptoms causing no or minimal interference with usual social & functional activities	Symptoms causing greater than minimal interference with usual social & functional activities	Symptoms causing inability to perform usual social & functional activities	Incapacitating symptoms of fatigue or malaise causing inability to perform basic self care functions
**Fever** (non axillary temperatures only)	38.0 to <38.6°C or 100.4 to <101.5°F	≥38.6 to <39.3°C or ≥101.5 to <102.7°F	≥39.3 to <40.0°C or ≥102.7 to <104.0°F	≥40.0°C or ≥104.0°F
**Pain**[9] (not associated with study agent injections and not specified elsewhere)<br>*Specify location*	Pain causing no or minimal interference with usual social & functional activities	Pain causing greater than minimal interference with usual social & functional activities	Pain causing inability to perform usual social & functional activities	Disabling pain causing inability to perform basic self care functions OR Hospitalization indicated
**Serum Sickness**[10]	Mild signs and symptoms	Moderate signs and symptoms AND Intervention indicated (eg, antihistamines)	Severe signs and symptoms AND Higher level intervention indicated (eg, steroids or IV fluids)	Life threatening consequences (eg, requiring pressor or ventilator support)
**Underweight**[11]<br>*>5 to 19 years of age*<br>*2 to 5 years of age*<br>*<2 years of age*	WHO BMI z score < 1 to 2<br>WHO BMI z score < 1 to 2<br>WHO BMI z score < 1 to 2	WHO BMI z score < 2 to 3<br>WHO Weight for height z score < 2 to 3<br>WHO Weight for length z score < 2 to 3	WHO BMI z score < 3<br>WHO Weight for height z score < 3<br>WHO Weight for length z score < 3	WHO BMI z score < 3 with life threatening consequences<br>WHO Weight for height z score < 3 with life threatening consequences<br>WHO Weight for length z score < 3 with life threatening consequences

8 Definition: A disorder characterized by nausea, headache, tachycardia, hypotension, rash, and/or shortness of breath.

9 For pain associated with injections or infusions, see the *Site Reactions to Injections and Infusions* section.

10 Definition: A disorder characterized by fever, arthralgia, myalgia, skin eruptions, lymphadenopathy, marked discomfort, and/or dyspnea.

11 WHO reference tables may be accesed by clicking the desired age range or by accessing the following URLs: http://www.who.int/growthref/who2007 bmi for age/en/ for participants >5 to 19 years of age and http://www.who.int/childgrowth/standards/chart catalogue/en/ for those ≤5 years of age.

SYSTEMIC				
**PARAMETER**	**GRADE 1 MILD**	**GRADE 2 MODERATE**	**GRADE 3 SEVERE**	**GRADE 4 POTENTIALLY LIFE-THREATENING**
**Unintentional Weight Loss** (excludes postpartum weight loss)	NA	5 to <9% loss in body weight from baseline	≥9 to <20% loss in body weight from baseline	≥20% loss in body weight from baseline OR Aggressive intervention indicated (eg, tube feeding, total parenteral nutrition)

URINARY				
**PARAMETER**	**GRADE 1 MILD**	**GRADE 2 MODERATE**	**GRADE 3 SEVERE**	**GRADE 4 POTENTIALLY LIFE-THREATENING**
**Urinary Tract Obstruction**	NA	Signs or symptoms of urinary tract obstruction without hydronephrosis or renal dysfunction	Signs or symptoms of urinary tract obstruction with hydronephrosis or renal dysfunction	Obstruction causing life threatening consequences

SITE REACTIONS TO INJECTIONS AND INFUSIONS				
**PARAMETER**	**GRADE 1 MILD**	**GRADE 2 MODERATE**	**GRADE 3 SEVERE**	**GRADE 4 POTENTIALLY LIFE-THREATENING**
**Injection Site Pain or Tenderness** *Report only one*	Pain or tenderness causing no or minimal limitation of use of limb	Pain or tenderness causing greater than minimal limitation of use of limb	Pain or tenderness causing inability to perform usual social & functional activities	Pain or tenderness causing inability to perform basic self care function OR Hospitalization indicated
**Injection Site Erythema or Redness**[12] *Report only one* *>15 years of age*	2.5 to <5 cm in diameter OR 6.25 to <25 cm^2 surface area AND Symptoms causing no or minimal interference with usual social & functional activities	≥5 to <10 cm in diameter OR ≥25 to <100 cm^2 surface area OR Symptoms causing greater than minimal interference with usual social & functional activities	≥10 cm in diameter OR ≥100 cm^2 surface area OR Ulceration OR Secondary infection OR Phlebitis OR Sterile abscess OR Drainage OR Symptoms causing inability to perform usual social & functional activities	Potentially life threatening consequences (eg, abscess, exfoliative dermatitis, necrosis involving dermis or deeper tissue)
≤15 years of age	≤2.5 cm in diameter	>2.5 cm in diameter with <50% surface area of the extremity segment involved (eg, upper arm or thigh)	≥50% surface area of the extremity segment involved (eg, upper arm or thigh) OR Ulceration OR Secondary infection OR Phlebitis OR Sterile abscess OR Drainage	Potentially life threatening consequences (eg, abscess, exfoliative dermatitis, necrosis involving dermis or deeper tissue)
**Injection Site Induration or Swelling** *Report only one* *>15 years of age*	Same as for **Injection Site Erythema or Redness,** *>15 years of age*	Same as for **Injection Site Erythema or Redness,** *>15 years of age*	Same as for **Injection Site Erythema or Redness,** *>15 years of age*	Same as for **Injection Site Erythema or Redness,** *>15 years of age*
≤15 years of age	Same as for **Injection Site Erythema or Redness,** *≤15 years of age*	Same as for **Injection Site Erythema or Redness,** *≤15 years of age*	Same as for **Injection Site Erythema or Redness,** *≤15 years of age*	Same as for **Injection Site Erythema or Redness,** *≤15 years of age*
**Injection Site Pruritus**	Itching localized to the injection site that is relieved spontaneously or in <48 hours of treatment	Itching beyond the injection site that is not generalized OR Itching localized to the injection site requiring ≥48 hours treatment	Generalized itching causing inability to perform usual social & functional activities	NA

[12] Injection Site Erythema or Redness should be evaluated and graded using the greatest single diameter or measured surface area.

LABORATORY VALUES*				
**CHEMISTRIES**				
**PARAMETER**	**GRADE 1 MILD**	**GRADE 2 MODERATE**	**GRADE 3 SEVERE**	**GRADE 4 POTENTIALLY LIFE-THREATENING**
**Acidosis**	NA	pH ≥7.3 to <LLN	pH <7.3 without life threatening consequences	pH <7.3 with life threatening consequences
**Albumin, Low** (g/dL; *g/L*)	3.0 to <LLN<br>*30 to <LLN*	≥2.0 to <3.0<br>*≥20 to <30*	<2.0<br>*<20*	NA
**Alkaline Phosphatase, High**	1.25 to <2.5×ULN	2.5 to <5.0×ULN	5.0 to <10.0×ULN	≥10.0×ULN
**Alkalosis**	NA	pH >ULN to ≤7.5	pH >7.5 without life threatening consequences	pH >7.5 with life threatening consequences
**ALT or SGPT, High**<br>*Report only one*	1.25 to <2.5×ULN	2.5 to <5.0×ULN	5.0 to <10.0×ULN	≥10.0×ULN
**Amylase (Pancreatic) or Amylase (Total), High**<br>*Report only one*	1.1 to <1.5×ULN	1.5 to <3.0×ULN	3.0 to <5.0×ULN	≥5.0×ULN
**AST or SGOT, High**<br>*Report only one*	1.25 to <2.5×ULN	2.5 to <5.0×ULN	5.0 to <10.0×ULN	≥10.0×ULN
**Bicarbonate, Low** (mEq/L; *mmol/L*)	16.0 to <LLN<br>*16.0 to <LLN*	11.0 to <16.0<br>*11.0 to <16.0*	8.0 to <11.0<br>*8.0 to <11.0*	<8.0<br>*<8.0*
**Bilirubin**<br>***Direct Bilirubin[13], High***<br>*>28 days of age*	NA	NA	>ULN with other signs and symptoms of hepatotoxicity	>ULN with life threatening consequences (eg, signs and symptoms of liver failure)
≤28 days of age	ULN to ≤1 mg/dL	>1 to ≤1.5 mg/dL	>1.5 to ≤2 mg/dL	>2 mg/dL
Total Bilirubin, High<br>*>28 days of age*	1.1 to <1.6×ULN	1.6 to <2.6×ULN	2.6 to <5.0×ULN with other signs and symptoms of hepatotoxicity	≥5.0×ULN with life threatening consequences (eg, signs and symptoms of liver failure)
≤28 days of age	See Appendix A. Total Bilirubin for Term and Preterm Neonates	See Appendix A. Total Bilirubin for Term and Preterm Neonates	See Appendix A. Total Bilirubin for Term and Preterm Neonates	See Appendix A. Total Bilirubin for Term and Preterm Neonates
**Calcium, High** (mg/dL; *mmol/L*)<br>*≥7 days of age*	10.6 to <11.5<br>*2.65 to <2.88*	11.5 to <12.5<br>*2.88 to <3.13*	12.5 to <13.5<br>*3.13 to <3.38*	≥13.5<br>*≥3.38*
<7 days of age	11.5 to <12.4<br>*2.88 to <3.10*	12.4 to <12.9<br>*3.10 to <3.23*	12.9 to <13.5<br>*3.23 to <3.38*	≥13.5<br>*≥3.38*
**Calcium (Ionized), High** (mg/dL; *mmol/L*)	>ULN to <6.0<br>*>ULN to <1.5*	6.0 to <6.4<br>*1.5 to <1.6*	6.4 to <7.2<br>*1.6 to <1.8*	≥7.2<br>*≥1.8*
**Calcium, Low** (mg/dL; *mmol/L*)<br>*≥7 days of age*	7.8 to <8.4<br>*1.95 to <2.10*	7.0 to <7.8<br>*1.75 to <1.95*	6.1 to <7.0<br>*1.53 to <1.75*	<6.1<br>*<1.53*
<7 days of age	6.5 to <7.5<br>*1.63 to <1.88*	6.0 to <6.5<br>*1.50 to <1.63*	5.50 to <6.0<br>*1.38 to <1.50*	<5.50<br>*<1.38*
**Calcium (Ionized), Low** (mg/dL; *mmol/L*)	<LLN to 4.0<br>*<LLN to 1.0*	3.6 to <4.0<br>*0.9 to <1.0*	3.2 to <3.6<br>*0.8 to <0.9*	<3.2<br>*<0.8*

* Reminder: An asymptomatic abnormal laboratory finding without an accompanying AE should not be reported to DAIDS in an expedited time frame unless it meets protocol-specific reporting requirements.

13 Direct bilirubin >1.5 mg/dL in a participant <28 days of age should be graded as Grade 2, if <10% of the total bilirubin.

LABORATORY VALUES				
CHEMISTRIES				
**PARAMETER**	**GRADE 1 MILD**	**GRADE 2 MODERATE**	**GRADE 3 SEVERE**	**GRADE 4 POTENTIALLY LIFE-THREATENING**
**Cardiac Troponin I, High**	NA	NA	NA	Levels consistent with myocardial infarction or unstable angina as defined by the local laboratory
**Creatine Kinase, High**	3 to <6×ULN	6 to <10×ULN	10 to <20×ULN	≥20×ULN
**Creatinine, High**<br>** Report only one*	1.1 to 1.3×ULN	>1.3 to 1.8×ULN OR Increase to 1.3 to <1.5 mg/dL above baseline	>1.8 to <3.5×ULN OR Increase of 1.5 to <2.0×participant's baseline	≥3.5×ULN OR Increase of ≥2.0×participant's baseline
**Creatinine Clearance[14] or eGFR, Low**<br>** Report only one*	NA	<90 to 60 ml/min or ml/min/1.73 m² OR 10 to <30% decrease from participant's baseline	<60 to 30ml/min or ml/min/1.73 m² OR 30 to <50% decrease from participant's baseline	<30 ml/min or ml/min/1.73 m² OR ≥50% decrease from participant's baseline or dialysis needed
**Glucose**<br>(mg/dL; *mmol/L*)<br>***Fasting, High***<br>***Nonfasting, High***	110 to 125<br>*6.11 to <6.95*<br><br>116 to 160<br>*6.44 to <8.89*	>125 to 250<br>*6.95 to <13.89*<br><br>>160 to 250<br>*8.89 to <13.89*	>250 to 500<br>*13.89 to <27.75*<br><br>>250 to 500<br>*13.89 to <27.75*	>500<br>*≥27.75*<br><br>>500<br>*≥27.75*
**Glucose, Low**<br>(mg/dL; *mmol/L*)<br>*≥1 month of age*<br>*<1 month of age*	55 to 64<br>*3.05 to 3.55*<br><br>50 to 54<br>*2.78 to 3.00*	40 to <55<br>*2.22 to <3.05*<br><br>40 to <50<br>*2.22 to <2.78*	30 to <40<br>*1.67 to <2.22*<br><br>30 to <40<br>*1.67 to <2.22*	<30<br>*<1.67*<br><br><30<br>*<1.67*
**Lactate, High**	ULN to <2.0×ULN without acidosis	≥2.0×ULN without acidosis	Increased lactate with pH <7.3 without life threatening consequences	Increased lactate with pH <7.3 with life threatening consequences
**Lipase, High**	1.1 to <1.5×ULN	1.5 to <3.0×ULN	3.0 to <5.0×ULN	≥5.0×ULN
**Lipid Disorders**<br>(mg/dL; *mmol/L*)<br>***Cholesterol, Fasting, High***<br>*≥18 years of age*<br>*<18 years of age*	 <br>200 to <240<br>*5.18 to <6.19*<br><br>170 to <200<br>*4.40 to <5.15*	 <br>240 to <300<br>*6.19 to <7.77*<br><br>200 to <300<br>*5.15 to <7.77*	 <br>≥300<br>*≥7.77*<br><br>≥300<br>*≥7.77*	 <br>NA<br><br><br>NA
LDL, Fasting, High<br>*≥18 years of age*<br>*>2 to <18 years of age*	130 to <160<br>*3.37 to <4.12*<br>110 to <130<br>*2.85 to <3.34*	160 to <190<br>*4.12 to <4.90*<br>130 to <190<br>*3.34 to <4.90*	≥190<br>*≥4.90*<br>≥190<br>*≥4.90*	NA<br><br>NA
Triglycerides, Fasting, High	150 to 300<br>*1.71 to 3.42*	>300 to 500<br>*>3.42 to 5.7*	>500 to <1,000<br>*>5.7 to 11.4*	>1,000<br>*>11.4*
**Magnesium[15], Low**<br>(mEq/L; *mmol/L*)	1.2 to <1.4<br>*0.60 to <0.70*	0.9 to <1.2<br>*0.45 to <0.60*	0.6 to <0.9<br>*0.30 to <0.45*	<0.6<br>*<0.30*
**Phosphate, Low**<br>(mg/dL; *mmol/L*)<br>*>14 years of age*<br>*1 to 14 years of age*<br><br>*<1 year of age*	2.0 to <LLN<br>*0.65 to <LLN*<br><br>3.0 to <3.5<br>*0.97 to <1.13*<br>3.5 to <4.5<br>*1.13 to <1.45*	1.4 to <2.0<br>*0.45 to <0.65*<br><br>2.5 to <3.0<br>*0.81 to <0.97*<br>2.5 to <3.5<br>*0.81 to <1.13*	1.0 to <1.4<br>*0.32 to <0.45*<br><br>1.5 to <2.5<br>*0.48 to <0.81*<br>1.5 to <2.5<br>*0.48 to <0.81*	<1.0<br>*<0.32*<br><br><1.5<br>*<0.48*<br><1.5<br>*<0.48*

[14] Use the applicable formula (ie, Cockroft-Gault in mL/min or Schwatrz, MDRD, CKD-Epi in mL/min/1.73m²). Sites should choose the method defined in their study and when not specified, use the method most relevant to the study population.

[15] To convert a magnesium value from mg/dL to mmol/L, laboratories should multiply by 0.4114.

* Reminder: Choose the method that selects for the higher grade.

LABORATORY VALUES				
**CHEMISTRIES**				
**PARAMETER**	**GRADE 1 MILD**	**GRADE 2 MODERATE**	**GRADE 3 SEVERE**	**GRADE 4 POTENTIALLY LIFE-THREATENING**
**Potassium, High**<br>(mEq/L; *mmol/L*)	5.6 to <6.0<br>*5.6 to <6.0*	6.0 to <6.5<br>*6.0 to <6.5*	6.5 to <7.0<br>*6.5 to <7.0*	≥7.0<br>*≥7.0*
**Potassium, Low**<br>(mEq/L; *mmol/L*)	3.0 to <3.4<br>*3.0 to <3.4*	2.5 to <3.0<br>*2.5 to <3.0*	2.0 to <2.5<br>*2.0 to <2.5*	<2.0<br>*<2.0*
**Sodium, High**<br>(mEq/L; *mmol/L*)	146 to <150<br>*146 to <150*	150 to <154<br>*150 to <154*	154 to <160<br>*154 to <160*	≥160<br>*≥160*
**Sodium, Low**<br>(mEq/L; *mmol/L*)	130 to <135<br>*130 to <135*	125 to <130<br>*125 to <130*	121 to <125<br>*121 to <125*	≤120<br>*≤120*
**Uric Acid, High**<br>(mg/dL; *mmol/L*)	7.5 to <10.0<br>*0.45 to <0.59*	10.0 to <12.0<br>*0.59 to <0.71*	12.0 to <15.0<br>*0.71 to <0.89*	≥15.0<br>*≥0.89*

LABORATORY VALUES				
**HEMATOLOGY**				
**PARAMETER**	**GRADE 1<br>MILD**	**GRADE 2<br>MODERATE**	**GRADE 3<br>SEVERE**	**GRADE 4<br>POTENTIALLY LIFE-THREATENING**
**Absolute CD4+ Count, Low**<br>(cell/mm^3; *cells/L*)<br>*>5 years of age (not HIV infected)*	300 to <400<br>*300 to <400*	200 to <300<br>*200 to <300*	100 to <200<br>*100 to <200*	<100<br>*<100*
**Absolute Lymphocyte Count, Low**<br>(cell/mm^3; *cells/L*)<br>*>5 years of age (not HIV infected)*	600 to <650<br>*0.600×10^9 to <0.650×10^9*	500 to <600<br>*0.500×10^9 to <0.600×10^9*	350 to <500<br>*0.350×10^9 to <0.500×10^9*	<350<br>*<0.350×10^9*
**Absolute Neutrophil Count, Low**<br>(cells/mm^3; *cells/L*)<br>*>7 days of age*	800 to 1,000<br>*0.800×10^9 to 1.000×10^9*	600 to 799<br>*0.600×10^9 to 0.799×10^9*	400 to 599<br>*0.400×10^9 to 0.599×10^9*	<400<br>*<0.400×10^9*
2 to 7 days of age	1,250 to 1,500<br>*1.250×10^9 to 1.500×10^9*	1,000 to 1,249<br>*1.000×10^9 to 1.249×10^9*	750 to 999<br>*0.750×10^9 to 0.999×10^9*	<750<br>*<0.750×10^9*
≤1 day of age	4,000 to 5,000<br>*4.000×10^9 to 5.000×10^9*	3,000 to 3,999<br>*3.000×10^9 to 3.999×10^9*	1,500 to 2,999<br>*1.500×10^9 to 2.999×10^9*	<1,500<br>*<1.500×10^9*
**Fibrinogen, Decreased** (mg/dL; *g/L*)	100 to <200<br>*1.00 to <2.00*<br>OR<br>0.75 to <1.00×LLN	75 to <100<br>*0.75 to <1.00*<br>OR<br>≥0.50 to <0.75×LLN	50 to <75<br>*0.50 to <0.75*<br>OR<br>0.25 to <0.50×LLN	<50<br>*<0.50*<br>OR<br><0.25×LLN<br>OR Associated with gross bleeding
**Hemoglobin[16], Low**<br>(g/dL; *mmol/L*)[17]<br>*≥13 years of age (male only)*	10.0 to 10.9<br>*6.19 to 6.76*	9.0 to <10.0<br>*5.57 to <6.19*	7.0 to <9.0<br>*4.34 to <5.57*	<7.0<br>*<4.34*
≥13 years of age (female only)	9.5 to 10.4<br>*5.88 to 6.48*	8.5 to <9.5<br>*5.25 to <5.88*	6.5 to <8.5<br>*4.03 to <5.25*	<6.5<br>*<4.03*
57 days of age to <13 years of age (male and female)	9.5 to 10.4<br>*5.88 to 6.48*	8.5 to <9.5<br>*5.25 to <5.88*	6.5 to <8.5<br>*4.03 to <5.25*	<6.5<br>*<4.03*
36 to 56 days of age (male and female)	8.5 to 9.6<br>*5.26 to 5.99*	7.0 to <8.5<br>*4.32 to <5.26*	6.0 to <7.0<br>*3.72 to <4.32*	<6.0<br>*<3.72*
22 to 35 days of age (male and female)	9.5 to 11.0<br>*5.88 to 6.86*	8.0 to <9.5<br>*4.94 to <5.88*	6.7 to <8.0<br>*4.15 to <4.94*	<6.7<br>*<4.15*
8 to ≤21 days of age (male and female)	11.0 to 13.0<br>*6.81 to 8.10*	9.0 to <11.0<br>*5.57 to <6.81*	8.0 to <9.0<br>*4.96 to <5.57*	<8.0<br>*<4.96*
≤7 days of age (male and female)	13.0 to 14.0<br>*8.05 to 8.72*	10.0 to <13.0<br>*6.19 to <8.05*	9.0 to <10.0<br>*5.59 to <6.19*	<9.0<br>*<5.59*
**INR, High**<br>(not on anticoagulation therapy)	1.1 to <1.5×ULN	1.5 to <2.0×ULN	2.0 to <3.0×ULN	≥3.0×ULN
**Methemoglobin** (% hemoglobin)	5.0 to <10.0%	10.0 to <15.0%	15.0 to <20.0%	≥20.0%

[17] Male and female sex are defined as sex at birth. For transgender participants ≥13 years of age who have been on hormone therapy for more than 6 consecutive months, grade hemoglobin based on the gender with which they identify (ie, a transgender female should be graded using the female sex at birth hemoglobin laboratory values).

[18] The most commonly used conversion factor to convert g/dL to mmol/L is 0.6206. For grading hemoglobin results obtained by an analytic method with a conversion factor other than 0.6206, the result must be converted to g/dL using the appropriate conversion factor for the particular laboratory.

LABORATORY VALUES				
**HEMATOLOGY**				
**PARAMETER**	**GRADE 1 MILD**	**GRADE 2 MODERATE**	**GRADE 3 SEVERE**	**GRADE 4 POTENTIALLY LIFE-THREATENING**
**PTT, High** (not on anticoagulation therapy)	1.1 to <1.66×ULN	1.66 to <2.33×ULN	2.33 to <3.00×ULN	≥3.00×ULN
**Platelets, Decreased** (cells/mm^3; *cells/L*)	100,000 to <125,000 *100.000×10^9 to <125.000×10^9*	50,000 to <100,000 *50.000×10^9 to <100.000×10^9*	25,000 to <50,000 *25.000×10^9 to <50.000×10^9*	<25,000 *<25.000×10^9*
**PT, High** (not on anticoagulation therapy	1.1 to <1.25×ULN	1.25 to <1.50×ULN	1.50 to <3.00×ULN	≥3.00×ULN
**WBC, Decreased** (cells/mm^3; *cells/L*)				
>7 days of age	2,000 to 2,499 *2.000×10^9 to 2.499×10^9*	1,500 to 1,999 *1.500×10^9 to 1.999×10^9*	1,000 to 1,499 *1.000×10^9 to 1.499×10^9*	<1,000 *<1.000×10^9*
≤7 days of age	5,500 to 6,999 *5.500×10^9 to 6.999×10^9*	4,000 to 5,499 *4.000×10^9 to 5.499×10^9*	2,500 to 3,999 *2.500×10^9 to 3.999×10^9*	<2,500 *<2.500×10^9*

LABORATORY VALUES				
**URINALYSIS**				
**PARAMETER**	**GRADE 1 MILD**	**GRADE 2 MODERATE**	**GRADE 3 SEVERE**	**GRADE 4 POTENTIALLY LIFE-THREATENING**
**Glycosuria** (random collection tested by dipstick)	Trace to 1+ or ≤250 mg	2+ or >250 to ≤500 mg	>2+ or >500 mg	NA
**Hematuria** (not to be reported based on dipstick findings or on blood believed to be of menstrual origin)	6 to <10 RBCs per high power field	≥10 RBCs per high power field	Gross, with or without clots OR With RBC casts OR Intervention indicated	Life threatening consequences
**Proteinuria** (random collection tested by dipstick)	1+	2+	3+ or higher	NA

10.6. Appendix 6: CDC Classification for HIV-1 Infection (2014)

Note that the CD4+ T-lymphocyte count takes precedence over the CD4+ T-lymphocyte percentage in HIV infection stages 1, 2, and 3. The CD4+ T-lymphocyte should only be considered if the count is missing.

HIV infection, stage 0

Indicates early HIV infection, inferred from a negative or indeterminate HIV test result within 180 days of a positive result. The criteria for stage 0 supersede and are independent of criteria used for other stages.

HIV infection, stage 1

- Laboratory confirmation of HIV infection with no AIDS-defining condition, and
  - CD4+ T-lymphocyte count of ≥500 cells/µL, or
  - CD4+ T-lymphocyte percentage of total lymphocytes of ≥26%.

HIV infection, stage 2

- Laboratory confirmation of HIV infection with no AIDS-defining condition, and
  - CD4+ T-lymphocyte count of 200 to 499 cells/µL, or
  - CD4+ T-lymphocyte percentage of total lymphocytes of 14% to 25%.

HIV infection, stage 3 (AIDS)

- Laboratory confirmation of HIV infection, and
  - CD4+ T-lymphocyte count of <200 cells/µL, or
  - CD4+ T-lymphocyte percentage of total lymphocytes of <14%, or
  - Documentation of an AIDS-defining condition (see below).

Documentation of an AIDS-defining condition supersedes a CD4+ T-lymphocyte count of >200 cells/µL and a CD4+ T-lymphocyte percentage of total lymphocytes of >14%.

HIV infection, stage unknown

- Laboratory confirmation of HIV infection, and
  - No information on CD4+ T-lymphocyte count or percentage, and
  - No information on presence of AIDS-defining conditions.

Stage-3-defining opportunistic illnesses in HIV infection

- Candidiasis of bronchi, trachea, or lungs
- Candidiasis of esophagus
- Cervical cancer, invasive
- Coccidioidomycosis, disseminated or extrapulmonary

- Cryptococcosis, extrapulmonary
- Cryptosporidiosis, chronic intestinal (>1 month's duration)
- Cytomegalovirus disease (other than liver, spleen, or nodes), onset at age >1
- month
- Cytomegalovirus retinitis (with loss of vision)
- Encephalopathy, HIV-related
- Herpes simplex: chronic ulcers (>1 month's duration) or bronchitis, pneumonitis,
- or oesophagitis (onset at age >1 month)
- Histoplasmosis, disseminated or extrapulmonary
- Isosporiasis, chronic intestinal (>1 month's duration)
- Kaposi's sarcoma
- Lymphoma, Burkitt's (or equivalent term)
- Lymphoma, immunoblastic (or equivalent term)
- Lymphoma, primary, of brain
- Mycobacterium avium complex or Mycobacterium kansasii, disseminated or
- extrapulmonary
- Mycobacterium tuberculosis of any site, pulmonary, disseminated or
- extrapulmonary
- Mycobacterium, other species or unidentified species, disseminated or
- extrapulmonary
- Pneumocystis jirovecii pneumonia
- Pneumonia, recurrent
- Progressive multifocal leukoencephalopathy
- Salmonella septicaemia, recurrent
- Toxoplasmosis of brain, onset at age >1 month
- Wasting syndrome attributed to HIV.

Reference

CDC. Revised Surveillance Case Definition for HIV Infection United States, 2014. MMWR 2014; 63 (RR-03);1-10.

10.7. Appendix 7: Regulatory, Ethical, and Study Oversight Considerations

10.7.1. Regulatory and Ethical Considerations

Investigator Responsibilities

The investigator is responsible for ensuring that the study is performed in accordance with the protocol, current ICH guidelines on Good Clinical Practice (GCP), and applicable regulatory and country-specific requirements.

Good Clinical Practice is an international ethical and scientific quality standard for designing, conducting, recording, and reporting studies that involve the participation of human participants. Compliance with this standard provides public assurance that the rights, safety, and well-being of study participants are protected, consistent with the principles that originated in the Declaration of Helsinki, and that the study data are credible.

Protocol Amendments

Neither the investigator nor the sponsor will modify this protocol without a formal amendment by the sponsor. All protocol amendments must be issued by the sponsor, and signed and dated by the investigator. Protocol amendments must not be implemented without prior IEC/IRB approval, or when the relevant competent authority has raised any grounds for non-acceptance, except when necessary to eliminate immediate hazards to the participants, in which case the amendment must be promptly submitted to the IEC/IRB and relevant competent authority. Documentation of amendment approval by the investigator and IEC/IRB must be provided to the sponsor. When the change(s) involve only logistic or administrative aspects of the study, the IEC/IRB (where required) only needs to be notified.

During the course of the study, in situations where a departure from the protocol is unavoidable, the investigator or other physician in attendance will contact the appropriate sponsor representative listed in the Contact Information page(s), which will be provided as a separate document. Except in emergency situations, this contact should be made before implementing any departure from the protocol. In all cases, contact with the sponsor must be made as soon as possible to discuss the situation and agree on an appropriate course of action. The data recorded in the CRF and source documents will reflect any departure from the protocol, and the source documents will describe this departure and the circumstances requiring it.

In case of restrictions and limitations arising from a pandemic, study procedures and assessments may be executed as detailed in Appendix 13.

Regulatory Approval/Notification

This protocol and any amendment(s) must be submitted to the appropriate regulatory authorities in each respective country, if applicable. A study may not be initiated until all local regulatory requirements are met.

Required Prestudy Documentation

The following documents must be provided to the sponsor before shipment of study intervention to the study-site:

- Protocol and amendment(s), if any, signed and dated by the principal investigator.
- A copy of the dated and signed (or sealed, where appropriate per local regulations), written IEC/IRB approval of the protocol, amendments, ICF, any recruiting materials, and if applicable, participant compensation programs. This approval must clearly identify the specific protocol by title and number and must be signed (or sealed, where appropriate per local regulations) by the chairman or authorized designee.
- Name and address of the IEC/IRB, including a current list of the IEC/IRB members and their function, with a statement that it is organized and operates according to GCP and the applicable laws and regulations. If accompanied by a letter of explanation, or equivalent, from the IEC/IRB, a general statement may be substituted for this list. If an investigator or a member of the study-site personnel is a member of the IEC/IRB, documentation must be obtained to state that this person did not participate in the deliberations or in the vote/opinion of the study.
- Regulatory authority approval or notification, if applicable.
- Signed and dated statement of investigator (eg, Form FDA 1572), if applicable.
- Documentation of investigator qualifications (eg, curriculum vitae).
- Completed investigator financial disclosure form from the principal investigator, where required.
- Signed and dated Clinical Trial Agreement, which includes the financial agreement.
- Any other documentation required by local regulations.

The following documents must be provided to the sponsor before enrollment of the first participant:

- Completed investigator financial disclosure forms from all subinvestigators
- Documentation of subinvestigator qualifications (eg, curriculum vitae)
- Name and address of any local laboratory conducting tests for the study, and a dated copy of current laboratory normal ranges for these tests, if applicable
- Local laboratory documentation demonstrating competence and test reliability (eg, accreditation/license), if applicable

Independent Ethics Committee or Institutional Review Board

Before the start of the study, the investigator (or sponsor where required) will provide the IEC/IRB with current and complete copies of the following documents (as required by local regulations):

- Final protocol and, if applicable, amendments
- Sponsor-approved ICF (and any other written materials to be provided to the participants)

- IB (or equivalent information) and amendments/addenda
- Sponsor-approved participant recruiting materials
- Information on compensation for study-related injuries or payment to participants for participation in the study, if applicable
- Investigator's curriculum vitae or equivalent information (unless not required, as documented by the IEC/IRB)
- Information regarding funding, name of the sponsor, institutional affiliations, other potential conflicts of interest, and incentives for participants
- Any other documents that the IEC/IRB requests to fulfill its obligation

This study will be undertaken only after the IEC/IRB has given full approval of the final protocol, amendments (if any, excluding the ones that are purely administrative, with no consequences for participants, data or study conduct, unless required locally), the ICF, applicable recruiting materials, and participant compensation programs, and the sponsor has received a copy of this approval. This approval letter must be dated and must clearly identify the IEC/IRB and the documents being approved.

During the study the investigator (or sponsor where required) will send the following documents and updates to the IEC/IRB for their review and approval, where appropriate:

- Protocol amendments (excluding the ones that are purely administrative, with no consequences for participants, data or study conduct)
- Revision(s) to ICF and any other written materials to be provided to participants
- If applicable, new or revised participant recruiting materials approved by the sponsor
- Revisions to compensation for study-related injuries or payment to participants for participation in the study, if applicable
- New edition(s) of the IB and amendments/addenda
- Summaries of the status of the study at intervals stipulated in guidelines of the IEC/IRB (at least annually)
- Reports of AEs that are serious, unlisted/unexpected, and associated with the study intervention
- New information that may adversely affect the safety of the participants or the conduct of the study
- Deviations from or changes to the protocol to eliminate immediate hazards to the participants
- Report of deaths of participants under the investigator's care
- Notification if a new investigator is responsible for the study at the site
- Development Safety Update Report and Line Listings, where applicable
- Any other requirements of the IEC/IRB

For all protocol amendments (excluding the ones that are purely administrative, with no consequences for participants, data or study conduct), the amendment and applicable ICF revisions must be submitted promptly to the IEC/IRB for review and approval before implementation of the change(s).

At least once a year, the IEC/IRB will be asked to review and reapprove this study, where required.

At the end of the study, the investigator (or sponsor where required) will notify the IEC/IRB about the study completion (if applicable, the notification will be submitted through the head of investigational institution).

Other Ethical Considerations

For study-specific ethical design considerations, refer to Section 4.2.1.

10.7.2. Financial Disclosure

Investigators and subinvestigators will provide the sponsor with sufficient, accurate financial information in accordance with local regulations to allow the sponsor to submit complete and accurate financial certification or disclosure statements to the appropriate regulatory authorities. Investigators are responsible for providing information on financial interests during the course of the study and for 1 year after completion of the study.

Refer to Required Prestudy Documentation (above) and contracts for details on financial disclosure.

10.7.3. Informed Consent Process

Each participant must give written consent according to local requirements after the nature of the study has been fully explained. The ICF(s) must be signed before performance of any study-related activity. The ICF(s) that is/are used must be approved by both the sponsor and by the reviewing IEC/IRB and be in a language that the participant can read and understand. The informed consent should be in accordance with principles that originated in the Declaration of Helsinki, current ICH and GCP guidelines, applicable regulatory requirements, and sponsor policy.

Before enrollment in the study, the investigator or an authorized member of the study-site personnel must explain to potential participants the aims, methods, reasonably anticipated benefits, and potential hazards of the study, and any discomfort participation in the study may entail. Participants will be informed that their participation is voluntary and that they may withdraw consent to participate at any time. They will be informed that choosing not to participate will not affect the care the participant will receive for the treatment of his or her disease. Participants will be told that alternative treatments are available if they refuse to take part and that such refusal will not prejudice future treatment. Finally, they will be told that the investigator will maintain a participant identification register for the purposes of long-term follow-up if needed and that their records may be accessed by health authorities and authorized sponsor personnel without violating the confidentiality of the participant, to the extent permitted by the applicable law(s) or regulations. By signing the ICF the participant is authorizing such access, which includes permission to obtain

information about his or her survival status. It also denotes that the participant agrees to allow his or her study physician to recontact the participant for the purpose of obtaining consent for additional safety evaluations, and subsequent disease-related treatments, if needed. The physician may also recontact the participant for the purpose of obtaining consent to collect information about his or her survival status.

The participant will be given sufficient time to read the ICF and the opportunity to ask questions. After this explanation and before entry into the study, consent should be appropriately recorded by means of the participant's personally dated signature. After having obtained the consent, a copy of the ICF must be given to the participant. Participants undergoing DEXA scanning for measurement of body composition will provide separate informed consent.

Participants who are re-screened are required to sign a new ICF.

10.7.4. Data Protection

Privacy of Personal Data

The collection and processing of personal data from participants enrolled in this study will be limited to those data that are necessary to fulfill the objectives of the study.

These data must be collected and processed with adequate precautions to ensure confidentiality and compliance with applicable data privacy protection laws and regulations. Appropriate technical and organizational measures to protect the personal data against unauthorized disclosures or access, accidental or unlawful destruction, or accidental loss or alteration must be put in place. Sponsor personnel whose responsibilities require access to personal data agree to keep the identity of participants confidential.

The informed consent obtained from the participant includes explicit consent for the processing of personal data and for the investigator/institution to allow direct access to his or her original medical records (source data/documents) for study-related monitoring, audit, IEC/IRB review, and regulatory inspection. This consent also addresses the transfer of the data to other entities and to other countries.

The participant has the right to request through the investigator access to his or her personal data and the right to request rectification of any data that are not correct or complete. Reasonable steps will be taken to respond to such a request, taking into consideration the nature of the request, the conditions of the study, and the applicable laws and regulations.

10.7.5. Long-Term Retention of Samples for Additional Future Research

Samples collected in this study may be stored for up to 15 years (or according to local regulations) for additional research. Samples will only be used to understand CAB LA+RPV LA, to understand HIV-1 infection, to understand differential intervention responders, and to develop tests/assays related to CAB LA+RPV LA and HIV-1 infection. The research may begin at any time during the study or the post-study storage period.

Stored samples will be coded throughout the sample storage and analysis process and will not be labeled with personal identifiers. Participants may withdraw their consent for their samples to be stored for research.

10.7.6. Committees Structure

Independent Data Monitoring Committee

An IDMC will be established to review interim data. This committee will consist of at least one medical expert in the relevant therapeutic area and at least one statistician; committee membership responsibilities, authorities, and procedures will be documented in its charter. The committee will meet periodically to review interim data. After the review, the IDMC will make recommendations regarding the continuation of the study.

Publication Policy/Dissemination of Clinical Study Data

All information, including but not limited to information regarding CAB LA+RPV LA or the sponsor's operations (eg, patent application, formulas, manufacturing processes, basic scientific data, prior clinical data, formulation information) supplied by the sponsor to the investigator and not previously published, and any data, including research data, generated as a result of this study, are considered confidential and remain the sole property of the sponsor. The investigator agrees to maintain this information in confidence and use this information only to accomplish this study and will not use it for other purposes without the sponsor's prior written consent.

The investigator understands that the information developed in the study will be used by the sponsor in connection with the continued development of CAB LA+RPV LA, and thus may be disclosed as required to other clinical investigators or regulatory agencies. To permit the information derived from the clinical studies to be used, the investigator is obligated to provide the sponsor with all data obtained in the study.

The results of the study will be reported in a Clinical Study Report generated by the sponsor and will contain data from all study sites that participated in the study as per protocol. Recruitment performance or specific expertise related to the nature and the key assessment parameters of the study will be used to determine a coordinating investigator for the study. Results of analyses performed after the Clinical Study Report has been issued will be reported in a separate report and will not require a revision of the Clinical Study Report.

Study participant identifiers will not be used in publication of results. Any work created in connection with performance of the study and contained in the data that can benefit from copyright protection (except any publication by the investigator as provided for below) shall be the property of the sponsor as author and owner of copyright in such work.

Consistent with Good Publication Practices and International Committee of Medical Journal Editors (ICMJE) guidelines, the sponsor shall have the right to publish such primary (multicenter) data and information without approval from the investigator. The investigator has the right to publish study-site-specific data after the primary data are published. If an investigator wishes to publish information from the study, a copy of the manuscript must be provided to the sponsor for

review at least 60 days before submission for publication or presentation. Expedited reviews will be arranged for abstracts, poster presentations, or other materials. If requested by the sponsor in writing, the investigator will withhold such publication for up to an additional 60 days to allow for filing of a patent application. In the event that issues arise regarding scientific integrity or regulatory compliance, the sponsor will review these issues with the investigator. The sponsor will not mandate modifications to scientific content and does not have the right to suppress information. For multicenter study designs and substudy approaches, secondary results generally should not be published before the primary endpoints of a study have been published. Similarly, investigators will recognize the integrity of a multicenter study by not submitting for publication data derived from the individual study-site until the combined results from the completed study have been submitted for publication, within 18 months after the study end date, or the sponsor confirms there will be no multicenter study publication. Authorship of publications resulting from this study will be based on the guidelines on authorship, such as those described in the ICMJE Recommendations for the Conduct, Reporting, Editing and Publication of Scholarly Work in Medical Journals, which state that the named authors must have made a significant contribution to the conception or design of the work; or the acquisition, analysis, or interpretation of the data for the work; and drafted the work or revised it critically for important intellectual content; and given final approval of the version to be published; and agreed to be accountable for all aspects of the work in ensuring that questions related to the accuracy or integrity of any part of the work are appropriately investigated and resolved.

Registration of Clinical Studies and Disclosure of Results

The sponsor will register and disclose the existence of and the results of clinical studies as required by law. The disclosure of the final study results will be performed after the end of study in order to ensure the statistical analyses are relevant.

10.7.7. Data Quality Assurance

Data Quality Assurance/Quality Control

Quality tolerance limits (QTLs) will be predefined to identify systematic issues that can impact participant safety and/or reliability of study results. These predefined parameters will be monitored during the study and important deviations from the QTLs and remedial actions taken will be summarized in the Clinical Study Report.

Steps to be taken to ensure the accuracy and reliability of data include the selection of qualified investigators and appropriate study sites, review of protocol procedures with the investigator and study-site personnel before the study, and periodic monitoring visits by the sponsor. Written instructions will be provided for collection, handling, storage, and shipment of samples.

Guidelines for CRF completion will be provided and reviewed with study-site personnel before the start of the study.

The sponsor will review CRF for accuracy and completeness during on-site monitoring visits and after transmission to the sponsor; any discrepancies will be resolved with the investigator or

designee, as appropriate. After upload of the data into the study database they will be verified for accuracy and consistency with the data sources.

10.7.8. Case Report Form Completion

Case report forms are prepared and provided by the sponsor for each participant in electronic format. All data relating to the study must be recorded in CRF. All CRF entries, corrections, and alterations must be made by the investigator or authorized study-site personnel. The investigator must verify that all data entries in the CRF are accurate and correct.

The study data will be transcribed by study-site personnel from the source documents onto an electronic CRF, if applicable. Study-specific data will be transmitted in a secure manner to the sponsor.

Worksheets may be used for the capture of some data to facilitate completion of the CRF. Any such worksheets will become part of the participant's source documents. Data must be entered into CRF in English. The CRF must be completed as soon as possible after a participant visit and the forms should be available for review at the next scheduled monitoring visit.

All participative measurements (eg, pain scale information or other questionnaires) will be completed by the same individual who made the initial baseline determinations whenever possible.

If necessary, queries will be generated in the eDC tool. If corrections to a CRF are needed after the initial entry into the CRF, this can be done in either of the following ways:

- Investigator and study-site personnel can make corrections in the eDC tool at their own initiative or as a response to an auto query (generated by the eDC tool).
- Sponsor or sponsor delegate can generate a query for resolution by the investigator and study-site personnel.

10.7.9. Source Documents

At a minimum, source documents consistent in the type and level of detail with that commonly recorded at the study-site as a basis for standard medical care must be available for the following: participant identification, eligibility, and study identification; study discussion and date of signed informed consent; dates of visits; results of safety and efficacy parameters as required by the protocol; record of all AEs and follow-up of AEs; concomitant medication; intervention receipt/dispensing/return records; study intervention administration information; and date of study completion and reason for early discontinuation of study intervention or withdrawal from the study, if applicable.

The author of an entry in the source documents should be identifiable. Given that PROs are reports of a patient's health condition that come directly from the patient, without interpretation by a clinician or anyone else, the responses to PRO measures entered by trial participants into source records cannot be overridden by site-staff or investigators.

Specific details required as source data for the study and source data collection methods will be reviewed with the investigator before the study and will be described in the monitoring guidelines (or other equivalent document).

The following data will be recorded directly into the CRF and will be considered source data:

- Race
- Blood pressure and pulse/heart rate
- Height and weight
- Details of physical examination
- PRO and Health Economics data

The minimum source documentation requirements for Section 5.1, Inclusion Criteria and Section 5.2, Exclusion Criteria that specify a need for documented medical history are as follows:

- Complete history of medical notes at the site

Inclusion and exclusion criteria not requiring documented medical history must be verified at a minimum by participant interview or other protocol-required assessment (eg, physical examination, laboratory assessment) and documented in the source documents.

An eSource system may be utilized, which contains data traditionally maintained in a hospital or clinic record to document medical care (eg, electronic source documents) as well as the clinical study-specific data fields as determined by the protocol. This data is electronically extracted for use by the sponsor. If eSource is utilized, references made to the CRF in the protocol include the eSource system but information collected through eSource may not be limited to that found in the CRF.

10.7.10. Monitoring

The sponsor will use a combination of monitoring techniques central, remote, or on-site monitoring to monitor this study.

The sponsor will perform on-site monitoring visits as frequently as necessary. The monitor will record dates of the visits in a study-site visit log that will be kept at the study-site. The first post-initiation visit will be made as soon as possible after enrollment has begun. At these visits, the monitor will compare the data entered into the CRF with the source documents (eg, hospital/clinic/physician's office medical records). The nature and location of all source documents will be identified to ensure that all sources of original data required to complete the CRF are known to the sponsor and study-site personnel and are accessible for verification by the sponsor study-site contact. If electronic records are maintained at the study-site, the method of verification must be discussed with the study-site personnel.

Direct access to source documents (medical records) must be allowed for the purpose of verifying that the recorded data are consistent with the original source data. Findings from this review will be discussed with the study-site personnel. The sponsor expects that, during monitoring visits, the

relevant study-site personnel will be available, the source documents will be accessible, and a suitable environment will be provided for review of study-related documents. The monitor will meet with the investigator on a regular basis during the study to provide feedback on the study conduct.

In addition to on-site monitoring visits, remote contacts can occur. It is expected that during these remote contacts, study-site personnel will be available to provide an update on the progress of the study at the site.

Central monitoring will take place for data identified by the sponsor as requiring central review.

10.7.11. On-Site Audits

Representatives of the sponsor's clinical quality assurance department may visit the study-site at any time during or after completion of the study to conduct an audit of the study in compliance with regulatory guidelines and company policy. These audits will require access to all study records, including source documents, for inspection. Participant privacy must, however, be respected. The investigator and study-site personnel are responsible for being present and available for consultation during routinely scheduled study-site audit visits conducted by the sponsor or its designees.

Similar auditing procedures may also be conducted by agents of any regulatory body, either as part of a national GCP compliance program or to review the results of this study in support of a regulatory submission. The investigator should immediately notify the sponsor if he or she has been contacted by a regulatory agency concerning an upcoming inspection.

10.7.12. Record Retention

In compliance with the ICH/GCP guidelines, the investigator/institution will maintain all CRF and all source documents that support the data collected from each participant, as well as all study documents as specified in ICH/GCP Section 8, Essential Documents for the Conduct of a Clinical Trial, and all study documents as specified by the applicable regulatory requirement(s). The investigator/institution will take measures to prevent accidental or premature destruction of these documents.

Essential documents must be retained until at least 2 years after the last approval of a marketing application in an ICH region and until there are no pending or contemplated marketing applications in an ICH region or until at least 2 years have elapsed since the formal discontinuation of clinical development of the investigational product. These documents will be retained for a longer period if required by the applicable regulatory requirements or by an agreement with the sponsor. It is the responsibility of the sponsor to inform the investigator/institution as to when these documents no longer need to be retained.

If the responsible investigator retires, relocates, or for other reasons withdraws from the responsibility of keeping the study records, custody must be transferred to a person who will accept the responsibility. The sponsor must be notified in writing of the name and address of the new

custodian. Under no circumstance shall the investigator relocate or dispose of any study documents before having obtained written approval from the sponsor.

If it becomes necessary for the sponsor or the appropriate regulatory authority to review any documentation relating to this study, the investigator/institution must permit access to such reports.

10.7.13. Study and Site Start and Closure

First Act of Recruitment

The first site open is considered the first act of recruitment and it becomes the study start date.

Study/Site Termination

The sponsor reserves the right to close the study-site or terminate the study at any time for any reason at the sole discretion of the sponsor. Study sites will be closed upon study completion. A study-site is considered closed when all required documents and study supplies have been collected and a study-site closure visit has been performed.

The investigator may initiate study-site closure at any time, provided there is reasonable cause and sufficient notice is given in advance of the intended termination.

Reasons for the early closure of a study-site by the sponsor or investigator may include but are not limited to:

- Failure of the investigator to comply with the protocol, the requirements of the IEC/IRB or local health authorities, the sponsor's procedures, or GCP guidelines
- Inadequate recruitment of participants by the investigator
- Discontinuation of further study intervention development

10.8. Appendix 8: Adverse Events, Serious Adverse Events, Product Quality Complaints, and Other Safety Reporting: Definitions and Procedures for Recording, Evaluating, Follow-up, and Reporting

10.8.1. Adverse Event Definitions and Classifications

Adverse Event

An adverse event is any untoward medical occurrence in a clinical study participant administered a pharmaceutical (investigational or non-investigational) product. An adverse event does not necessarily have a causal relationship with the intervention. An adverse event can therefore be any unfavorable and unintended sign (including an abnormal finding), symptom, or disease temporally associated with the use of a medicinal (investigational or non-investigational) product, whether or not related to that medicinal (investigational or non-investigational) product. (Definition per International Council on Harmonisation [ICH])

This includes any occurrence that is new in onset or aggravated in severity or frequency from the baseline condition, or abnormal results of diagnostic procedures, including laboratory test abnormalities.

Note: The sponsor collects AEs starting with the signing of the ICF (refer to All Adverse Events under Section 8.3.1, Time Period and Frequency for Collecting Adverse Events and Serious Adverse Events Information, for time of last adverse event recording).

Serious Adverse Event

A serious adverse event based on ICH and EU Guidelines on Pharmacovigilance for Medicinal Products for Human Use is any untoward medical occurrence that at any dose:

- Results in death
- Is life-threatening
  (The participant was at risk of death at the time of the event. It does not refer to an event that hypothetically might have caused death if it were more severe)
- Requires inpatient hospitalization or prolongation of existing hospitalization
- Results in persistent or significant disability/incapacity
- Is a congenital anomaly/birth defect
- Is a suspected transmission of any infectious agent via a medicinal product
- Is Medically Important*

*Medical and scientific judgment should be exercised in deciding whether expedited reporting is also appropriate in other situations, such as important medical events that may not be immediately life-threatening or result in death or hospitalization but may jeopardize the participant or may require intervention to prevent one of the other outcomes listed in the definition above. These should usually be considered serious.

If a serious and unexpected adverse event occurs for which there is evidence suggesting a causal relationship between the study intervention and the event (eg, death from anaphylaxis), the event must be reported as a serious and unexpected suspected adverse reaction even if it is a component of the study endpoint (eg, all-cause mortality).

Unlisted (Unexpected) Adverse Event/Reference Safety Information

An adverse event is considered unlisted if the nature or severity is not consistent with the applicable product reference safety information. For CAB LA+RPV LA, the expectedness of an adverse event will be determined by whether or not it is listed in the IB. For cART with a marketing authorization, the expectedness of an adverse event will be determined by whether or not it is listed in the SmPC.

10.8.2. Attribution Definitions

Assessment of Causality

The causal relationship to study intervention is determined by the Investigator. The following selection should be used to assess all AEs.

Related

There is a reasonable causal relationship between study intervention administration and the AE.

Not Related

There is not a reasonable causal relationship between study intervention administration and the AE.

The term "reasonable causal relationship" means there is evidence to support a causal relationship.

10.8.3. Severity Criteria

An assessment of severity grade will be made using the following general categorical descriptors:

Mild: Awareness of symptoms that are easily tolerated, causing minimal discomfort and not interfering with everyday activities.

Moderate: Sufficient discomfort is present to cause interference with normal activity.

Severe: Extreme distress, causing significant impairment of functioning or incapacitation. Prevents normal everyday activities.

The investigator should use clinical judgment in assessing the severity of events not directly experienced by the participant (eg, laboratory abnormalities).

10.8.4. Special Reporting Situations

Safety events of interest on a sponsor study intervention in an interventional study that may require expedited reporting or safety evaluation include, but are not limited to:

- Overdose of a sponsor study intervention

- Suspected abuse/misuse of a sponsor study intervention
- Accidental or occupational exposure to a sponsor study intervention
- Medication error, intercepted medication error, or potential medication error involving a Johnson & Johnson medicinal product (with or without patient exposure to the Johnson & Johnson medicinal product, eg, product name confusion, product label confusion, intercepted prescribing or dispensing errors)
- Exposure to a sponsor study intervention from breastfeeding

Special reporting situations should be recorded in the CRF. Any special reporting situation that meets the criteria of a serious adverse event should be recorded on the serious adverse event page of the CRF.

10.8.5. Procedures

All Adverse Events

All AEs, regardless of seriousness, severity, or presumed relationship to study intervention, must be recorded using medical terminology in the source document and the CRF. Whenever possible, diagnoses should be given when signs and symptoms are due to a common etiology (eg, cough, runny nose, sneezing, sore throat, and head congestion should be reported as "upper respiratory infection"). Investigators must record in the CRF their opinion concerning the relationship of the adverse event to study therapy. All measures required for adverse event management must be recorded in the source document and reported according to sponsor instructions.

For all studies with an outpatient phase, including open-label studies, the participant must be provided with a "wallet (study) card" and instructed to carry this card with them for the duration of the study indicating the following:

- Study number
- Statement, in the local language(s), that the participant is participating in a clinical study
- Investigator's name and 24-hour contact telephone number
- Local sponsor's name and 24-hour contact telephone number (for medical personnel only)
- Site number
- Participant number
- Any other information that is required to do an emergency breaking of the blind

Serious Adverse Events

All SAEs that have not resolved by the end of the study, or that have not resolved upon the participant's discontinuation from the study, must be followed until any of the following occurs:

- The event resolves
- The event stabilizes

- The event returns to baseline, if a baseline value/status is available
- The event can be attributed to agents other than the study intervention or to factors unrelated to study conduct
- It becomes unlikely that any additional information can be obtained (participant or health care practitioner refusal to provide additional information, lost to follow-up after demonstration of due diligence with follow-up efforts)

Any event requiring hospitalization (or prolongation of hospitalization) that occurs during participation in the study must be reported as a serious adverse event, except hospitalizations for the following:

- Hospitalizations not intended to treat an acute illness or adverse event (eg, social reasons such as pending placement in long-term care facility).
- Surgery or procedure planned before entry into the study (must be documented in the CRF). Note: Hospitalizations that were planned before the signing of the ICF, and where the underlying condition for which the hospitalization was planned has not worsened, will not be considered SAEs. Any adverse event that results in a prolongation of the originally planned hospitalization is to be reported as a new serious adverse event.
- For convenience the investigator may choose to hospitalize the participant for the duration of the intervention period.

The cause of death of a participant in a study within 24 hours of the last dose of study intervention, whether or not the event is expected or associated with the study intervention, is considered a serious adverse event.

Information regarding SAEs will be transmitted to the sponsor using a serious adverse event reporting form and safety report form of the CRF, which must be completed and reviewed by a physician from the study-site, and transmitted in a secure manner to the sponsor within 24 hours. The initial and follow-up reports of a serious adverse event should be transmitted in a secure manner electronically or by facsimile (fax). Telephone reporting should be the exception and the reporter should be asked to complete the appropriate form(s) first.

10.8.6. Product Quality Complaint Handling

Definition

A PQC is defined as any suspicion of a product defect related to manufacturing, labeling, or packaging, ie, any dissatisfaction relative to the identity, quality, durability, reliability, or performance of a distributed product, including its labeling, drug delivery system, or package integrity. A PQC may have an impact on the safety and efficacy of the product. In addition, it includes any technical complaints, defined as any complaint that indicates a potential quality issue during manufacturing, packaging, release testing, stability monitoring, dose preparation, storage or distribution of the product or the drug delivery system.

Procedures

All initial PQCs must be reported to the sponsor by the study-site personnel within 24 hours after being made aware of the event.

A sample of the suspected product should be maintained under the correct storage conditions until a shipment request is received from the sponsor.

10.8.7. Contacting Sponsor Regarding Safety, Including Product Quality

The names (and corresponding telephone numbers) of the individuals who should be contacted regarding safety issues, PQC, or questions regarding the study are listed in the Contact Information page(s), which will be provided as a separate document.

10.9. Appendix 9: Contraceptive and Barrier Guidance

Participants must follow contraceptive measures as outlined in Section 5.1, Inclusion Criteria. Pregnancy information will be collected and reported as noted in Section 8.3.5, Pregnancy and Appendix 8 Adverse Events, Serious Adverse Events, Product Quality Complaints, and Other Safety Reporting: Definitions and Procedures for Recording, Evaluating, Follow-up, and Reporting.

Definitions

Woman of Childbearing Potential (WOCBP)

A woman is considered fertile following menarche and until becoming postmenopausal unless permanently sterile (see below).

Woman Not of Childbearing Potential

- **premenarchal**
  A premenarchal state is one in which menarche has not yet occurred.
- **postmenopausal**
  A postmenopausal state is defined as no menses for 12 months without an alternative medical cause. A high FSH level (>40 IU/L or mIU/mL) in the postmenopausal range may be used to confirm a postmenopausal state in women not using hormonal contraception or HRT, however in the absence of 12 months of amenorrhea, a single FSH measurement is insufficient. If there is a question about menopausal status in women on HRT, the woman will be required to use one of the non-estrogen-containing hormonal highly effective contraceptive methods if she wishes to continue HRT during the study.
- **permanently sterile (for the purpose of this study)**
  Permanent sterilization methods include hysterectomy, bilateral salpingectomy, and bilateral oophorectomy.

Note: If the childbearing potential changes after start of the study (eg, a premenarchal woman experiences menarche) or the risk of pregnancy changes (eg, a woman who is not heterosexually active becomes active), a woman must begin a highly effective method of contraception, as described throughout the inclusion criteria.

If reproductive status is questionable, additional evaluation should be considered.

Contraceptive (birth control) use by men or women should be consistent with local regulations regarding the acceptable methods of contraception for those participating in clinical studies.

Typical use failure rates may differ from those when used consistently and correctly. Use should be consistent with local regulations regarding the use of contraceptive methods for participants in clinical studies.

Examples of Contraceptives

EXAMPLES OF CONTRACEPTIVES[a] ALLOWED DURING THE STUDY INCLUDE:
**USER INDEPENDENT**<br>**Highly Effective Methods That Are User Independent** *Failure rate of <1% per year when used consistently and correctly.*
• Implantable progestogen-only hormone contraception associated with inhibition of ovulation[b]
• Intrauterine device (IUD)
• Intrauterine hormone-releasing system (IUS)
• Bilateral tubal occlusion
• Azoospermic partner *(vasectomized or due to medical cause)*<br>*(Vasectomized partner is a highly effective contraceptive method provided that the partner is the sole sexual partner of the woman of childbearing potential and the absence of sperm has been confirmed. If not, additional highly effective method of contraception should be used. Spermatogenesis cycle is approximately 74 days.)*
**USER DEPENDENT**<br>**Highly Effective Methods That Are User Dependent** *Failure rate of <1% per year when used consistently and correctly.*
• Combined (estrogen- and progestogen-containing) hormonal contraception associated with inhibition of ovulation[b]<br>– oral<br>– intravaginal<br>– transdermal<br>– injectable
• Progestogen-only hormone contraception associated with inhibition of ovulation[b]<br>– oral<br>– injectable
• Sexual abstinence<br>*(Sexual abstinence is considered a highly effective method only if defined as refraining from heterosexual intercourse during the entire period of risk associated with the study intervention. The reliability of sexual abstinence needs to be evaluated in relation to the duration of the study and the preferred and usual lifestyle of the participant.)*
**NOT ALLOWED AS SOLE METHOD OF CONTRACEPTION DURING THE STUDY (not considered to be highly effective - failure rate of ≥1% per year)**
• Progestogen-only oral hormonal contraception where inhibition of ovulation is not the primary mode of action.
• Male or female condom
• Cap, diaphragm, or sponge with spermicide
• A combination of male condom with either cap, diaphragm, or sponge (double-barrier methods)[c]
• Periodic abstinence (calendar, symptothermal, post-ovulation methods)
• Withdrawal (coitus-interruptus)
• Spermicides alone
• Lactational amenorrhea method (LAM)

a) Typical use failure rates may differ from those when used consistently and correctly. Use should be consistent with local regulations regarding the use of contraceptive methods for participants in clinical studies.
b) Hormonal contraception may be susceptible to interaction with the study intervention, which may reduce the efficacy of the contraceptive method. In addition, consider if the hormonal contraception may interact with the study intervention.
c) Male condom and female condom should not be used together (due to risk of failure with friction).

10.10. Appendix 10: EuroQoL-5D-5L Health Questionnaire (EQ-5D-5L) – Sample UK English Version

EQ-5D-5L

Sample

Health Questionnai[illegible]

English version for [illegible] UK

UK (English) © 2009 EuroQol Group EQ-5D™ is a trade mark of the EuroQol Group

CCI

[redacted]

2

UK (English) © 2009 EuroQol Group EQ-5D™ is a trade mark of the EuroQol Group

CCI

[redacted]

3

UK (English) © 2009 EuroQol Group EQ-5D™ is a trade mark of the EuroQol Group

10.11. Appendix 11: MOS-HIV – 35 Item Instrument

MOS-HIV 35-ITEM INSTRUMENT

CCI

[redacted]

CCI

[redacted]

CCI

[redacted]

CCI

[redacted]

CCI

[redacted]

CCI

[redacted]

10.12. Appendix 12: HIV Treatment Satisfaction Questionnaire

HIV Treatment Satisfaction Questionnaire (HIVTSQs)

CCI

[redacted]

10.13. Appendix 13: Guidance on Study Conduct During a Pandemic

It is recognized that a pandemic situation (eg, Coronavirus Disease 2019 [COVID-19], or other) may have an impact on the conduct of this clinical study due to, for example: self-isolation/quarantine by participants and/or study-site personnel; travel restrictions and/or limited access to public places, including hospitals; and study-site personnel being reassigned to critical tasks.

In alignment with health authority guidance, the Sponsor is providing options for study-related patient management in the event of disruption to the conduct of the study. These recommendations do not supersede any local or government requirements or the clinical judgment of the investigator to protect the health and well-being of participants and site-staff. If, at any time, a participant's safety is considered to be at risk, study intervention will be discontinued, and follow-up will be conducted.

GENERAL GUIDANCE

Scheduled visits that cannot be conducted in person at a study-site will be performed remotely/virtually to the extent possible or may be delayed until such time that on-site visits can be resumed. At each contact, participants will be interviewed to collect safety data. Participants will also be questioned regarding their general health status.

Every effort should be made to adhere to protocol-specified assessments for participants on study intervention, including follow-up. Modifications to protocol-required assessments may be permitted after consultation between the participant and investigator, and with the agreement of the Sponsor. Missed assessments/visits will be captured in the clinical trial management system for protocol deviations. Discontinuations of study interventions and withdrawal from the study should be documented with the appropriate prefix (eg, "COVID-19-related" or other in accordance with Sponsor recommendations) in the case report form.

The Sponsor will continue to monitor the conduct and progress of the clinical study, and any changes will be communicated to the sites and to the health authorities according to local guidance. If a participant has tested positive for a pandemic infection, the investigator should contact the Sponsor's responsible medical officer to discuss plans for study intervention and follow-up. Modifications made to the study conduct as a result of the pandemic should be summarized in the Clinical Study Report.

GUIDANCE SPECIFIC TO THIS PROTOCOL:

These emergency provisions are meant to ensure the safety of participants on study while site capabilities are compromised by pandemic restrictions. As restrictions are lifted, sites should revert to original protocol conduct as soon as feasible.

Enrollment of new participants may be halted at the Sponsor's discretion and restarted once the situation permits. The investigator's assessment of the risks versus benefit, depending on the situation at their particular site and the ability to monitor the safety of participants must be considered when restarting enrollment at a specific site.

The following provisions are being implemented by the Sponsor with respect to the following protocol-mandated procedures to mitigate the impact of a pandemic:

1. Study intervention
2. Management of discontinuations and withdrawals due to a pandemic
3. Options for efficacy and other assessments
4. Options for safety assessments
5. Handling of missed assessments, visits or doses
6. Management of participants with COVID-19 infection
7. Statistical considerations

1. ***Study Intervention***

a. Study Intervention Administration: Home-administration

CAB LA+RPV LA and cART will be dispensed at the study-site throughout the study, where possible. The study interventions should be continued if, in the assessment of the investigator, it does not result in risk to the participant. In cases where a site visit is not possible under the restrictions and limitations due to the COVID-19 pandemic, alternatives to study medication dispensing, administration, and clinical safety laboratory assessments (including home health nursing) will allow continued study participation for participants in this study. In cases where site visits are not possible under the restrictions and limitations due to a pandemic, the investigator after authorization by the lead scientist/chief investigator, may permit the use of daily oral RPV 25mg and CAB 30mg as a "bridging" strategy for participants who have begun CAB LA+RPV LA. In case of insufficient availability of oral RPV and oral CAB for the bridging strategy, the lead scientist/chief investigator may permit the use of SOC antiretroviral therapy per local country guidelines for participants in the CAB LA+RPV LA group, till the time site visits are resumed or sufficient oral RPV 25mg and CAB 30mg doses are procured. Oral RPV and oral CAB may be delivered to the subjects via direct to patient (DTP) shipments. Provision and supply of medication to the cART group will be managed by the national program.

b. Notes on Shipment of Study Intervention to Participants

If it is necessary to ship the study intervention directly to study participants, shipment by the study-site itself is preferred under this exception due to the COVID-19 pandemic. Shipment should be made in a manner that allows tracking of both transport and delivery.

The study intervention must be delivered directly to the participant or a person authorized by the participant, and must not be given to neighbors or deposited at a storage location. Written confirmation of dose and dose regimens by the investigator should also be obtained prior to shipment.

For direct shipment of study intervention to participants, written instructions on storage and return of used and unused study intervention should be provided to participants. When shipped by study sites, the receipt, consumption and return of study intervention must be documented in a form that

allows the study-site to meet its documentation requirements (ie, drug accountability), as defined in ICH GCP 4.6.3.

c. Treatment Compliance

Participants will be instructed to bring their original containers, whether empty or containing the oral study treatment with them at each visit. During the course of the study, the investigator or designated study research staff will be responsible for providing additional instruction to reeducate any participant who is not compliant with taking the oral treatment.

The investigator or designated study personnel will maintain a log of all medications dispensed and returned. Supplies for each participant will be inventoried and accounted for throughout the study. Treatment compliance (adherence) will be assessed by tablet count as documented on the drug accountability form.

Participants who receive DTP shipments of the study treatment due to COVID-19 restrictions will also receive detailed instructions for storage, administration, and handling of unused study medications. Treatment supplies for each subject will be inventoried and accounted for. Adherence counseling will be administered regarding the importance of maintaining the cART or oral RPV+CAB regimen. Treatment compliance will be assessed for all study participants at each study visit, or via telephone as applicable, if visits cannot be performed.

d. Discontinuation of Study Intervention

If at any time the safety of a participant is considered to be at risk, study treatment will be temporarily or permanently discontinued, while every effort should be made to maintain follow-up on study.

The benefit of continuing study treatment should be considered by the investigator for each individual participant, considering the potential impact of reduced direct clinical supervision on a participant's safety. Discontinuations of study interventions due to pandemic-related reasons should be documented with a prefix (eg, "COVID-19-related" or in accordance with Sponsor recommendations) in the CRF.

2. Management of Discontinuations and Withdrawals due to a Pandemic

Withdrawal from the study due to pandemic-related reasons should be documented with a prefix (eg, "COVID-19-related" or in accordance with Sponsor recommendations) in the CRF. Study intervention assigned to the withdrawn participant may not be assigned to another participant. If the sample size is not achieved due to pandemic-related reasons or other emergency situations, enrollment of additional participants will be considered by the Sponsor.

3. Options for Efficacy and Other Assessments

HIV-1 RNA testing will be carried out at the time-points detailed in the protocol. The HIV-1 RNA testing may also be performed using home collection of samples or at a local laboratory. Missing

visits and missing laboratory assessments will be documented as protocol deviations with the prefix "COVID-19-related".

To safely maintain participants on study treatment while site capabilities are compromised by pandemic or other emergency restrictions, participants may have home health nursing visits until such time that on-site visits can be resumed. Normal study procedures should be followed for the applicable visit as closely as possible.

Patient-reported outcomes (PRO) questionnaires will be deferred till the next site visit. Actual dates of assessments will be recorded and transcribed to the CRF. Missed assessments/visits will be captured in the clinical trial management system for protocol deviations.

If assessments are not possible as outlined in the Schedule of Activities, missed assessments should be documented as a deviation related to the pandemic and be made up as soon as possible.

4. Options for Safety Assessments

All efforts should be done to perform the following safety assessments, when applicable:

- Vital signs
- Weight
- Urine pregnancy testing
- Safety assessments related to study intervention administration
- Clinical Safety Laboratory Assessments (including through home collection of samples or at certified local laboratory, where possible)

Home testing may be employed for urine pregnancy tests. The urine pregnancy test will be shipped in advance to the participants. For women of childbearing potential, a negative pregnancy test must be recorded at the time-points detailed in the instructions provided by the site-staff.

Clinical laboratory safety monitoring may be performed using home collection of samples or at a local laboratory. A copy of the external laboratory report should be reviewed by the investigator and retained, along with reference ranges, for source documentation and captured in the CRF.

5. Handling of Missed Assessments/Visits/Doses

Relevant study data elements impacted by a pandemic or other emergency situations should be documented (eg, "COVID-19-related" or in accordance with Sponsor recommendations) in CRFs and/or other study systems, as directed by Sponsor guidance; these may include missed/delayed/modified study visits/assessments, and instances where temporary measures such as those above are implemented.

6. Management of Participants Infected With SARS-CoV-2

If a participant develops SARS-CoV-2 infection or related disease, the investigator should contact the Sponsor to discuss plans for study intervention and follow-up. CAB LA+RPV LA or cART may be interrupted at the discretion of the investigator, depending on symptoms and concomitant

medication used for the treatment of COVID-19. Treatment must be interrupted if prohibited medication is used. Standard adverse event and serious adverse event reporting requirements apply.

If one or more antiretroviral medications is held due to toxicity or AEs, all antiretroviral medications must be held to reduce the risk of development of resistance taking into account both the length of the planned interruption and the pharmacokinetic half-life of each antiretroviral of the regimen, in a way to minimize the risk of development of resistance.

All decisions regarding dose interruption / resumption must be discussed with the scientific lead and chief investigator in advance. Consenting and re-consenting of participants will be performed as applicable for the measures taken and according to local guidance for informed consent applicable during the COVID-19 pandemic.

7. *Statistical Considerations*

Statistical analysis will be done under the authority of the Sponsor. A general description of the statistical methods to be used to analyze the efficacy and safety data is detailed in the protocol.

The Sponsor will evaluate the totality of impact of the restrictions and limitations arising from a pandemic on collection of key study data and additional data analyses will be outlined in the Statistical Analysis Plan(s).

The sample size may be further revised to compensate for major protocol deviations during a pandemic which could potentially impact the primary endpoint. The new sample size will also account for any future dropouts.

Note: In case of emergency situations where the recommendations specified in this protocol are insufficient, the investigator or site-staff are instructed to follow local guidelines and recommendations, or contact the Sponsor for guidance.

10.14. Appendix 14: Protocol Amendment History

This is Amendment 3 to the original protocol. The Protocol Amendment Summary of Changes Table for the current amendment is located directly before the Table of Contents (TOC).

Amendment 2 (16 June 2023)

Overall Rationale for the Amendment: To provide clarity on the timepoints used in the protocol and details on continued access to the study intervention following the end of the study.

The changes made to the clinical protocol TMC278LAHTX3002 as part of Protocol Amendment 2 are listed below, including the rationale of each change and a list of all applicable sections. Changes made in previous protocol amendments are listed in Section 10.14, Appendix 14: Protocol Amendment History.

**Section Number and Name**	**Description of Change**	**Brief Rationale**
Synopsis<br>Overall Design;<br>1.3 Schedule of Activities<br>4 Study Design	A footnote was added to Schedule of Activities. "Note: Throughout the protocol, visits and analysis timepoints expressed in months from baseline are interpreted as multiples of 4-weeks from baseline ie, Month 12 is interpreted as Week 48 and Month 24 is interpreted as Week 96 from baseline. "	To clarify that timepoints expressed in months throughout the protocol are to be interpreted as multiples of 4-weeks.
Synopsis<br>Intervention Groups and Duration;<br>6.6 Continued Access to Study Intervention After the End of the Study	The following text was added:-<br>"At the end of their participation in the study, participants from the CAB LA + RPV LA group who have completed the study and continue to benefit clinically from the study intervention, as determined by their investigator and are willing to continue treatment, will receive continued access to CAB LA and RPV LA for up to 4 years after study completion. Participants will continue to receive study intervention until:-<br>• the participant no longer derives clinical benefit;<br>• the participant meets a protocol-defined reason for discontinuation;<br>• CAB LA and RPV LA are registered and reimbursed in the country; or<br>• either CAB LA or RPV LA development program is terminated; whichever occurs earlier."<br>This continued access will be provided through a post-trial access (PTA) program. If this program is not operational at the end of the study, participants may continue to receive CAB LA and RPV LA via the study in order to avoid treatment interruption. The continued access to CAB LA and RPV LA within the study will serve as a bridge to the operationalization of the post-trial access program. During this bridging, participants will continue to receive care at respective study sites.	To provide clarity on continued access to study intervention after the end of the study.

Section Number and Name	Description of Change	Brief Rationale
	Under the PTA program, Janssen will continue to provide access to CAB LA and RPV LA until patients can obtain the study intervention outside the post-trial access program or through commercial access within local healthcare systems per local/national regulations. Post-trial access will continue for up to four years, after which an evaluation of available local access options will be performed and access determined on a case-by-case basis, based on patient needs and available access options. For the purpose of this study, 'commercial access' means after approval by the competent national authorities for reimbursement under the national health insurance or comparable third-party payment programs.	

Amendment 1 (11 July 2022)

Overall Rationale for the Amendment: The protocol was amended to add additional exploratory endpoints to determine the prevalence of hepatitis B surface antibody (anti-HBs) positivity and hepatitis B virus (HBV) deoxyribonucleic acid (DNA) among the study participants, and to provide the background and rationale on the same.

Other changes included modifications to the inclusion and exclusion criteria and Schedule of Activities (SoA).

The changes made to the clinical protocol TMC278LAHTX3002 as part of Protocol Amendment 1 are listed below, including the rationale of each change and a list of all applicable sections.

Section Number and Name	Description of Change	Brief Rationale
1.1 Synopsis<br>3 Objectives and Endpoints	The following exploratory objectives were added:<br>• *To determine the prevalence HBV DNA among participants on stable combination antiretroviral therapy (cART) containing nucleoside reverse transcriptase inhibitors (NRTI) who test negative for HBsAg and positive for hepatitis B core antibodies (anti HBc) at screening.*<br>• *To determine the prevalence of anti HBs positivity among participants on stable cART containing NRTI who test negative for hepatitis B surface antigen (HBsAg) and positive for anti HBc at screening.*<br>The following exploratory endpoints were added:<br>• *Proportion of participants who test negative for HBsAg and positive for anti HBc with a detectable HBV viral load at screening.*<br>• *Proportion of participants at screening who test positive for anti HBs among those who test negative for HBsAg and positive for anti HBc at screening.*	To assess the prevalence of occult hepatitis B infection among participants screened in the study.
Section 1.3 Schedule of Activities	The following assessments were added to the SoA along with the corresponding footnotes:<br>• Concomitant medication at Month 2<br>• Urine pregnancy test at Screening<br>• Plasma storage at Screening: Footnote 'k' was added for plasma storage at screening: Plasma stored (4ml EDTA) at screening for batched testing for HBV DNA.<br>• Serum storage at Screening: Footnote 'l' was added: serum stored (4ml redtop) at screening for batched testing for anti-HBs.<br>• The HIV Treatment Satisfaction Questionnaire - Change (HIVTSc) was added to the table.<br>• Medical resource utilization survey was added at Month 12.	To capture assessments that were omitted in the Schedule of Activities.
2.3.4 Significance of anti-HBc Positivity Among Screen Failures	A new section was added to discuss the rationale for the additional HBV assessments in participants positive for anti-HBc at screening and the potential impact on anti-HBc positive participants treated with CAB+RPV.	To provide context and rationale for the testing of screening plasma samples for HBV.
8.5 Additional Stored Blood Samples for Characterization of HBV Profile in Screen Failures Based on HBV Serology	A new section was added to detail to provide a rationale for additional exploration of HBV profile, and storage procedures for plasma and serum samples from participants who test negative for HBsAg and positive for anti-HBc at screening.	To explore the proportion of participants who harbor HBV DNA, and who are at risk of

Section Number and Name	Description of Change	Brief Rationale
		HBV reactivation and hepatic flares.
5.4 Screen Failures	The following text was added to the section:<br>*Screen failures who tested negative for HBsAg but positive for anti HBc, and did not have a sample stored at screening, will be invited for a repeat blood sampling for the testing of HBV DNA and anti HBs. Informed consent will be obtained from these participants for the repeat blood sampling.*	In case plasma samples from screen failures were discarded by the study sites, participants will be re-invited for additional samples.
5.1 Inclusion Criteria	Inclusion criterion 1: modified to include 'males or females as determined at birth'.	To provide clarity in populations where there are more than two gender options, or where gender at birth may differ from gender assigned later in life.
	Inclusion criterion 5: modified from '*confirmed plasma HIV 1 RNA measurement >200 c/mL at any time*'<br>to<br>'*two consecutive plasma HIV 1 RNA measurements >200 c/mL at any time*'.	To provide clarity on the definition of virologic failure.
	Inclusion criterion 7: the definitions of non-reproductive potential was modified to pre-menopausal women with a *history of* tubal ligation, stereoscopic tubal occlusion procedure with follow-up confirmation of bilateral tubal occlusion, hysterectomy or bilateral oophorectomy.	Documentation of tubal ligation is rarely available from participants. Given the importance of including female participants in the trial, the decision was taken to accept a history of these procedures for the purposes of trial eligibility, and to prevent the exclusion of otherwise eligible participants.
5.2 Exclusion criteria	Exclusion criterion 7: modified from '*more than one seizure within one year….*' to:<br>*Either:*<br>*Has had one or more seizures within one year (defined as within 365 days) prior to entry, or unstable or poorly controlled seizure disorder, as determined by the investigator or designee, based on available medical records*<br>*or*<br>*Participants determined by the Investigator to have a high risk of seizures, including participants with an unstable or poorly controlled seizure disorder. A participant with a prior history of seizure may be considered for enrolment if the Investigator believes the risk of seizure recurrence is low."*	To exclude participants with poorly controlled seizures, as even one seizure a year may be considered as poor control.
6.8.3 Prohibited Medications with CAB and/or RPV	A note was added to proton pump inhibitors, such as esomeprazole, lansoprazole, omeprazole, pantoprazole, rabeprazole.	To align the protocol with the information in

Section Number and Name	Description of Change	Brief Rationale
	Must not be administered during oral intake of CAB or RPV; no contraindication for injectable formulation.	the Investigator Brochure.
1.1 Synopsis<br>1.3 Schedule of Activities<br>8.1.4 Patient-reported Outcomes	A treatment preference question was added to the SoA along with the timepoints of the assessments.<br>Further details were added to the patient-reported outcomes section:<br>*To understand the choice and lived experiences of participants randomized to the CAB LA+RPV LA arm receiving OLI versus those directly transitioning to injectable CAB LA+RPV, in depth interviews (IDIs) will be carried out to understand individual perspectives of participants and focus group discussions (FGDs) will be used to capture group perspectives. FGDs may include 6 to 10 participants including both males and females, or segregated gender groups. Informed consent will be obtained from the participants for the collection of this data.*<br>*Additionally, treatment preference will be assessed among participants in the CAB LA+RPV LA arm at the timepoints detailed in the Schedule of Activities. A detailed survey on medical resource utilization will also be done at Month 12 at selected sites.*	To capture the acceptability and lived experiences of the study participants.
10.2 Appendix 2: Clinical Laboratory Tests	% Reticulocytes was deleted from RBC indices in the protocol-required safety laboratory assessments.	This test is not routinely performed as a part of complete blood count. Additionally, it is expensive and requires additional investment from the sites. Since it does not add value to the trial endpoints or benefit participant safety or data integrity, this test was not deemed necessary for the study.
Throughout the protocol	"RPV LA+CAB LA" was changed to "CAB LA + RPV LA".	To reflect the preferred order of the drugs as they appear in the study acronym CARES.
Throughout the protocol	Minor grammatical, formatting, or spelling changes were made.	Minor errors were noted

11. REFERENCES

Cabotegravir + Rilpivirine Every 2 Months Is Non-inferior To Monthly: Atlas-2M Study. Abstract Presented at the annual Conference on Retroviruses and Opportunistic Infections (CROI). March 8-11, 2020 | Boston, Massachusetts.

Clinical Study Report TMC278-TiDP6-C209. A Phase III, randomized, double-blind trial of TMC278 25 mg q.d. versus efavirenz 600 mg q.d. in combination with a fixed background regimen consisting of tenofovir disoproxil fumarate and emtricitabine in antiretroviral-naïve HIV-1 infected subjects. Tibotec Pharmaceuticals (24 Oct 2011).

Dolgin, E. Long-acting HIV drugs advanced to overcome adherence challenge. Nat Med 20, 323–324 (2014)..

Dolutegravir (DTG) and the fixed dose combination (FDC) of tenofovir/lamivudine/dolutegravir (TLD). Briefing Note. April 2018.

FDA [homepage on the Internet] FDA approved first two-drug regimen for certain patients with HIV. [Accessed August 24, 2020]. Available from: https://www.fda.gov/newsevents/newsroom/pressannouncements/ucm586305.htm.

FDA [homepage on the Internet]. FDA approved changes to the DOVATO (dolutegravir/lamivudine) product labeling. [Accessed August 24, 2020]. Available from https://www.fda.gov/drugs/human-immunodeficiency-virus-hiv/fda-approved-changes-dovato-dolutegravirlamivudine-product-labeling.

Fernandez C, van Halsema CL. Evaluating cabotegravir/rilpivirine long-acting, injectable in the treatment of HIV infection: emerging data and therapeutic potential. HIV AIDS (Auckl). 2019; 11: 179–192.

GlaxoSmithKline Document Number RM2007/00683/09: GSK1349572 Clinical Investigator's Brochure, Version 09, 09 October 2015.

HIV drug resistance report 2017. Geneva: World Health Organization; 2017. License: CC BY-NC-SA 3.0 IGO.

Kanters S, Vitoria M, Doherty M, Socias ME, et al. Comparative efficacy and safety of first-line antiretroviral therapy for the treatment of HIV infection: a systematic review and network metaanalysis. Lancet HIV. 2016;3:e510–20.

Margolis DA, Brinson CC, Smith GHR, et al. Cabotegravir plus rilpivirine, once a day, after induction with cabotegravir plus nucleoside reverse transcriptase inhibitors in antiretroviral-naive adults with HIV-1 infection (LATTE): a randomised, phase 2b, dose-ranging trial. Lancet Infect Dis. 2015;15(10):1145-1155. doi:10.1016/S1473-3099(15)00152-8.

Nachega JB, Uthman OA, Mills EJ, Quinn TC. Adherence to Antiretroviral Therapy for the Success of Emerging Interventions to Prevent HIV Transmission: A Wake up Call. J AIDS Clin Res. 2013 Oct 22; 2012(Suppl 4): 007. doi: 10.4172/2155-6113.S4-007.

Orkin C, Aratséh K, Hernández-Mora Gorgolas M, et al. Long acting cabortegravir + rilpivirine for HIV maintenance: FLAIR week 48 results. Conference on Retroviruses and Opportunistic Infections; 2019:Abstract 140 Seattle.

Panel on Antiretroviral Guidelines for Adults and Adolescents. Guidelines for the use of antiretroviral agents in HIV-1 infected adults and adolescents. Department of Health and Human Services (DHHS). July 14, 2016. Available at: http://www.aidsinfo.nih.gov/ContentFiles/AdultandAdolescentGL.pdf. Accessed 21 June 2017.

Pau AK, George JM. Infect Dis Clin North Am. 2014 September; 28(3): 371–402. doi:10.1016/j.idc.2014.06.001.

Raimondo G, Allain JP, Brunetto MR, et al. Statements from the Taormina expert meeting on occult hepatitis B virus infection. J Hepatol. 2008 Oct;49(4):652-7.

Swindells S, Andrade-Villanueva JF, Richmond GJ, et al. Long-Acting Cabotegravir and Rilpivirine for Maintenance of HsIV-1 Suppression. N Engl J Med. 2020;382(12):1112-1123. doi:10.1056/NEJMoa1904398.

TMC278 (rilpivirine, oral [EDURANT] and parenteral) Investigator's Brochure. Edition 13. July 2020.

World Health Organization. 2015. "Guidelines For The Prevention, Care And Treatment Of Persons With Chronic Hepatitis B Infection."

World Health Organization. 2017. Global Hepatitis Report, 2017.

World Health Organization. 2021. Consolidated Guidelines on HIV Prevention, Testing, Treatment, Service Delivery and Monitoring: Recommendations for a Public Health Approach.

Wood E, Hogg RS, Yip B, Harrigan PR, O'Shaughnessy MV, Montaner JS. Effect of medication adherence on survival of HIV-infected adults who start highly active antiretroviral therapy when the CD4+ cell count is 0.200 to 0.350×10(9) cells/L. Ann Intern Med 2003; 139:810–6.1.

Woodcock, A, Bradley C. Validation of the revised 10-item HIV Treatment Satisfaction Questionnaire status version (HIVTSQs) and the new change version (HIVTSQc). Value in Health,2006: 9(5) 320-333.

Wu AW, Revicki DA, Jacobson D, Malitz FE. Evidence for reliability, validity and usefulness of the Medical Outcomes Study HIV Health Survey (MOS-HIV). Qual Life Res. 1997 Aug;6(6):481-93.

INVESTIGATOR AGREEMENT

I have read this protocol and agree that in conjunction with the accompanying [Master/ISA] protocol,] it contains all necessary details for carrying out this study. I will conduct the study as outlined herein and will complete the study within the time designated.

I will provide copies of the protocol and all pertinent information to all individuals responsible to me who assist in the conduct of this study. I will discuss this material with them to ensure that they are fully informed regarding the study intervention, the conduct of the study, and the obligations of confidentiality.

Coordinating Investigator (where required):

Name (typed or printed): ____________________

Institution and Address: ____________________

Signature: ____________________ Date: ____________________

(Day Month Year)

Principal (Site) Investigator:

Name (typed or printed): ____________________

Institution and Address: ____________________

Telephone Number: ____________________

Signature: ____________________ Date: ____________________

(Day Month Year)

Sponsor's Responsible Medical Officer:

Name (typed or printed): PPD

Institution: Janssen Research & Development

Signature: electronic signature appended at the end of the protocol Date: ____________________

(Day Month Year)

Note: If the address or telephone number of the investigator changes during the study, written notification will be provided by the investigator to the sponsor, and a protocol amendment will not be required.

Signature

User	Date	Reason
PPD	13-Dec-2023 11:57:46 (GMT)	Document Approval

DocuSign Envelope ID: 96163BD4-4A3C-43D2-85B5-568466F27032

Cabotegravir And Rilpivirine: Efficacy and Safety Study: The CARES Study

A Phase 3b, Randomized, Multicentre, Open-Label Study Evaluating the Efficacy, Safety, and Tolerability of Switching to Long-Acting Cabotegravir Plus Long-Acting Rilpivirine from Current Antiretroviral Regimen in HIV-1 Infected, Virologically Suppressed Adults in Sub-Saharan Africa

Trial Identification: PACTR202104874490818

Statistical Analysis Plan

Version 1.1 Dated 25 May 2023

**Prepared by**	**Designation**	**Signature**	**Date**
Ms Caroline Otike	Statistician	DocuSigned by: Caroline Otike 7C969DB44E19476...	5/25/2023
Mr Joseph Musaazi	Trial Statistician	DocuSigned by: 🖊 C65943E1802E4D5...	5/25/2023
**Reviewed by**	**Designation**	**Signature**	**Date**
Dr Ivan Mambule	Project Lead	DocuSigned by: Ivan Mambule AD039B64976547A...	25/05/2023
Prof Nick Paton	Scientific Lead	DocuSigned by: Nicholas Paton Signer Name: Nicholas Paton Signing Reason: I approve this document Signing Time: 29 May 2023 | 12:17 SAST DAC57715F6BD41C3A78486A47F80F306	29 May 2023 | 12:18 SAST
Authorised by	**Designation**	**Signature**	**Date**
Dr Cissy Kityo Mutuluuza	Chief Investigator		

Amendments made from Version 1.0 are listed in Appendix A

DocuSign Envelope ID: 96163BD4-4A3C-43D2-85B5-568466F27032

Table of Contents

Summary of the Trial 4
1.1 Aims 4
1.2 Primary aim 4
1.2.1 Secondary aims 4
1.2.2 Exploratory aims 4
1.3 Hypothesis 4
1.4 Overall Design 4
1.5 Participants 5
1.6 Intervention groups and Duration 5
1.7 Method of randomization 6
1.8 Sample size calculation 6
1.9 Organization 6
2.0 OUTCOME MEASURES 7
2.1 Primary outcome measure 7
2.2 Key secondary outcome measure 7
2.3 Main secondary outcome measures 7
2.4 Other outcome measures 7
2.5 Additional outcome measures at month 24 7
2.6 Outcomes measured in sub-studies or ancillary studies 7
3.0 ANALYSIS POPULATIONS 8
4.0 DERIVATION OF DATA TO BE ANALYSED 9
4.1 Definition of baseline 9
4.2 Definition of nominal month for measurements of laboratory and specific clinical parameters 9
4.3 Definition of treatment switches 9
4.4 Evaluating virological response: handling missing data and treatment switches 10
4.4.1 Data for the primary outcome 10
4.4.2 Data for sensitivity analyses on the primary outcome 11
4.4.3 Data for analysis of virological response at threshold of 200 copies/ml 12
4.5 Evaluating other outcomes: handling treatment switches and missing data 12
4.6 Definition of confirmed VL failure 12
4.7 Definition of resistance and predicted drug activity 13
4.8 Quality of life 14
4.9 Timing of events counted in the analysis 14
4.10 Counting of events 14

DocuSign Envelope ID: 96163BD4-4A3C-43D2-85B5-568466F27032

4.11 Definition of Censoring **15**

4.12 Classification of body mass index **15**

5. STATISTICAL ANALYSIS ***15***

5.1 Interpretation of multiple comparisons **15**

5.2 Enrolment **16**

5.3 Baseline characteristics **16**

5.4 Description of follow-up, completeness **17**

5.5 Antiretroviral therapy received **17**

5.6 Efficacy **18**

5.6.1 Analysis of the primary efficacy outcome 18

5.6.2 Sensitivity analyses of the primary outcome 18

5.6.3 Subgroup analyses of the primary outcome: virological response 19

5.6.4 Analysis of the key secondary outcome: confirmed virological failure 19

5.6.5 Analysis of main secondary and other outcome measures 20

5.7 Safety analyses **20**

5.7.1 General adverse events reported by sites 20

5.7.2 Laboratory safety outcomes, specifically measured 21

5.7.3 Injection site reactions 22

5.8 Sub-study analyses **22**

5.9 Interim analysis **22**

6.0 References ***24***

DocuSign Envelope ID: 96163BD4-4A3C-43D2-85B5-568466F27032

Summary of the Trial

1.1 Aims

1.2 Primary aim

To demonstrate the non-inferior antiviral activity of switching to LA administered every 2 months compared with continuation of cART administered daily over 12 months in HIV-1 infected participants in a resource limited setting.

1.2.1 Secondary aims:

1. To demonstrate the antiviral and immunologic activity of switching to LA administered every 2 months compared to continuation of cART over 12 and 24 months of follow-up.
2. To evaluate the safety and tolerability of switching to LA every 2 months compared to continuation of cART.
3. To assess viral resistance in participants experiencing protocol-defined confirmed virologic failure (plasma HIV-1 RNA ≥200 c/mL).
4. To assess the incidence of on-treatment genotypic resistance to CAB, RPV and other on-study cART up to Month 12 and 24.
5. To evaluate adherence to treatment.

1.2.2 Exploratory aims:

1. To evaluate the effects of LA every 2 months on BMI over time compared to continuation of cART over time
2. To evaluate the effect of LA on trunk fat compared with cART
3. To explore the effect of patient characteristics (eg, demographic factors, baseline disease characteristics) on the virologic response/non-response and immunologic response to LA compared to continuation of cART
4. Retrospective analysis of archived resistance and virological outcomes using PBMCs at baseline.
5. To evaluate the effect of LA on well-being and health status.
6. To evaluate the effect of LA on quality of life.
7. To assess participant satisfaction with the injectable intervention.
8. To assess preference for LA compared to oral cART.
9. To evaluate pharmacokinetics in women who become pregnant on LA.
10. To evaluate health resource utilization among participants in the study.

1.3 Hypothesis

The antiviral effect of switching to LA is non-inferior (margin -10%) to continuation of first-line treatment with daily triple drug oral cART at Month 12 in HIV-1 infected, virologically suppressed participants.

1.4 Overall Design

CARES is a randomized, open-label, active-controlled, multicentre, interventional study in virologically suppressed (<50 c/mL) HIV-1 infected adult participants to demonstrate that the antiviral effect of switching to LA is non-inferior to continuation of first-line cART containing 2 nucleoside reverse transcriptase inhibitor (NRTIs; tenofovir [TDF] plus either lamivudine

[3TC] or emtricitabine [FTC]) plus an INI (dolutegravir [DTG]) or a non-nucleoside reverse transcriptase inhibitors (NNRTI) (efavirenz [EFV] or nevirapine [NVP]) at Month 12.

1.5 Participants

The trial will enrol 512 HIV-1 infected adults on a stable ARV regimen (TDF plus either 3TC or FTC, plus an INI [DTG] or a NNRTI [EFV or NVP] per local country guidelines) for at least 6 months prior to screening, and who are virologically suppressed (plasma HIV-1 RNA levels <50c/mL), with documented evidence of:

- Viral load <50 c/mL at screening AND
- Viral load <50 c/mL on a test preceding screening (within 4-12 months prior to
- screening) AND
- No two consecutive viral load tests ≥50 c/mL in the 12 months prior to screening AND
- No prior history of virologic failure (defined as a confirmed plasma HIV-1 RNA measurement >200 c/mL) at any time.

Patients who have poor adherence to therapy, require concomitant medication with known major interactions with study drugs, or women who are pregnant, breastfeeding or unwilling to use effective contraception will not be enrolled. For more details refer to section 5 of the protocol

Participants will be recruited across 8 sites from 3 countries (3 sites in Uganda, 3 sites in Kenya and 2 sites in South Africa) and followed up on treatment period of 24 months.

1.6 Intervention groups and Duration

Patients will be randomized on a 1: 1, to either continue cART (control arm) or to discontinue cART and begin therapy with LA (research arm) administered every 2 months. The interventions received are as follows;

cART Group: Participants will take a regimen of 2 NRTIs (TDF 300 mg + [3TC 300 mg/FTC 200 mg]) + DTG (50 mg)/EFV (600 mg)/NVP (200 mg), as a single tablet or Fixed-Dose Combination regimen as per local country guidelines up to Month 24. Participants will be permitted to switch cART drugs in case of toxicity or for treatment optimization and convenience after viral load testing.

CAB LA+RPV LA Group (LA): The participants who opt for the OLI Phase will receive the study intervention in 2 phases:
Oral Lead-in Phase: Starting on Day 1, participants will receive RPV 25 mg + CAB 30 mg once daily for 4 weeks to be taken at approximately the same time each day with a meal. The purpose of the optional OLI Phase is to allow an opportunity, when desired, for participants to assess tolerability of the combination prior to administration of CAB LA+RPV LA.

Maintenance Phase: After the 4-week OLI Phase, participants will return for the Month 1 visit to take the last dose of oral CAB+RPV at the study site, and to receive the first IM RPV LA 900 mg + CAB LA 600 mg initiations injections. The second initiation injections with RPV

LA 900 mg + CAB LA 600 mg will be administered at Month 2, and then continuation injections will be administered every 2 months thereafter.
Participants who opt for direct LA (ie, without the OLI Phase) injections will remain on cART for 4 weeks after randomization on Day 1, and will receive the first initiation injection of LA at the Month 1 visit. The second initiation injections with LA will be administered at Month 2, followed by continuation injections of LA every 2 months thereafter.

1.7 Method of randomization

Participants will be randomly assigned to 1 of 2 intervention groups based on a computer-generated randomization schedule prepared before the study. The randomization will be balanced by using randomly permuted blocks stratified according to the third agent class (NNRTI or INI) at screening. The randomization will be balanced by using random permuted blocks.

1.8 Sample size calculation

A sample size of 512 (256 per arm) is required for demonstrating non-inferiority of the LA group versus cART group on the proportion of participants with a virologic response (plasma HIV-1 RNA <50 c/mL) at month 12 (the primary comparison), with the following parameters and assumptions:

1. Estimated 94% virologic response rate in each group.
2. 10% non-inferiority margin
3. 2-sided $\alpha = 0.05$ and 90% power.

The null hypothesis is that LA is non-inferior to cART (non-inferiority margin -10%) in the proportion of participants with plasma HIV-1 RNA <50 c/mL.

For the key secondary hypothesis of non-inferiority of LA in the proportion of participants with virologic failure, this sample size will provide 85% power at a 2-sided 5% significance level with a non-inferiority margin of 4% and assumption of 1.7% virologic failure in a hierarchical testing procedure. At Week 48, the virologic response rate 94% and virologic failure rate 1.7% were observed on Q8W group from the ATLAS-2M study. Therefore, 256 participants per group with total 512 participants are considered adequate for testing the primary and key secondary hypotheses.

1.9 Organization

Sponsor: Joint Clinical Research Centre, Wakiso, Uganda
Uganda Main Funder: Janssen
Coordinator: Joint Clinical Research Centre, Wakiso, Uganda

DocuSign Envelope ID: 96163BD4-4A3C-43D2-85B5-568466F27032

CARES

2.0 OUTCOME MEASURES

2.1 Primary outcome measure
- Virological response (plasma HIV-1 RNA <50 copies/mL) at Month 12

2.2 Key secondary outcome measure
- Confirmed virological failure ≥200 copies/mL by Month 12

2.3 Main secondary outcome measures
- Virological non-response (plasma HIV-1 RNA ≥50 copies/mL) at Month 12
- Virological response (plasma HIV-1 RNA < 200 copies/mL) at Month 12
- Confirmed virological failure ≥200 copies/mL with new genotypic drug resistance mutation by Month 12

2.4 Other outcome measures
- CD4+ lymphocyte count change from baseline at Month 12
- Adherence to treatment through Month 12
- Change from Baseline in BMI at Month 12
- Change from baseline in EQ-5D-5L scores at Month 12
- Change from baseline in MOS-HIV at Month 12
- Change from baseline in HIVTSQ scores at Month 12
- Treatment preference (assessed in LA arm only) at Month 12
- Adherence to treatment through Month 12

Safety
- Injection site reactions (assessed in LA arm only)
- New AE of Grade 3 or higher severity by Month 12.
- New AE leading to discontinuation of treatment by Month 12
- Change in selected laboratory parameters by Month 12
- Disease progression event (new serious non-AIDS or AIDS or death) by Month 12

2.5 Additional outcome measures at month 24
The same set of outcomes (2.1 to 2.5) will be evaluated at Month 24

2.6 Outcomes measured in sub-studies or ancillary studies
- Health economics
- Body composition measurements by DEXA
- Anti-retroviral plasma concentrations during pregnancy
- Social science studies conducted to understand patient preferences

DocuSign Envelope ID: 96163BD4-4A3C-43D2-85B5-568466F27032

3.0 ANALYSIS POPULATIONS

The intention-to-treat exposed (ITT) population comprises all randomized patients who received at least one dose of study intervention (whether LA taken as oral dosing or injection, or any antiretroviral drug post-randomisation in the cART arm). Patients in the ITT population will be analysed according to the group to which they were randomized.

The per-protocol (PP) population comprises all the participants in the ITT population above, with the exclusion of patients that have:

(i) Experienced one or more episodes of duration > 7 days without adequate ART cover:
   – delayed LA injection by > 14 days after scheduled injection, discounting days on which oral bridging was given
   – missing >7 consecutive days of oral bridging treatment
   – missing >7 consecutive days of cART

(ii) Taken concomitant medication for more than 7 days that is known to reduce levels of study medication to an extent that would be expected to have clinical impact and that is stated as a contraindicated medication for this reason in the SPC or IB

(iii) Other formally-diagnosed medical condition that would be expected to have a significant effect on ingestion, absorption or metabolism of one or more of the study medications and reduce drug levels to an extent that would be expected to have clinical impact and that is stated as a caution or contraindication for this reason in the SPC or IB.

The ITT population will be the primary population used for all analyses. The PP will be used for sensitivity analyses of viral load efficacy outcomes (see section 5.6). The decision to perform all analyses (including that for the primary outcome) on the intention-to-treat population (rather than on the per-protocol population) is because the aim of this trial is to look at treatment effectiveness for long-acting injectable ART delivered in programme settings. It is vital to include adverse (or good) outcomes that are associated with deficiencies in treatment delivery or with participant risk factors for adverse outcomes (recognised or unrecognised) in order to properly interpret the performance of the intervention in the target population. The ITT population will give the answer that is the easiest to interpret and is most relevant for the programme setting.

Although a PP population has traditionally been regarded as more conservative for non-inferiority trials, the CONSORT guidelines extension for non-inferiority trials takes a neutral position, allowing for either intention-to-treat or per-protocol or both populations to be used as the basis for concluding non-inferiority (Piaggio et al. 2012). Recent convention for HIV non-inferiority drug trials has been to base the main conclusions on the intention-to-treat population (Hernandez et al. 2013). Thus, this trial will follow the current convention and base determination of non-inferiority for the primary outcome on the analysis of the intention-to-treat population alone; the analysis on the per-protocol population will not affect the overall determination of non-inferiority.

4.0 DERIVATION OF DATA TO BE ANALYSED

Time will be measured from randomization. Time to event analyses will include follow-up until 12 and 24 months (end of trial follow-up).

4.1 Definition of baseline

Baseline values used to define changes in laboratory (e.g., CD4 count) and other measurements (e.g., body weight) over time for each participant are defined as the nearest measurement to randomization date +1 and within 42 days prior (i.e., day -42 to day +1).

4.2 Definition of nominal month for measurements of laboratory and specific clinical parameters

A month is defined as a period of 4 weeks (28 days). Therefore, Month 12 is 48 weeks from randomisation and Month 24 is 96 weeks from randomisation. Laboratory measurements, and other clinical parameters (e.g., weight), at any nominal month of a scheduled visit are defined as those taken nearest to the nominal month at which a measurement is scheduled to be done. The midpoint between two scheduled visit months at which a measurement is due should be taken as belonging to the latter visit window (see below).

Where there are two measurement values within the window around a nominal visit month, but both equidistant from the nominal visit month, the later value will be used.

For the special case of the month 24 visit, the upper limit of the visit will extend for 6 weeks (i.e., to week 102 from randomisation).

4.3 Definition of treatment switches

For the purposes of FDA snapshot algorithm the following will count as switches in treatment:

- Introduction of a new drug, from a different class from the initial regimen prescribed at randomisation
- Switch from LA to cART

Temporary changes, lasting 31 days or less, with return to the previously prescribed regimen will not count as switches.

Change from cabotegravir and rilpivirine injectible to oral bridging or back again will not count as a switch.

Changes within classes within the cART arm will not count as switches.

As described in section 3 above, analysis will be according to intention-to-treat, i.e. treatment substitutions and treatment switches will be ignored in deciding the treatment group for the primary analysis. The intention of defining treatment changes here is merely to enable a description and comparison of antiretroviral therapy received in each randomised group and

for the purposes of determining who has switched for the purposes of the FDA snapshot analysis.

4.4 Evaluating virological response: handling missing data and treatment switches

4.4.1 Data for the primary outcome

A modified FDA snapshot algorithm will be used to determine the primary outcome of virological response at month 12, as well as for other timepoints at which viral load is measured. The approach to classification and presentation of data is given below.

1. The window for the Month 12 visit for viral load is the period between month 9 and month 15

2. The month 12 VL test will be based on the value in this window that is closest to the nominal visit month (ignoring any other values within the window). If two values within the window are equidistant to the nominal (on either side of month 12) visit week, the later (later date) value will be used unless the patient has switched treatment between the earlier and later values, in which case the earlier value will be used. Where the viral load has been repeated *on the same sample* at month 12 because the initial viral load value was between 50 and 200 copies/ml (≥ 50 copies/ml and ≤ 200 copies/ml), the value for this sample will be set to < 50 copies/ml if the repeat test yields a value of < 50 copies/ml; and will be retained at the value obtained on the original test if the value on the repeat test is ≥ 50 copies/ml.

3. The month 12 viral load will be classified as VL < 50 copies/ml (virologic response), VL ≥ 50 copies/ml (virologic non-response), or as "no data in window".

4. If ART was switched before the Month 12 VL test for any reason, including toxicity (see definition of switch in section 4.3 above; note that switch in ART for toxicity is different from withdrawal from trial follow-up due to toxicity, covered in 5, below);
   OR
   If there is no month 12 VL test and ART was switched at any time before the close of the month12 window:

   Classify with reference to the timing and result of the last VL test done on or prior to the switch:
   a) If there was no VL done at or after Month 6 visit (i.e. after day 84; the day when the analysis window opens for the Month 6 visit) and prior to the switch:
      Classify according to the Month 12 VL, as in 3 above (if there is no month 12 VL, classify as no virological data in window)
   b) If the last VL test prior to the switch was done at or after month 6 (i.e. after day 84) and the result was ≥ 50 copies/ml:
      Classify as VL ≥ 50 copies/ml, irrespective of the result within the month 12 visit window.
   c) If the last VL test prior to the switch was done at or after month 6 (i.e. after day 84) and the result was < 50 copies/ml:

Classify according to the value of the month 12 VL (if there is no month 12 VL, classify as “no virological data in window”)

5. If the patient withdrew from the trial (defined by formal declaration by the patient); or was lost- to-follow up (defined as repeated failure to return for scheduled visits and is documented as unable to be contacted by the study site) or is known to have moved away and had their last study visit before the month 12 VL test (or before the close of the month 12 window if there is no month 12 VL test) and did so for reasons other than adverse event or death (which are handled as in 6 below):
   Classify according to the result of the last VL test done at or prior to the last study visit before withdrawal or lost-to-follow-up occurred:

   a) If there was no VL done at or after month 6 (i.e. after day 84) and prior to the date withdrawal: Classify as “No VL data in window, withdrew from trial for other reasons” (as per 6 below)
   b) If the last VL test prior to withdrawal was done at or after month 6 (i.e. after day 84) and the result was ≥ 50 copies/ml:
      Classify as VL ≥ 50copies/ml, irrespective of any result within the month 12 visit window.

   c) If the last VL test prior to withdrawal was done at or after month 6 (i.e. after day 84) and the result was < 50 copies/ml:
      Classify as “No virological data in window, withdrew from trial for other reasons” (as per 6 below)

6. If there is no month 12 VL result within the month 12 visit window, and if there has been no switch in ART following a previous VL ≥ 50 copies/ml (as in 4b, above) or no withdrawal or loss to follow up following a previous VL ≥ 50 copies/ml (as in 5b, above):
   Classify as “No virological data in window” under the following sub-headings:

   a) “Withdrew from the trial due to adverse event/death”:
      This is irrespective of the VL results at or before the time of withdrawal; i.e. even if VL was ≥ 50 copies/ml on last test prior to discontinuation/withdrawal for adverse event/death the patient would still be classified under this category

   b) “Withdrew from trial for other reasons”:
      Including withdrawal of consent, lost to follow-up, moved away, and reasons other than adverse events/death; classify in this category only if the VL prior to withdrawal was < 50 copies, or not done (as in 5 above); if VL prior to withdrawal was ≥ 50 copies, classify month 12 VL as ≥ 50 copies, not under this category.

   c) “On trial, missing data in window”
      Has not withdrawn from trial but does not have data in the window

4.4.2 Data for sensitivity analyses on the primary outcome

For additional sensitivity analyses for the primary outcome of virological response at month 12 (see section 5.6), the following alternative approaches to handling missing data will be used:

DocuSign Envelope ID: 96163BD4-4A3C-43D2-85B5-568466F27032

CARES

- A complete case analysis, in which only observed values of VL at month 12 will be used

- An analysis with imputation of missing (i.e., not observed) viral load values at month 12 using multiple imputation chained estimating equations (MICE). Viral load shall be imputed as a binary outcome categorised as < 50 or 50 copies throughout the imputation. Site, study arm, viral load (last observed value prior to month 12) and any other baseline factor associated with missing values on viral load at month 12, shall be used as potential auxiliary variables in the multiple imputation models. Number of imputations used will be based on the proportion of missing data.
  This sensitivity analysis using imputation will only be done if the proportion of participants with missing VL values at month 12 exceeds 5% of the total population in the ITT analysis.

4.4.3 Data for analysis of virological response at threshold of 200 copies/ml

For the analyses of viral load below 200 copies/ml (secondary outcome), month 12 values will be obtained using the snapshot algorithm approach described in 4.4.1 above, with the alternative threshold replacing the 50 copies/ml threshold in the algorithm throughout.

4.5 Evaluating other outcomes: handling treatment switches and missing data

Analyses other than virologic response (at month 12 and 24) will be based on observed data only i.e. will use complete case analysis. Treatment switches will be ignored. If more than 10% of data are missing on specific outcomes, then predictors of missing data will be explored. The strategy would be to use sensitivity analyses to provide plausible bounds for effect estimates under different missingness mechanisms, rather than to provide a single estimate of effect. As stated in section 3, patients will be analyzed according to the group to which they were randomized.

4.6 Definition of confirmed VL failure

Confirmed virological failure ≥ 200 copies/ml by month 12, the key secondary outcome, will be defined for the purposes of statistical analysis as the occurrence of two consecutive plasma HIV-1 RNA levels ≥ 200 copies/ml taken at least 4 weeks (28 days) and no more than 32 weeks (224 days) apart. The initial value for the rebound episode must be from a test performed at or after month 6 (at or after day 84, when the scheduled month 6 visit analysis window opens).

For the purposes of determining whether VL rebound has occurred, results will be evaluated independent of the visit month (i.e., the initial and confirmatory VL results can be within the same study visit window but not those before day 84).

The definition of "consecutive" will permit one intervening VL that is ≥ 50 and < 200 copies/ml, provided that the immediately preceding test and immediately following test have values ≥ 200copies/ml. A VL result < 50 copies/ml will terminate the episode for the purposes of meeting the definition of VL rebound; counting will begin again with the next detectable VL ≥ 200 copies/ml.

The date of confirmed virological failure will be taken as the date of the first VL result in the episode that has a value ≥ 200 copies/ml (not the date of the subsequent VL test that was used to confirm the rebound), and this will be allocated to the nearest scheduled visit month.

To count towards the outcome "confirmed virological failure ≥ 200 copies/ml by month 12" the date of this first VL must be at or before the date of the test used as the VL value for the month 12 visit (i.e. the one closest to the nominal visit month, as described in section 4.2, above) or before the opening of the month 12 visit (if there is no VL within the month 12 visit window).

The same principles will be followed to define confirmed virological failure by month 24

Note that these definitions for the purposes of statistical analysis of outcomes differ from the recommended definitions in the protocol for clinical management that require that confirmatory VL test is done 6 weeks (window 4-6) or 12 (10-16) weeks after the initial test in the LA and cART arms respectively; that do not allow for intervening VL results ≥50, <200 in the definition of consecutive VL rebound; and allow for a third VL test to be performed to confirm rebound prior to switch of ART in the cART arm.

4.7 Definition of resistance and predicted drug activity

Major resistance mutations and intermediate-high level resistance to study drugs (secondary outcomes) will be defined according to the most current IAS list of mutations and the Stanford database algorithms respectively at the time of analysis.

Minor mutations will be presented in descriptive analyses and will be defined as those which appear both in the most current IAS list of minor mutations and are also listed on the most current WHO list for surveillance of transmitted drug resistance (for RT and PRO) or comparable list for INSTI mutations(Tzou et al. 2020), in order to exclude mutations that have been identified as polymorphisms for any subtype.

Resistance mutations observed on tests performed following VL rebound during the trial will be compared with the viral sequence obtained at baseline (archived, tested in stored PBMCs) to classify the mutations as acquired or present at baseline. In the case where there is no viral sequence available at baseline, each resistance mutation detected during the trial will be classified as presumed present at baseline if there is any record of pre-trial exposure to any those drugs with which that resistance mutation is associated (whether as a known signature mutation of that drug or as a mutation that causes cross-resistance to that drug); if not classified as presumed present at baseline, it will be classified as presumed acquired.

Intermediate/high level resistance at the time of viral load rebound will be assessed based on the resistance test performed at that time (i.e. will not subtract or add any resistance mutations detected at baseline).

4.8 Quality of life

Patient responses on the MOS-HIV questionnaire will be converted to scores on 11 subscales, ranging from 0 to 100, with higher scores indicating better health. The scores are then synthesized into a physical health summary score (PHS) and a mental health summary score (MHS).

4.9 Timing of events counted in the analysis

"Start date" indicated on Adverse Event Log will be used as the date of occurrence for adverse events.

Adverse events or other conditions that have a start date prior to randomisation will be regarded as pre-existing and not included in the analysis of incident events in the trial.

Adverse events that have a start date on the day of randomisation will not be regarded as pre-existing and will be included in the analysis of incident events in the trial, because it is possible for an AE on the day of randomisation to be causally related to randomised group.

Disease progression events (HIV-associated conditions, AIDS) that have a start date on the day of randomisation will be regarded as pre-existing (and not included in incident events) unless there is an adverse event component (i.e. it is also classified as a Grade 3 or 4 event; or SAE with start date on or after the date of randomisation).

All deaths that occur on or after the date of randomisation and before the end of the trial follow-up period appropriate for the analysis (ending at the month 12 trial visit, or close of month 12 visit window if there is no visit within the month 12 window; similarly for month 24, ending at week 106 if there is no visit within the month 24 window, as in section 4.2) will be included trial events, regardless of the date of diagnosis of the cause of death. Deaths that occur after the month 12 (or month 24) trial visit, but that are caused by progression of a condition that was present at the Month 12 trial visit (e.g. a malignancy), will be counted as trial deaths if the date of death is before the close of the Month 12 or 24 analysis window.

The "date of death" on End of Study Treatment form will be used as the date of occurrence for deaths.

An additional category of treatment-emergent adverse events will be defined for participants in the LA arm only. These will comprise AEs occurring at or after the initial administration of study intervention (LA, oral or injection) up to the day of last dose of LA treatment (oral or injection) plus 30 days. AEs with onset date on the day of randomisation will be automatically included, unless they are known to have occurred prior to the first dose of study medication. This category will be used for descriptive purposes in the LA arm. It will not be used for the purposes of comparison of AEs in the LA and cART arm as there is no comparable definition for cART which is continuous throughout the trial.

4.10 Counting of events

Adverse Events are defined for this protocol as any new, recurrent, or increased severity (on DAIDS grading scale) of a disease, symptom, physical sign or a clinically-significant

DocuSign Envelope ID: 96163BD4-4A3C-43D2-85B5-568466F27032

laboratory abnormality. The definition of clinically-significant laboratory abnormality is one that:

a) Suggests underlying disease and/or organ toxicity that is worsening and/or
b) Requires additional active management e.g. change of dose, discontinuation of drug, close observation, more frequent follow-up assessments or further diagnostic investigation.

AEs are coded using the Medical Dictionary for Regulatory Activities (MedDRA) using the verbatim terms reported in the CRF by investigators.

Non-serious adverse events will be analysed separately, according to each graded component. An event that increases in grade will be analysed as a single event at the higher grade but with the onset date as the date the event started.

SAEs will be analysed as episodes, with all components of the same clinical SAE presented as one episode (eg "Anaemia + renal failure").

Death from an unknown cause will be analysed as a grade 5 event. Where cause of death is known this will be counted as a single grade 5 event with the name of the underlying event. (i.e. not double counted as death and the underlying event as separate events). If the cause of death is a grade 3 (or lower) event, it will be upgraded to a grade 5 event because it has led to death; and will be counted as above.

4.11 Definition of Censoring

For time to event analyses and calculation of rates of deaths, last contact with clinic staff will be the date of censoring for patients who have not died. For all other time to event analysis and rate calculations, the last clinic visit with be the date of censoring of patients because we cannot be sure that no other events occurred unless they visit the clinic.

4.12 Classification of body mass index

Body mass index will be classified into the following categories: <18.5 (underweight); 18.5 to<25 (normal); 25 to <30 (overweight); ≥30 (obese)

5. STATISTICAL ANALYSIS

5.1 Interpretation of multiple comparisons

For the primary endpoint treatment comparison at month 12, no adjustment for multiple comparisons is necessary for testing non-inferiority followed by superiority since testing follows a pre-specified sequence of hypotheses such that if the first hypothesis tested is not significant, the subsequent tests will not be performed. This fixed sequence procedure controls the type I error rate at the nominal level.

No formal adjustment for multiple testing on other comparisons will be made, but all significance tests will be interpreted in the context of the total number of comparisons performed. The study report will include a statement of the number of significance tests performed for subgroup analyses of the primary outcome, for secondary outcomes and for

exploratory outcomes along with a statement of the number of statistically significant results that would be expected to occur by chance alone, following the approach recommended by Wang et al (NEJM 2007).

5.2 Enrolment

The following shall be presented in a table as frequency and percentages (all participants, not split by treatment arm).

- Screening and randomization by calendar time
- Total screened and randomized by site and the baseline third agent class (NNRTI or INI) strata, with dates of first and latest randomization
- Reasons for not randomizing
- Eligibility: number and reasons for ineligibility and number of patients randomized in error and excluded according to the protocol section 5.2.
- Time between screening and enrolment visit in those randomized; median (IQR), range, distribution into categories; <2, 2-<4, 4-<6, 6-<8, > 8

5.3 Baseline characteristics

Tabulations by treatment arm and with a “total” column will be produced for the following baseline characteristics

- sex: number (%) male, female, undifferentiated, unknown
- Number of women of childbearing potential (if woman): number (%)
- Women not of childbearing potential (reason): number (%) Post-Menopausal, Premenarchal, Surgically Sterile, Other
- age at last birthday: median (IQR), range, distribution into categories: < 35, 35-49, ≥50 years
- Study site
- Country of birth: Uganda, Kenya, South Africa, other
- Race of subject: number (%) Black, White, Asian, Coloured, Other race, Not reported/Unknown.
- CD4 count at screening: mean (SD), median (IQR), range, distribution into categories; 0-49, 50-199, 200-349, ≥ 350 cells/mm3
- Baseline viral load ≥50 copies/ml
- Weight: mean (SD), median (IQR), range
- BMI (male, female separately): mean (SD), median (IQR), range; BMI distribution by category: <18.5, 18.5 to <25, 25 to <30, ≥30
- Years since started combination ART: median (IQR), range, distribution into categories; 1-2, 3-4, 5+.
- ART Drugs (ever) received prior randomization (tenofovir, TAF, lamivudine, emtricitabine, zidovudine, stavudine, didanosine, abacavir, efavirenz, nevirapine, raltegravir, dolutegravir, bictegravir, lopinavir, atazanavir, darunavir, other): n (%) for each drug.
- Blood results at screening: haemoglobin, neutrophils, platelets, creatinine, ALT, eGFR: mean (SD): median (IQR), range

- Glucose at screening: mean (SD), median (IQR), range; fasted, n (%) and distribution into categories < 5.6, 5.6-6.9, ≥ 7mmol/L; non-fasted, n(%) and distribution into categories < 11.1 mmol/L, ≥ 11.1 mmol/L; not done
- CVD risk factors:
  - history of CVD/stroke,
  - family history of CVD/stroke,
  - history of diabetes,
  - taking blood pressure medication,
  - smoking status (Never, current, ex): n (%)
- Health status (EQ-5D-5L scores)
- MOS-HIV scores (physical and mental function summary scores)
- HIVTSQs score: total score
- Viral subtype (from PBMC measurements): A, C, D, Other, n, (%)
- NRTI, NNRTI and INSTI resistance (detected from PBMC)

5.4 Description of follow-up, completeness

- Number died before Month 12, Month 24 follow up visit
- Number formally withdrawn consent before Month 12, Month 24 follow-up visit
- Number not seen in clinic in the 8 months prior to Month 12, Month 24
- Number of scheduled study visits completed overall, and by assessment week, as absolute number and percentage (excluding from denominator those who had died, formally withdrawn, or lost-to-follow up before each visit week).

5.5 Antiretroviral therapy received

- ART prescribed at randomization n(%) on each regimen
- Oral Lead-in (OLI) Phase (for LA arm only): number (%) opted for a 4-week Oral Lead-in (OLI) Phase.
- Substitutions: number (%) of patients making substitutions, reasons for substitutions, drugs substituted, time to first substitution
- % of time spent (over months of follow up from randomization to last visit; considering periods of interruption > 1 month recorded on ART log) on:
  - Initial prescribed regimen
  - Regimen with within-class substitutions
  - Regimen with other substitutions (switch)
  - not on ART
- Adherence to ART
  - *cART*

- number (%) of attended study visits (excluding baseline) to month 12 and to month 24 where patient reported less than complete adherence (missing at least one dose of cART in the previous 4 weeks)
- number (%) of dispensing episodes (of ≥ 28 days duration) where < 90% of pills required for the interval were consumed

  - *LA*

-Injections administered from pharmacy refill records for LA arm as (%) required

-Injections administered > 7 days from target scheduled date.

5.6 Efficacy

5.6.1 Analysis of the primary efficacy outcome

The primary analysis will test the hypothesis that the LA arm is non-inferior to the cART arm on the primary outcome of virological response (plasma HIV-1 RNA <50 copies/mL) at Month 12 in the intention to treat population.

For comparing LA with cART, the difference between groups (LA minus cART) in proportion of patients with VL<50 copies/ml and a 95% binomial confidence interval for that difference will be calculated (Cochran Mantel-Haenszel Weighted Miettinen & Nurminen Method). All CIs will be two sided and the analysis will be stratified according to the baseline (pre-randomisation) third agent class (NNRTI or INSTI).

Treatment with LA will be considered non-inferior to cART if the lower limit of the binomial-based 95% confidence interval of the difference in efficacy between the treatment groups is a higher value than -10%.

5.6.2 Sensitivity analyses of the primary outcome

Additional sensitivity analyses for the primary outcome will include the following:

- Stratified analysis including adjustment for the third agent class (as for the main analysis) but with additional adjustment for site, gender and screening CD4 count (< 200 or ≥ 200 cells/mm3) (factors which commonly affect outcomes in HIV treatment trials).
- Per-protocol analysis, based on the modified FDA snapshot algorithm and done in the per-protocol population (as defined in section 3).
- Analysis using imputation of missing viral load data; done in the intention-to-treat population (only if more than 5% missing data), as described in section 4.4.2
- Complete case analysis in which only patients in the intention-to-treat population with observed values of viral load at month 12 will be included in the analysis.

Non-inferiority will be decided based on the analysis in the ITT population alone and analysis of the per-protocol population will not affect the overall determination of non-inferiority (see section 3). However, non-inferiority will need to be shown on both populations before proceeding to test for superiority. If both the main intention to treat analysis and the per-protocol analysis show non-inferiority of the LA group, then the hypothesis that LA is superior to the standard-of-care cART group will be tested on the intention-to-treat population and modified FDA snapshot algorithm, with superiority declared if the lower limit of the 95% confidence interval for the difference between groups is above 0%. If superiority is tested then a P value from chi-squared test will be presented.

5.6.3 Subgroup analyses of the primary outcome: virological response

Subgroup analyses will be performed for the LA versus cART comparison to assess heterogeneity of differences between randomized groups, using the same intention-to-treat population and modified FDA snapshot algorithm used for the primary comparison (with omission of patients from individual subgroup analyses if they lack data for classification in that subgroup).

Results will be presented as a forest plot, with percentage virologic response in each subgroup, percentage risk difference and 95% CI and a test for heterogeneity.
We recognise that type I error is elevated by performing multiple tests. As stated in in section 5.2, a statement about the number of significant findings that would be expected by chance alone will be included with the results.

The following subgroups are pre-specified:

- Sex at birth
- Country of enrolment
- Age :<35, 35-49, ≥50 years
- Screening CD4+ cell count (< 200 or ≥ 200 cells/mm^3)
- BMI (< 30kg/m^2; ≥ 30mg/m^2)
- Baseline major resistance mutations to rilpivirine
- Baseline intermediate-high level resistance to rilpivirine (Stanford algorithm, section 4.8)
- Baseline intermediate-high level resistance to cabotegravir (Stanford algorithm, section 4.8)
- Prior use of any NNRTI
- Prior use of any INSTI
- HIV-1 subtype (A6/A1 vs other)

A pre-specified subgroup comparison listed above will not be performed, unless of special interest, if there are fewer than 30 patients in a subgroup category within each of the two arms that are being compared. In such cases, the subgroup may be omitted; or thresholds may be modified or subgroup categories consolidated to achieve the minimum number of participants in a subgroup. Where revised thresholds are chosen, these should be justified by scientific and/or clinical rationale.

The subgroup analysis may be repeated for the per-protocol population.

Additional exploratory multivariable logistic regression analyses of predictors of virological suppression may be performed to assess the independent effects of a number of factors on the attainment of virological response.

5.6.4 Analysis of the key secondary outcome: confirmed virological failure

The key secondary outcome of confirmed virological failure ≥200 copies/mL by Month 12 will also be analysed formally for non-inferiority. However, a hierarchical testing procedure will be used such that formal evaluation for non-inferiority will only be done if non-inferiority has previously been demonstrated on the primary outcome of virological response in the ITT and per-protocol populations.

DocuSign Envelope ID: 96163BD4-4A3C-43D2-85B5-568466F27032

The definition of confirmed virological failure is given in section 4.6

The difference between groups (LA minus cART) in proportion of patients with confirmed virological failure ≥200 copies/mL by Month 12 and a 95% binomial confidence interval for that difference will be calculated. As for the primary outcome, CIs will be two sided and the analysis will be stratified according to the baseline (pre-randomisation) third agent class (NNRTI or INSTI).

Treatment with LA will be considered non-inferior to cART for confirmed virological failure if the upper limit of the 95% confidence interval of the difference is below 4%.

A similar approach to hierarchical testing will be followed to that used for the evaluation of non-inferiority on the primary outcome. Non-inferiority for the outcome of confirmed virological failure will be made solely based on the intention-to-treat analysis. A sensitivity analysis may be done in the per-protocol population if non-inferiority is shown in the ITT population. A test of superiority will be performed only if non-inferiority is demonstrated in both the ITT and PP populations.

Depending on the number of cases of failure observed, additional exploratory multivariable logistic regression analyses of predictors of confirmed virological failure may be performed to assess the independent effects of a number of factors on incident virological failure.

5.6.5 Analysis of main secondary and other outcome measures

Analysis of secondary viral load outcomes will use the same intention to treat approach and population as that described for the primary outcome above, with results expressed as risk differences with 95% CI.

Analysis of other outcomes will follow the intention to treat approach but using complete case analysis. Mean change from Baseline in CD4+ lymphocyte count at Month 12 will be compared between groups using t-tests or analysis of variance. Mean change in BMI from baseline to Month 12 and month 24 will be analysed using a similar approach. Linear mixed effects regression model and repeated ANOVA may be used to compare the two groups over time if substantive differences are seen at Month 12 between the groups. The distribution of body mass index strata (section 4.12) at scheduled visits after randomization will be presented in a figure.

For quality of life analysis, Linear mixed effects regression model will be used to compare the two treatment groups in terms of changes from baseline in the summary PHS and MHS quality of life indexes over the follow-up period.

5.7 Safety analyses

5.7.1 General adverse events reported by sites

Adverse events (AEs) will be tabulated by the 2 randomized drug groups showing number of patients in each group with at least one event of the specified event type, (and % of total patients with at least one event of the specified type) as well as the total number of events of the specified type.

Adverse events will be presented as descriptive data only and no formal statistical comparison between groups will be made. Events will be presented as MedDRA preferred terms, and by System Organ Classes using the standard international order.

The following will be presented:

Serious adverse events (SAEs):
Total and by SAE category (fatal, life-threatening, resulting in prolonged hospitalization, associated with persistent of major disability or incapacity, congenital anomaly or birth defect, or other important medical condition). SAEs presented as total patients with at least one event, % of patients with at least one event, number of events. SAE category presented as number of events only. A separate line listing of SAEs showing event name, study week the event started, treatment group, and an indication of whether the event was considered at least possibly related to the CARES drugs will be provided.

Grade 3 or 4 adverse events (irrespective of relationship to study drug):
Total and by body system. Grade 3/4 events total presented as total patients with at least one event, % of patients with at least one event, number of events. Grade 3/4 events by body system presented as number of events only. A separate line listing of actual event names will be presented under each body system showing number of events with that event name.

Grade 3 or 4 adverse events (considered at least possibly related to a study drug):
Presented as total patients with at least one event, % of patients with at least one event, number of events.

Adverse events of any severity leading to discontinuation of the study regimen
Discontinuation is defined as permanent cessation of at least one of the protocol-mandated study drugs. Presented as total patients with at least 1 event, % of patients with at least one event, number of events; and as a separate line listing of event name, grade, study week when event started; study arm as randomized; study drug(s) discontinued and study week of discontinuation.

Disease progression event:
Defined as any new serious non-AIDS event (INSIGHT criteria), AIDS or death at or following randomisation. Presented as total patients with at least 1 event, % of patients with at least one event, number of events; and as a separate line listing of event name and study week when event started.

5.7.2 Laboratory safety outcomes, specifically measured

Mean change from baseline in the following selected laboratory parameters will also be presented in line charts by arm at time points when protocol-mandated measurements are done:

- Haemoglobin
- Total white cell count,
- Neutrophil count
- Platelets

- Creatinine
- ALT
- Bilirubin
- Alkaline phosphatase
- Glucose
- Cholesterol
- Triglycerides

These parameters will be compared between arms using the same approach used for weight and BMI above (i.e. analysis of variance; linear regression models).

In addition, the proportion of participants with one or more measurements of low haemoglobin (threshold of < 9 g/dl, corresponding to DAIDS Grade 3) and reduced eGFR (threshold of <60 ml/min/1.73m2, corresponding to stage 3 chronic kidney disease) during follow up will be presented by treatment arm.

5.7.3 Injection site reactions

This will be presented as descriptive data in the LA arm only based on responses to the Injection Site Reaction (ISR) questionnaire administered to each participant at the time of each injection. Data will be presented as proportion at each injection time point that report each type of injection site reaction.

5.8 Sub-study analyses

Analysis of outcomes measured in sub-studies will be described in separate plans that will be included as appendices to this SAP when ready. These will cover analyses of:

- Health economics
- Body composition measurements by DEXA
- Anti-retroviral plasma concentrations during pregnancy
- Social science studies conducted to understand patient preferences

5.9 Interim analysis

An interim analysis will be performed once approximately 60% of participants have completed the Month 6 visit.

The IDMC will consider at this interim analysis, whether the LA arm demonstrates clear inferiority to the standard of care arm, based on the proportion of patients with viral load suppression below 50 copies/ml. This is the primary trial outcome assessed at 12 months, although for the interim analysis it will be assessed primarily on 6-month follow-up data. Statistically, this judgement of inferiority should meet the Haybittle-Peto rule based on the 99.9% confidence interval for the difference in viral suppression (i.e. a P value of < 0.001).

Clinically, which should be given equal or greater weight than the statistical threshold in any recommendation to stop the trial, the judgement should be based on the magnitude of the

observed inferiority with the long acting versus the standard of care arm being assessed as sufficiently large as to convince the majority of clinicians and policymakers - with responsibilities for resource-limited settings - that a long-acting therapy regimen would not be a viable treatment option for the public health approach. Such a decision would also need to give due weight to the short duration of follow-up at the time of the interim evaluation that might limit prediction of meaningful long-term outcomes, as well as the limited value of viral suppression below 50 copies as a marker of long-term regimen success. Before making such a recommendation to stop the trial based on inferior viral suppression, the IDMC should also consider the rate of viral suppression at higher thresholds (e.g. 200 copies/ml threshold) that may be less affected by assay random variation, as well as the number of cases of confirmed virological failure at the thresholds of 200 copies/ml that might be considered meaningful for the public health approach.

DocuSign Envelope ID: 96163BD4-4A3C-43D2-85B5-568466F27032

CARES

6.0 References

Hernandez, Adrian v., Vinay Pasupuleti, Abhishek Deshpande, Priyaleela Thota, Jaime A. Collins, and Jose E. Vidal. 2013. "Deficient Reporting and Interpretation of Non-Inferiority Randomized Clinical Trials in HIV Patients: A Systematic Review." *PLoS ONE* 8 (5). https://doi.org/10.1371/journal.pone.0063272.

Piaggio, Gilda, Diana R Elbourne, Stuart J Pocock, Stephen J W Evans, and Douglas G Altman. 2012. "Reporting of Noninferiority and Equivalence Randomized Trials Extension of the CONSORT 2010 Statement." *JAMA* 308 (24): 2596–2604. www.jama.com.

Tzou, Philip L., Soo Yon Rhee, Diane Descamps, Dana S. Clutter, Bradley Hare, Orna Mor, Maxime Grude, et al. 2020. "Integrase Strand Transfer Inhibitor (INSTI)-Resistance Mutations for the Surveillance of Transmitted HIV-1 Drug Resistance." *Journal of Antimicrobial Chemotherapy* 75 (1): 170–82. https://doi.org/10.1093/jac/dkz417.

DocuSign Envelope ID: 96163BD4-4A3C-43D2-85B5-568466F27032

CARES

APPENDIX A SUMMARY OF REVISIONS IN VERSION 1.1

Section	Change	Rationale
**4.4.1**	Added clarification that when the Month 12 VL is repeated on the same sample after an initial result >50 and ≤200 copies/ml, and the repeat test yields a value of < 50 copies/ml, the outcome for that sample will be taken as <50 copies/ml.	To reflect the additional VL testing procedure at M12 that has been adopted to minimise the impact on primary outcome of low-level blips arising from laboratory technical artefact.
**5.6.5**	Edited the proposed analysis method from generalised linear regression model to Linear mixed effects model or repeated ANOVA, for comparing changes over the follow-up period in CD4+ lymphocyte count, PHS and MHS quality of life indexes.	Proposed statistical analysis method that accounts for correlation of outcomes due to repeated measures within the same subject.
