# Supplementary material for: Cabotegravir and rilpivirine for treatment of HIV infection in Africa: week 96 results from the phase 3b randomized, open-label, noninferiority CARES trial
Source: Nat Med. 2025 Nov 4;32(1):168–77. doi: 10.1038/s41591-025-04041-7 (PMC12823427; doi:10.1038/s41591-025-04041-7)
Supplement: Supplementary file 2 — Reporting Summary [file 41591_2025_4041_MOESM2_ESM.pdf]

nature portfolio

Corresponding author(s): Nicholas Paton

Last updated by author(s): Sep 20, 2025

# Reporting Summary

Nature Portfolio wishes to improve the reproducibility of the work that we publish. This form provides structure for consistency and transparency in reporting. For further information on Nature Portfolio policies, see our Editorial Policies and the Editorial Policy Checklist.

## Statistics

For all statistical analyses, confirm that the following items are present in the figure legend, table legend, main text, or Methods section.

| n/a | Confirmed | |
|---|---|---|
| ☐ | ☒ | The exact sample size (*n*) for each experimental group/condition, given as a discrete number and unit of measurement |
| ☒ | ☐ | A statement on whether measurements were taken from distinct samples or whether the same sample was measured repeatedly |
| ☐ | ☒ | The statistical test(s) used AND whether they are one- or two-sided<br>*Only common tests should be described solely by name; describe more complex techniques in the Methods section.* |
| ☐ | ☒ | A description of all covariates tested |
| ☐ | ☒ | A description of any assumptions or corrections, such as tests of normality and adjustment for multiple comparisons |
| ☐ | ☒ | A full description of the statistical parameters including central tendency (e.g. means) or other basic estimates (e.g. regression coefficient) AND variation (e.g. standard deviation) or associated estimates of uncertainty (e.g. confidence intervals) |
| ☐ | ☒ | For null hypothesis testing, the test statistic (e.g. *F*, *t*, *r*) with confidence intervals, effect sizes, degrees of freedom and *P* value noted<br>*Give P values as exact values whenever suitable.* |
| ☒ | ☐ | For Bayesian analysis, information on the choice of priors and Markov chain Monte Carlo settings |
| ☒ | ☐ | For hierarchical and complex designs, identification of the appropriate level for tests and full reporting of outcomes |
| ☒ | ☐ | Estimates of effect sizes (e.g. Cohen's *d*, Pearson's *r*), indicating how they were calculated |

*Our web collection on statistics for biologists contains articles on many of the points above.*

## Software and code

Policy information about availability of computer code

| | |
|---|---|
| Data collection | MediData Rave |
| Data analysis | All analyses were performed with the use of Stata sortware version 16.1(StataCorp), except for the estimate of difference in proportions for the efficacy analysis that was performed in R version 4.3.3. |

For manuscripts utilizing custom algorithms or software that are central to the research but not yet described in published literature, software must be made available to editors and reviewers. We strongly encourage code deposition in a community repository (e.g. GitHub). See the Nature Portfolio guidelines for submitting code & software for further information.

## Data

Policy information about availability of data

All manuscripts must include a data availability statement. This statement should provide the following information, where applicable:

- Accession codes, unique identifiers, or web links for publicly available datasets
- A description of any restrictions on data availability
- For clinical datasets or third party data, please ensure that the statement adheres to our policy

Anonymized individual participant data that underlie the results reported in this article (including data dictionaries) and study documents can be requested for a period of up to 24 months after publication of this paper. An independent review process of data access requests is required under the trial governance structure which formed the basis for local ethics and regulatory approvals. Requests should be sent to Dr Kityo (ckityo@jcrc.org.ug) accompanied by an outline proposal for

the intended analysis and a list of variables required. Requests will be reviewed by and subject to approval of the independent Trial Steering Committee. A response will be provided to the requesting party within a maximum of 2 months of receipt of the request.

## Research involving human participants, their data, or biological material

Policy information about studies with human participants or human data. See also policy information about sex, gender (identity/presentation), and sexual orientation and race, ethnicity and racism.

| | |
|---|---|
| Reporting on sex and gender | Sex was as assigned at birth. We have reported the numbers of each sex in the overall study population (applicable to efficacy and safety analyses); we have reported a pre-specified subgroup analysis of the main outcome by sex; and reported the body mass and metabolic outcomes stratified by sex. |
| Reporting on race, ethnicity, or other socially relevant groupings | The trial was performed in sub-Saharan Africa and all except 2 participants are of Black race. This is noted in the baseline characteristics table. We have not performed any analyses by race or ethnicity. |
| Population characteristics | Reported in the baseline characteristics table. |
| Recruitment | Participants were enrolled from those attending for their routine clinical care at the trial site, or by referral from clinics within the surrounding areas. Screening and subsequent trial procedures were done only at the trial site. We have described the eligibility criteria in full in the trial population section of the methods. The numbers of screen failures are indicated in the CONSORT diagram. Enrolled participants were those who were motivated to switch to long-acting therapy and are not necessarily representative of all those receiving HIV treatment. However, this resembles how the intervention would be used in practice - it would not be given to those who did not want to switch from oral therapy - so this does not represent a bias towards overestimating long acting therapy efficacy as such. Outcomes in those randomised to oral therapy were also good, suggesting that any disappointment at not receiving long acting therapy had little impact. |
| Ethics oversight | The trial protocol was approved by the ethics body responsible for each clinical research site (Joint Clinical Research Centre Research Ethics Committee and the Uganda National Council for Science and Technology, all in Uganda; Moi Teaching and Referral Hospital Institutional Scientific and Ethics Review Committee, Aga Khan University Institutional Scientific and Ethics Review Committee, and Kenya Medical Research Institute Scientific and Ethics Review Unit, all in Kenya; University of The Witwatersrand Johannesburg Human Research Ethics Committee, and South African Medical Research Council Human Research Ethics Committee, all in South Africa); and by national regulatory agencies responsible for reviewing and approving trials in the participating countries. |

Note that full information on the approval of the study protocol must also be provided in the manuscript.

# Field-specific reporting

Please select the one below that is the best fit for your research. If you are not sure, read the appropriate sections before making your selection.

☒ Life sciences ☐ Behavioural & social sciences ☐ Ecological, evolutionary & environmental sciences

For a reference copy of the document with all sections, see nature.com/documents/nr-reporting-summary-flat.pdf

# Life sciences study design

All studies must disclose on these points even when the disclosure is negative.

| | |
|---|---|
| Sample size | The derivation of the sample size for the main trial is described in detail in the statistical analysis section of the manuscript. This sample size was used for the analysis of the main outcomes. For some secondary outcomes, complete case analysis was performed (using all available data, but without imputing missing data); this is explicitly stated where it is applicable. The basis for the sample size for the DEXA substudy is also described in the statistical analysis section. |
| Data exclusions | No data were excluded from analyses, with the exception of the body weight and some metabolic analyses where women who were pregnant at the time of the scheduled assessment were not included in the analysis at that timepoint due to the potential confounding effects of pregnancy. All such data exclusions are specified in the footnotes to the respective tables. Exclusion of data from women during pregnancy from the analyses of body weight and BMI change and from selected metabolic assessments was not prespecified in the analysis plan but is common practice and the rationale is clear. |
| Replication | Replication is not generally performed within a single clinical trial because data collection and analysis is at specific timepoints that cannot be revisited. Results were similar at week 48 and week 96 which provides a degree of reassurance that the trial findings are robust, as does the body of evidence from other clinical trials testing the same intervention in different populations and clinical care systems. |
| Randomization | This was a randomised controlled trial, with all participants analyses according to the group to which they were randomised (intention to treat analysis). Randomisation was performed using a Web-based system, as described in the Randomisation and masking section of the methods. |
| Blinding | The trial was open-label meaning that both the participants and the caregivers were aware of the treatment group allocation. The trial management team at the coordinating centre did not have access to aggregate unmasked data, so effectively were blinded to treatment allocation. |

# Reporting for specific materials, systems and methods

We require information from authors about some types of materials, experimental systems and methods used in many studies. Here, indicate whether each material, system or method listed is relevant to your study. If you are not sure if a list item applies to your research, read the appropriate section before selecting a response.

## Materials & experimental systems

| n/a | Involved in the study |
|---|---|
| ☒ | ☐ Antibodies |
| ☒ | ☐ Eukaryotic cell lines |
| ☒ | ☐ Palaeontology and archaeology |
| ☒ | ☐ Animals and other organisms |
| ☐ | ☒ Clinical data |
| ☒ | ☐ Dual use research of concern |
| ☒ | ☐ Plants |

## Methods

| n/a | Involved in the study |
|---|---|
| ☒ | ☐ ChIP-seq |
| ☒ | ☐ Flow cytometry |
| ☒ | ☐ MRI-based neuroimaging |

## Clinical data

Policy information about clinical studies

All manuscripts should comply with the ICMJE guidelines for publication of clinical research and a completed CONSORT checklist must be included with all submissions.

| | |
|---|---|
| Clinical trial registration | PACTR registration:202104874490818 |
| Study protocol | The full trial protocol is included with the supplementary materials published with this paper. |
| Data collection | The trial was conducted at 8 clinics in 3 countries in sub-Saharan Africa (Uganda, Kenya, South Africa). These sites comprised private and public sector institutions; all had facilities and experience to conduct clinical trials. Participants were screened and enrolled into the trial between 1 September 2021 to 31 August 2022, and each participant was followed up for 96 weeks. The last participant attended their week 96 trial visit on 3 July 2024. |
| Outcomes | Outcomes were pre-defined in the protocol and statistical analysis plan. The main efficacy outcomes are based on plasma viral load that was tested every 24 weeks during the trial: viral load < 50 copies/ml at week 96 (main outcome); confirmed virological failure (two consecutive values ≥ 200 copies/ml) by week 96 (key secondary outcome). Safety was assessed by reported clinical adverse events at each visit, supplemented by blood tests for routine safety parameters at protocol-mandated time points. The main safety outcome was the occurrence of one or more adverse events of at least grade 3 severity. |

## Plants

| | |
|---|---|
| Seed stocks | *Report on the source of all seed stocks or other plant material used. If applicable, state the seed stock centre and catalogue number. If plant specimens were collected from the field, describe the collection location, date and sampling procedures.* |
| Novel plant genotypes | *Describe the methods by which all novel plant genotypes were produced. This includes those generated by transgenic approaches, gene editing, chemical/radiation-based mutagenesis and hybridization. For transgenic lines, describe the transformation method, the number of independent lines analyzed and the generation upon which experiments were performed. For gene-edited lines, describe the editor used, the endogenous sequence targeted for editing, the targeting guide RNA sequence (if applicable) and how the editor was applied.* |
| Authentication | *Describe any authentication procedures for each seed stock used or novel genotype generated. Describe any experiments used to assess the effect of a mutation and, where applicable, how potential secondary effects (e.g. second site T-DNA insertions, mosiacism, off-target gene editing) were examined.* |
